# Global Analysis of Protein *N*-Myristoylation and Exploration of *N*-Myristoyltransferase as a Drug Target in the Neglected Human Pathogen *Leishmania donovani*

**DOI:** 10.1016/j.chembiol.2015.01.003

**Published:** 2015-03-19

**Authors:** Megan H. Wright, Daniel Paape, Elisabeth M. Storck, Remigiusz A. Serwa, Deborah F. Smith, Edward W. Tate

**Affiliations:** 1 Department of Chemistry, Imperial College London, London SW7 2AZ, UK; 2 Centre for Immunology and Infection, Department of Biology, University of York, York YO10 5DD, UK

## Abstract

*N*-Myristoyltransferase (NMT) modulates protein function through the attachment of the lipid myristate to the N terminus of target proteins, and is a promising drug target in eukaryotic parasites such as *Leishmania donovani*. Only a small number of NMT substrates have been characterized in Leishmania, and a global picture of *N*-myristoylation is lacking. Here, we use metabolic tagging with an alkyne-functionalized myristic acid mimetic in live parasites followed by downstream click chemistry and analysis to identify lipidated proteins in both the promastigote (extracellular) and amastigote (intracellular) life stages. Quantitative chemical proteomics is used to profile target engagement by NMT inhibitors, and to define the complement of *N*-myristoylated proteins. Our results provide new insight into the multiple pathways modulated by NMT and the pleiotropic effects of NMT inhibition. This work constitutes the first global experimental analysis of protein lipidation in *Leishmania*, and reveals the extent of NMT-related biology yet to be explored for this neglected human pathogen.

## Highlights

• Alkyne-tagged probes for protein lipidation are applied in *Leishmania donovani*
• A global profile of protein lipidation in two life stages is presented
• *N*-Myristoylated proteome defined by chemical knockdown and quantitative proteomics
• Evidence for NMT as a drug target in the leishmaniases

## Graphical Abstract

**Figure:**
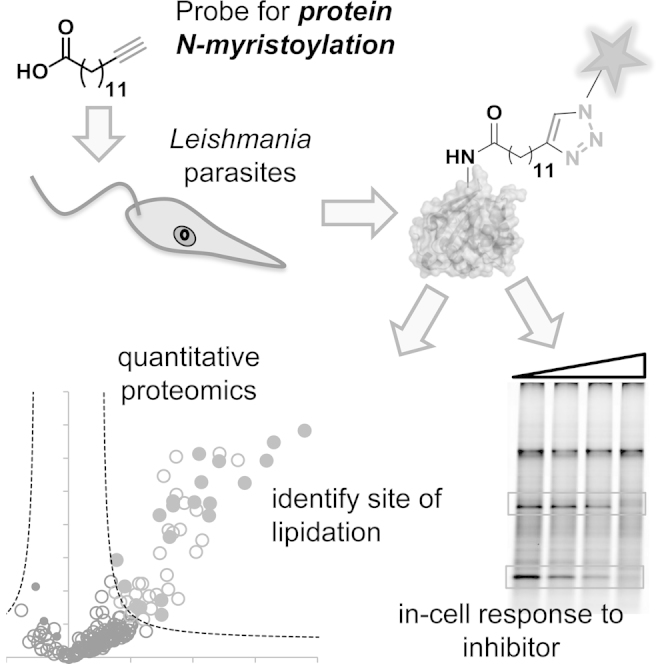

## Teaser

Wright et al. use metabolic tagging with an alkyne-myristate analog, click chemistry, and proteomics to identify lipidated proteins in two life stages of the parasite *Leishmania donovani*. Quantitative profiling of *N*-myristoyltransferase inhibition and identification of lipidation sites define the *N*-myristoylated proteome in this human pathogen.

## Introduction

Over 300 million people are at risk from the leishmaniases, a spectrum of neglected tropical parasitic diseases strongly associated with poverty, which cause around 30,000 deaths annually (Alvar et al., 2012). The diseases take two main forms: cutaneous leishmaniasis (CL), manifesting as skin lesions, and visceral leishmaniasis (VL), also known as kala-azar, a systemic infection causing swelling of organs, fever, and anemia, that is almost invariably fatal if left untreated. The causative agents of these debilitating diseases are species of the *Leishmania* protozoan parasite, a unicellular eukaryote. *Leishmania donovani* (Ld) is the principle causative agent of VL while *Leishmania major* (Lm) is the main species responsible for CL. The parasite is transmitted by sand fly vectors, which inoculate the promastigote forms into the human host. Here, they are taken up by phagocytic cells, mainly macrophages, and transform into the obligate intracellular amastigotes, which then replicate and can infect other phagocytes. Transmission occurs when a sand fly takes up infected cells in a blood meal. Inside the sand fly midgut, amastigotes transform into promastigotes. There are currently few drugs to treat the leishmaniases, and those that are available have problems of toxicity, teratogenicity, high cost, difficult administration, and/or parasite resistance. These drugs work via nonspecific antibiotic effects, such as disruption of cell membranes, or their mode of action is unknown (Tate et al., 2014). Novel drugs with a defined mechanism of action, high efficacy and low toxicity are urgently required to combat these devastating diseases.

**Figure 1:**
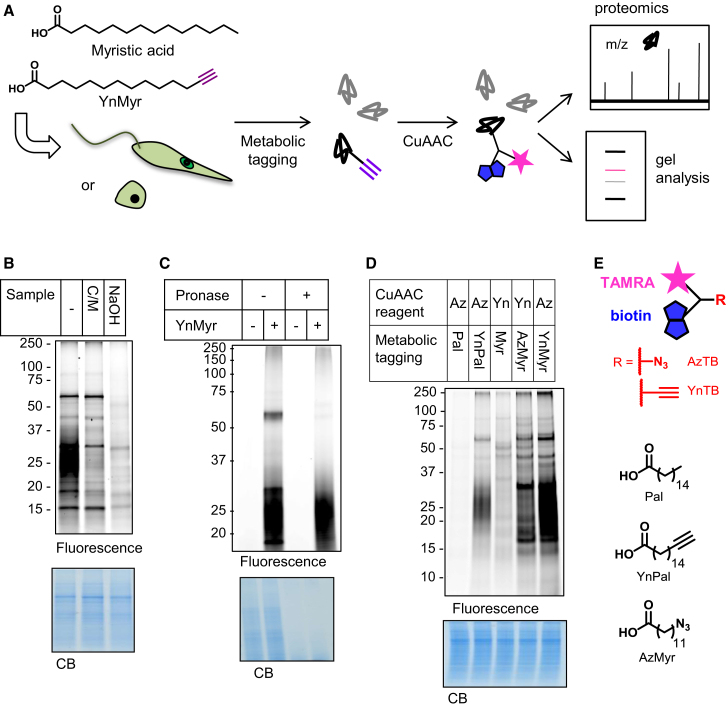
Tagging of Proteins by YnMyr in *Leishmania* (A) Overview of metabolic tagging of proteins with myristic acid analog YnMyr and analysis following CuAAC labeling. (B) YnMyr tagging in *L. donovani* promastigotes. Following CuAAC labeling with reagent AzTB, samples were precipitated with CHCl_3_/MeOH (CM) or with MeOH and then treated with NaOH as indicated. CB, Coomassie blue. (C) The effect of pronase digestion on tagging. Pronase digests protein resulting in a loss of the discrete fluorescent bands but leaving the diffuse labeling relatively unaffected. (D) YnMyr tagging compared with other analogs YnPal and AzMyr in promastigotes. Pal, palmitic acid; Myr, myristic acid. CuAAC capture reagent: Az, AzTB; Yn, YnTB. (E) Chemical tools. Additional labeling data are shown in Figure S1.

The enzyme myristoyl-CoA:protein *N*-myristoyltransferase (NMT) has recently been validated as a promising drug target in trypanosomiasis (Frearson et al., 2010) and malaria (Wright et al., 2014), diseases caused by the protozoan parasites *Trypanosoma brucei* and species of *Plasmodium*, respectively. NMT has also been proposed as a potential drug target for infections caused by parasitic nematodes (Galvin et al., 2014) and for the leishmaniases (Price et al., 2003). NMT catalyzes a protein modification, the attachment of the C14:0 lipid myristate (Myr, tetradecanoic acid, Figure 1A) to the N-terminal glycine residue of specific target proteins in the cell. This reaction usually occurs cotranslationally during ribosomal synthesis of the protein, affording a stable, amide-linked lipid that modulates protein function, typically promoting protein localization to cellular membranes (Wright et al., 2010). NMT has been characterized in Ld and Lm and appears to be essential for survival of promastigotes in both organisms (Brannigan et al., 2010; Price et al., 2003), although evidence for the importance of NMT in amastigotes has yet to be reported. Structure-based design and high-throughput screening has led to the discovery of several distinct NMT inhibitor series (Bell et al., 2012; Brannigan et al., 2014; Hutton et al., 2014; Olaleye et al., 2014). Around 60 *Leishmania* proteins have been predicted to be *N*-myristoylated using bioinformatic approaches based on learning sets from unrelated organisms (Mills et al., 2007), but experimental evidence has only been reported for six; these include ADP-ribosylation factor-like protein LdARL1 (Sahin et al., 2008), LdARL3A (Cuvillier et al., 2000), and several proteins that have been shown to carry a dual *N*-myristoylation and *S*-palmitoylation acylation motif. This latter modification involves the incorporation of hexadecanoic acid or other long chain fatty acids via a thioester linkage at a cysteine side chain, sometimes near the N terminus close to an *N*-myristoylation site. An example is dually acylated protein HASPB, a member of a family of hydrophilic acylated surface proteins important for parasite development in the insect vector (Sadlova et al., 2010). HASPB is unusual for an *N*-myristoylated protein in being localized to the outer leaflet of the plasma membrane in infective stages (Denny et al., 2000; Maclean et al., 2012). Similarly, SMP-1, one of several small myristoylated proteins, is flagellum-targeted by acylation (Tull et al., 2004). The SMPs are targeted to different subcellular compartments depending on N-terminal acylation and other sequence motifs (Tull et al., 2012).

Global profiling of protein lipidation in living cells by standard biochemical methods is challenging. We and others have made use of bioorthogonal labeling chemistries such as the copper-catalyzed cycloaddition of an alkyne and azide (CuAAC) to capture proteins metabolically tagged with alkyne or azide fatty acids (Grammel and Hang, 2013). In this approach, fatty acid analogs bearing the biologically inert azide or alkyne functional group are incubated with cells in culture and incorporated by cellular machinery into lipidated proteins. Following cell lysis, proteins can be functionalized via CuAAC with useful groups such as a fluorophore for visualization by in-gel fluorescence and a biotin affinity handle for enrichment, enabling subsequent mass spectrometry analysis and identification of modified proteins. Here, we use an alkyne-tagged myristate analog, YnMyr (tetradecynoic acid, Figure 1A), which has been previously used as a mimic for myristic acid in several systems, including mammalian cells (Thinon et al., 2014) and *Plasmodium falciparum* (Wright et al., 2014), to profile the lipidome of *L. donovani*. We further combine this chemical proteomic tool with small molecule NMT inhibitors to confirm target engagement inside live parasites, and to define the complement of NMT substrates.

## Results

### YnMyr Tags Proteins in *L. donovani*

We first sought to demonstrate that YnMyr can tag proteins in *Leishmania*, since this has not previously been reported. Metabolic tagging of Ld promastigotes with 50 μM YnMyr followed by CuAAC with biotin and fluorophore-functionalized reagent AzTB (Figures 1 and S1) gave several distinct fluorescent bands, and a diffuse smear between 18–30 kDa (Figure 1B). This diffuse labeling was effectively removed by CHCl_3_/MeOH precipitation or treatment with strong base and was resistant to pronase digestion (Figures 1B and 1C), suggesting YnMyr incorporation into a nonprotein component. *Leishmania* species synthesize complex glycolipid macromolecules, including glycosylphosphatidylinositol (GPI)-anchored proteins, glycoinositol phospholipids (GIPLs), and the complex glycoconjugate lipophosphoglycan (LPG) (Ferguson, 1997). Since these molecules contain acyl-fatty acids or ether-linked alkyl chains, YnMyr incorporation into such molecules is not unexpected; indeed, myristate is known to be incorporated into GIPLs and the GPI anchors of some proteins (Etges et al., 1986; Ralton et al., 2002). In addition to removal of the diffuse band, treatment with base in promastigotes resulted in loss of several other bands (Figure 1B). A well-studied protein in *Leishmania* is the surface protease GP63, which incorporates myristic acid into its GPI anchor (Etges et al., 1986). Consistent with this, GP63 could be detected after pull-down of YnMyr tagged promastigote lysate onto streptavidin beads (Figure S1). Hydroxylamine treatment of lysates gave a slight reduction in signal intensity of several weak bands, suggesting that YnMyr may also be incorporated into some *S*-acylation sites (Figure S1).

Labeling in promastigotes was time dependent, outcompeted by myristic acid and inhibited in the presence of the protein synthesis inhibitor cycloheximide (Figure S1), showing that YnMyr incorporation is dependent on de novo protein synthesis. Interestingly, although azido-myristate AzMyr produced a similar, but not identical, labeling pattern, this analog did not label the diffuse bands (Figure 1D), suggesting that the biosynthetic pathways incorporating the analog into these molecules may be less tolerant to modifications in the lipid structure. The background labeling generated by CuAAC with YnTB, the alkyne version of the AzTB capture reagent, was also higher (Figure 1D), as has been noted previously by others (Charron et al., 2009). These data suggest that YnMyr is the more suitable probe for labeling in *Leishmania*. Longer chain palmitic acid analog YnPal showed a distinct labeling pattern to the myristate analogs (Figure 1D), indicating that fatty acyl chain length is important for protein lipidation in these cells.

**Figure 2:**
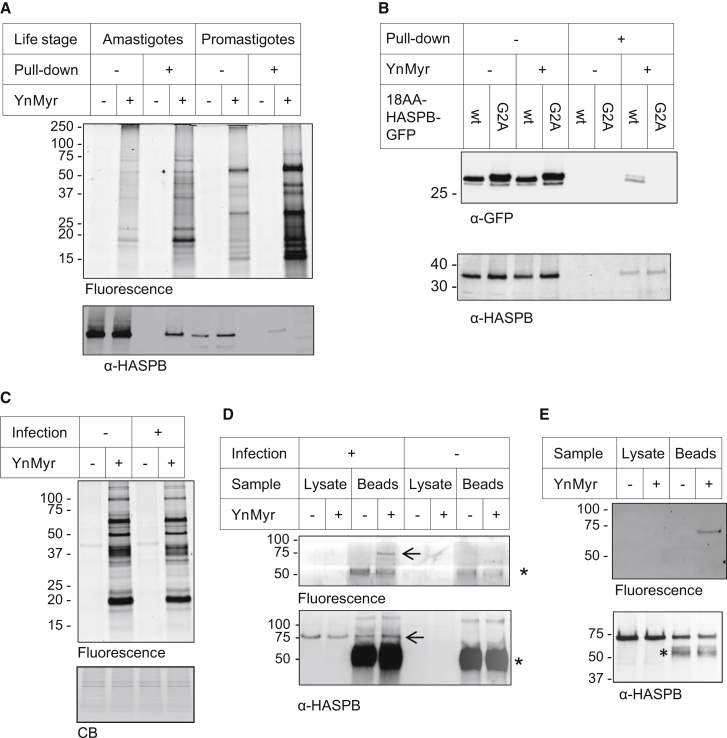
YnMyr Tags Known *N*-Myristoylated Protein HASPB (A) HASPB is labeled in promastigotes and amastigotes. Parasites were tagged with myristic acid or YnMyr, proteins labeled via CuAAC with AzTB, and a portion enriched by pull-down (PD) onto streptavidin beads. Four-fold more sample loaded after PD compared with before. Western blot (WB) revealed that HASPB is specifically enriched in the YnMyr samples. (B) HASPB is labeled on the N-terminal glycine. *L. major* parasites expressing the HASPB N terminus (18 residues; WT) fused to GFP or a G2A mutant were tagged with YnMyr and processed as above. Top: WB for GFP shows that only the WT protein and not the G2A mutant is tagged. Bottom: endogenous HASPB is tagged in both samples. (C) In-gel fluorescence analysis of YnMyr tagged proteins from macrophages infected with *L. donovani* or cultured alone. (D) HASPB is tagged in intracellular amastigotes. HASPB was immunoprecipitated (IP) from lysates derived from infected or uninfected macrophages tagged with YnMyr, and beads incubated with CuAAC reagents. In-gel fluorescence (top) shows a YnMyr- and infection-specific band at the expected migration for HASPB (arrow). WB (bottom) shows that HASPB (arrow) is present only in infected samples. ^∗^ indicates antibody IgG heavy chain. (E) HASPB IP and CuAAC using ex vivo amastigotes.

Metabolic tagging with YnMyr in the amastigote stage gave a distinct labeling pattern compared with promastigotes and no diffuse bands (Figure 2A). While GIPLs are expressed in both life cycle stages, LPG and GPI-anchored proteins are downregulated in the amastigote stage (McConville and Blackwell, 1991), suggesting that the diffuse labeling observed in promastigotes is related to specific glycolipid biosynthetic pathways. Known *N*-myristoylated protein HASPB (Alce et al., 1999) could be detected by western blot after pull-down of tagged proteins onto streptavidin beads in both life stages (Figure 2A), although the overall incorporation of YnMyr into HASPB was low, as may be expected given that the tagged analog competes for substrates with endogenous lipid. To confirm the nature of HASPB labeling, YnMyr tagging was performed in *L. major* strains expressing a fusion protein consisting of GFP fused to either the N-terminal 18 residues of HASPB (wild-type [WT] 18AA-HASPB-GFP), or to a mutant in which the N-terminal glycine was mutated to alanine (G2A) (Denny et al., 2000). The global labeling pattern was similar between these strains and comparable with that in *L. donovani* (Figure S1). Following enrichment of YnMyr tagged proteins, GFP was detected in the WT sample but not in the G2A mutant, consistent with canonical attachment of YnMyr to the N-terminal glycine by NMT; endogenous HASPB was enriched from both WT and G2A mutants (Figure 2B).

The freshly harvested ex vivo amastigotes used in the current study are a practical model system for studying the host infective stage of *Leishmania*, but methods to study this intracellular parasite in its native environment inside mammalian macrophages would be particularly useful for future host-parasite studies. To establish whether YnMyr can penetrate the amastigote inside the parasitophorous vacuole, macrophages infected with Ld amastigotes were incubated with YnMyr for 18 hr, lysed, and samples analyzed by gel; uninfected macrophages were used as a control. Strong labeling of macrophage proteins dominated in-gel fluorescence of both infected and uninfected samples, as might be expected from the low multiplicity of infection (∼10) of this intracellular model (Figure 2C). HASPB was immunoprecipitated (IP) from lysates and incubated with CuAAC reagents on-bead, followed by protein elution and analysis by western blot. HASPB was present only in infected samples, as expected, and a fluorescent band was also visible specifically in YnMyr tagged samples at the same molecular weight (Figure 2D). This IP-click approach, which also allows for detection of tagged HASPB in ex vivo amastigote samples (Figure 2E), circumvents the challenges posed by the relatively low abundance of parasite relative to host cell material. These data show that YnMyr also tags the *N*-myristoylated protein HASPB in the context of *Leishmania*-infected macrophages.

### Global Proteomic Identification of YnMyr Tagged Proteins in Promastigotes and Amastigotes

**Figure 3:**
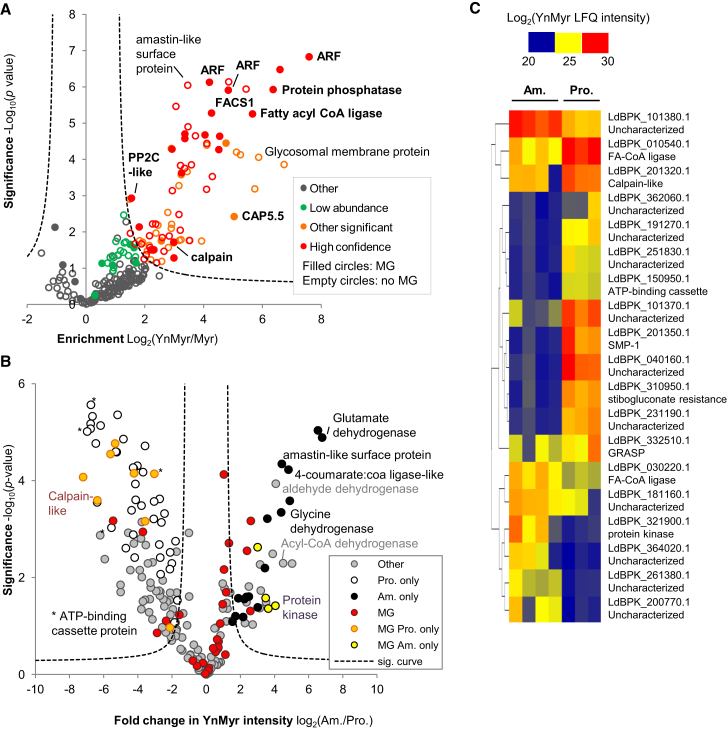
Proteomic Analysis of YnMyr Tagged Proteins in *Leishmania* (A) Volcano plot showing the results of a two-sample t test (FDR 0.05, S0 1) applied to amastigote LFQ intensities to assess enrichment in YnMyr samples over myristic acid controls (myr). MG, sequence contains an N-terminal glycine; high confidence, significant and absent from myr controls; low abundance, not significant in the t test but absent from controls; other significant, significant at FDR 0.05 but also detected in myr controls; other, other proteins identified. See also Figure S2. (B) Comparison of YnMyr intensities of amastigote and promastigote hits (t test significant and low abundance hits) by t test (FDR 0.05, S0 2) after imputation of missing values. Proteins are color coded to indicate those containing an N-terminal glycine (MG) and those found in only one of promastigote (Pro.) or amastigote (Am.) data sets. (C) Heatmap of YnMyr intensities across replicates for those MG proteins significantly differing between Am. and Pro.

Validating the tagging of specific proteins by western blot, as shown above for HASPB, is a low-throughput method and dependent on the availability of an antibody for the protein of interest. To carry out the global identification of YnMyr tagged proteins, Neutravidin-enriched proteins were digested on-bead with trypsin and peptides analyzed via liquid chromatography-tandem mass spectrometry (LC-MS/MS). For the MS experiments, a capture reagent (AzRB, Supplemental Experimental Procedures) containing a trypsin-cleavable motif between the azide and biotin groups was designed and used. We have previously used similar reagents to facilitate release of YnMyr-modified peptides from Neutravidin beads for identification (Thinon et al., 2014). A label-free quantification (LFQ) approach (Cox et al., 2014) was used to assess enrichment over myristic acid controls. The nature of an enrichment experiment means that hits are often not detected in controls, and analysis of the data was approached in two ways. First, values for missing LFQ intensities were imputed from a normal distribution to simulate values at the lower limit of detection and a permutation-corrected (false discovery rate, FDR) t test was applied to define significance (Figure 3A; Figure S2). Second, the absence of a protein from controls was considered. Proteins were defined as high confidence hits if they reached significance in the t test and were absent from controls, while a protein was defined as a low abundance hit if it was absent from controls but did not reach statistical significance due to low intensity in the YnMyr samples. This latter group is more likely to contain false positives in addition to true hits.

In amastigotes, 81 proteins were significantly enriched (FDR 0.05) and, of these, 49 proteins were assigned as high confidence using the definition above. A further 20 proteins were classified as low abundance hits (Table S1). In promastigotes, 134 proteins were significantly enriched (FDR 0.01) and, of these, 113 were high confidence; 28 proteins were assigned as low abundance (Table S2). Notably, 59% of high confidence hits in amastigotes did not contain an N-terminal glycine (MG), proposed to be a prerequisite of NMT-dependent myristoylation. In promastigotes, this figure rose to 78%, suggesting that YnMyr may not exclusively tag *N*-myristoylated proteins in *Leishmania*. However, a number of proteins were indeed detected for which there is prior experimental evidence for *N*-myristoylation in *Leishmania*, including ARL1 (Sahin et al., 2008), SMP-1 (Tull et al., 2004), CAP5.5 (Hertz-Fowler et al., 2001), and PPEF (Mills et al., 2007). Other identified MG proteins include other ARF/ARL proteins, Golgi reassembly stacking protein GRASP, several other calpain-like cysteine peptidases, and protein phosphatases/kinases.

Probable integral membrane proteins were prominent in the non-MG data sets; of the 107 significant non-MG hits in promastigotes, 57 had predicted transmembrane (TM) helices, and of the 58 non-MG hits in amastigotes, 28 had this feature (Tables S1 and S2). Conversely, only 3 MG proteins across both data sets had a predicted TM domain. *S*-Palmitoylation of TM proteins is well known to occur in other eukaryotes (Blaskovic et al., 2013) and it is possible that YnMyr is incorporated into these proteins by palmitoyltransferases. Indeed, bioinformatic analysis of the protein sequence suggested that many hits that lacked the MG motif could be *S*-palmitoylated (Figure S2; Table S3). Putative GPI-anchored proteins were enriched by YnMyr tagging, including five uncharacterized proteins (LdBPK_331200.1, LdBPK_352190.1, LdBPK_090690.1, LdBPK_364340.1, LdBPK_350860.1), three surface antigens (LdBPK_050900.1, LdBPK_051210.1, LdBPK_040180.1), and a putative vacuolar-type proton translocating pyrophosphatase (Table S3).

Comparison of hits (defined as proteins significantly enriched over myristic acid controls or low abundance hits) in the two *L. donovani* life stages showed high overlap of 67% (Table S3). A t test was used to compare the YnMyr LFQ intensities of hits (Figure 3B) revealing that select MG proteins are differentially expressed. Proteomic studies have revealed changes related to the transition from promastigote to amastigote stage, in particular an increase in proteins associated with gluconeogenesis and mitochondrial metabolism in amastigotes (Biyani and Madhubala, 2012; Rosenzweig et al., 2008). The most strongly differentially regulated putative *N*-myristoylated proteins identified here are mostly proteins of unknown function, apart from SMP-1 and sodium stibogluconate resistance protein, enriched in promastigotes, and a putative protein kinase found only in amastigotes (Figure 3C). Overall, likely *N*-myristoylated proteins with important functions including transport, protein (de)phosphorylation, proteasomal degradation, and Golgi function are conserved in both *Leishmania* life stages.

### Chemical Knockdown of *N*-Myristoylation Defines NMT Substrates

**Figure 4:**
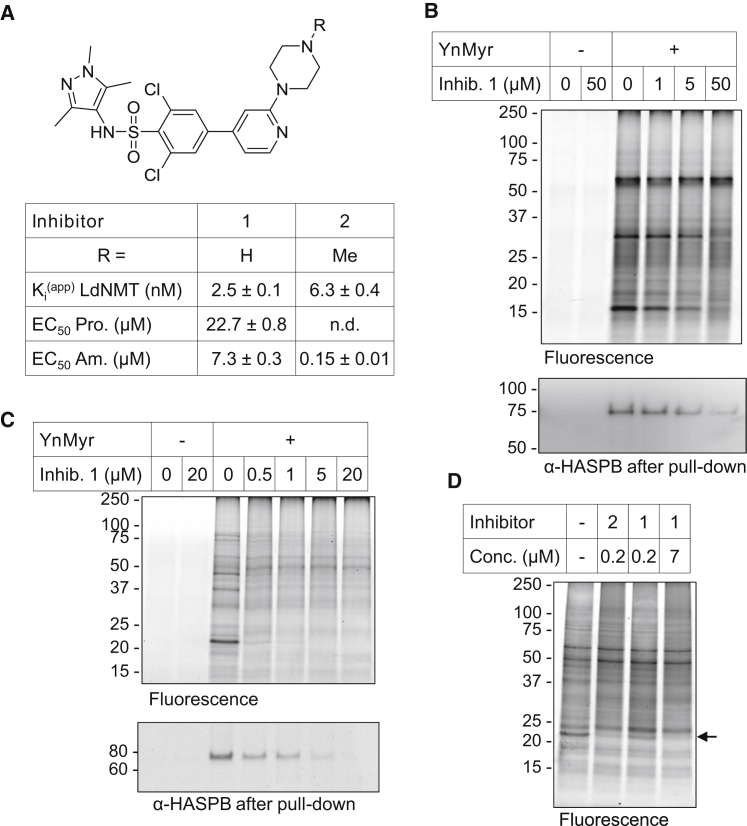
Effect of NMT Inhibition on YnMyr Tagging (A) Structures of inhibitors **1** and **2** and summary of their activity against recombinant LdNMT (apparent inhibition constant, *K*_i_^(app)^) and against promastigotes (Pro.) or ex vivo amastigotes (Am.) (half maximal effective concentration, EC_50_). (B) Gel-based analysis of YnMyr tagging in promastigotes treated with inhibitor **1**. Top: in-gel fluorescence showing the effect of increasing **1** concentrations. Bottom: western blot (WB) of HASPB following enrichment of YnMyr tagged proteins by pull-down (PD) onto streptavidin beads. (C) Gel-based analysis of YnMyr tagging in amastigotes treated with inhibitor **1**. Top: in-gel fluorescence showing the effect of increasing **1** concentrations. Bottom: WB of HASPB following enrichment of YnMyr tagged proteins by PD onto streptavidin beads. (D) In-gel fluorescence analysis of YnMyr tagging in amastigotes treated with inhibitor **1** or **2**. Arrow indicates most prominent loss of YnMyr tagging upon inhibition. Full blots and Coomassie gels are shown in Figure S3.

Although the global proteomic approach identified a number of likely *N*-myristoylated proteins, the complexity of YnMyr labeling in *Leishmania* complicates the firm assignment of specific proteins as NMT substrates. We therefore sought to use chemical knockdown of NMT in combination with YnMyr tagging to distinguish those proteins that are *N*-myristoylated from other lipidated proteins, an approach recently used to define substrates of the human NMTs in cancer cells (Thinon et al., 2014). Compound **1** (Figure 4A) was first reported as a *T. brucei* NMT inhibitor with in vivo efficacy in a rodent model of trypanosomiasis (Brand et al., 2012; Frearson et al., 2010), and we have subsequently shown that this inhibitor also acts on-target to inhibit NMT in malaria parasites (Wright et al., 2014) and human cancer cell lines (Thinon et al., 2014). Compound **1** has a *K*_i_^(app)^ (apparent inhibition constant) of 2.5 nM against LdNMT and EC_50_s (half maximal effective concentrations) of 7 μM and 23 μM against Ld amastigotes and promastigotes, respectively (Paape et al., 2014). Parasites were preincubated with **1** for 1 hr at varying concentrations and then YnMyr was added for 12 hr. Following CuAAC, a drop in intensity in specific bands could be observed in both life stages in response to inhibitor (Figures 4B and 4C). Many bands were completely unaffected, possibly because these proteins are not tagged via NMT activity, consistent with the proteomic analyses above suggesting that YnMyr does not label only *N*-myristoylated proteins. Blotting for HASPB after pull-down revealed a dose-dependent decrease in the YnMyr tagging of this protein (Figures 4B and 4C). These data suggest that compound **1** inhibits NMT inside the cell.

While **1** translates very poorly from enzyme to cellular potency, analog **2** shows similar enzyme inhibitory activity but is nearly 50-fold more potent in the amastigote growth inhibition assay (Figure 4A). We hypothesized that this contrast could be explained by the distinct physicochemical properties of the compounds, which can affect uptake, efflux, and metabolism, or by off-targets of **2**. Incubation with **2** at a concentration around its EC_50_ (0.2 μM) gave a similar decrease in YnMyr labeling to that observed at the EC_50_ of **1** (7 μM) (Figure 4D), suggesting that despite very different translation from recombinant enzyme to cell potency, both inhibitors engage NMT inside the cell. As expected, a lower concentration of **1** (0.2 μM) resulted in only a partial loss of specific labeling.

**Figure 5:**
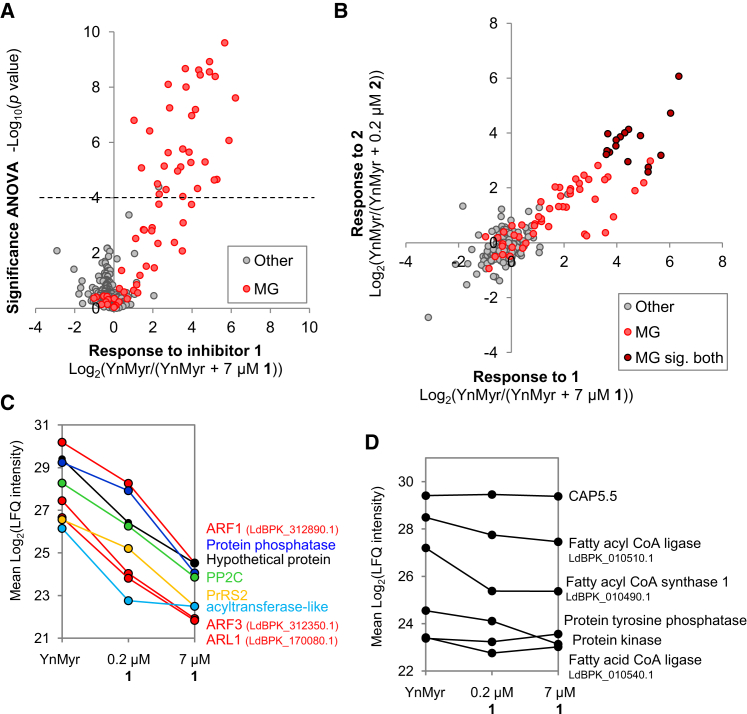
Proteomics Analysis of YnMyr Tagged Proteins in Amastigotes Treated with NMT Inhibitors (A) ANOVA test (Benjamini Hochberg correction, FDR 0.001) applied to LFQ intensities to compare inhibition conditions. The fold change in response to 7 μM **1** is plotted against −log_10_(*p* value). Proteins above the dashed line reached significance in this test. MG, sequence contains an N-terminal glycine. Volcano plots of two-sample t tests are shown in Figure S5. (B) Comparison of LFQ ratios for 0.2 μM **2** and 7 μM **1** data sets after removal of nonspecific binders. The proteins assigned as significantly affected by both inhibitors are indicated. See Figure S5 for direct comparison of these ratios by t test. (C) Response of selected MG proteins to 0.2 and 7 μM **1**. Mean LFQ intensity before imputation of missing values is plotted. (D) MG proteins where YnMyr tagging does not respond to NMT inhibition.

Inhibited samples were subject to pull-down, digest, and LC-MS/MS. Biological and technical reproducibility of LFQ intensities across replicates was high (*R*^2^ 0.76–0.96 after imputation of missing values; Figure S4) and the 580 proteins present in biological duplicates in the YnMyr samples were selected for further analysis (Table S4). After filtering out nonspecific binders, missing values were imputed. ANOVA (Benjamini Hochberg correction, FDR 0.001) was used to compare across all four conditions (YnMyr, 0.2 μM **2**, 0.2 μM **1**, and 7 μM **1**) and permutation-corrected t tests were used to compare each inhibition condition with YnMyr controls. In all analyses there was a significant reduction in intensity in a subset of MG proteins (Figure 5A; Table S4; Figure S5). Relative to the YnMyr control, 30 proteins in the 7 μM **1** data set were significantly reduced and all contained an N-terminal glycine.

Several bioinformatic tools (Bologna et al., 2004; Maurer-Stroh et al., 2002) have been developed to predict *N*-myristoylation. However, these tools are based on learning sets derived from other eukaryotes and, of the MG proteins identified here as enriched through YnMyr tagging, the predictors disagreed on whether a protein was a substrate in 30% of cases (Table S5). In addition, several high confidence hits were assigned as nonmyristoylated by both tools, underlining the value of experimental approaches to test bioinformatic predictions.

For those hits detected and assigned as significant at both concentrations of inhibitor **1**, there was a clear dose-dependent decrease in intensity in response to **1** (Figure 5C). Several MG proteins showed no response and a subset of hits were significantly decreased only at the higher concentration (7 μM) of **1** (Table S4). This latter group may contain proteins that are less sensitive to NMT inhibition, possibly higher affinity substrates (Thinon et al., 2014). Other proteins (Figure 5D) also did not show strong responses to **1** and **2**. All 16 proteins significantly affected by inhibitor **2** were also hits in the **1** data set (Figure 5B; Table S4). However, comparing the LFQ intensities of hits qualitatively and via a t test indicated that in fact 0.2 μM **2** did not inhibit *N*-myristoylation as effectively as 7 μM **1** (Figure S5). A potential explanation for this result is that **2** does inhibit NMT in amastigotes but may have additional off-target effects that slightly increase its potency in cells.

**Figure 6:**
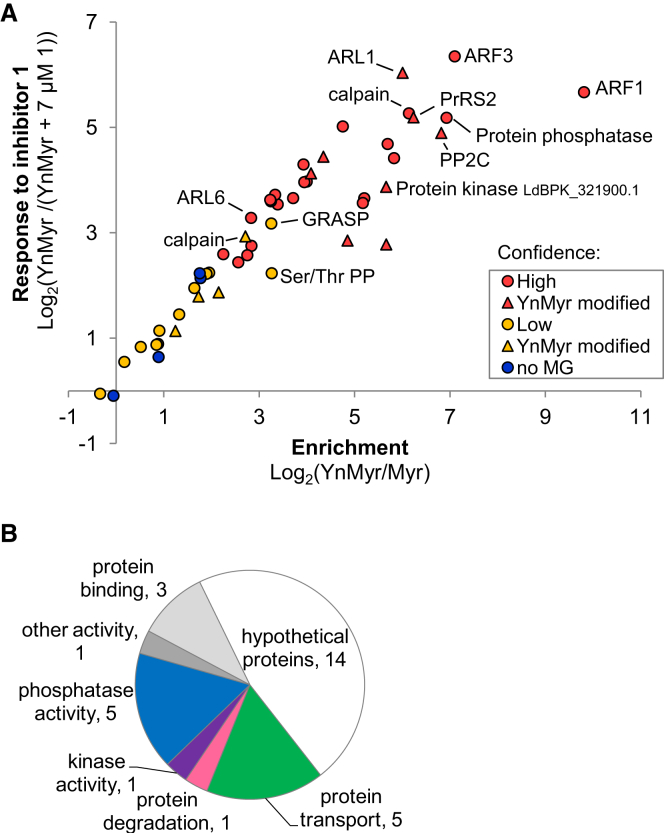
Analysis of Proteins whose Acylation Responds to NMT Inhibitors (A) Enrichment over myristic acid (Myr) control compared with response to inhibitor (7 μM **1**) for hits. High confidence, significantly affected by inhibition (t test FDR 0.001); low confidence, not detected in inhibitor-treated samples but not significant in the t test due to low intensity in the YnMyr controls. Proteins for which YnMyr modification on the N-terminal glycine was detected are indicated (see also Table S6). (B) GO annotation (TriTrypDB) of 30 high confidence NMT substrates.

Since 7 μM **1** resulted in the strongest response, this data set was used in combination with visual examination of heatmaps to define high and low confidence hits (Table 1; Table S4; Figure 6A). All 30 proteins significant in the t test were defined as high confidence NMT substrates since they showed either a dose-response relationship or were absent from inhibited samples. Low confidence hits were defined as those not reaching significance in the t test but also not detected in the inhibited sample, and include proteins expected to be *N*-myristoylated such as GRASP, a calpain, and a serine/threonine phosphatase, but also four proteins that have no N-terminal MG motif and so are unlikely to be myristoylated. Of the 30 high confidence proteins, 18 had been identified as significant or low abundance hits in the global amastigote analysis, but a further 11 had been identified in only one or two replicates (Table S1), highlighting the value of employing additional tools to explore such complex samples.

To provide further insight into the YnMyr modification of proteins, we used the trypsin-cleavable site in AzRB to enable direct identification of YnMyr-modified peptides. De novo-aided sequencing (Zhang et al., 2012) was used to search for peptides containing the YnMyr-AzRB-derived adduct at their N terminus. A total of 20 proteins were detected with this modification on a glycine residue corresponding to the N terminus of the protein (Table S6), and no peptides were identified in searches of myristic acid controls. Of these 20 proteins, 8 had been identified as high confidence NMT substrates via quantification of their response to NMT inhibition and include known *N*-myristoylated protein LdARL1, a proteasome subunit, a PP2C-like protein, a predicted protein kinase and four uncharacterized proteins (Tables 1 and S6; Figure 6A). An additional four lower confidence substrates were also modified. Interestingly, modification on the N-terminal glycine of three proteins (two fatty acyl-CoA ligases and uncharacterized LdBPK_300680.1) that did not respond robustly to NMT inhibition was detected; these proteins may be NMT substrates that are additionally modified elsewhere, for example, at their enzyme active site or at alternative sites, or may be modified on their N terminus by a mechanism distinct from NMT. Several other modified proteins were identified that had not been included in the global analyses due to weak detection. Gene ontology analysis of the 30 high confidence NMT substrates revealed that they have diverse functions and include phosphatases and kinases, proteins involved in transport and degradation, as well as many (∼50%) proteins of unknown function (Figure 6B).

## Discussion

Here we combine chemical proteomic tools with inhibitors and quantitative mass spectrometry to define the complement of *N*-myristoylated proteins in *L. donovani* parasites, and explore NMT as a potential drug target in this important human pathogen. Proteomic and gel-based analyses reveal that YnMyr is incorporated into diverse sets of proteins, including *N*-myristoylated and GPI-anchored proteins, as well as into what are most likely complex glycolipids. YnMyr incorporation into proteins other than NMT substrates is not unprecedented; for example, we observed significant YnMyr tagging of GPI-anchored proteins in malaria parasites (Wright et al., 2014). However, the proteomic analyses carried out here suggest that while YnMyr does tag GPI-anchored proteins in Ld, these are by no means the most abundant hits. A possibility is incorporation into *S*-acylation sites, since in mammalian cells YnMyr and similar probes label both *N*-myristoylated and *S*-palmitoylated proteins (Charron et al., 2009; Thinon et al., 2014) and palmitoyltransferases have varying acyl-CoA specificities (Jennings and Linder, 2012). Alternatively, YnMyr may be metabolized to longer or shorter chain analogs retaining the alkyne tag that are subsequently incorporated into proteins, or it may be that alternative lipid modifications, such as the poorly characterized lysine *N*-acylation (Lin et al., 2012), are prevalent in *Leishmania*. Although enzymes with lysine deacylase activity have been described (Jiang et al., 2013), those that carry out lysine acylation have not been identified and it has been suggested that such modifications may in fact be nonenzymatic (Wagner and Hirschey, 2014). *Leishmania* may metabolize YnMyr to a reactive intermediate that nonenzymatically modifies proteins.

In addition to identification of lipidated proteins, we provide evidence that compounds **1** and **2** inhibit NMT in live parasites. While the potent in vitro activities of these compounds translate to cellular activity rather poorly, and to very different extents, we show here that this activity is accompanied by a drop in *N*-myristoylation levels of specific proteins and thus provide the first direct evidence for the druggability of NMT in *Leishmania*. Problems with translation from enzyme to cellular assays have been reported previously in *Leishmania*, particularly in amastigotes (Walker et al., 2011), but we anticipate that the methodology developed here will be useful for screening NMT inhibitors for target engagement inside the cell. Further experiments are now required to confirm that the antiparasitic effects of inhibitors **1** and **2** arise solely through inhibition of NMT, and to identify which NMT substrates mediate these effects.

The depth of coverage of the YnMyr tagged proteome identified could be improved; for example, HASPB, despite being detectably tagged in gel-based analyses, was not identified in MS studies; this may be due in large part to the sequence of this protein, which contains multiple repeats and is highly hydrophilic. Several possible NMT substrates were detected but did not meet the stringent filtering criteria applied and it is thus likely that more NMT substrates remain to be validated in the future.

The set of proteins assigned as NMT substrates here is generally consistent with previous literature on *N*-myristoylated proteins in *Leishmania* and other eukaryotes. Surprisingly, the lipidation levels of CAP5.5 did not respond to inhibition of NMT (Figure 5D; Table S4). CAP5.5 contains a strongly predicted myristoylation motif (Table S5) and its ortholog has been shown experimentally to be *N*-myristoylated and *S*-palmitoylated in *T. brucei*, the latter presumably on a nearby cysteine residue (Hertz-Fowler et al., 2001). Similarly, although the phosphatase PPEF did respond to NMT inhibition, basal YnMyr tagging was still detectable (Table 1). There is evidence that PPEF is dually acylated in *L. major*, with palmitoylation being dependent on myristoylation, suggesting that in the absence of myristoylation no labeling should occur (Mills et al., 2007). It is possible that the relatively high concentration of YnMyr used here could force incorporation into *S*-acylation or other sites, or that experimental differences between studies may account for these different results; the dynamic nature of *S*-acylation may render labeling highly dependent on incubation time and other culture conditions. Alternatively, we cannot exclude the existence of another enzyme with acyltransferase activity that carries out N-terminal *N*-lipidation in *Leishmania*, although no experimental or bioinformatic evidence has yet been reported to suggest this, and there is no evidence for compensation on deletion of NMT in promastigotes (Brannigan et al., 2010).

Just over half of the 30 high confidence NMT substrates identified here are uncharacterized proteins, highlighting the huge amount of NMT-related biology yet to be explored in *Leishmania*. However, some predictions for the outcome of NMT inhibition can be drawn from these data. ARF/ARL proteins, of which five were identified as strongly affected by NMT inhibition, are important mediators of intracellular trafficking in all eukaryotes and their specific localization is often dependent on *N*-myristoylation. LdARL1 is localized to the *trans*-Golgi network and involved in control of endocytosis (Sahin et al., 2008); its *T. brucei* homolog is also a Golgi protein essential in bloodstream parasites, where its depletion causes defects in exocytosis and Golgi structure (Price et al., 2005). TbARL6 is involved in flagellum extension, interaction with microtubules, and the BBSome (Price et al., 2012), which is important for specific trafficking events in all eukaryotes and is essential for virulence in *Leishmania* (Price et al., 2013). Therefore basic cellular functions such as Golgi structure, endo- and exocytosis are very likely to be affected by inhibition of NMT, perhaps affecting parasite virulence. The identification of three protein phosphatases, one kinase and phosphatidylinositol phosphate kinase, suggests that signaling processes dependent on specific (de)phosphorylation events will also be disrupted. Finally, the proteasome has already been shown to be a potential drug target in *Plasmodium* (Li et al., 2014) and *N*-myristoylation of this subunit linked to function in yeast, where removal of the myristoylation site causes proteasome mislocalization, accumulation of misfolded protein, and growth defects (Kimura et al., 2012). In summary, these data predict a strongly pleiotropic effect of NMT inhibition on parasite cellular function and provide evidence for NMT as a valid drug target in *Leishmania*.

## Significance

**The leishmaniases are a spectrum of neglected tropical diseases for which few drugs are available and novel approaches are urgently required. A deeper understanding of the underlying biology, and chemical validation, of potential drug targets may improve the chances of successfully developing new treatments. NMT catalyzes a specific and essential lipidation of proteins in eukaryotes and the present study explores protein lipidation in *Leishmania donovani*, the causative agent of the most severe form of the leishmaniases. We take advantage of the global picture afforded by a chemical proteomic approach using a bioorthogonally tagged lipid analog to show that protein lipidation in the parasite is diverse and complex. To overcome this complexity and identify NMT substrates, we quantify protein lipidation levels in the presence of NMT inhibitors and support this with direct lipidation site identification. These results predict pleiotropic effects of NMT inhibition in parasites and provide a method to assay for target engagement in the cell. This work also constitutes the first global experimental analysis of protein lipidation in *Leishmania* and contributes a large proteomic data set for this neglected human pathogen. These data may provide further insights into other drug targets, for example, the complex glycolipid pathways or individual functionally important NMT substrates.**

## Experimental Procedures

### Supplemental Experimental Procedures

The following procedures were carried out essentially as described previously and details are given in the Supplemental Experimental Procedures: ex vivo amastigote inhibition, macrophage cytotoxicity, and enzyme inhibition assays (Hutton et al., 2014); sample preparation for proteomics analysis and LC-MS/MS (Thinon et al., 2014).

### Chemical Tools

The following chemical tools were synthesized as described previously: YnMyr, YnPal, and AzTB (Heal et al., 2011); AzMyr and YnTB (Heal et al., 2012); inhibitors **1** and **2** (Thinon et al., 2014). Synthesis of AzRB is given in the Supplemental Experimental Procedures. Myristic and palmitic acids, cycloheximide (CHX), and all other chemicals were purchased.

### Ethics Statement

Animal experiments were approved by the University of York Animal Procedures and Ethics Committee and performed under UK Home Office license (Immunity and Immunopathology of Leishmaniasis Ref. no. PPL 60/3708).

### Parasite Culture

The Ethiopian LV9 strain of *Leishmania donovani* (MHOM/ET/67/L28) was maintained by serial passage in Rag-2^−/−^ mice. Amastigotes were extracted from spleen as described previously (Smelt et al., 1997) and cultured in RPMI/10% fetal calf serum (FCS). Promastigotes were obtained by transforming 1 × 10^7^ freshly isolated amastigotes in promastigote medium (St-Denis et al., 1999) at 26°C (∼2 days for transformation). Promastigotes were passaged twice a week for up to 10 passages. Further details are given in the Supplemental Experimental Procedures.

### Metabolic Tagging Experiments

Promastigotes were cultured in RPMI/10% FCS containing the appropriate probe (50 μM YnMyr, myristic acid, or as indicated) at a parasite density of 7.5 × 10^7^ parasites/ml. Cultures were incubated at 26°C for 12 hr (or as indicated). Cells were collected (800 × *g*, 15 min, 4°C), washed twice with cold PBS, then lysed at 1 × 10^9^ parasites/ml by sonication on ice (4 × 10 s burst, amplitude 45 with 1 min interval) in 1% NP40, 1% sodium deoxycholate, 0.5% SDS, 50 mM Tris (pH 7.4), 150 mM NaCl, EDTA-free protease inhibitor. Insoluble material was separated by centrifugation (16,100 × *g*, 30 min, 4°C). Tagging of ex vivo amastigotes was carried out as above but parasites were cultured at 37°C. For inhibition studies, the appropriate inhibitor was preincubated with parasite culture for 1 hr at 37°C, parasites pelleted and resuspended in fresh medium containing inhibitor plus probe (YnMyr etc). Further details are given in the Supplemental Experimental Procedures.

### Intracellular Amastigote Tagging

BALB/c mice were obtained from Charles River. Macrophages (BMDM) were differentiated from bone marrow of BALB/c mice as described previously (Weinheber et al., 1998) and plated out at 1 × 10^6^ cells per well in six-well plates. BMDMs were adhered overnight and then infected at a multiplicity of infection of 10 with freshly isolated *L. donovani* amastigotes (see above). After 24 hr no free parasites were observed. Tagging was performed for 18 hr with YnMyr or myristic acid at a final concentration of 100 μM. Cells were washed three times with PBS at room temperature (RT) before 300 μl of lysis buffer was added and cells scraped off. The lysate was sonicated (3× 10 s, amplitude 45 with 1 min intervals), then centrifuged for 30 min at 16,100 × *g* at 4°C. The supernatant was transferred into a fresh tube and flash frozen in liquid nitrogen. BMDMs were maintained in DMEM supplemented with 4 mM L-glutamine and 4% L929-cell conditioned medium. All experiments were performed at 37°C and 5% CO_2_.

### CuAAC Labeling and Pull-down

Proteins were precipitated with chloroform/methanol (MeOH:CHCl_3_:ddH_2_O 4:1:3), or acetone (4 volumes at −20°C for 1 hr) and then resuspended at 1 mg/mL in 1% SDS in PBS. This precipitation step was found to increase labeling intensity after CuAAC. Premixed click reagents (100 μM AzTB, 1 mM CuSO_4_, 1 mM TCEP, 100 μM TBTA, final concentrations) were added (Heal et al., 2012) and samples vortexed for 1 hr at RT, then quenched by the addition of 10 mM EDTA. Proteins were precipitated again with MeOH/CHCl_3_ or with 10 volumes of MeOH (overnight at −80°C), washed with ice-cold MeOH, air-dried, and resuspended in 2% SDS, 10 mM EDTA in PBS. For direct gel analysis, 4× sample loading buffer (NuPAGE LDS sample buffer) with 2-mercaptoethanol (4% final) was added. For hydroxylamine (NH_2_OH) and NaOH treatment, samples were treated with 1 M NH_2_OH (pH 7) or 0.2 M NaOH at RT for 1 hr. Samples were quenched by addition of 4× sample loading buffer. Note that addition of EDTA to the samples before NH_2_OH treatment is important to avoid sample degradation. Proteins were heated for 3 min at 95°C prior to SDS-PAGE. Pull-down was carried out with Dynabeads MyOne Streptavidin C1 as described previously (Wright et al., 2014) (see Supplemental Experimental Procedures).

### Gel and Western Blot Analysis

Samples were separated by SDS-PAGE and scanned with Cy3 filters to detect the TAMRA fluorophore using an Ettan DIGE scanner (GE Healthcare).

For western blot, proteins were transferred from gels to polyvinylidene fluoride membrane (Immobilon-P^SQ^, Millipore) using a semidry system (Invitrogen). Following blocking (5% milk in Tris-buffered saline, 1% Tween), membranes were incubated with primary antibody for 1 hr at RT or overnight at 4°C in blocking solution, then incubated with secondary antibody (goat anti-rabbit IgG-HRP, Invitrogen, 1:10,000) for 1 hr in blocking solution. Detection was carried out using Luminata Crescendo Western HRP substrate (Millipore) according to the manufacturer’s instructions and on a Fujifilm LAS 3000 imager. Primary antibodies, LdHASPB (rabbit; ab 2-AE) (Alce et al., 1999), LmHASPB (rabbit, 336) (Alce et al., 1999), GP63 (rabbit, polyclonal; provided by R. McMaster, University of British Columbia) (Frommel et al., 1990), GFP (mouse, Santa Cruz), were used at 1:1,000–2,000.

### Proteomic Data Searching and Analysis

#### Data Searching and Analysis

The data were processed with MaxQuant version 1.3.0.5 and the peptides were identified from the MS/MS spectra searched against The TriTrypDB-6.0 *L. donovani* LdBPK282A1 database using the Andromeda search engine. The protein HASPB was not present in this database and so the Uniprot sequence (O77300_LEIDO) was appended to the FASTA file. Cysteine carbamidomethylation was used as a fixed modification and methionine oxidation and N-terminal acetylation as variable modifications. The false discovery rate was set to 0.01 for peptides, proteins, and sites. Other parameters were used as preset in the software. “Unique and razor peptides” mode was selected to allow for protein grouping; this calculates ratios from unique and razor peptides (razor peptides are uniquely assigned to protein groups and not to individual proteins). Data were elaborated using Perseus versions 1.4.0.20 and 1.5.0.31. LFQ experiments in MaxQuant were performed using the built-in label-free quantification algorithm (Cox et al., 2014).

#### Modified Peptide Identification

MS data were processed with PEAKS7 suite (Zhang et al., 2012) The data were searched against a reference *L. donovani* database from Uniprot (27/11/2014). Trypsin (specific, up to three missed cleavages allowed) was selected for database searches and no enzyme was chosen for de novo searches. The maximal mass error was set to 5 ppm for precursor ions and 0.01 Da for product ions. Carbamidomethylation was selected as a fixed modification and methionine oxidation as well as the lipid-derived adduct (+534.3278 Da) to any amino acid at peptide N terminus were set as variable modifications. The maximal number of modifications per peptide was set as five. The false discovery rate was set to 0.01 for peptides and b1 ions were required for N-terminally modified peptides. Within PEAKS, a module called SPIDER searches for point mutations to further enhance the discovery (Ma and Johnson, 2011).

#### Data Processing: YnMyr Tagged Proteins in Amastigotes and Promastigotes

For amastigotes, four replicates were performed independently, starting from the same metabolically labeled lysates (Myr and YnMyr samples). Data were filtered to require three valid values across the four replicates, label-free intensities were logarithmized (base 2), and empty values were imputed with random numbers from a normal distribution, whose mean and SD were chosen to simulate low abundance values close to noise level (impute criteria: width 0.1, downshift 1.8). A modified t test with permutation-based FDR statistics was applied (250 permutations; FDR 0.05; S0 1).

For promastigotes, three replicates were performed independently, starting from the same metabolically labeled lysates (Myr and YnMyr samples). Data were analyzed as described above with three valid values per group required, imputation (width 0.1, downshift 1.8), and t test (250 permutations; FDR 0.01; S0 1).

For the comparison of amastigotes and promastigotes, data were filtered for at least three valid values in at least one group (groups: Am_Myr, Am_YnMyr, Pro_Myr, Pro_YnMyr). YnMyr intensities of hits (proteins that were either t test significant or classed as low abundance hits in the independent analyses) were compared by t test (250 permutations; FDR 0.05; S0 2) after imputation of missing values (width 0.1, downshift 2.5). Hierarchical clustering was carried out in Perseus.

#### Data Processing: YnMyr Tagging in the Presence of NMT Inhibitors (Amastigotes)

Independent biological experiments were carried out where amastigotes were incubated with YnMyr, YnMyr + 0.2 μM **2**, YnMyr + 0.2 μM **1**, or YnMyr + 7 μM **1**. A Myr control was included in one of these experiments. For proteomics analysis, all samples were prepared and processed in duplicate (technical replicates: A and B; C and D). The “Match between runs” option (time window 2 min) in MaxQuant was enabled during the searches. Data were grouped, filtered to retain only those proteins present in both biological experiments, and nonspecific binders (enrichment over control <2) removed. Missing values were imputed from a normal distribution (width 0.1, downshift 1.8) and modified two-sample t tests were applied (250 permutations; FDR 0.001; S0 1). ANOVA (Benjamini Hochberg correction, FDR 0.001) was also applied to LFQ intensities to compare across all data sets.

## Author Contributions

M.H.W. performed sample handling and analysis downstream of parasite culture. D.P. performed parasite culture, metabolic tagging, and live parasite assays. E.M.S. synthesized reagent AzRB. R.A.S. carried out searches to identify modified peptides. D.F.S. and E.W.T. conceived and directed the study. M.H.W. wrote the manuscript with input from all the other authors.

## Figures and Tables

**Table 1:**
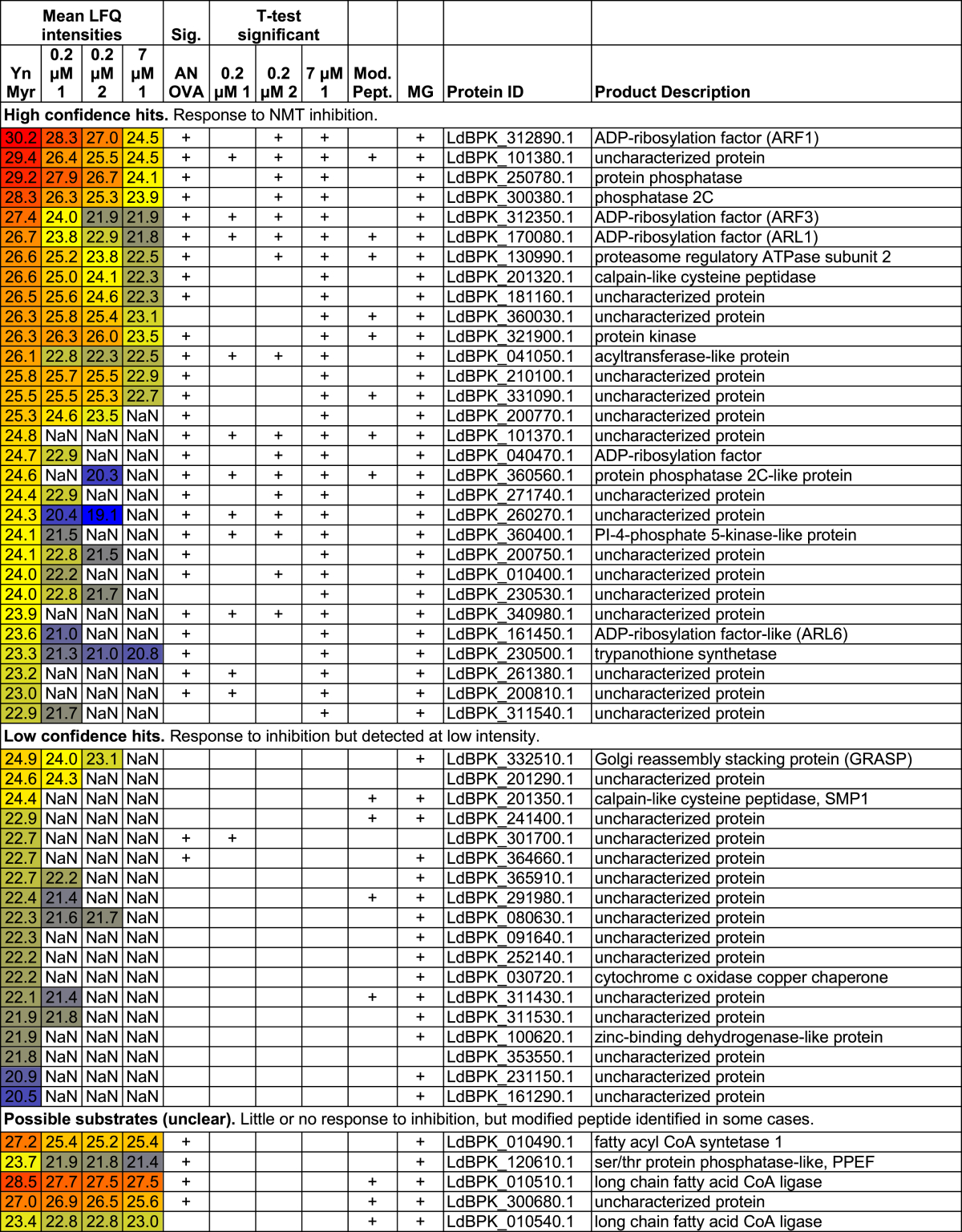
High and Low Confidence NMT Substrates Identified by Quantitative Proteomics with YnMyr in Combination with NMT Inhibitors

Mean log_2_(LFQ intensity) is given and cells are color coded based on value. NaN, not a number (not found); Sig., significant. Proteins for which the YnMyr-modified N terminus was detected are indicated. See also Tables S4 and S6.

## Supplemental Information

Document S1. Figures S1–S6 and Supplemental Experimental Procedures

Table S1. Identification of YnMyr Tagged Proteins in Amastigotes Related to Figure 3

Table S2. Identification of YnMyr Tagged Proteins in Promastigotes Related to Figure 3

Table S3. Comparison of Proteomics in Promastigotes and Amastigotes, and Bioinformatic Annotation of Hits; Related to Figure 3

Table S4. YnMyr Proteomics in the Presence of NMT Inhibitors Related to Figures 5 and 6

Table S5. All N-terminal Glycine-Containing (MG) L. donovani Proteins; Related to Figures 3, 5, and 6

Table S6. Identification of YnMyr Modified Peptides, Related to Figure 6 and Table 1

Document S2. Article plus Supplemental Information
