# Supplementary material for: Global Analysis of Protein N-Myristoylation and Exploration of N-Myristoyltransferase as a Drug Target in the Neglected Human Pathogen Leishmania donovani
Source: Chem Biol. 2015 Mar 19;22(3):342–54. doi: 10.1016/j.chembiol.2015.01.003 (PMC4372256; doi:10.1016/j.chembiol.2015.01.003)
Supplement: Document S1. Figures S1–S6 and Supplemental Experimental Procedures [file mmc1.pdf]

Chemistry & Biology, Volume *22*

Supplemental Information

Global Analysis of Protein *N*-Myristoylation and Exploration of *N*-Myristoyltransferase as a Drug Target in the Neglected Human Pathogen *Leishmania donovani*

Megan H. Wright, Daniel Paape, Elisabeth M. Storck, Remigiusz A. Serwa, Deborah F. Smith, and Edward W. Tate

Supplemental Information

Table of Contents

Supplemental Figures and Tables 2
Supplemental Experimental Procedures 22
Synthesis of AzRB 22
Parasite culture (extra detail) 23
Metabolic tagging experiments (extra detail) 24
Ex vivo amastigotes inhibition assay 24
Macrophage cytotoxicity assay 24
CuAAC labeling and pull-down 24
Gel and Western blot analysis 25
Proteomics experiments 25
Bioinformatics 28
Supplemental References 29

Supplemental Figures and Tables

Supplemental Figures

Figure S1: Additional gel-based data and controls, related to Figures 1 & 2. Time-dependent labeling; competition with myristic acid; sensitivity of labeling to cycloheximide treatment; hydroxylamine treatment of lysates; YnMyr tagging of GP63; YnMyr tagging in *L. major* 18AA-HASPB-GFP strains; structures of AzTB and YnTB.

Figure S2: Proteomics data related to Figure 3. Volcano plots of YnMyr proteomics (t-test); prediction of *S*-palmitoylation for non-MG hits in amastigotes and promastigotes.

Figure S3: Inhibition full gel and blot data, including controls related to Figure 4.

Figure S4: Scatter plots to assess biological and technical reproducibility of inhibition proteomics experiments related to Figure 5.

Figure S5: Inhibitor proteomics additional data related to Figures 5 & 6.

Figure S6: YnMyr modified peptide, assigned spectra related to Figure 6 and Table 1.

Supplemental Tables

Supplemental Tables are provided as separate Excel spreadsheets.

Table S1: Identification of YnMyr tagged proteins in amastigotes related to Figure 3. Identification of proteins following metabolic tagging with Myr or YnMyr, CuAAC, pull-down, tryptic digest and LC-MS/MS analysis. Four replicates were performed independently, starting from the same metabolically labelled lysates (Myr and YnMyr samples). Data processing is described In Supplemental Procedures below (Data processing: YnMyr tagged proteins in amastigotes and promastigotes).

Table S2: Identification of YnMyr tagged proteins in promastigotes related to Figure 3. Identification of proteins following metabolic tagging with Myr or YnMyr, CuAAC, pull-down, tryptic digest and LC-MS/MS analysis. Three replicates were performed independently, starting from the same metabolically labelled lysates (Myr and YnMyr samples). Data processing is described In Supplemental Procedures below (Data processing: YnMyr tagged proteins in amastigotes and promastigotes).

Table S3: **Comparison of proteomics in promastigotes and amastigotes, and bioinformatic annotation of hits; related to Figure 3.** The datasets shown in Tables S1 & S2 were compared. GPI-anchor and *S*-palmitoylation prediction was carried out for hits. Data processing is described In Supplemental Procedures below (Data processing: YnMyr tagged proteins in amastigotes and promastigotes).

Table S4: **YnMyr proteomics in the presence of NMT inhibitors related to Figures 5 & 6.** Amastigotes were metabolically tagging with Myr or YnMyr in the presence of inhibitors 1 or 2 at indicated concentrations; CuAAC, pull-down, tryptic digest and LC-MS/MS analysis were performed to identify proteins. Two technical replicates (sample preparation from the lysates) of two biological replicates were carried out. Data processing is described In Supplemental Procedures below (Data processing: YnMyr tagging in the presence of NMT inhibitors (amastigotes)).

Table S5: All N-terminal glycine-containing (MG) *L. donovani* proteins; related to Figures 3, 5 & 6. Prediction of *N*-myristoylation by bioinformatic tools and cross-referencing to current study was performed for all *L. donovani* MG proteins in the database.

Table S6: Identification of YnMyr modified peptides, related to Figure 6 and Table 1. Proteomic data were further analyzed by de novo-aided sequencing to identify peptides modified by the the lipid-derived adduct (+534.3278 Da) on any amino acid at peptide N-terminus. All peptides reported are N-terminal peptides unique for the protein that they were assign to by the software.

Figure S1. Additional labeling data for *L. donovani* promastigotes. Related to Figs. 1 & 2. **A.** *Ld* promastigotes were cultured for the indicated timepoints in the presence of myristic acid (M) or YnMyr (Yn), lysed and proteins subject to CuAAC reaction and analysis by in-gel fluorescence. **B.** Promastigotes were labeled with YnMyr in the presence of varying amounts of myristic acid (Myr). **C.** YnMyr incorporation into proteins is dependent on protein synthesis, but incorporation into putative glycolipid is less affected. Promastigotes were tagged with myristic acid (-) or YnMyr in the presence of 50 µM cycloheximide (CHX) for indicated timepoints and samples processed by CuAAC. **D.** Treatment of YnMyr tagged samples with base (NaOH) or hydroxylamine (HA; pH7). C/M: precipitation with $CHCl_3$/MeOH. **E.** Analysis of YnMyr tagged samples by Western blot for GPI-anchored protein GP63 after CuAAC and pull-down. Click: sample reacted with CuAAC reagents; PD B: sample pulled-down onto streptavidin-coated beads and proteins eluted from beads by boiling. * = non-specific band, streptavidin monomer. Note that the percentage incorporation of YnMyr is clearly low, as it must compete with endogenous lipid. Numbers are molecular weight markers in kDa. **F.** Related to Fig. 2; total protein labeling in HASPB GFP-fusion strains. **G.** Structures of AzTB and YnTB.

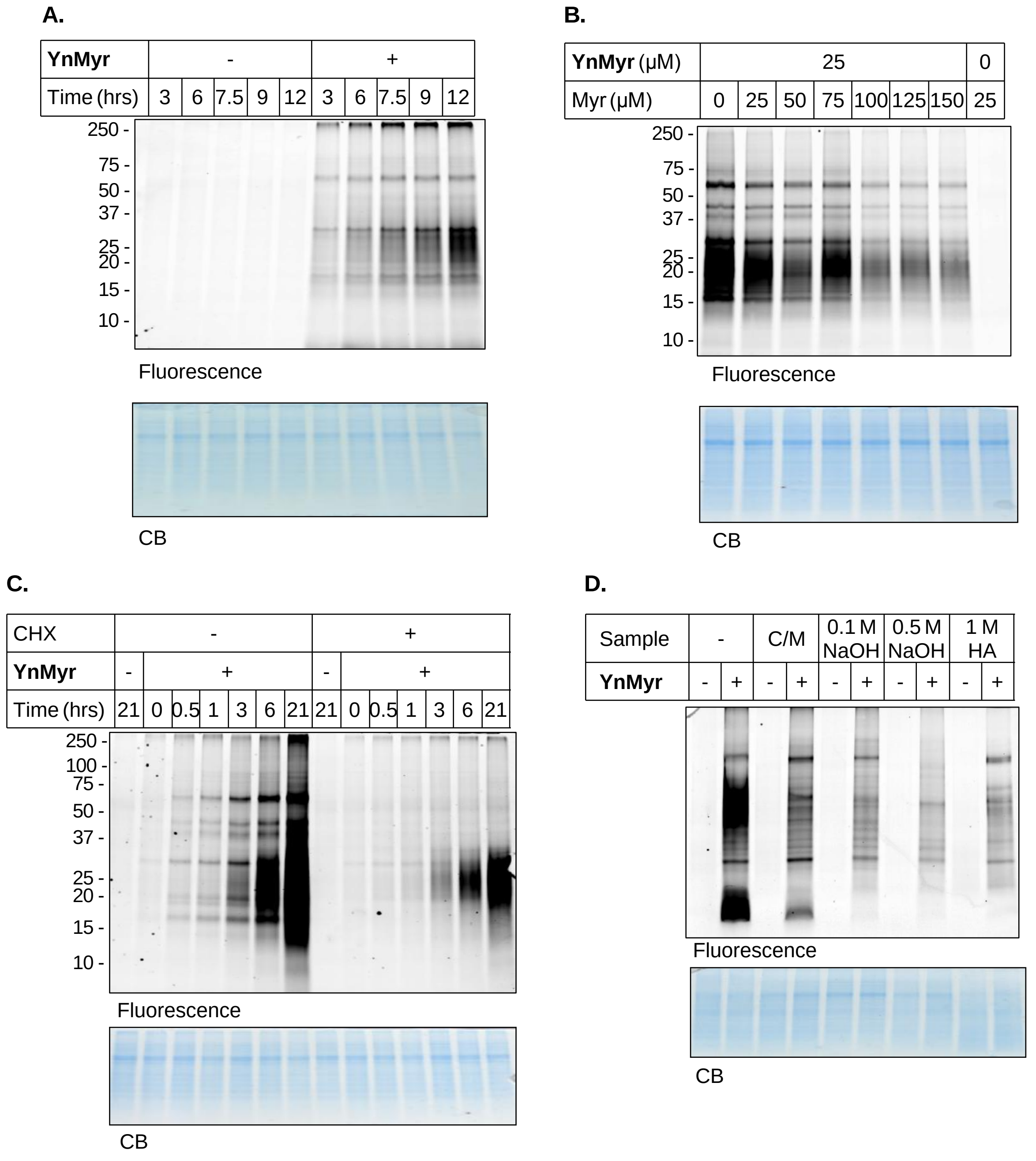

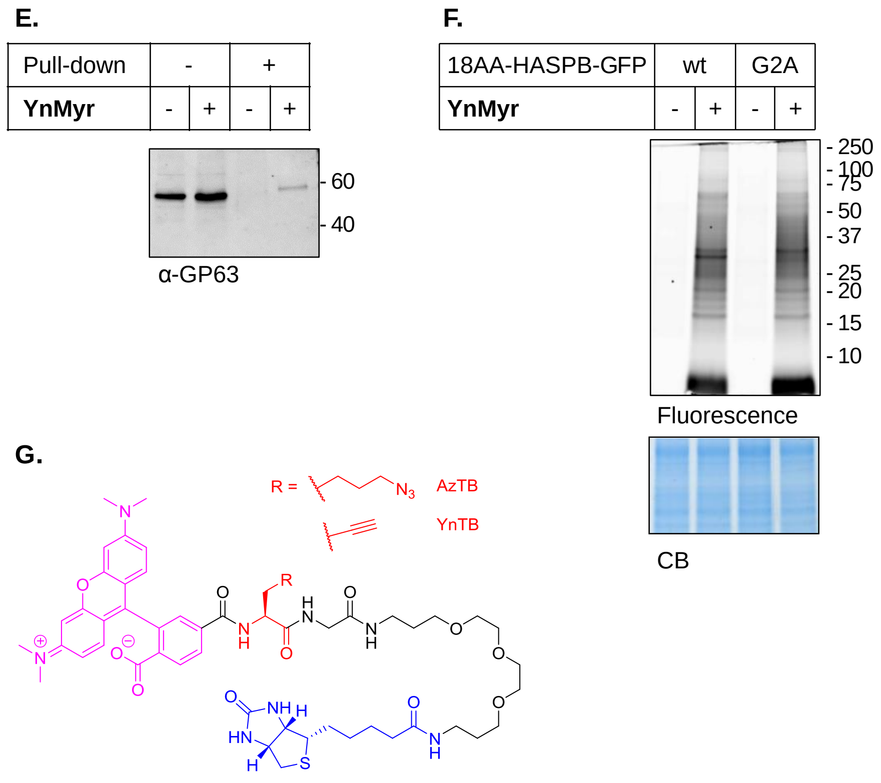
E.
Pull-down
-
+
YnMyr
- + - +
60
40
α-GP63
F.
18AA-HASPB-GFP
wt
G2A
YnMyr
- + - +
250
100
75
50
37
25
20
15
10
Fluorescence
CB
G.
R =
AzTB
YnTB

Figure S2: YnMyr proteomics in amastigotes and promastigotes; related to Fig. 3. Permutation-corrected two-sample t-tests were applied to compare YnMyr and myristic acid (Myr) controls after imputation of missing values. An LFQ enrichment ratio was calculated from the ratio of YnMyr and Myr LFQ intensities (mean). 'High confidence': protein significant and absent from controls; 'low abundance': protein absent from myristic acid controls but did not reach statistical significance due to low intensity in the YnMyr samples; 'Other significant': protein significant but present in controls **A.** T-test in promastigotes. The 274 proteins present in all three replicates. T-test: FDR = 0.01, S0 = 1, 250 permutations. **B.** T-test in amastigotes. T-test: FDR = 0.05, S0 = 1, 250 permutations. **C.** & **D.** Volcano plots with prediction of *S*-palmitoylation using CSS-Palm (Ren et al., 2008) indicated for non-MG proteins in amastigotes and promastigotes (see also Supp. Table S3).

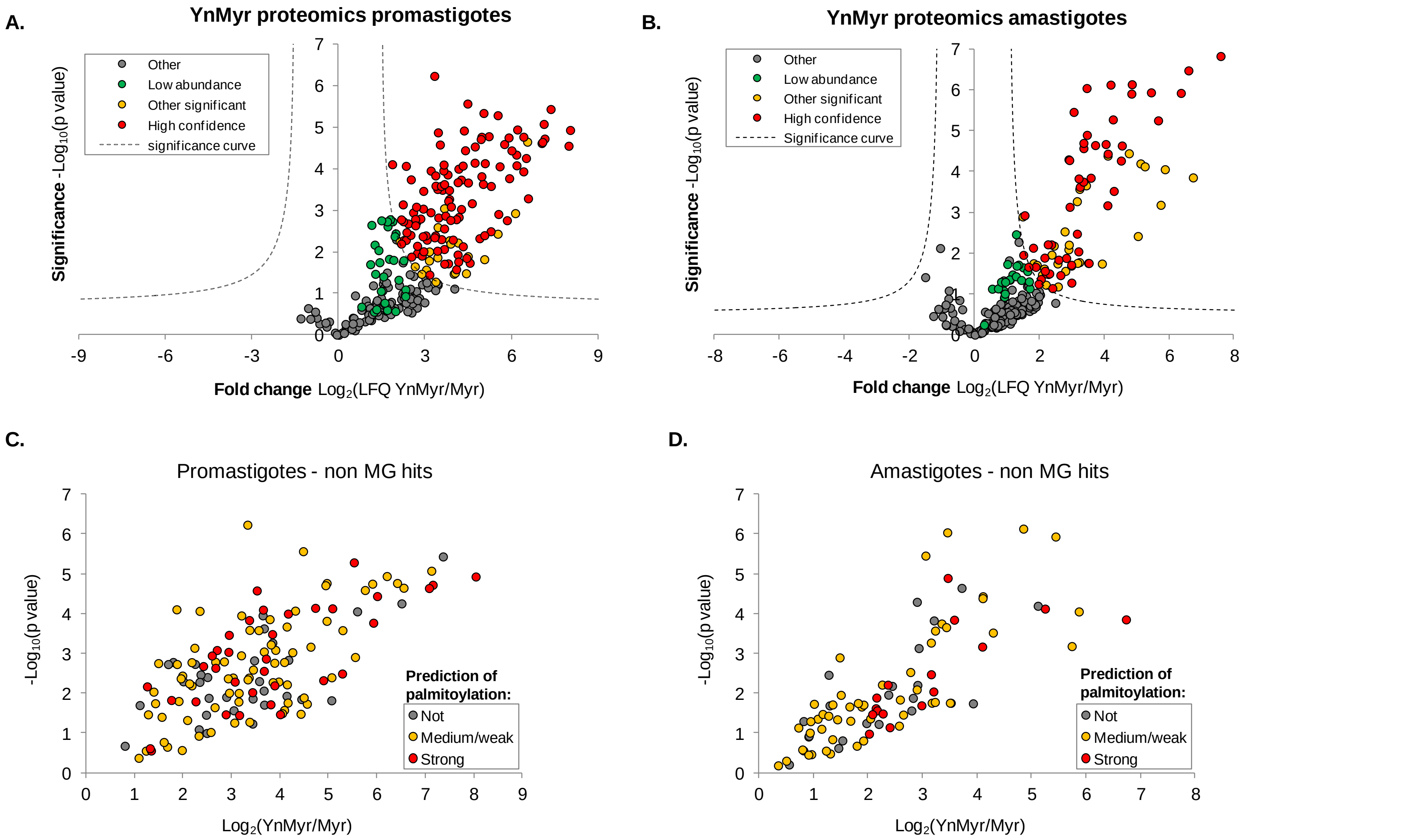

Figure S3: Related to Fig. 4; full blots and control gels for inhibition experiments. **A.** Coomassie (CB) staining for the gel shown in Fig. 4B. **B.** Western blot against HASPB (portion of image boxed with dashed line is shown in Fig. 4B) before and after pull-down of HASPB onto streptavidin beads after CuAAC in promastigotes. Four-fold more protein is loaded after pull-down compared to before. **C.** Coomassie staining for the gel shown in Fig. 4C. **D.** Western blots against HASPB before and after pull-down of HASPB in amastigotes. Four-fold more protein loaded after pull-down compared to before. Top and bottom are replicate experiments performed independently starting from the same lysate. **E.** Independent biological experiment processed in the same way as those in D. Numbers are molecular weights in kDa.

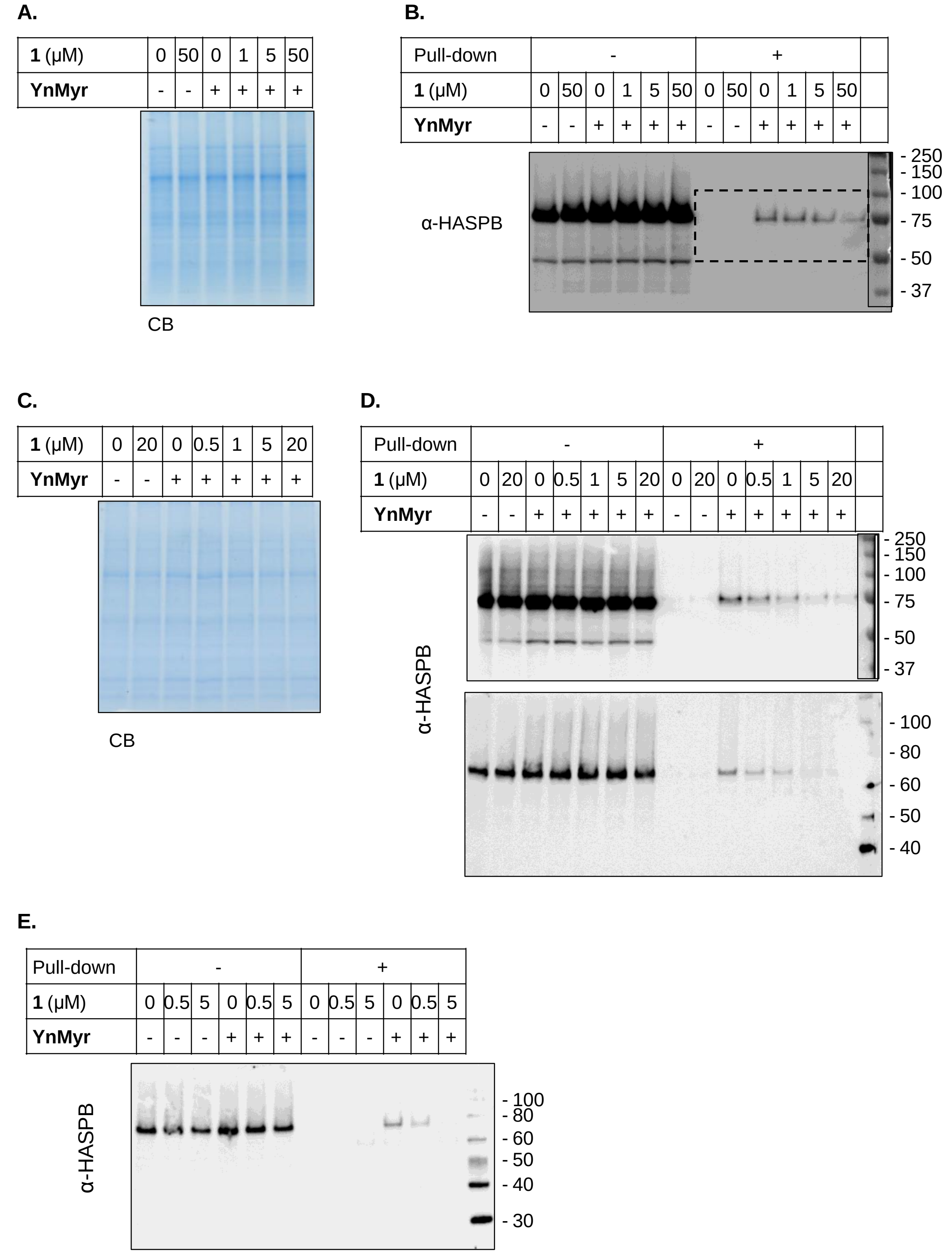

Figure S4: Related to Fig. 5. Multiscatter plots to assess biological and technical reproducibility of replicate inhibition proteomics samples. A & B: technical replicates of first biological replicate; C & D: technical replicates of second biological replicate. Dataset consists of 580 proteins identified in both independent biological experiments; Log_2(LFQ intensity) is plotted after imputation of missing values. Numbers in the top left corner of each plot indicates R^2 value (correlation); proteins containing an N-terminal glycine are coloured red, other proteins are black.

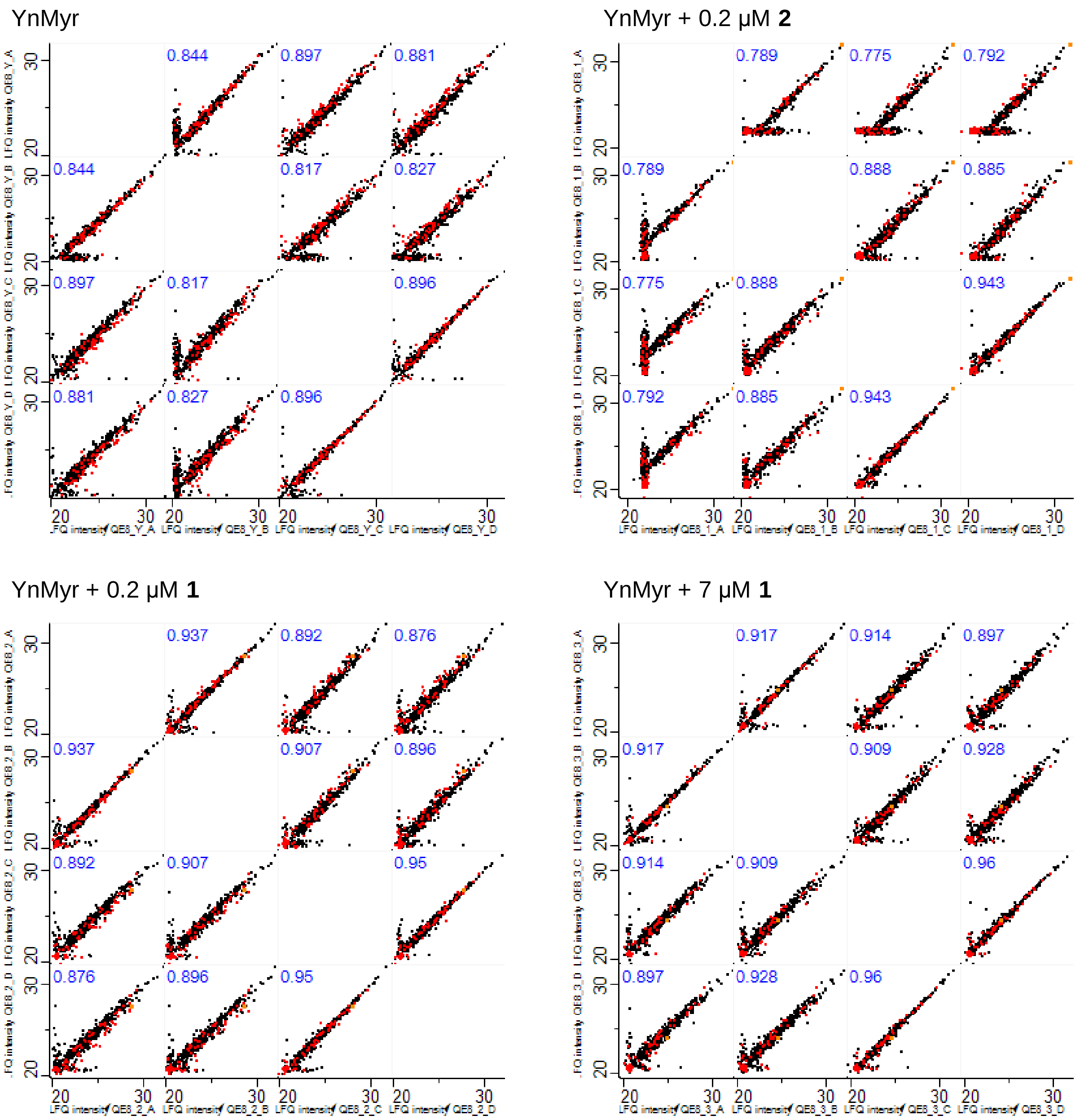

Figure S5: YnMyr inhibition proteomics; related to Figs. 5 & 6. **A.** T-test comparing 7 µM **1** (left) or 0.2 µM **1** (right) and YnMyr datasets, after removal of non-specific binders. FDR = 0.001, S0 = 1, 250 permutations. **B.** T-test comparing 0.2 µM **2** and YnMyr datasets, after removal of non-specific binders. FDR = 0.001, S0 = 1, 250 permutations. **C.** T-test comparing response to 0.2 µM **1** and 7 µM **1**: a ratio of LFQ intensities for YnMyr/(inhibitor treatment) was calculated for each replicate experiment (A-D) and a two-sample t-test (FDR 0.05, S0 1) performed to compare the response to the two inhibitors. **D.** Profile plots of the response of selected MG proteins to inhibition. Log_2(mean LFQ intensity) across four replicates. As for main text Figure 5C but including data for 0.2 µM **2**.

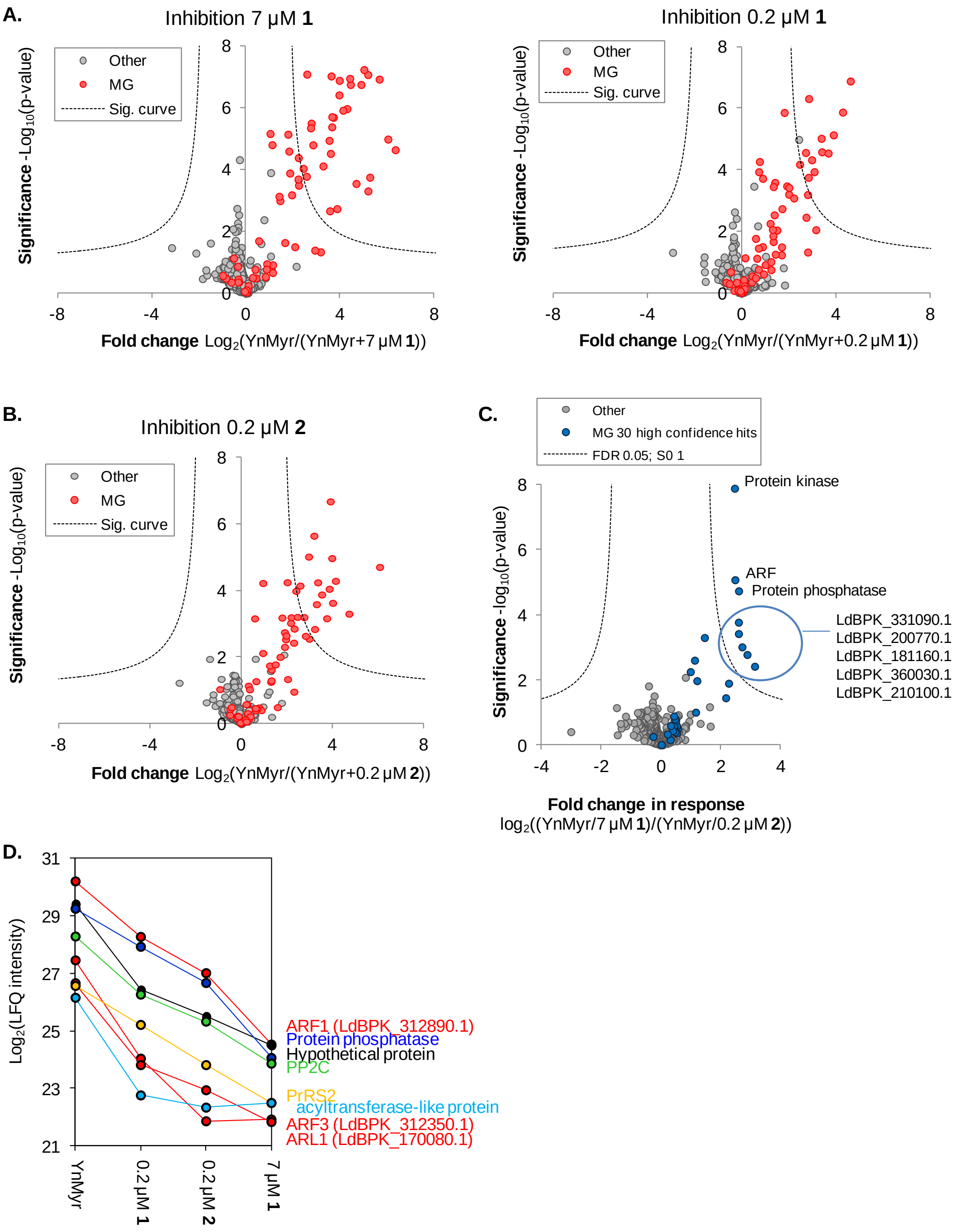

Figure S6: assigned spectra for YnMyr modified peptides. Related to Fig. 6 and Table S6.

LDBPK_231190.1 (uncharacterized protein)

Experiment PRO2

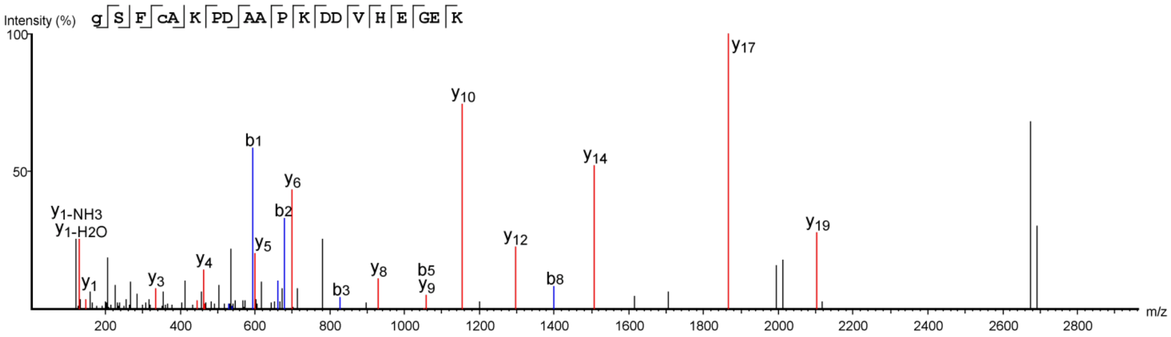

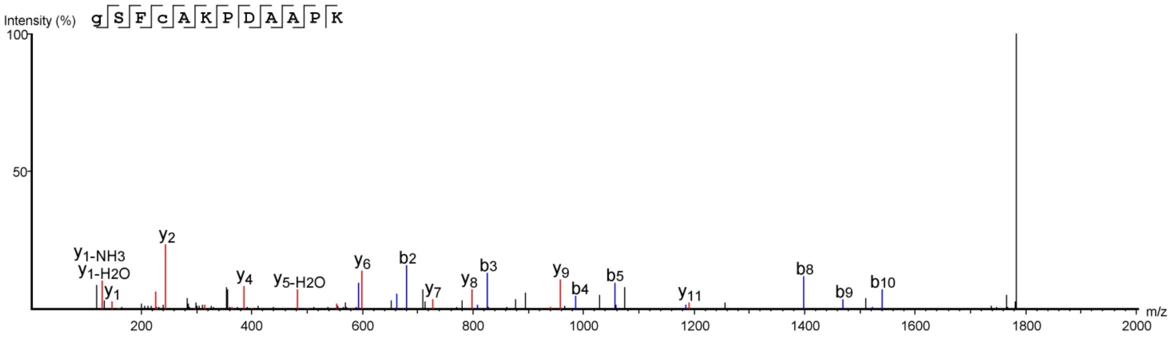

Experiment PRO3

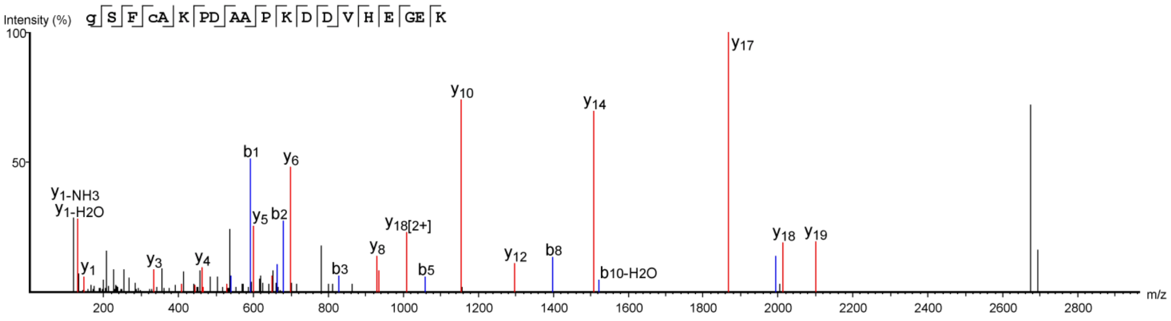

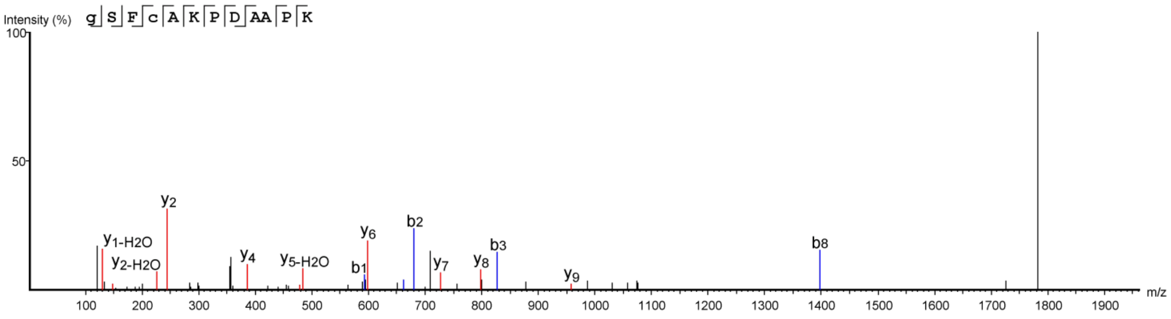

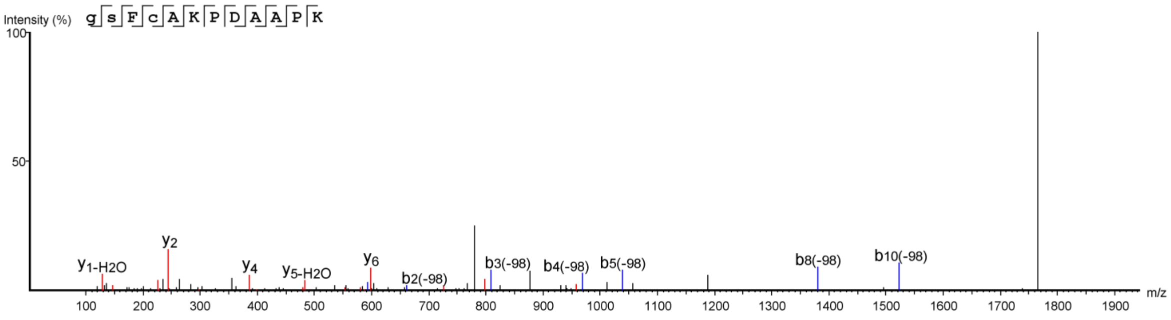

LDBPK_101370.1 (uncharacterized protein)

Experiment PRO2

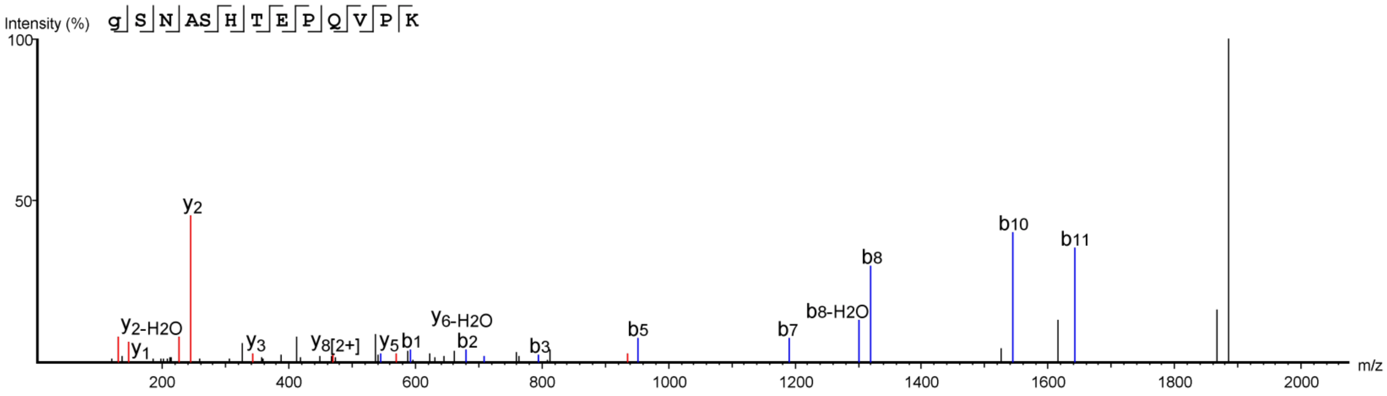

Experiment PRO3

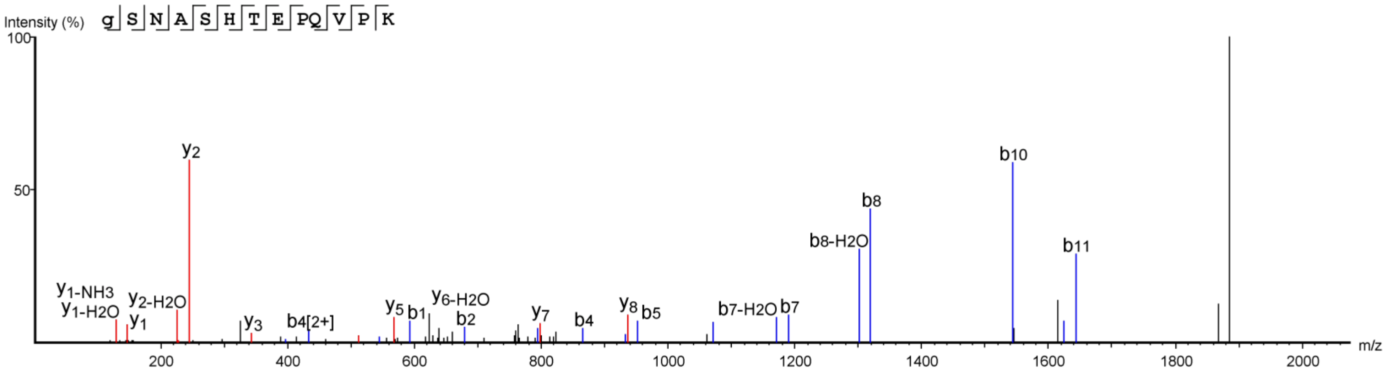

Experiment Inhib. YN_B

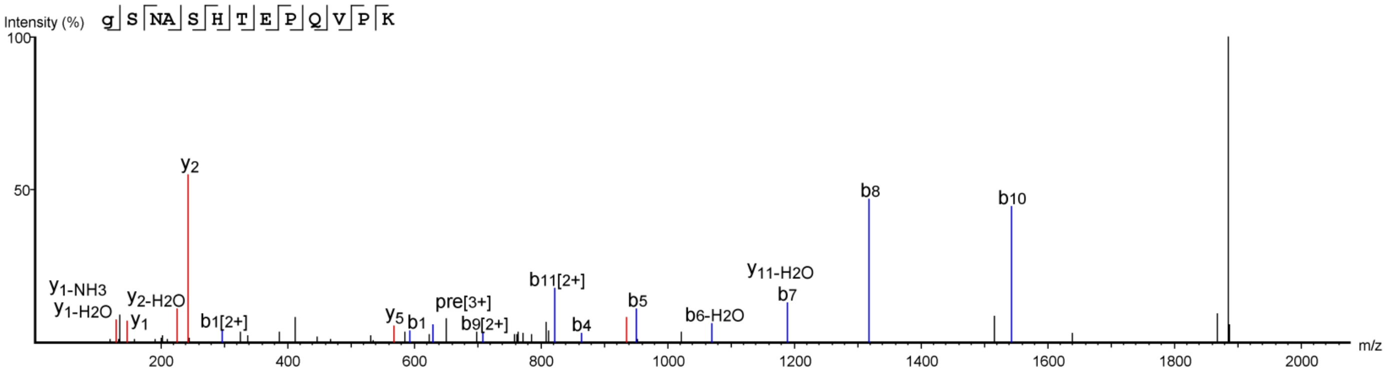

LDBPK_201260.1 (Uncharacterized protein)

Experiment PRO3

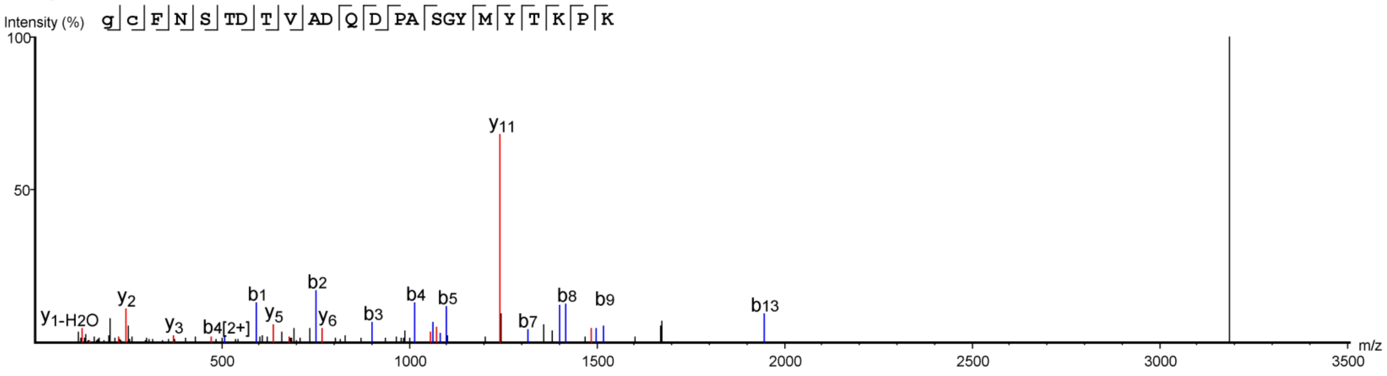

LDBPK_101380.1 (uncharacterized protein)

Experiment PRO2

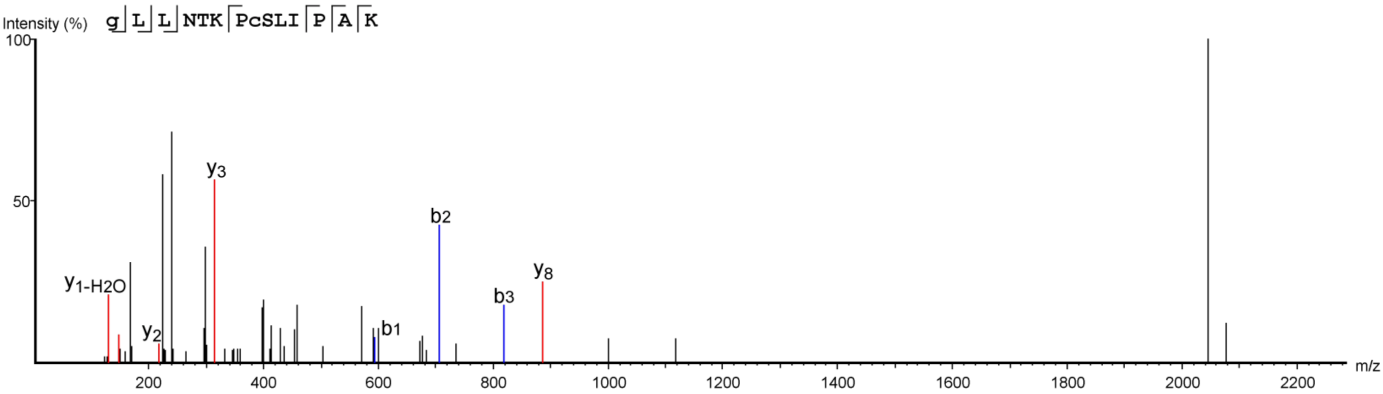

Experiment PRO3

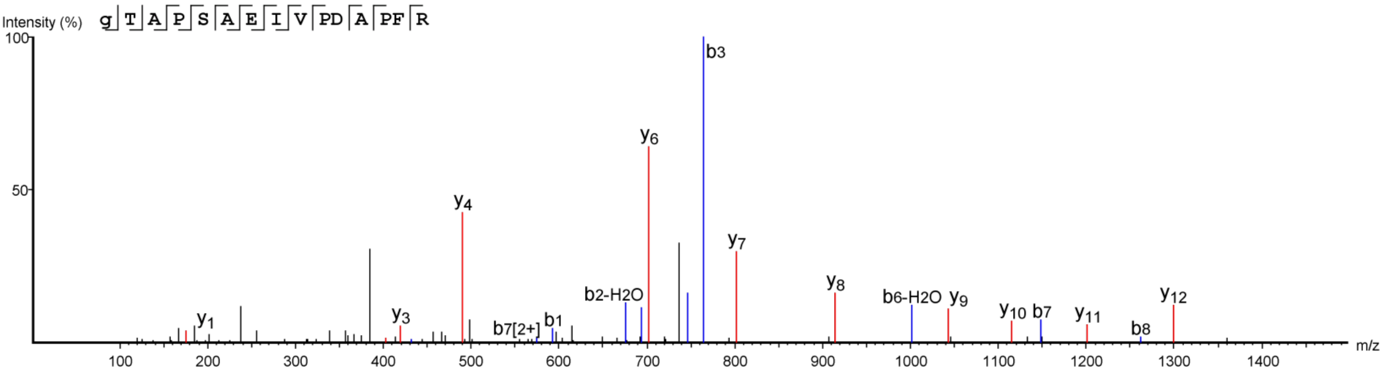

Experiment AM3

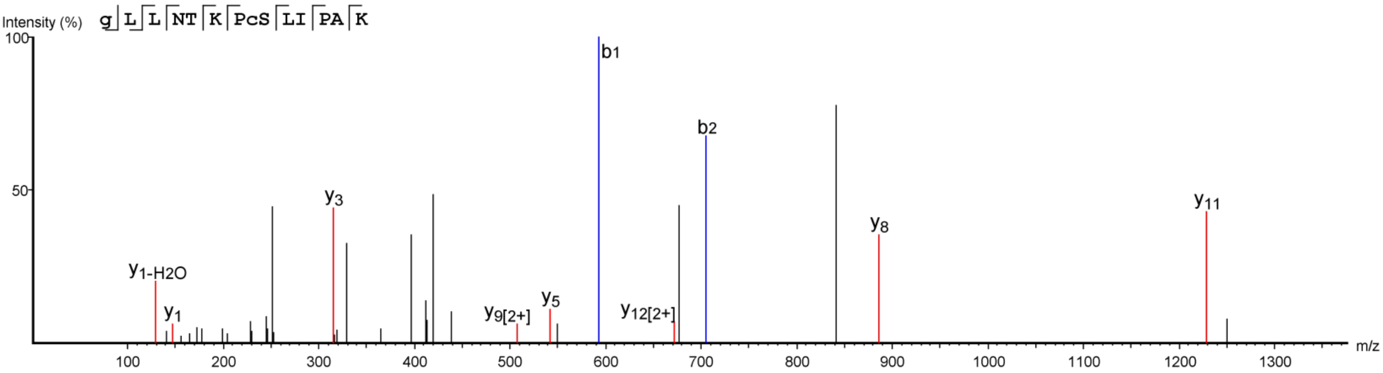

Experiment AM4

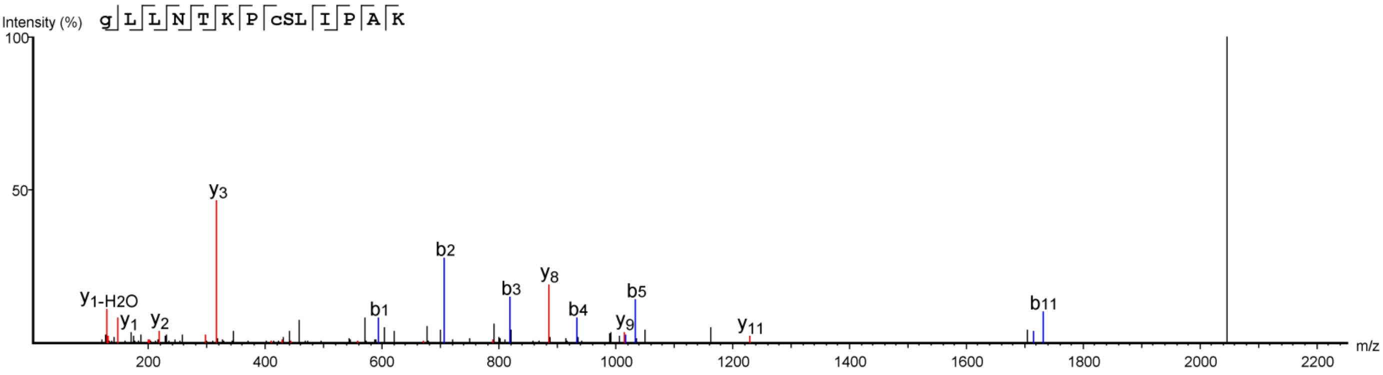

LDBPK_101380.1 (uncharacterized protein) cont.

Experiment Inhib. YN_A

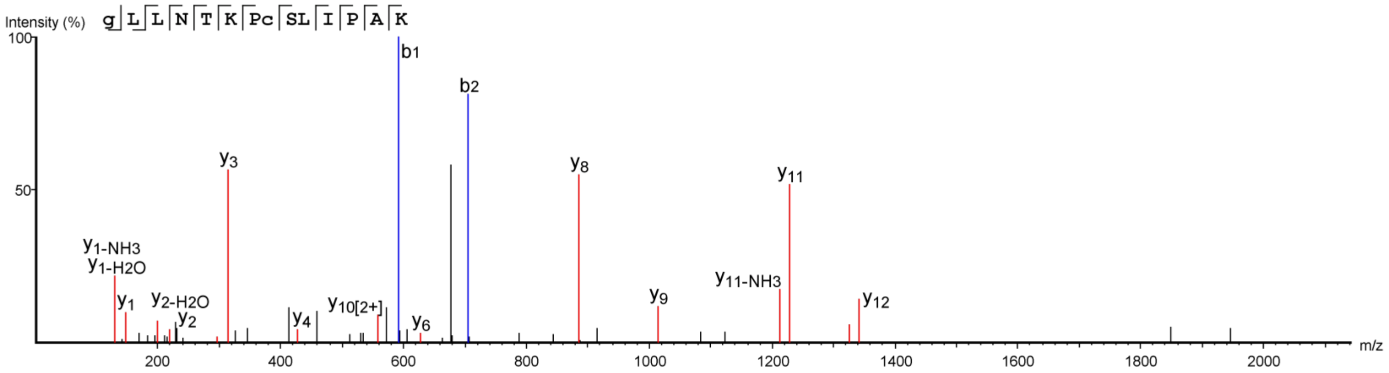

Experiment Inhib. YN_B

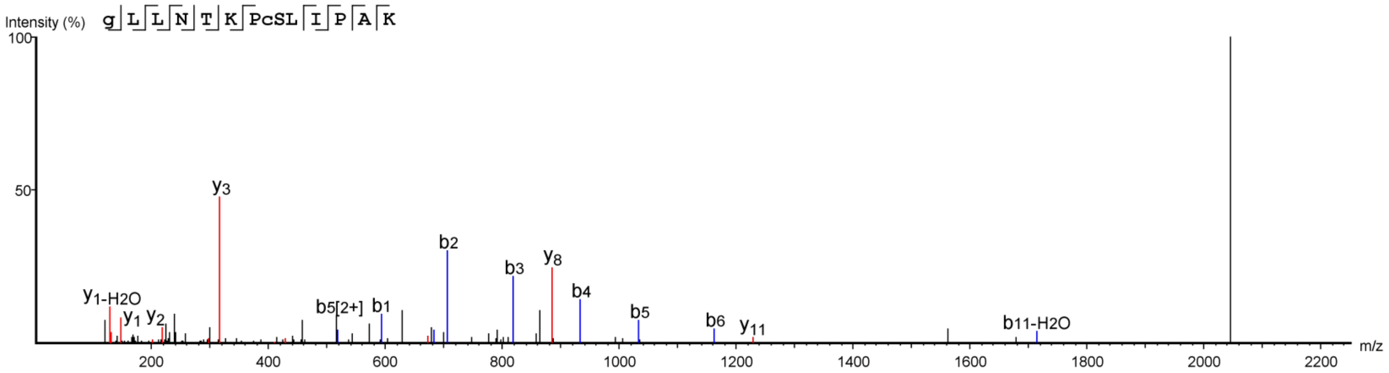

Experiment Inhib. YN_C

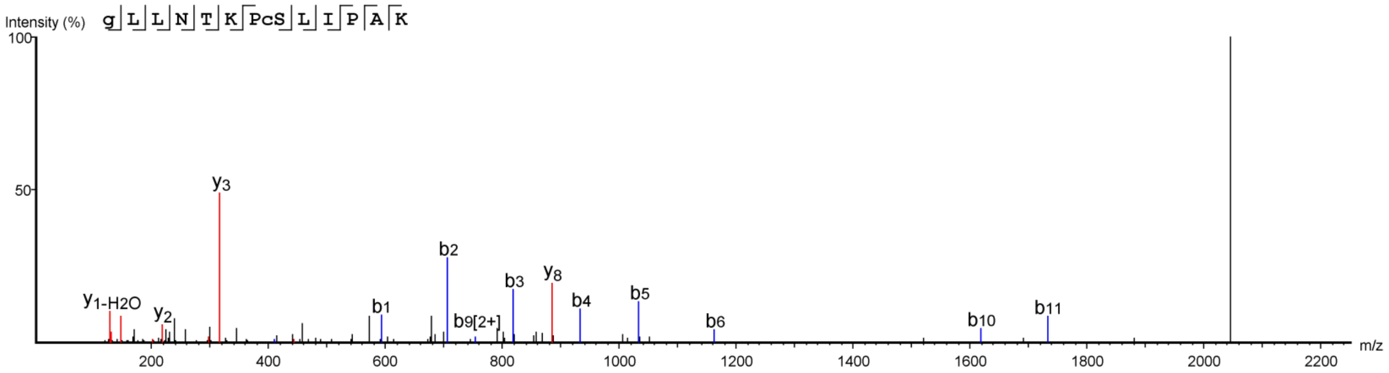

Experiment Inhib. YN_D

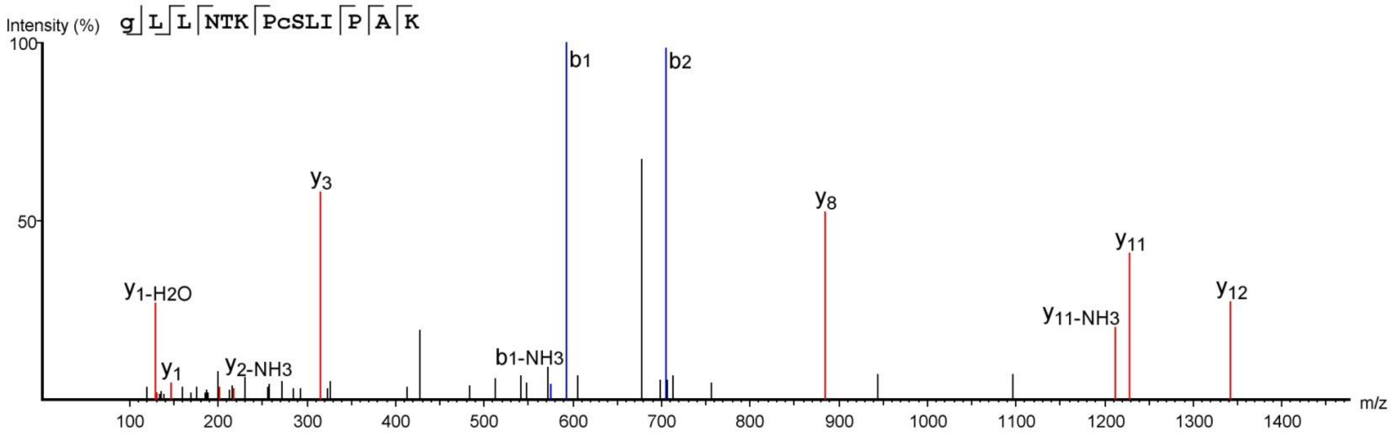

LDBPK_360560.1 (PP2C-like protein)

Experiment PRO3

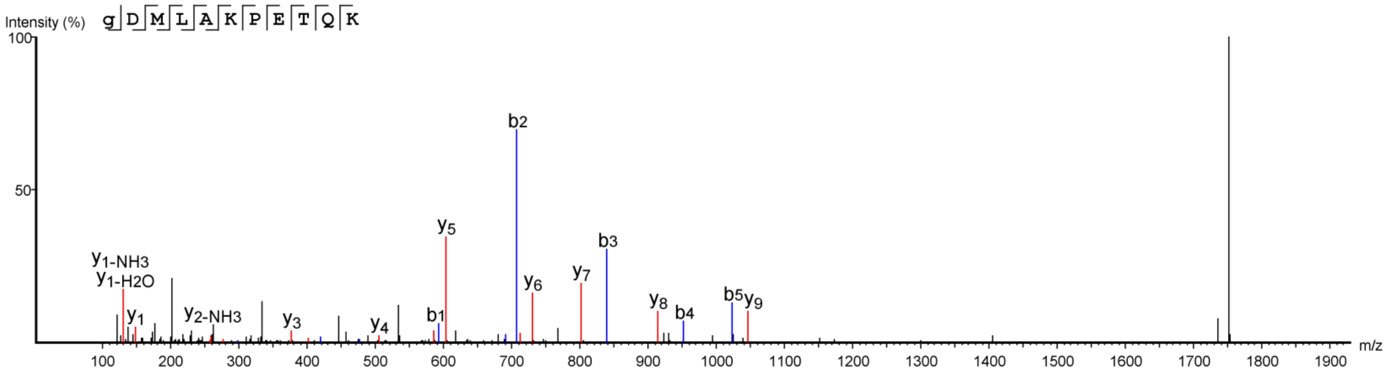

Experiment AM3

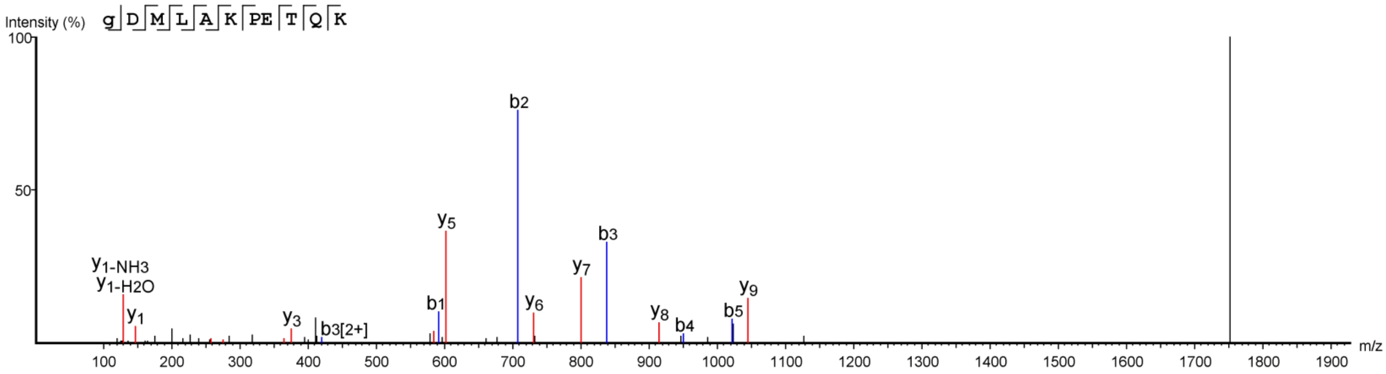

Experiment Inhib. YN_A

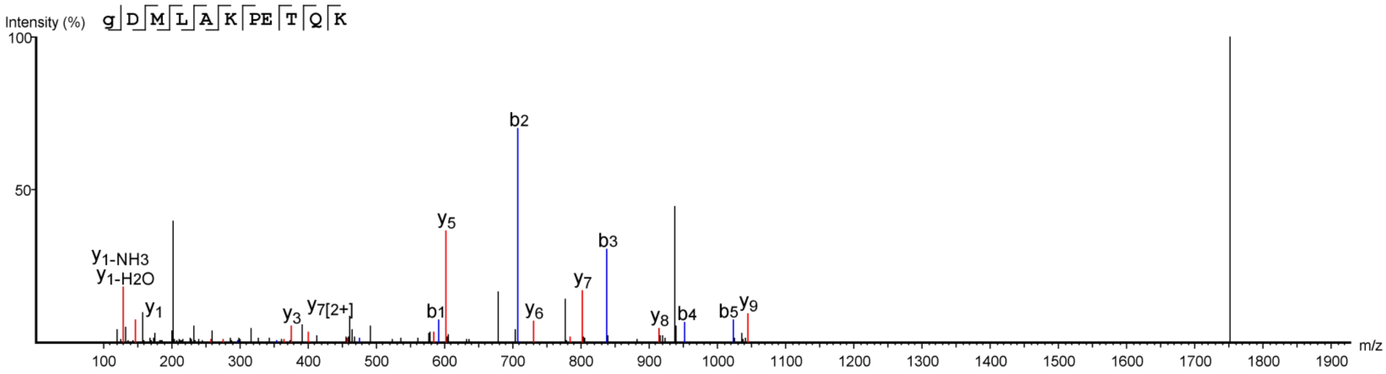

LDBPK_160930.1 (Flagellar calcium-binding protein)

Experiment PRO3

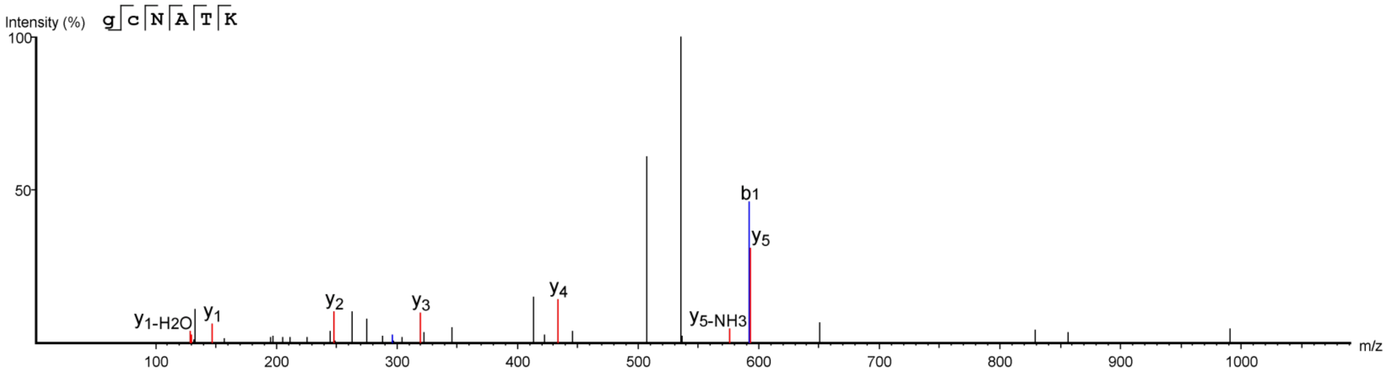

LDBPK_010510.1 (Long chain fatty acid CoA ligase)

Experiment PRO3

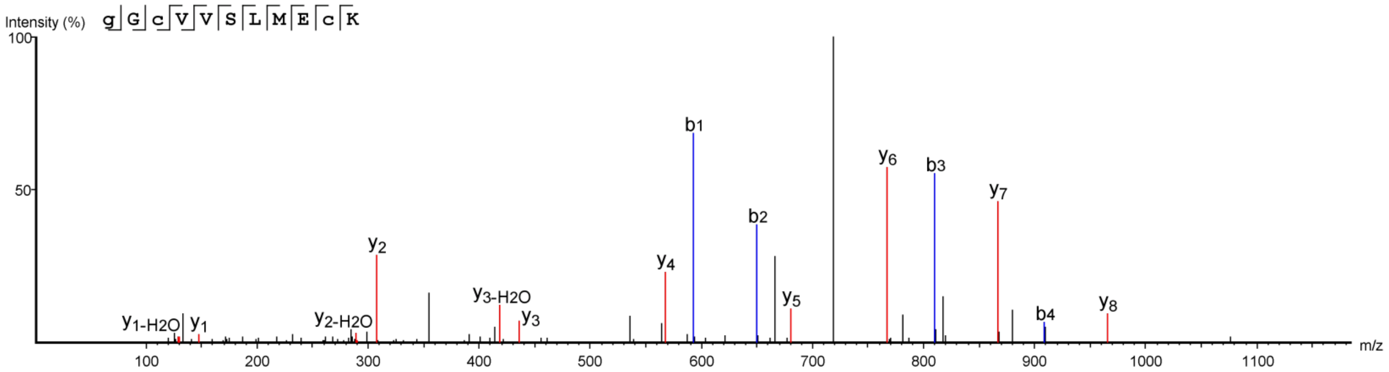

Experiment Inhib. YN_B

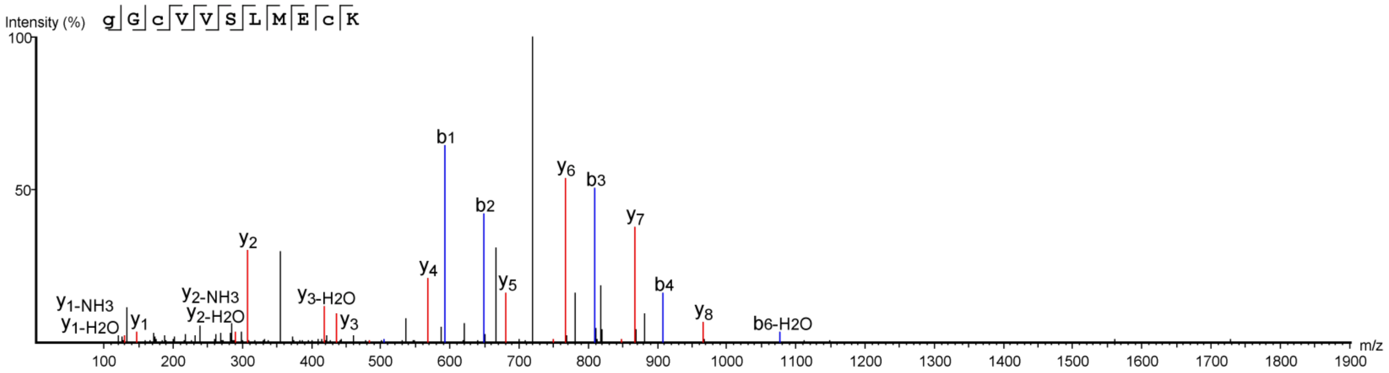

Experiment Inhib. YN_C

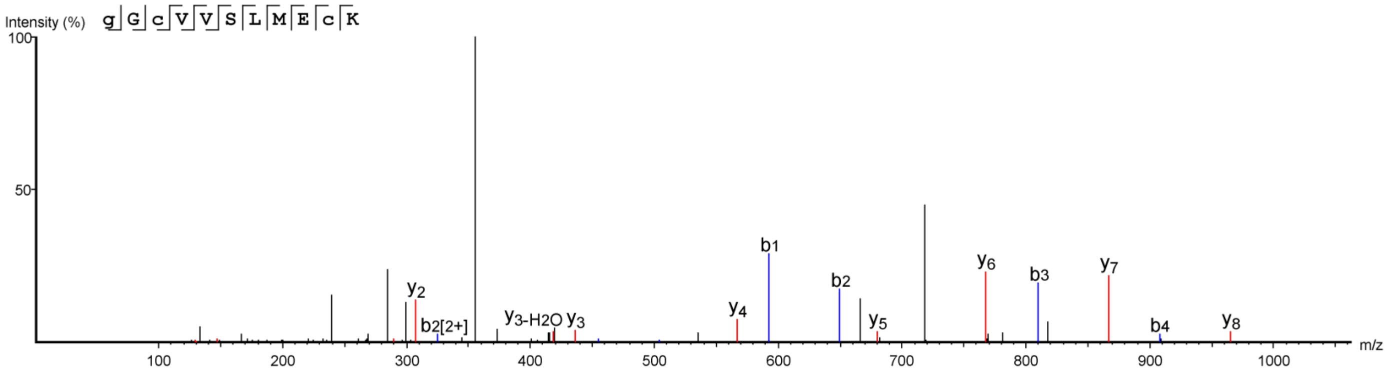

LDBPK_366730.1 (Uncharacterized protein)

Experiment Inhib. Y_A

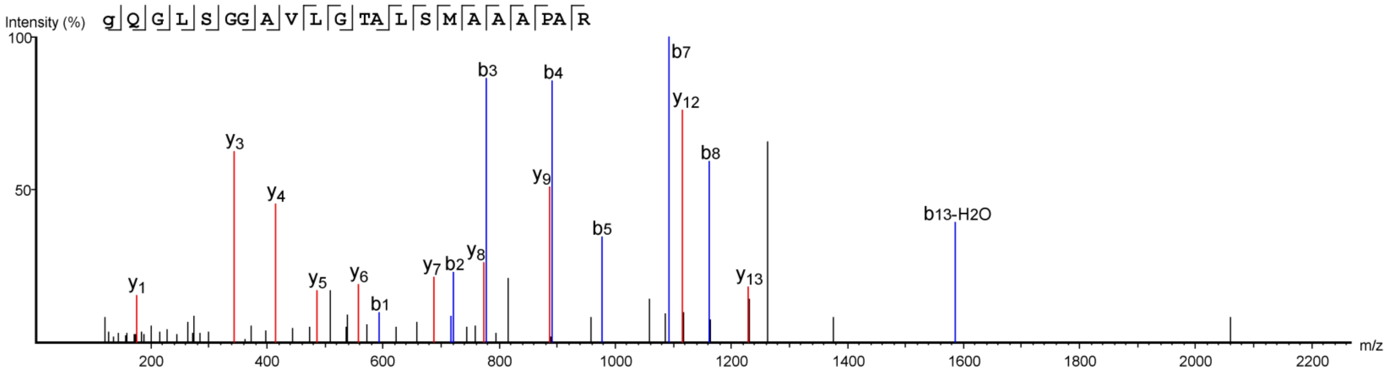

LDBPK_300680.1 (uncharacterized protein)

Experiment PRO3

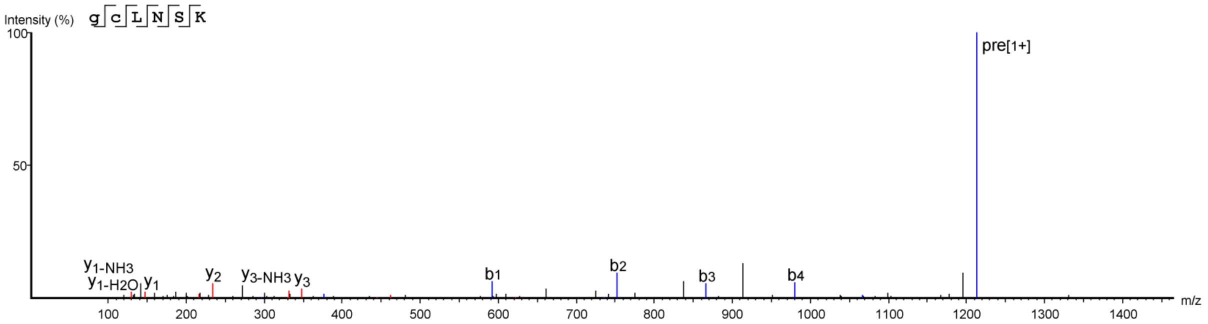

Experiment AM4

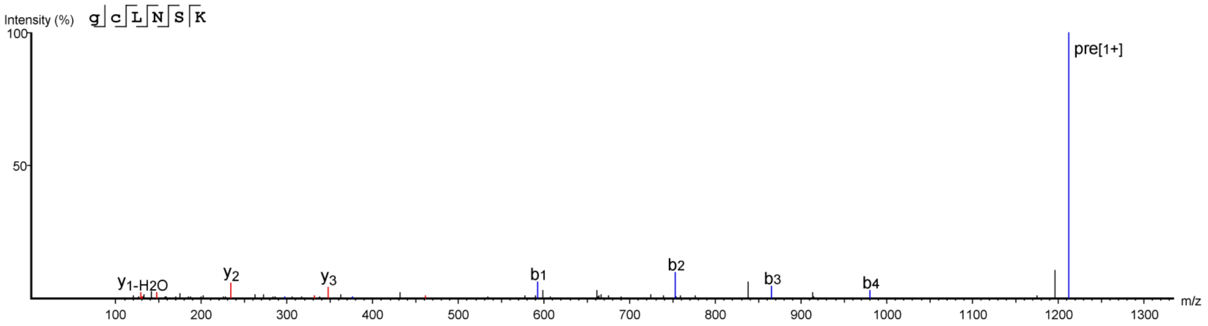

Experiment Inhib. YN_A

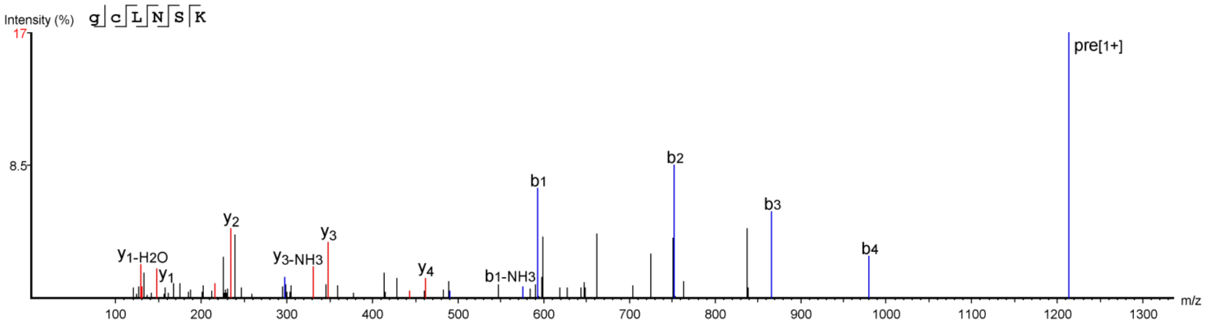

Experiment Inhib. YN_C

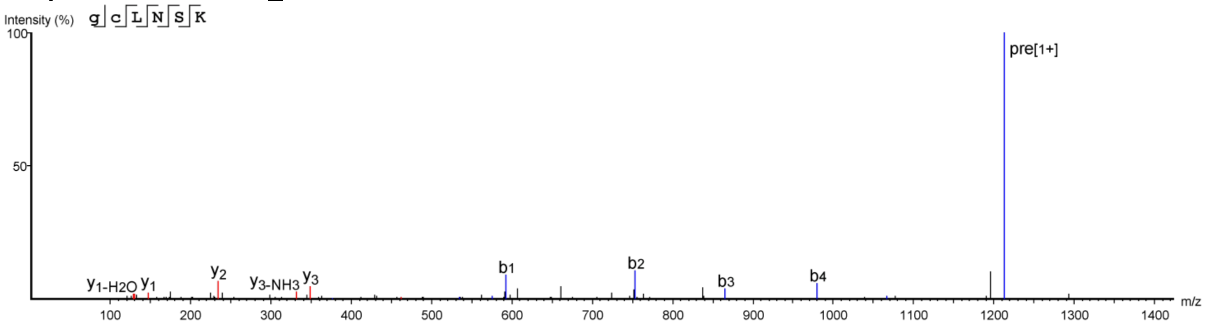

Experiment Inhib. YN_D

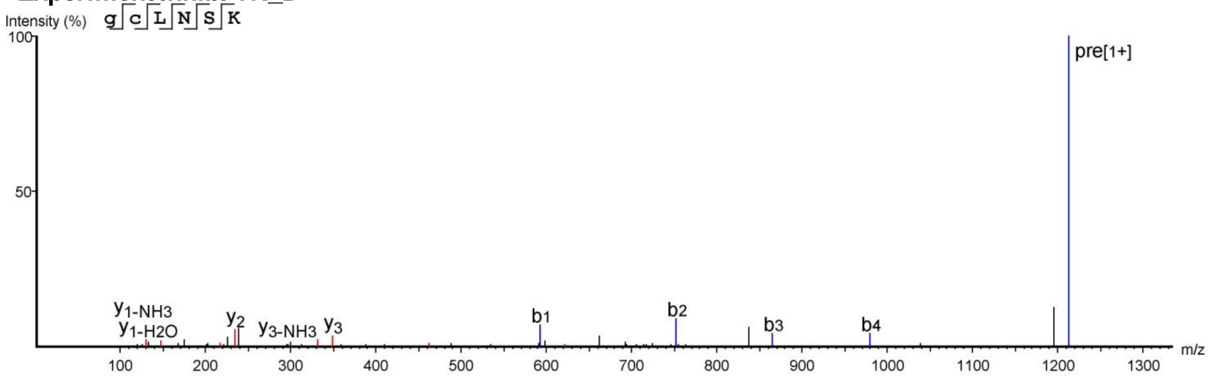

LDBPK_010540.1 (Long chain fatty acid CoA ligase)

Experiment PRO3

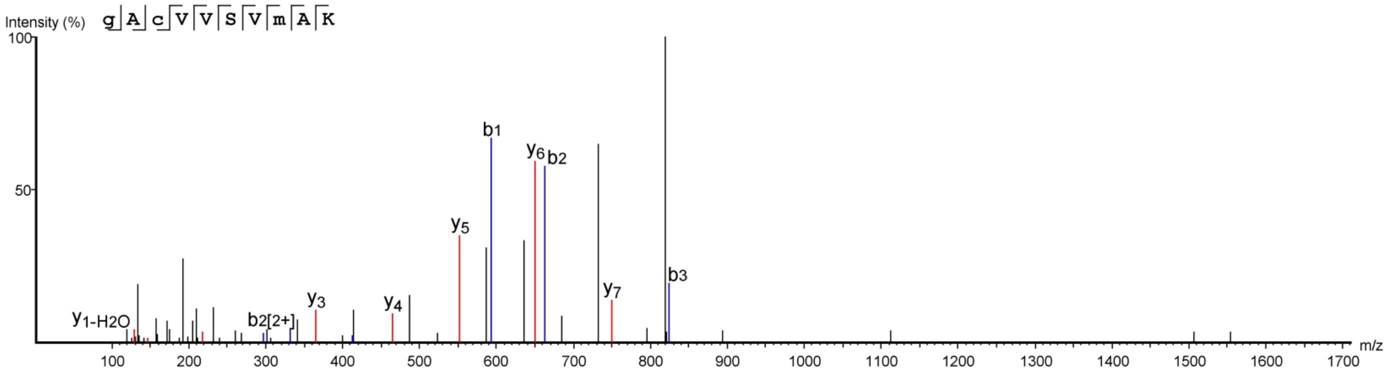

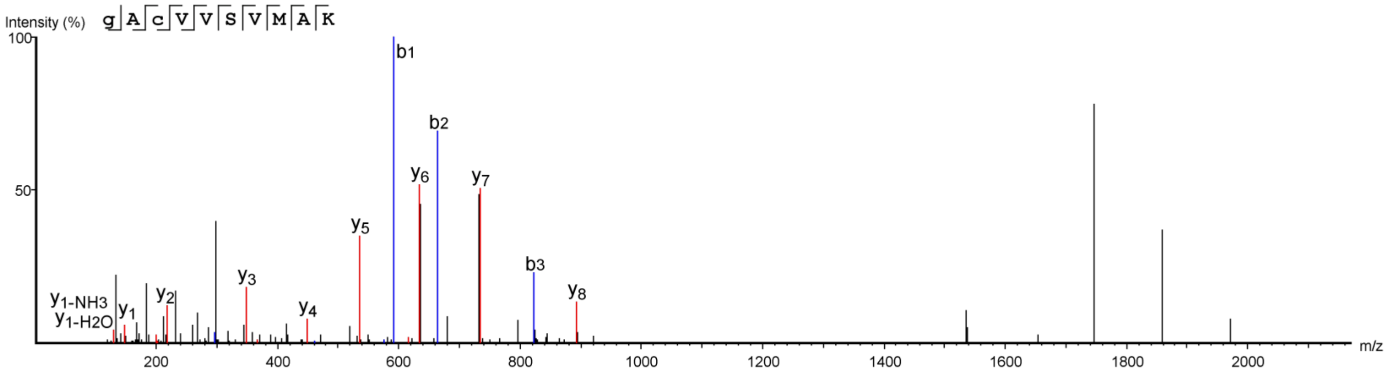

LDBPK_291980.1 (Uncharacterized protein)

Experiment PRO3

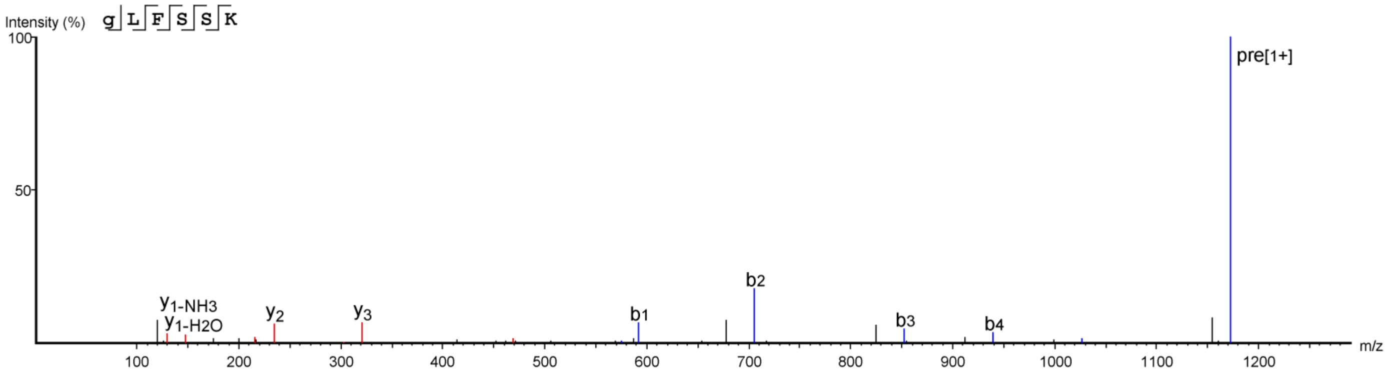

Experiment Inhib. Y_C

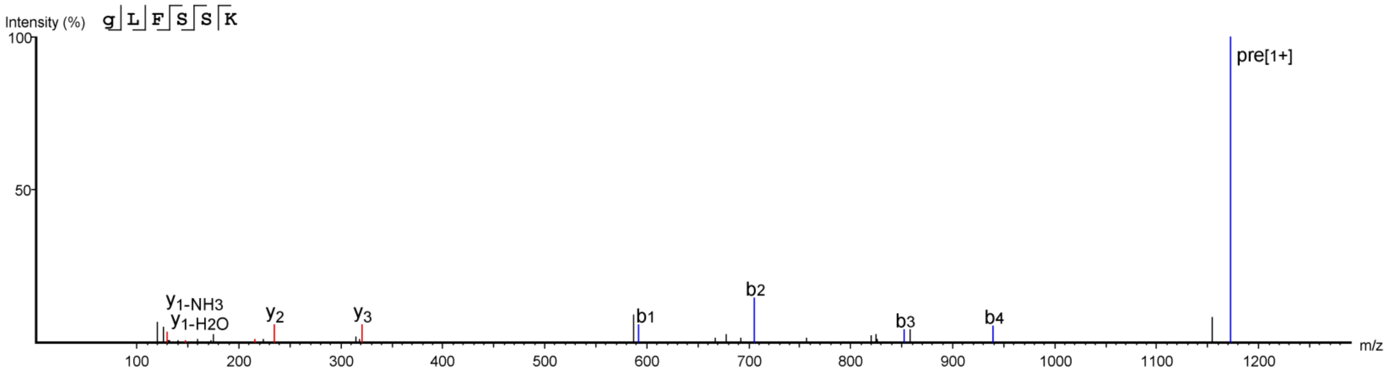

LDBPK_170080.1 (ARF)

Experiment PRO3

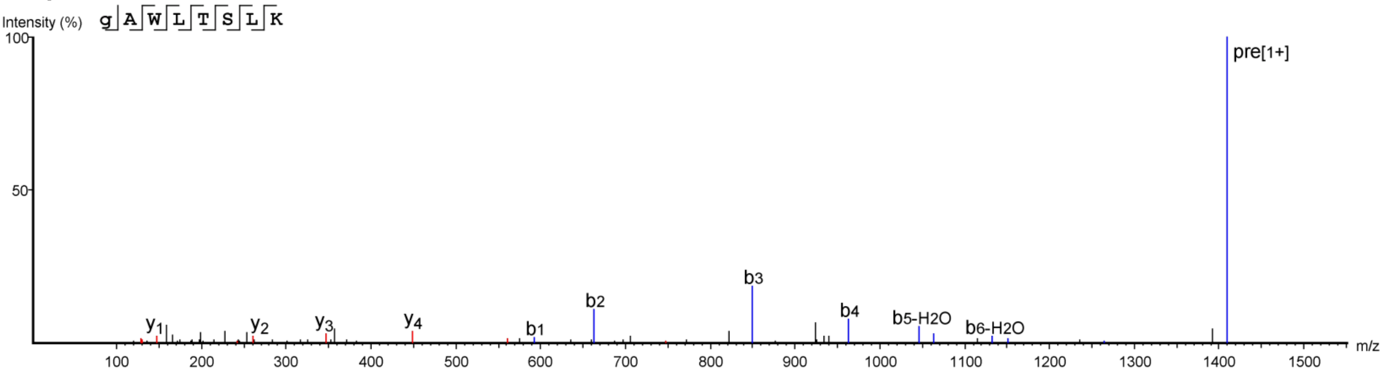

Experiment Inhib. Y_C

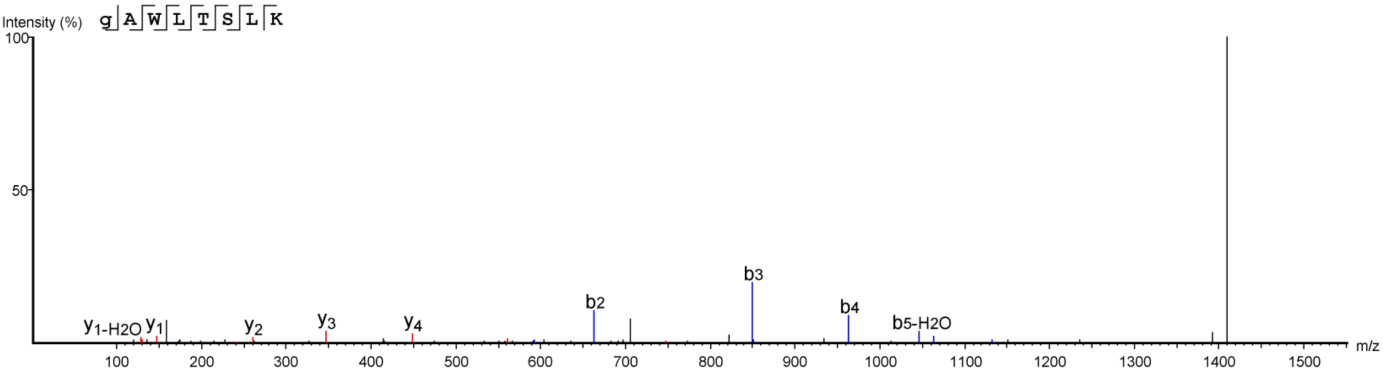

LDBPK_201350.1 (Calpain-like cysteine peptidase)

Experiment PRO3

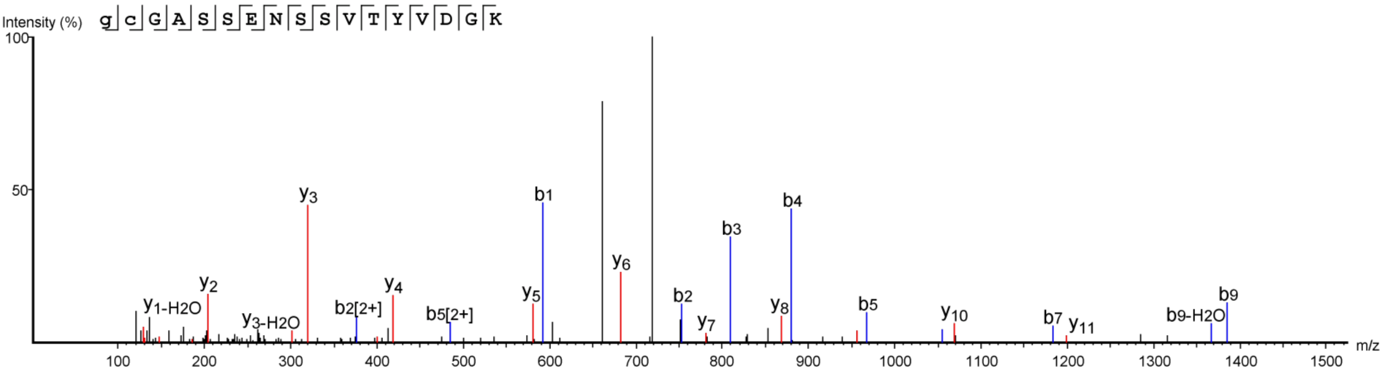

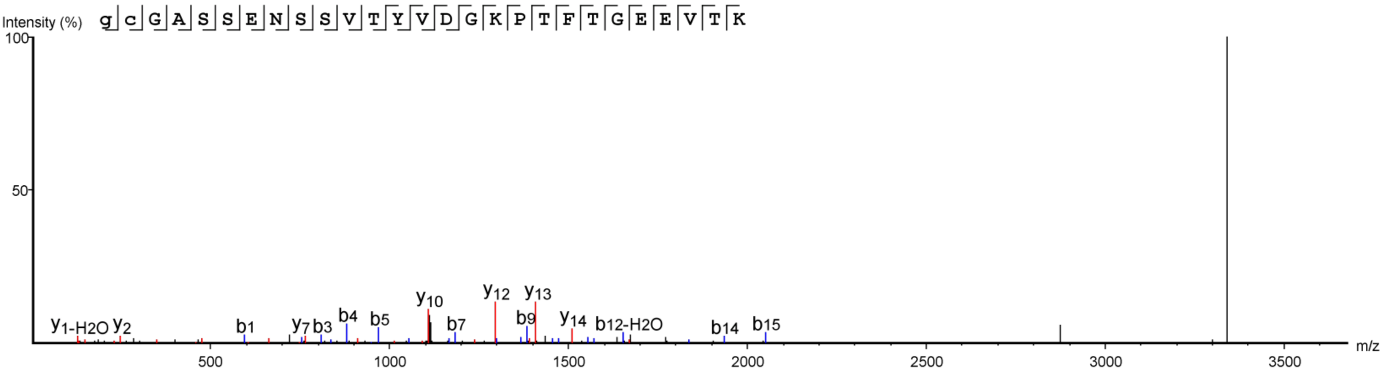

LDBPK_ 331090.1 (Uncharacterized protein)

Experiment PRO3

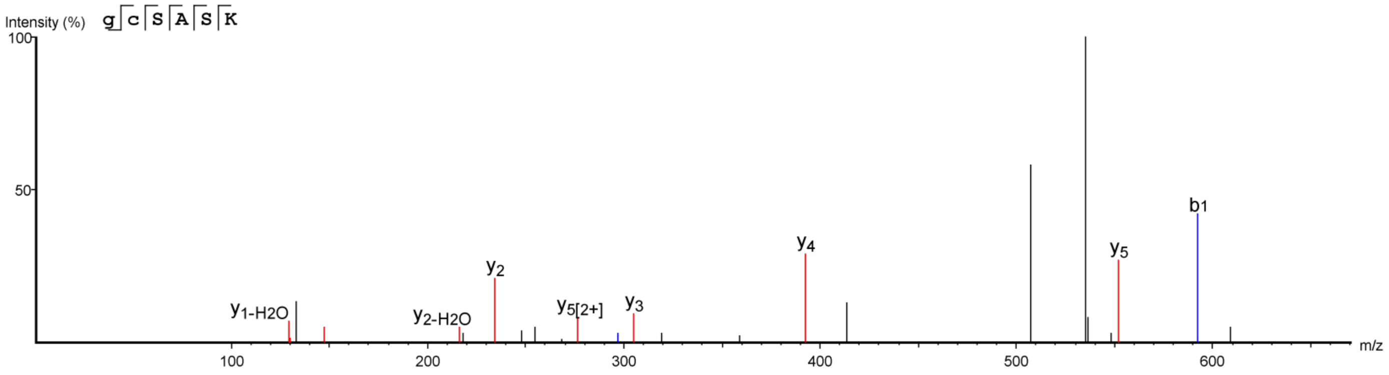

Experiment Inhib. Y_B

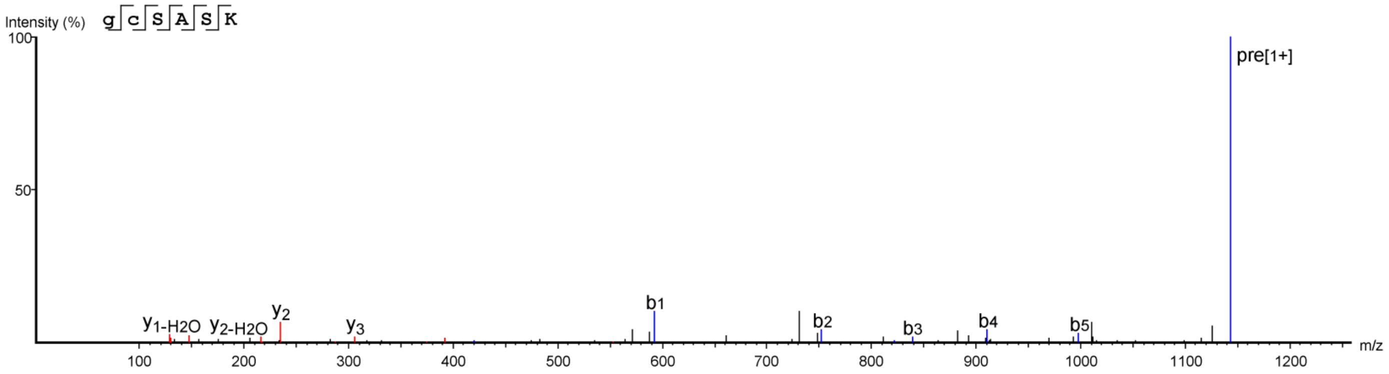

Experiment Inhib. Y_C

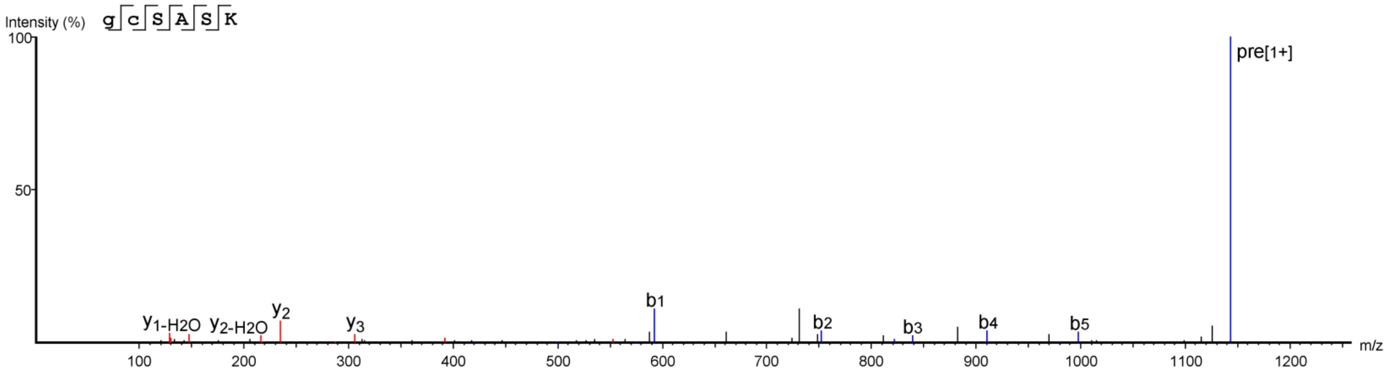

Experiment Inhib. Y_D

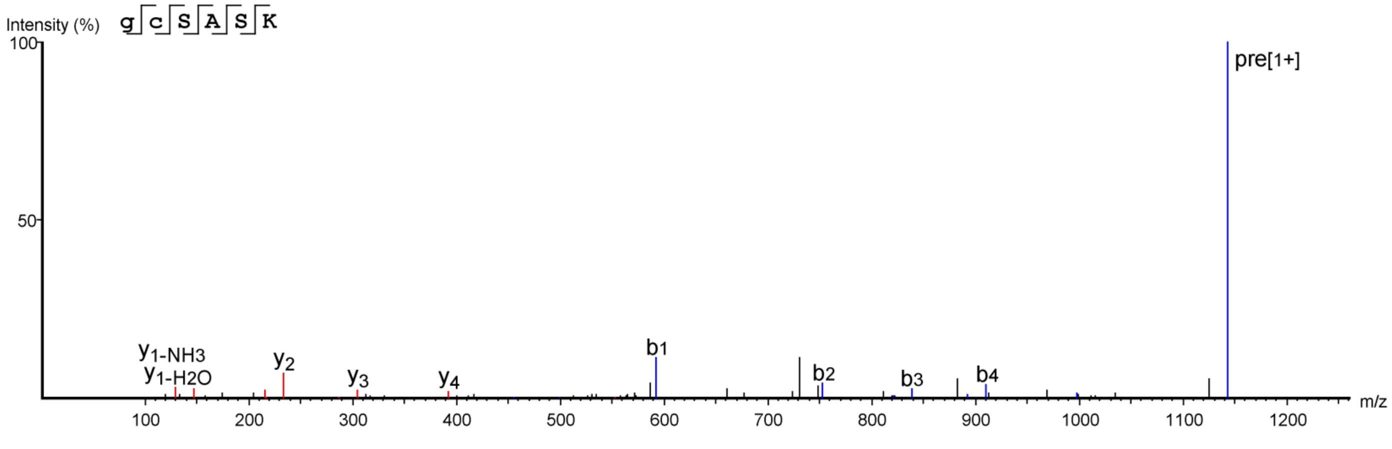

LDBPK_130990.1 (Proteasome regulatory ATPase subunit 2)

Experiment Inhib. Y_A

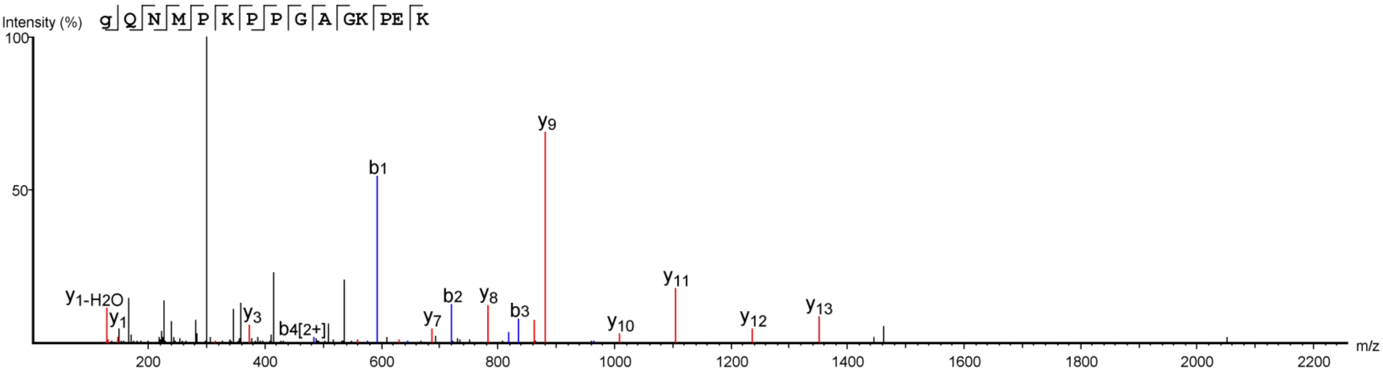

Experiment Inhib. Y_C

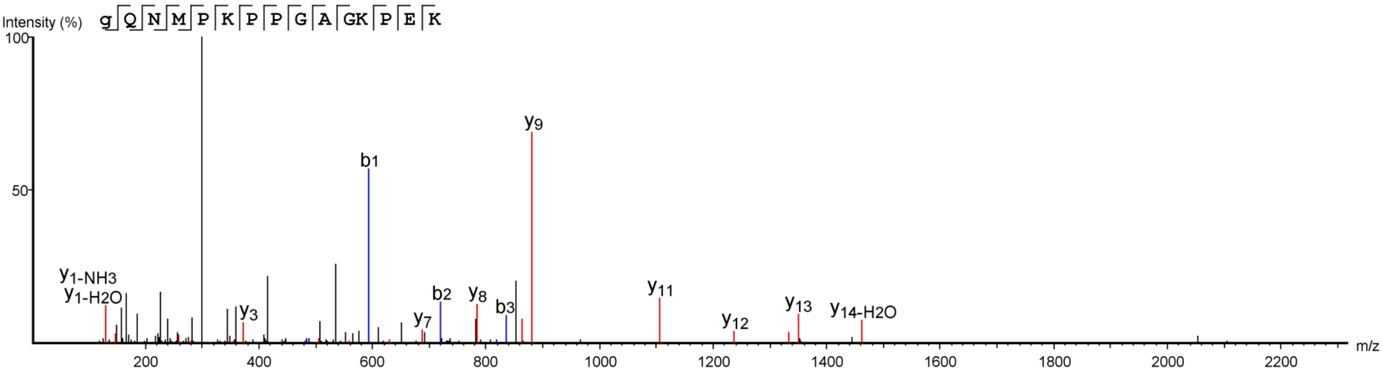

Experiment Inhib. Y_D

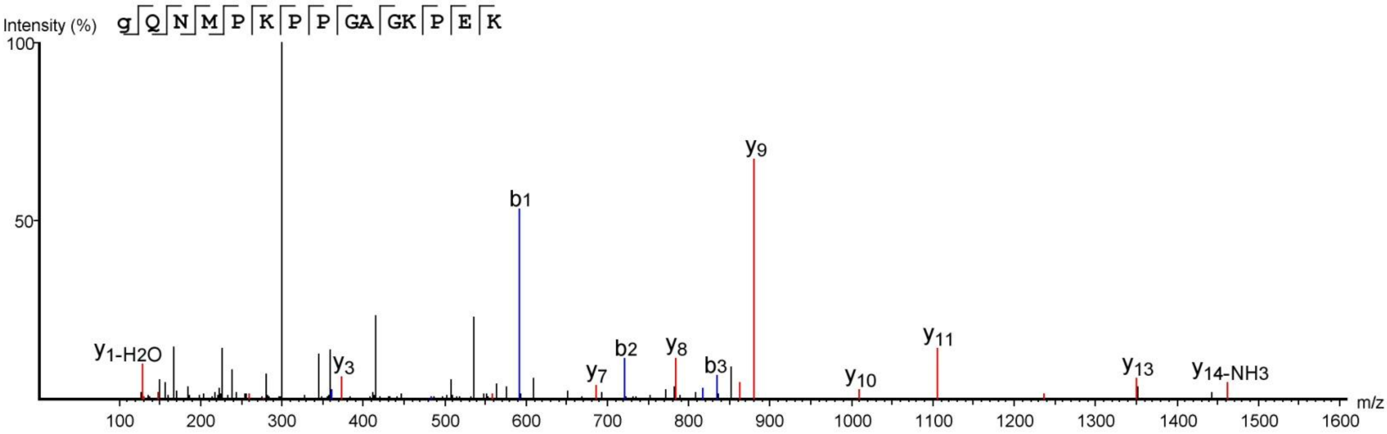

LDBPK_241400.1 (Uncharacterized protein)

Experiment Inhib. Y_A

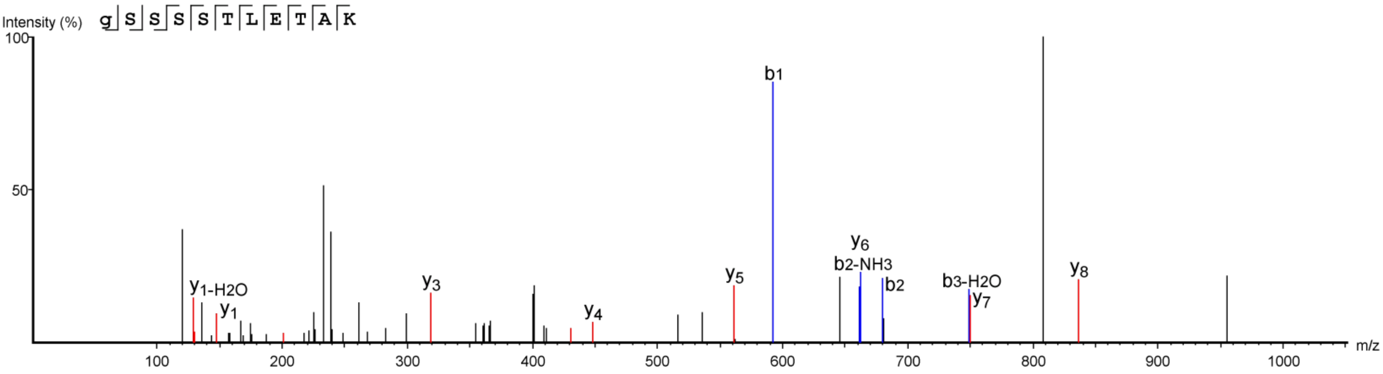

LDBPK_311430.1 (Uncharacterized protein)

Experiment Inhib. Y_C

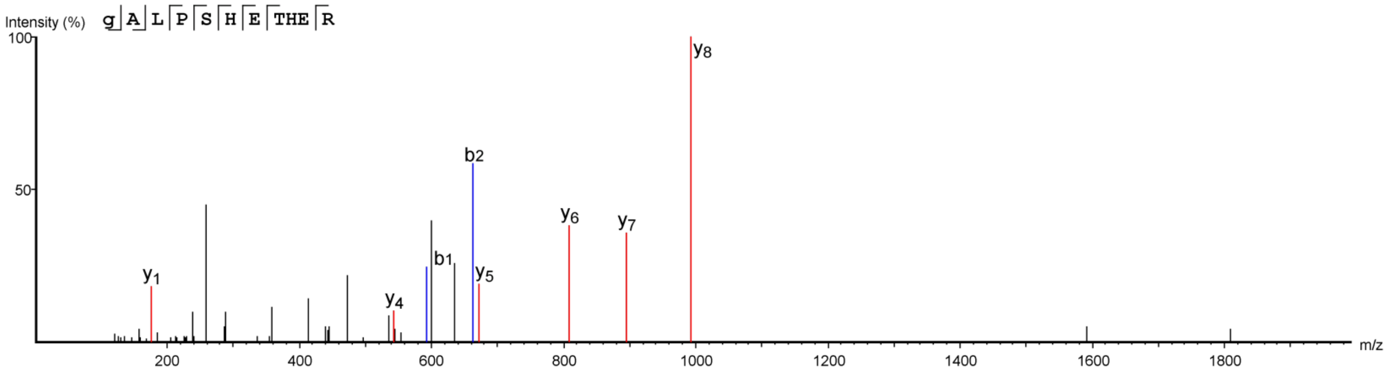

LDBPK_360030.1 (Uncharacterized protein)

Experiment Inhib. Y_C

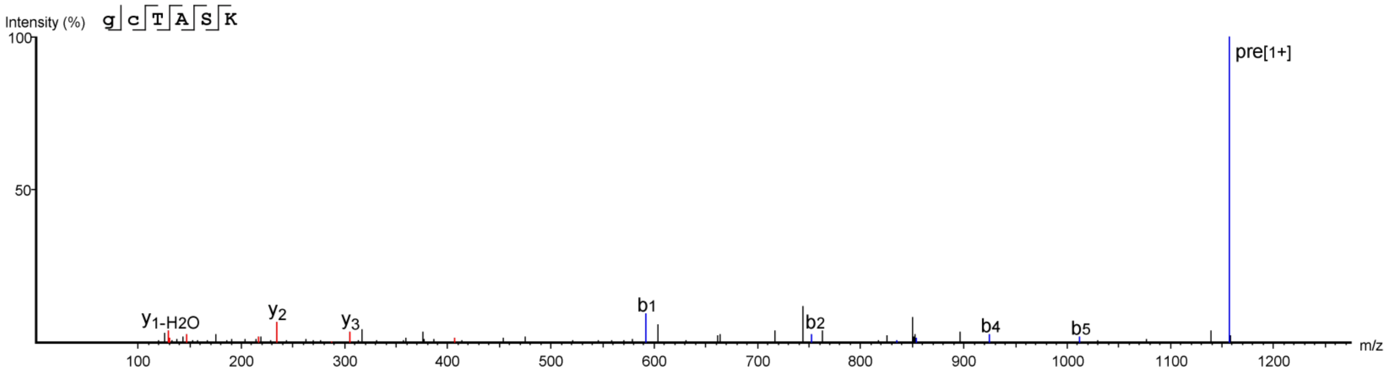

LDBPK_321900.1 (Protein kinase)

Experiment Inhib. Y_C

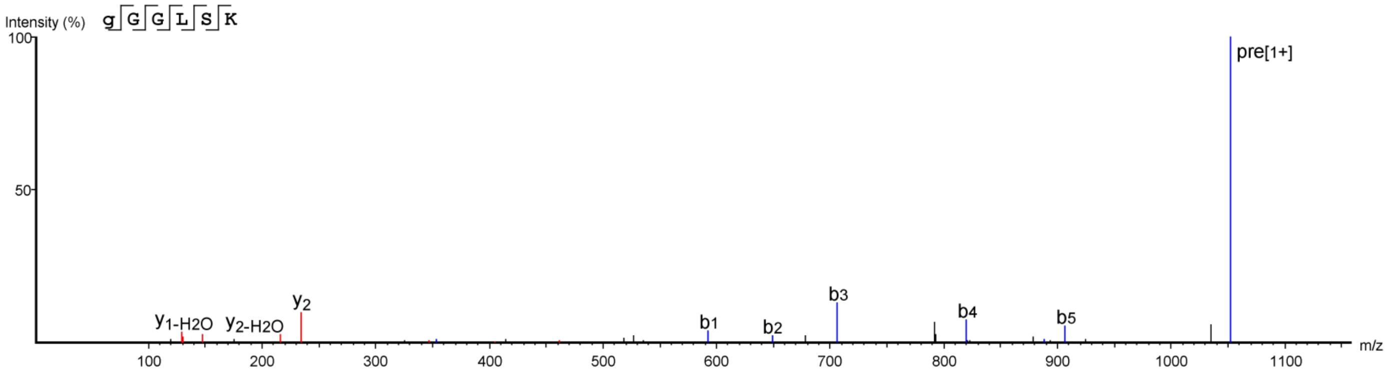

Supplemental Experimental Procedures

Synthesis of AzRB

Capture reagent AzRB was synthesised by solid phase peptide synthesis on a *Biotin-PEG NovaTag™* resin (Novabiochem®). Fmoc-protected amino acids and coupling reagents were sourced from AGTC Bioproducts. Azidoacetic acid was sourced from Sigma Aldrich. Ultrapure water was obtained from a MilliQ Millipore water purification system. LC-MS analysis and purification was performed on a Waters HPLC system fitted with a Waters 515 HPLC pump, Waters 2767 autosampler, Waters XBridge C18 4.6 mm × 100 mm (analytical) or 19mm × 100 mm (preparative) column, Waters 3100 mass spectrometer and Waters 2998 photodiode array. NMR was performed on a Bruker AV-400 instrument. High resolution mass spectrometry was performed by the Imperial College Department of Chemistry Mass Spectrometry Service.

The resin (208 mg, 100 µmol) was placed in a fritted syringe and swollen in DMF for 30 min before Fmoc deprotection in 20% piperidine/DMF (3 mL, 3 x 3 min). The resin was washed in DMF (3 x 3mL), DCM (3 x 3 mL) and DMF (3 x 3mL) after which Fmoc-ε-Ahx-OH (177 mg, 500 µmol), Fmoc-Gly-OH (149 mg, 500 µmol), Fmoc-Arg(Pbf)-OH (324 mg, 500 µmol), Fmoc-Ala-OH (156 mg, 500 µmol) and azidoacetic acid (37 µL, 500 µmol) were double-coupled sequentially. The coupling of Fmoc-ε-Ahx-OH was performed using HATU (186 mg, 490 µmol) and DIPEA (174 µL, 1.0 mmol) and for all other residues HBTU (186 mg, 490 µmol) and DIPEA (174 µL, 1.0 mmol) were used. Each coupling was performed as follows: the amino acid and coupling reagents were dissolved in DMF (2 mL) and activated for 5 min before addition to the resin. The resin was shaken for 1-3 hours, washed in DMF (3 x 3mL), DCM (3 x 3 mL) and DMF (3 x 3 mL). A fresh batch of activated amino acid was added and the resin shaken for a further 1-3 hours. After washing, Fmoc deprotection was performed as above, followed by washing. The procedure was then sequentially repeated for each residue, finishing with the coupling of the azidoacetic acid moiety, which required no Fmoc deprotection. After the final coupling the resin was washed in DMF (3 x 3 mL), DCM (3 x 3 mL), MeOH (3 x 3 mL) and Et_2O (3 x 3 mL) and dried under vacuum overnight. The capture reagent was cleaved from the resin by incubation in a solution of 95% TFA, 2.5% TIS and 2.5% DTT (3 mL) for 3 hours. The cleavage mixture containing the cleaved product was transferred into a 15 mL falcon tube, and the resin washed twice with cleavage mixture (0.5 mL). The solution was concentrated to a volume of 1 mL under a stream of nitrogen, after which the product was precipitated by addition of ice-cold *tert*-butyl methyl ester (TBME) (10 mL). The product was pelleted by centrifugation. The pellet was washed twice with TBME (10 mL) and dried overnight in a vacuum dessicator. The pellet was resuspended in ultrapure water and purified by preparative RP-HPLC in a gradient of methanol and water both supplemented with 0.1% formic acid (0-10 min 5-98% MeOH, 10-12 min 98% MeOH). The fractions containing product were combined and the solvents removed by speedvac. The product was resuspended in ultrapure water (1 mL) and lyophilised to dryness to yield a white solid (24.3 mg, 26 µmol, 26%).

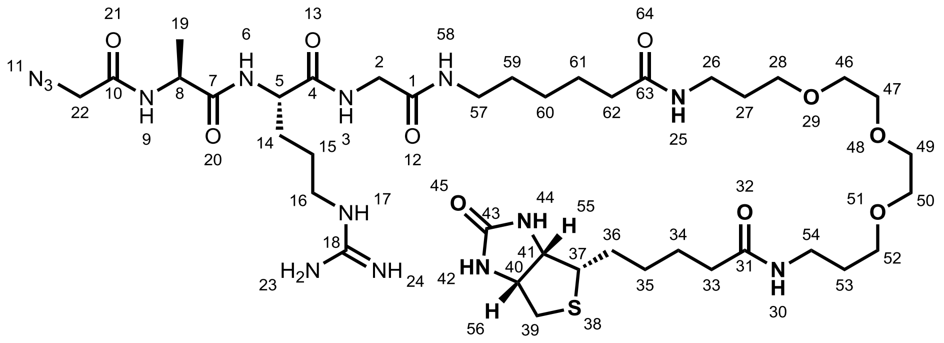

δ_H/ppm (400 MHz, D_2O) 8.39 (s, 1H), 4.58 (dd, *J* = 7.9, 4.8 Hz, 1H, *H56*), 4.40 (dd, *J* = 8.0, 4.4 Hz, 1H, *H55*), 4.36 – 4.23 (m, 2H, *H8* & *H5*), 4.02 (s, 2H, *H22*), 3.93 – 3.77 (m, 2H, *H2*), 3.73-3.59 (m, 8H), 3.55 (2xt, *J* = 6.4, 4H), 3.31 (dt, *J* = 9.7, 5.1 Hz, 1H, *H37*), 3.27 – 3.09 (m, 8H), 2.97 (dd, *J* = 13.1, 5.0 Hz, 1H, ½*H39*), 2.75 (d, *J* = 13.2 Hz, 1H, ½*H39*), 2.22 (2xt, *J* = 7.6 Hz, 4H), 2.03 – 1.17 (m, 23H); δ_C/ppm (100 MHz, CD_3OD) 175.99, 175.94, 175.34, 174.14, 171.21, 170.52, 170.26, 166.09 (6 x C=O, 1 x C=O tautomer), 158.65 (*C17*), 71.53 (2xC), 71.23 (2xC), 69.91 (2xC), 63.39, 61.62, 57.04, 54.53, 52.52, 50.98, 43.39, 41.95, 41.06, 40.30, 37.82 (2xC), 37.02, 36.86, 30.45 (2xC), 29.99, 29.82, 29.59, 29.53, 27.49, 26.92, 26.68, 26.15, 17.66; HRMS, *m/z* (ESI) found 927.5200 ($C_{39}H_{71}N_{14}O_{10}S$, $[M + H]^+$, requires 927.5198).

Parasite culture (extra detail)

Animal experiments were approved by the University of York Animal Procedures and Ethics Committee and performed under UK Home Office licence ('Immunity and Immunopathology of Leishmaniasis' Ref # PPL 60/3708).

The Ethiopian strain of *Leishmania donovani* (MHOM/ET/67/HU3, also known as LV9) was maintained by serial passage in Rag-$2^{-/-}$ mice. Rag-$2^{-/-}$ mice were bred in-house, housed under specific pathogen-free conditions and used at 6–12 weeks of age. Amastigotes were extracted from spleen as described previously (Smelt et al., 1997): briefly, the spleen was removed into unsupplemented RPMI, transferred to a homogeniser and topped up with RPMI medium (approx. 40 mL). The spleen was homogenised to a single cell suspension, transferred to a tube and centrifuged at 800 rpm for 5 minutes; the supernatant was retained. A 50 mL falcon tube was coated with 25 mg saponin per 40 mL supernatant, the supernatant poured in, mixed and left to stand at RT for 5 min. The tube was centrifuged at 3100 rpm for 10 min and supernatant discarded. The pellet was washed 3× by resuspending in 25 mL RPMI and centrifuging at 3100 rpm for 10 minutes. The washed pellet was resuspended in 20 mL RPMI with 10% FCS.

Mice were infected with 3×10^7 *L. donovani* amastigotes intravenously (i.v.) via the tail vein in 200 µl of RPMI 1640 (GIBCO, Paisley, UK).

Promastigotes were obtained by transforming 1×10^7 freshly isolated amastigotes in promastigotes medium (St-Denis et al., 1999) at 26 °C (~2 days for transformation). Parasites were maintained in T25 flasks with non vented lids containing 10 mL medium and passaged twice a week for up to 10 passages.

RPMI medium for *L. donovani* promastigote culture (St-Denis et al., 1999): RPMI 1640 (Sigma #R6748), containing 20 % FCS, 1 % Pen/Strep, 100 μM adenine, 20 mM MES (pH 5.5 with NaOH), 5 μM hemin, 3 μM biopterin and 1 μM biotin.

Metabolic tagging experiments (extra detail)

Promastigotes were pelleted by centrifugation at 800 ×g, 15 min, 26 °C, and resuspended in RPMI/10% FCS containing the appropriate probe (50 μM YnMyr, or myristic acid, 100 μM AHA etc, unless otherwise indicated; from 100 mM stocks) at a parasite density of 7.5×10^7 parasites/mL. Cultures were incubated at 26 °C for 12 hours (or as indicated). Cells were pelleted by centrifugation 800 ×g, 15 min, 4 °C, washed twice with cold PBS then pelleted at 3200 rpm for 10 min at 4°C. After removal of the supernatant, cells were lysed in lysis buffer (1 % NP40, 1% sodium deoxycholate, 0.5 % SDS, 50 mM Tris pH 7.4, 150 mM NaCl, EDTA-free protease inhibitor, Roche) at 1×10^9 parasites/mL by sonication on ice (4× 10 sec burst, amplitude 45 with 1 min interval). Insoluble material was separated by centrifugation at 16,100 ×g for 30 min at 4°C, the supernatant transferred to fresh tubes and snap frozen in liquid N_2, then stored at -80 °C. Tagging of *ex vivo* amastigotes was carried out as above but parasites were cultured at 37 °C.

For inhibition studies, the appropriate inhibitor was pre-incubated with parasite culture for 1 h at 37°C, parasites pelleted by centrifugation at 2200 ×g, 10 min, supernatant removed and parasites resuspended in fresh medium containing inhibitor plus probe (YnMyr, myristic acid etc).

Ex vivo amastigotes inhibition assay

As described in (Hutton et al., 2014). Briefly, *L. donovani* amastigotes (MHOM/ET/67/L28/LV9) isolated from the spleens of infected Rag-$2^{-/-}$ mice were placed in 96 well plates at a density of 4×10^5 *ex vivo* amastigotes per well in a total volume of 200 μL of supplemented RPMI 1640 medium (see ‘promastigote medium’, above) containing test compounds diluted over a threefold range. After incubation for 72 hr at 26 °C, 20 μL of an Alamar blue solution (Trek Diagnostics) was added and plate incubated for a further 6 hr at 26°C. Fluorescence per well was measured using a POLARstar Optima instrument (BMG labtech; ex.: 544 nm, em.: 590 nm). All assays were run in triplicate and the data analysed by a standard package (GraphPad Prism v5).

Macrophage cytotoxicity assay

As described in (Hutton et al., 2014). Briefly, compounds were tested in parallel against mouse primary macrophages (Alamar blue assay) in triplicate, and analysed as above. In brief, primary macrophages derived from BALB/c bone marrow were seeded into 96 well plates at 4.2×10^4 macrophages/well in a total volume of 200 μL (DMEM (Invitrogen, UK), 10% FCS, 4% L-929 cell conditioned supernatant). Compounds diluted over a threefold range were added, with assays run in duplicate. After incubation for 72 hr at 37°C/5% CO_2, 20 μL Alamar blue was added and the plate incubated for a further 6 hr at 37°C/5% CO_2, prior to fluorescence measurements as above.

CuAAC labeling and pull-down

Proteins were precipitated with chloroform/methanol (MeOH:$CHCl_3$:ddH_2O 4:1:3), or acetone (4 vol. -20 °C 1 h) and then resuspended at 1 mg/mL in 1 % SDS in PBS. This precipitation step was found to increase labeling intensity after CuAAC, likely due to the presence of probe-incorporating glycolipids in the lysates (see main text). Premixed click reagents (100 µM AzTB, 1 mM $CuSO_4$, 1 mM TCEP, 100 µM TBTA, final concentrations) were added as described previously (Heal et al., 2012) and samples vortexed for 1 h RT, then quenched by the addition of 10 mM EDTA. Proteins were precipitated again with MeOH/$CHCl_3$, washed with ice-cold MeOH, air-dried for ~15 min, and then resuspended in 2 % SDS, 10 mM EDTA in PBS. For direct gel analysis, 4 x sample loading buffer (NuPAGE LDS sample buffer) with 2-mercaptoethanol (4 % final) was added and proteins heated for 3 min at 95 °C prior to SDS-PAGE.

For hydroxylamine (NH_2OH) and NaOH treatment, samples were resuspended after CuAAC as above, then 1 M NH_2OH pH7 or 0.2M NaOH added and samples incubated at RT for 1 hour. Samples were quenched by addition of 4 x sample loading buffer. Note that addition of EDTA to the samples before NH_2OH treatment is important to avoid sample degradation.

For pull-down, protein was resuspended at 10 mg/mL in 2 % SDS, 10 mM EDTA in PBS, and then diluted to 1 mg/mL with PBS. DTT (from a fresh 100 × stock in water) was added to give a final concentration of 1 mM. Proteins were incubated with Dynabeads® MyOne™ Streptavidin C1 (pre-washed 3 × 0.2 % SDS in PBS) for 1.5-2 h at RT with rotation. Following removal of the supernatant, beads were washed with 3 × 1 % SDS in PBS, then boiled for 10 mins in sample loading buffer elute bound proteins.

Gel and Western blot analysis

Samples were separated by SDS-PAGE and the gel soaked in fixing solution (10 % AcOH, 40 % MeOH), then rinsed in water for in-gel fluorescent imaging: gels were scanned with Cy3 filters to detect the TAMRA fluorophore using an Ettan DIGE scanner, GE Healthcare. Molecular weight markers (Precision Plus All Blue Standards, Bio-Rad) were detected with Cy5 filters.

For Western blot, proteins were transferred from gels to PVDF membrane (Immobilon-P^{SQ}, Millipore) using a semi-dry system (Invitrogen). Following blocking (5 % milk in TBS, 1% Tween), membranes were incubated with primary antibody for 1 hour at room temperature or overnight at 4°C in blocking solution, then incubated with secondary antibody (goat anti-rabbit IgG-HRP, Invitrogen, 1:10000) for 1 hr in blocking solution. Detection was carried out using Luminata Crescendo Western HRP substrate (Millipore) according to the manufactures instructions and on a Fujifilm LAS 3000 imager. Primary antibodies, LdHASPB (rabbit; ab 2-AE) (Alce et al., 1999), LmHASPB (rabbit, 336) (Alce et al., 1999), GP63 (rabbit, polyclonal; provided by R. McMaster, University of British Columbia) (Frommel et al., 1990), GFP (mouse, Santa Cruz), were used at 1:1000-2000.

Proteomics experiments

Pull-down and preparation of peptides for MS. Proteins were captured by CuAAC as before with the following modifications: CuAAC reaction was carried out for 2 hours and, for some samples, with AzRB in place of AzTB. When AzRB was used, proteins were precipitated following CuAAC via a modified chloroform/methanol precipitation procedure: 4 volumes of MeOH, 1 vol. $CHCl_3$, 3 vol. H_2O were added to the sample, which was

centrifuged at 17,000 ×g for 5 min to pellet proteins at the interface. Both layers were then removed simultaneously, the pellet resuspended in 0.2 % SDS/PBS to the original volume and the precipitation procedure repeated. The pellet was then washed twice with MeOH. Resuspension and affinity enrichment were performed for all samples as before with the following modifications: NeutrAvidin agarose resin (Thermo Scientific, pre-washed 3 × 0.2 % SDS in PBS; typically 50 µL of bead slurry was used for 0.5 mg of lysate) was used in place of the magnetic Dynabeads due to increased stability of the resin during on-bead reduction and alkylation of proteins. Beads were stringently washed following pull-down: 3 × 1 % SDS in PBS, 3 × 4M Urea in 50 mM PBS, 5 × AMBIC (50 mM ammonium bicarbonate). For a 50 µL bed of beads resuspended in 50 µL AMBIC, samples were reduced (5 µL of 100 mM DTT in 50 mM AMBIC) at 60 °C for 30 minutes and allowed to cool to room temperature. The beads were washed with 2 x AMBIC. Cysteines were alkylated (5 µL of 100 mM iodoacetamide in AMBIC) at room temperature for 30 min in the dark. The beads were washed with 2 x AMBIC. Trypsin (3 µg Sequencing Grade Modified Trypsin (Promega) dissolved at 0.2 µg/µL in AMBIC per mg starting lysate) was added to the beads and samples were placed on a shaker and digested overnight at 37 °C. The samples were centrifuged and the supernatant was transferred into clean tubes. The beads were washed twice with 0.1% aqueous formic acid, and these washes were combined with the first supernatant. The solutions were stage-tipped according to a published protocol (Rappsilber et al., 2003). Elution from the sorbent (SDC-XC from 3M) with 70 % acetonitrile in water was followed by speed-vac-assisted solvent removal, reconstitution of peptides in 0.5 % TFA, 2 % acetonitrile in water, and transferred into LC-MS sample vials.

LC-MS/MS analysis. The analysis was performed using an Acclaim PepMap RSLC column 50 cm × 75 µm inner diameter (Thermo Fisher Scientific) using a 2 h acetonitrile gradient in 0.1 % aqueous formic acid at a flow rate of 250 nL/min. Easy nLC-1000 was coupled to a Q Exactive mass spectrometer via an easy-spray source (all Thermo Fisher Scientific). The Q Exactive was operated in data-dependent mode with survey scans acquired at a resolution of 75,000 at m/z 200 (transient time 256 ms). Up to 10 of the most abundant isotope patterns with charge +2 or higher from the survey scan were selected with an isolation window of 3.0 m/z and fragmented by HCD with normalized collision energies of 25. The maximum ion injection times for the survey scan and the MS/MS scans (acquired with a resolution of 17 500 at m/z 200) were 20 and 120 ms, respectively. The ion target value for MS was set to 10^6 and for MS/MS to 10^5, and the intensity threshold was set to 8.3×10^2.

Data searching and analysis. The data were processed with MaxQuant version 1.3.0.5, and the peptides were identified from the MS/MS spectra searched against TriTrypDB-6.0 L. donovani LdBPK282A1 database using the Andromeda search engine. The protein HASPB was not present in this database and so the Uniprot sequence (O77300_LEIDO) was appended to the FASTA file. Cysteine carbamidomethylation was used as a fixed modification, and methionine oxidation and N-terminal acetylation as variable modifications. The false discovery rate was set to 0.01 for peptides, proteins and sites. Other parameters were used as pre-set in the software. “Unique and razor peptides” mode was selected to allow for protein grouping; this calculates ratios from unique and razor peptides (razor peptides are uniquely assigned to protein groups and not to individual proteins). Data were elaborated using Microsoft Office Excel 2007 and Perseus versions 1.4.0.20 and 1.5.0.31. Label free quantification (LFQ) experiments in MaxQuant were performed using the built-in label-free quantification algorithm (Cox et al., 2014).

Modified peptide identification. MS data were processed with PEAKS7 suite (Zhang et al., 2012). The data were searched against a reference *L. donovani* database from Uniprot (27/11/2014). Trypsin (specific, up to three missed cleavages allowed) was selected for database searches, and no enzyme was chosen for de novo searches. The maximal mass error was set to 5 ppm for precursor ions and 0.01 Da for product ions. Carbamidomethylation was selected as a fixed modification, and methionine oxidation as well as the lipid-derived adduct (+534.3278 Da) to any amino acid at peptide N-terminus were set as variable modifications. The maximal number of modifications per peptide was set as five. The false discovery rate was set to 0.01 for peptides, and b1 ions were required for N-terminally modified peptides. Within PEAKS, a module called SPIDER searches for point mutations to further enhance the discovery (Ma and Johnson, 2011). All peptides reported are N-terminal peptides unique for the protein that they were assign to by the software.

Data processing: YnMyr tagged proteins in amastigotes and promastigotes. Amastigote experiments: four replicates were performed independently, starting from the same metabolically labelled lysates (Myr and YnMyr samples) through the sample preparation steps and LC-MS/MS analysis. The replicates were grouped together (groups: Myr, YnMyr). YnMyr protein group was filtered to require three valid values across the four replicates. Label free intensities were logarithmized (base 2) and empty values were imputed with random numbers from a normal distribution, whose mean and standard deviation were chosen to simulate low abundance values close to noise level (impute criteria: width 0.1 and down shift 1.8). A modified t-test with permutation based FDR statistics was applied (250 permutations; FDR = 0.05; S0 = 1). Imputing missing values enables assessment of whether a protein is enriched above background noise levels and facilitates statistical analyses, but is likely to generate false negatives where a genuine hit protein is of low abundance in the sample. To explore this possibility empty values were reinstated and data analysed to assess whether a protein was absent from the control but still met the other criteria (present in three out of four amastigote replicates). Proteins absent from the control sample but not reaching significance were designated ‘low abundance hits’. Proteins reaching significance in the t-test and absent from the control sample were designated as ‘high confidence hits’.

Promastigote experiments: three replicates were performed independently, starting from the same metabolically labelled lysates (Myr and YnMyr samples) through the sample preparation steps and LC-MS/MS analysis. Data were analysed as described above with 3 valid values per group required, imputation (width 0.1 and down shift 1.8), and t-test (250 permutations; FDR = 0.01; S0 = 1).

For the comparison of amastigotes and promastigotes, data were filtered for at least three valid values in at least one group (groups: Am_Myr, Am_YnMyr, Pro_Myr, Pro_YnMyr). Missing values were imputed as above and proteins cross-referenced with the independent analyses described above that defined t-test significant, low abundance and high confidence hits. Datasets were also compared by a two sample t-test: YnMyr intensities of hits (defined as proteins that were either t-test significant or classed as low abundance hits in the independent analyses) were compared by permutation-corrected t-test (250 permutations; FDR 0.05, S0 2) after imputation of missing values (width 0.1, downshift 2.5). These parameters were chosen so that proteins only found in one of the two datasets were significant in the t-test.

Data processing: YnMyr tagging in the presence of NMT inhibitors (amastigotes). The experiment design was as follows: independent biological experiments were carried out where amastigotes were incubated with YnMyr, YnMyr + 0.2 μM **2**, YnMyr + 0.2 μM **1**, or YnMyr + 7 μM **1**. A Myr control was included in one of these experiments. For proteomics analysis, all samples were prepared and processed in duplicate (technical replicates: A&B; C&D). The 'Match between runs' option (time window 2 minutes) in Maxquant was enabled during the searches. Data were grouped, filtered to retain only those proteins present in both biological experiments, and non-specific binders removed (leaving 346 proteins); for the latter step, enrichment over control ('Myr LFQ ratio'), calculated by taking the mean of the YnMyr intensities and dividing by the mean of the Myr intensities, was required to be ≥2. Missing values were imputed from a normal distribution (width 0.1 and down shift 1.8) and modified two sample t-tests with permutation based FDR statistics were applied to assess differences between groups (250 permutations; FDR = 0.001; S0 = 1, unless otherwise indicated). An ANOVA (Benjamini Hochberg correction, FDR=0.001) was also applied to LFQ intensities to compare across all datasets.

Bioinformatics

Modification of proteins was predicted using the following bioinformatic tools:

For *N*-myristoylation: the Myr predictor (MPred) (Maurer-Stroh et al., 2002) and the Myristoylator (Myr) (Bologna et al., 2004).

For *S*-palmitoylation CSS-Palm 3.0 (Ren et al., 2008; Zhou et al., 2006).

For GPI-anchor modification PredGPI (Pierleoni et al., 2008), GPI-SOM (Fankhauser and Maser, 2005) and FragAnchor (Poisson et al., 2007).

Supplemental References

Alce, T.M., Gokool, S., McGhie, D., Stager, S., and Smith, D.F. (1999). Expression of hydrophilic surface proteins in infective stages of Leishmania donovani. Mol. Biochem. Parasitol. *102*, 191-196.

Bologna, G., Yvon, C., Duvaud, S., and Veuthey, A.L. (2004). N-Terminal myristoylation predictions by ensembles of neural networks. Proteomics *4*, 1626-1632.

Cox, J., Hein, M.Y., Luber, C.A., Paron, I., Nagaraj, N., and Mann, M. (2014). Accurate Proteome-wide Label-free Quantification by Delayed Normalization and Maximal Peptide Ratio Extraction, Termed MaxLFQ. Mol. Cell. Proteomics *13*, 2513-2526.

Fankhauser, N., and Maser, P. (2005). Identification of GPI anchor attachment signals by a Kohonen self-organizing map. Bioinformatics *21*, 1846-1852.

Frommel, T.O., Button, L.L., Fujikura, Y., and McMaster, W.R. (1990). The major surface glycoprotein (GP63) is present in both life stages of Leishmania. Mol. Biochem. Parasitol. *38*, 25-32.

Heal, W.P., Wright, M.H., Thinon, E., and Tate, E.W. (2012). Multifunctional protein labeling via enzymatic N-terminal tagging and elaboration by click chemistry. Nat. Protoc. *7*, 105-117.

Hutton, J.A., Goncalves, V., Brannigan, J.A., Paape, D., Wright, M.H., Waugh, T.M., Roberts, S.M., Bell, A.S., Wilkinson, A.J., Smith, D.F.*, et al.* (2014). Structure-Based Design of Potent and Selective Leishmania N-Myristoyltransferase Inhibitors. J. Med. Chem.

Ma, B., and Johnson, R. (2011). De novo sequencing and homology searching. Mol. Cell. Proteomics *11*, O111 014902.

Maurer-Stroh, S., Eisenhaber, B., and Eisenhaber, F. (2002). N-terminal N-myristoylation of proteins: prediction of substrate proteins from amino acid sequence. J. Mol. Biol. *317*, 541-557.

Pierleoni, A., Martelli, P.L., and Casadio, R. (2008). PredGPI: a GPI-anchor predictor. BMC Bioinformatics *9*, 392.

Poisson, G., Chauve, C., Chen, X., and Bergeron, A. (2007). FragAnchor: a large-scale predictor of glycosylphosphatidylinositol anchors in eukaryote protein sequences by qualitative scoring. Genomics Proteomics Bioinformatics *5*, 121-130.

Rappsilber, J., Ishihama, Y., and Mann, M. (2003). Stop and go extraction tips for matrix-assisted laser desorption/ionization, nanoelectrospray, and LC/MS sample pretreatment in proteomics. Anal. Chem. *75*, 663-670.

Ren, J., Wen, L., Gao, X., Jin, C., Xue, Y., and Yao, X. (2008). CSS-Palm 2.0: an updated software for palmitoylation sites prediction. Protein Eng Des Sel *21*, 639-644.

Smelt, S.C., Engwerda, C.R., McCrossen, M., and Kaye, P.M. (1997). Destruction of follicular dendritic cells during chronic visceral leishmaniasis. J. Immunol. *158*, 3813-3821.

St-Denis, A., Caouras, V., Gervais, F., and Descoteaux, A. (1999). Role of protein kinase C-alpha in the control of infection by intracellular pathogens in macrophages. J. Immunol. *163*, 5505-5511.

Zhang, J., Xin, L., Shan, B., Chen, W., Xie, M., Yuen, D., Zhang, W., Zhang, Z., Lajoie, G.A., and Ma, B. (2012). PEAKS DB: de novo sequencing assisted database search for sensitive and accurate peptide identification. Mol. Cell. Proteomics *11*, M111 010587.

Zhou, F., Xue, Y., Yao, X., and Xu, Y. (2006). CSS-Palm: palmitoylation site prediction with a clustering and scoring strategy (CSS). Bioinformatics *22*, 894-896.
