# Supplementary material for: Global Analysis of Protein N-Myristoylation and Exploration of N-Myristoyltransferase as a Drug Target in the Neglected Human Pathogen Leishmania donovani
Source: Chem Biol. 2015 Mar 19;22(3):342–54. doi: 10.1016/j.chembiol.2015.01.003 (PMC4372256; doi:10.1016/j.chembiol.2015.01.003)
Supplement: Document S2. Article plus Supplemental Information [file mmc8.pdf]

Article

Chemistry & Biology

Global Analysis of Protein *N*-Myristoylation and Exploration of *N*-Myristoyltransferase as a Drug Target in the Neglected Human Pathogen *Leishmania donovani*

Graphical Abstract

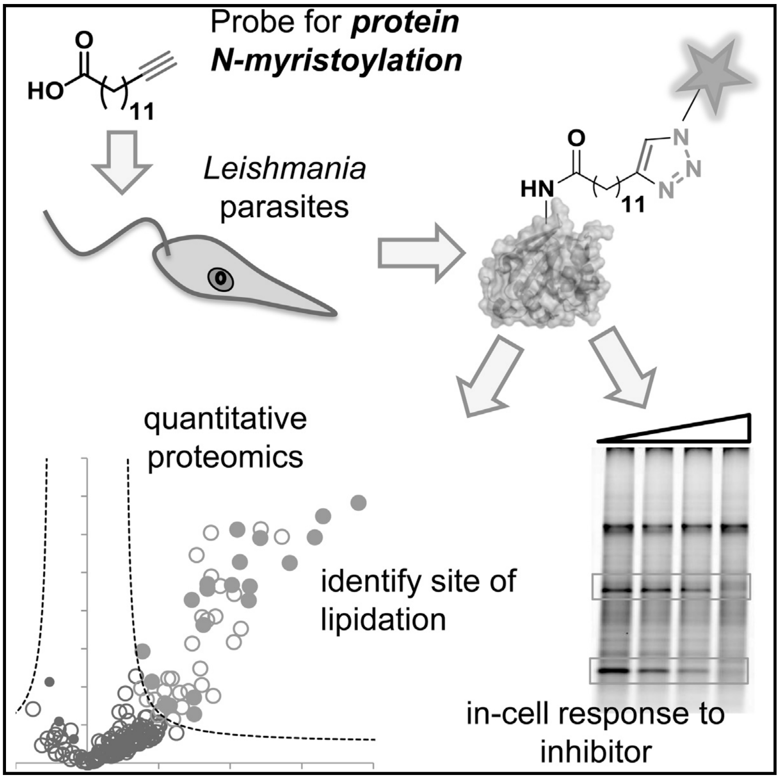

Highlights

- Alkyne-tagged probes for protein lipidation are applied in *Leishmania donovani*
- A global profile of protein lipidation in two life stages is presented
- *N*-Myristoylated proteome defined by chemical knockdown and quantitative proteomics
- Evidence for NMT as a drug target in the leishmaniases

Authors

Megan H. Wright, Daniel Paape, ..., Deborah F. Smith, Edward W. Tate

Correspondence

megan.wright@tum.de (M.H.W.), e.tate@imperial.ac.uk (E.W.T.)

In Brief

Wright et al. use metabolic tagging with an alkyne-myristate analog, click chemistry, and proteomics to identify lipidated proteins in two life stages of the parasite *Leishmania donovani*. Quantitative profiling of *N*-myristoyltransferase inhibition and identification of lipidation sites define the *N*-myristoylated proteome in this human pathogen.

Wright et al., 2015, Chemistry & Biology *22*, 342–354
March 19, 2015 ©2015 The Authors
http://dx.doi.org/10.1016/j.chembiol.2015.01.003

CellPress

Global Analysis of Protein *N*-Myristoylation and Exploration of *N*-Myristoyltransferase as a Drug Target in the Neglected Human Pathogen *Leishmania donovani*

Megan H. Wright,[1,3,*] Daniel Paape,[2,4] Elisabeth M. Storck,[1] Remigiusz A. Serwa,[1] Deborah F. Smith,[2] and Edward W. Tate[1,*]
[1]Department of Chemistry, Imperial College London, London SW7 2AZ, UK
[2]Centre for Immunology and Infection, Department of Biology, University of York, York YO10 5DD, UK
[3]Present address: Department of Chemistry, Technische Universität München, Garching 85478, Germany
[4]Present address: Wellcome Trust Centre for Molecular Parasitology, Institute of Infection, Immunity and Inflammation, University of Glasgow, Glasgow G12 8TA, UK
*Correspondence: megan.wright@tum.de (M.H.W.), e.tate@imperial.ac.uk (E.W.T.)
http://dx.doi.org/10.1016/j.chembiol.2015.01.003
This is an open access article under the CC BY license (http://creativecommons.org/licenses/by/4.0/).

SUMMARY

***N*-Myristoyltransferase (NMT) modulates protein function through the attachment of the lipid myristate to the N terminus of target proteins, and is a promising drug target in eukaryotic parasites such as *Leishmania donovani*. Only a small number of NMT substrates have been characterized in Leishmania, and a global picture of *N*-myristoylation is lacking. Here, we use metabolic tagging with an alkyne-functionalized myristic acid mimetic in live parasites followed by downstream click chemistry and analysis to identify lipidated proteins in both the promastigote (extracellular) and amastigote (intracellular) life stages. Quantitative chemical proteomics is used to profile target engagement by NMT inhibitors, and to define the complement of *N*-myristoylated proteins. Our results provide new insight into the multiple pathways modulated by NMT and the pleiotropic effects of NMT inhibition. This work constitutes the first global experimental analysis of protein lipidation in *Leishmania*, and reveals the extent of NMT-related biology yet to be explored for this neglected human pathogen.**

INTRODUCTION

Over 300 million people are at risk from the leishmaniases, a spectrum of neglected tropical parasitic diseases strongly associated with poverty, which cause around 30,000 deaths annually (Alvar et al., 2012). The diseases take two main forms: cutaneous leishmaniasis (CL), manifesting as skin lesions, and visceral leishmaniasis (VL), also known as kala-azar, a systemic infection causing swelling of organs, fever, and anemia, that is almost invariably fatal if left untreated. The causative agents of these debilitating diseases are species of the *Leishmania* protozoan parasite, a unicellular eukaryote. *Leishmania donovani* (Ld) is the principle causative agent of VL while *Leishmania major* (Lm) is the main species responsible for CL. The parasite is transmitted by sand fly vectors, which inoculate the promastigote forms into the human host. Here, they are taken up by phagocytic cells, mainly macrophages, and transform into the obligate intracellular amastigotes, which then replicate and can infect other phagocytes. Transmission occurs when a sand fly takes up infected cells in a blood meal. Inside the sand fly midgut, amastigotes transform into promastigotes. There are currently few drugs to treat the leishmaniases, and those that are available have problems of toxicity, teratogenicity, high cost, difficult administration, and/or parasite resistance. These drugs work via nonspecific antibiotic effects, such as disruption of cell membranes, or their mode of action is unknown (Tate et al., 2014). Novel drugs with a defined mechanism of action, high efficacy and low toxicity are urgently required to combat these devastating diseases.

The enzyme myristoyl-CoA:protein *N*-myristoyltransferase (NMT) has recently been validated as a promising drug target in trypanosomiasis (Frearson et al., 2010) and malaria (Wright et al., 2014), diseases caused by the protozoan parasites *Trypanosoma brucei* and species of *Plasmodium*, respectively. NMT has also been proposed as a potential drug target for infections caused by parasitic nematodes (Galvin et al., 2014) and for the leishmaniases (Price et al., 2003). NMT catalyzes a protein modification, the attachment of the C14:0 lipid myristate (Myr, tetradecanoic acid, Figure 1A) to the N-terminal glycine residue of specific target proteins in the cell. This reaction usually occurs cotranslationally during ribosomal synthesis of the protein, affording a stable, amide-linked lipid that modulates protein function, typically promoting protein localization to cellular membranes (Wright et al., 2010). NMT has been characterized in Ld and Lm and appears to be essential for survival of promastigotes in both organisms (Brannigan et al., 2010; Price et al., 2003), although evidence for the importance of NMT in amastigotes has yet to be reported. Structure-based design and high-throughput screening has led to the discovery of several distinct NMT inhibitor series (Bell et al., 2012; Brannigan et al., 2014; Hutton et al., 2014; Olaleye et al., 2014). Around 60 *Leishmania* proteins have been predicted to be *N*-myristoylated using bioinformatic approaches based on learning sets from unrelated organisms (Mills et al., 2007), but experimental evidence has only

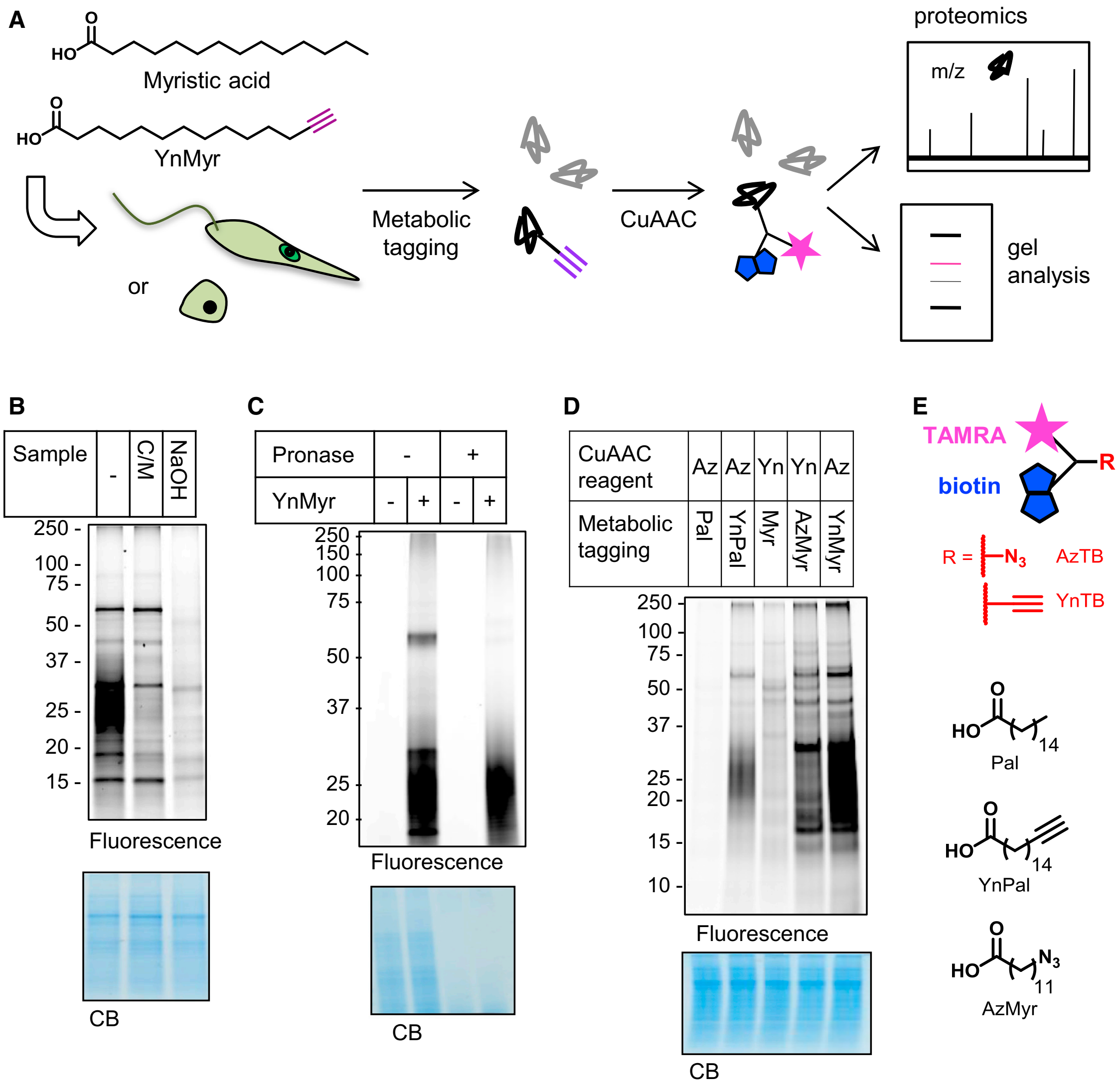

Figure 1. Tagging of Proteins by YnMyr in *Leishmania*

(A) Overview of metabolic tagging of proteins with myristic acid analog YnMyr and analysis following CuAAC labeling.

(B) YnMyr tagging in *L. donovani* promastigotes. Following CuAAC labeling with reagent AzTB, samples were precipitated with $CHCl_3$/MeOH (CM) or with MeOH and then treated with NaOH as indicated. CB, Coomassie blue.

(C) The effect of pronase digestion on tagging. Pronase digests protein resulting in a loss of the discrete fluorescent bands but leaving the diffuse labeling relatively unaffected.

(D) YnMyr tagging compared with other analogs YnPal and AzMyr in promastigotes. Pal, palmitic acid; Myr, myristic acid. CuAAC capture reagent: Az, AzTB; Yn, YnTB.

(E) Chemical tools. Additional labeling data are shown in Figure S1.

been reported for six; these include ADP-ribosylation factor-like protein LdARL1 (Sahin et al., 2008), LdARL3A (Cuvillier et al., 2000), and several proteins that have been shown to carry a dual *N*-myristoylation and *S*-palmitoylation acylation motif. This latter modification involves the incorporation of hexadecanoic acid or other long chain fatty acids via a thioester linkage at a cysteine side chain, sometimes near the N terminus close to an *N*-myristoylation site. An example is dually acylated protein HASPB, a member of a family of hydrophilic acylated surface proteins important for parasite development in the insect vector (Sadlova et al., 2010). HASPB is unusual for an *N*-myristoylated protein in being localized to the outer leaflet of the plasma membrane in infective stages (Denny et al., 2000; Maclean et al., 2012). Similarly, SMP-1, one of several small myristoylated proteins, is flagellum-targeted by acylation (Tull et al., 2004). The SMPs are targeted to different subcellular compartments depending on N-terminal acylation and other sequence motifs (Tull et al., 2012).

Global profiling of protein lipidation in living cells by standard biochemical methods is challenging. We and others have made use of bioorthogonal labeling chemistries such as the copper-catalyzed cycloaddition of an alkyne and azide (CuAAC) to capture proteins metabolically tagged with alkyne or azide fatty

acids (Grammel and Hang, 2013). In this approach, fatty acid analogs bearing the biologically inert azide or alkyne functional group are incubated with cells in culture and incorporated by cellular machinery into lipidated proteins. Following cell lysis, proteins can be functionalized via CuAAC with useful groups such as a fluorophore for visualization by in-gel fluorescence and a biotin affinity handle for enrichment, enabling subsequent mass spectrometry analysis and identification of modified proteins. Here, we use an alkyne-tagged myristate analog, YnMyr (tetradecynoic acid, Figure 1A), which has been previously used as a mimic for myristic acid in several systems, including mammalian cells (Thinon et al., 2014) and *Plasmodium falciparum* (Wright et al., 2014), to profile the lipidome of *L. donovani*. We further combine this chemical proteomic tool with small molecule NMT inhibitors to confirm target engagement inside live parasites, and to define the complement of NMT substrates.

RESULTS

YnMyr Tags Proteins in *L. donovani*

We first sought to demonstrate that YnMyr can tag proteins in *Leishmania*, since this has not previously been reported. Metabolic tagging of Ld promastigotes with 50 μM YnMyr followed by CuAAC with biotin and fluorophore-functionalized reagent AzTB (Figures 1 and S1) gave several distinct fluorescent bands, and a diffuse smear between 18–30 kDa (Figure 1B). This diffuse labeling was effectively removed by $CHCl_3$/MeOH precipitation or treatment with strong base and was resistant to pronase digestion (Figures 1B and 1C), suggesting YnMyr incorporation into a nonprotein component. *Leishmania* species synthesize complex glycolipid macromolecules, including glycosylphosphatidylinositol (GPI)-anchored proteins, glycoinositol phospholipids (GIPLs), and the complex glycoconjugate lipophosphoglycan (LPG) (Ferguson, 1997). Since these molecules contain acyl-fatty acids or ether-linked alkyl chains, YnMyr incorporation into such molecules is not unexpected; indeed, myristate is known to be incorporated into GIPLs and the GPI anchors of some proteins (Etges et al., 1986; Ralton et al., 2002). In addition to removal of the diffuse band, treatment with base in promastigotes resulted in loss of several other bands (Figure 1B). A well-studied protein in *Leishmania* is the surface protease GP63, which incorporates myristic acid into its GPI anchor (Etges et al., 1986). Consistent with this, GP63 could be detected after pull-down of YnMyr tagged promastigote lysate onto streptavidin beads (Figure S1). Hydroxylamine treatment of lysates gave a slight reduction in signal intensity of several weak bands, suggesting that YnMyr may also be incorporated into some *S*-acylation sites (Figure S1).

Labeling in promastigotes was time dependent, outcompeted by myristic acid and inhibited in the presence of the protein synthesis inhibitor cycloheximide (Figure S1), showing that YnMyr incorporation is dependent on de novo protein synthesis. Interestingly, although azido-myristate AzMyr produced a similar, but not identical, labeling pattern, this analog did not label the diffuse bands (Figure 1D), suggesting that the biosynthetic pathways incorporating the analog into these molecules may be less tolerant to modifications in the lipid structure. The background labeling generated by CuAAC with YnTB, the alkyne version of the AzTB capture reagent, was also higher (Figure 1D), as has been noted previously by others (Charron et al., 2009). These data suggest that YnMyr is the more suitable probe for labeling in *Leishmania*. Longer chain palmitic acid analog YnPal showed a distinct labeling pattern to the myristate analogs (Figure 1D), indicating that fatty acyl chain length is important for protein lipidation in these cells.

Metabolic tagging with YnMyr in the amastigote stage gave a distinct labeling pattern compared with promastigotes and no diffuse bands (Figure 2A). While GIPLs are expressed in both life cycle stages, LPG and GPI-anchored proteins are downregulated in the amastigote stage (McConville and Blackwell, 1991), suggesting that the diffuse labeling observed in promastigotes is related to specific glycolipid biosynthetic pathways. Known *N*-myristoylated protein HASPB (Alce et al., 1999) could be detected by western blot after pull-down of tagged proteins onto streptavidin beads in both life stages (Figure 2A), although the overall incorporation of YnMyr into HASPB was low, as may be expected given that the tagged analog competes for substrates with endogenous lipid. To confirm the nature of HASPB labeling, YnMyr tagging was performed in *L. major* strains expressing a fusion protein consisting of GFP fused to either the N-terminal 18 residues of HASPB (wild-type [WT] 18AA-HASPB-GFP), or to a mutant in which the N-terminal glycine was mutated to alanine (G2A) (Denny et al., 2000). The global labeling pattern was similar between these strains and comparable with that in *L. donovani* (Figure S1). Following enrichment of YnMyr tagged proteins, GFP was detected in the WT sample but not in the G2A mutant, consistent with canonical attachment of YnMyr to the N-terminal glycine by NMT; endogenous HASPB was enriched from both WT and G2A mutants (Figure 2B).

The freshly harvested ex vivo amastigotes used in the current study are a practical model system for studying the host infective stage of *Leishmania*, but methods to study this intracellular parasite in its native environment inside mammalian macrophages would be particularly useful for future host-parasite studies. To establish whether YnMyr can penetrate the amastigote inside the parasitophorous vacuole, macrophages infected with Ld amastigotes were incubated with YnMyr for 18 hr, lysed, and samples analyzed by gel; uninfected macrophages were used as a control. Strong labeling of macrophage proteins dominated in-gel fluorescence of both infected and uninfected samples, as might be expected from the low multiplicity of infection (~10) of this intracellular model (Figure 2C). HASPB was immunoprecipitated (IP) from lysates and incubated with CuAAC reagents on-bead, followed by protein elution and analysis by western blot. HASPB was present only in infected samples, as expected, and a fluorescent band was also visible specifically in YnMyr tagged samples at the same molecular weight (Figure 2D). This IP-click approach, which also allows for detection of tagged HASPB in ex vivo amastigote samples (Figure 2E), circumvents the challenges posed by the relatively low abundance of parasite relative to host cell material. These data show that YnMyr also tags the *N*-myristoylated protein HASPB in the context of *Leishmania*-infected macrophages.

Global Proteomic Identification of YnMyr Tagged Proteins in Promastigotes and Amastigotes

Validating the tagging of specific proteins by western blot, as shown above for HASPB, is a low-throughput method and

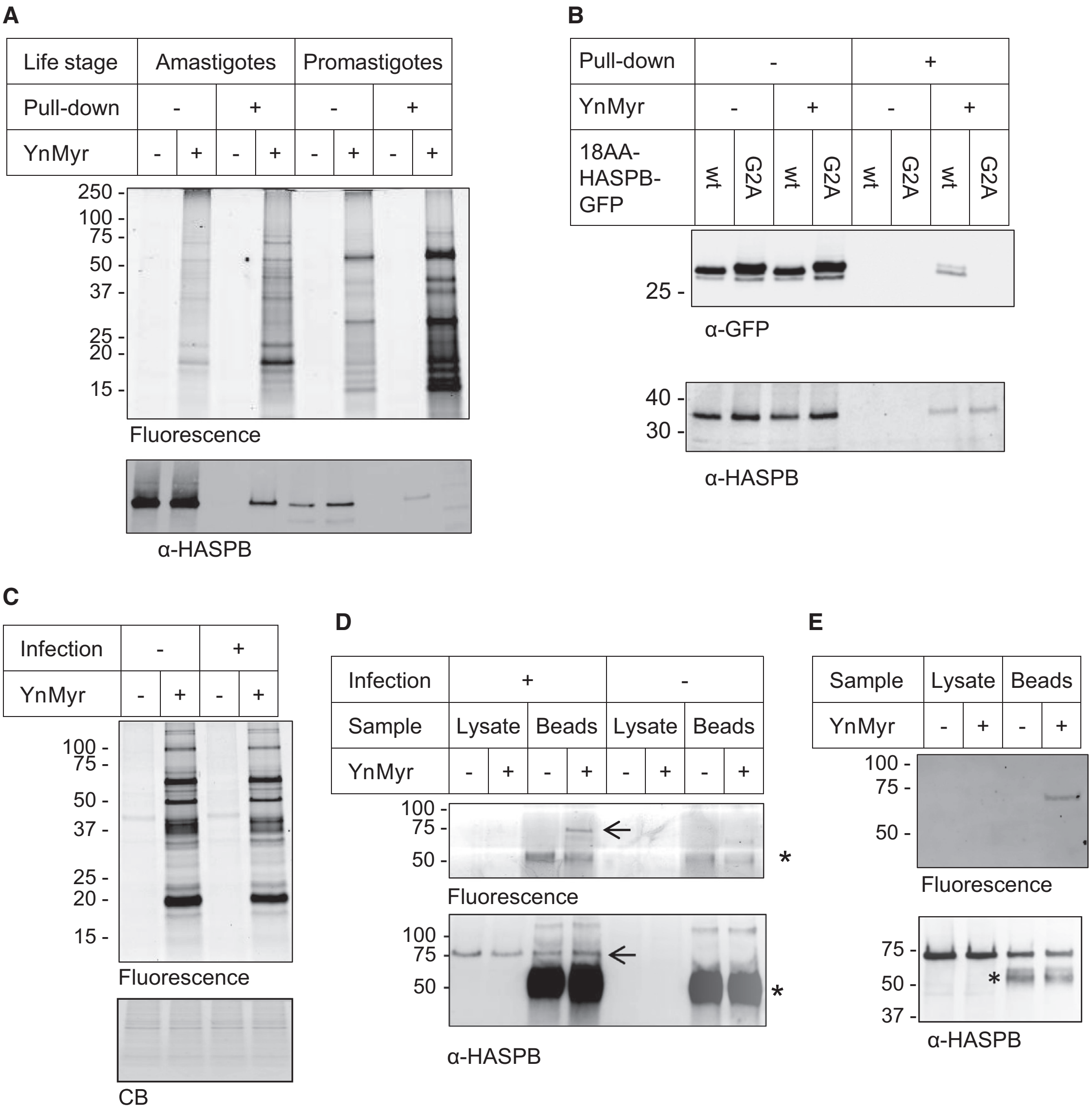

Figure 2. YnMyr Tags Known *N*-Myristoylated Protein HASPB

(A) HASPB is labeled in promastigotes and amastigotes. Parasites were tagged with myristic acid or YnMyr, proteins labeled via CuAAC with AzTB, and a portion enriched by pull-down (PD) onto streptavidin beads. Four-fold more sample loaded after PD compared with before. Western blot (WB) revealed that HASPB is specifically enriched in the YnMyr samples.

(B) HASPB is labeled on the N-terminal glycine. *L. major* parasites expressing the HASPB N terminus (18 residues; WT) fused to GFP or a G2A mutant were tagged with YnMyr and processed as above. Top: WB for GFP shows that only the WT protein and not the G2A mutant is tagged. Bottom: endogenous HASPB is tagged in both samples.

(C) In-gel fluorescence analysis of YnMyr tagged proteins from macrophages infected with *L. donovani* or cultured alone.

(D) HASPB is tagged in intracellular amastigotes. HASPB was immunoprecipitated (IP) from lysates derived from infected or uninfected macrophages tagged with YnMyr, and beads incubated with CuAAC reagents. In-gel fluorescence (top) shows a YnMyr- and infection-specific band at the expected migration for HASPB (arrow). WB (bottom) shows that HASPB (arrow) is present only in infected samples. * indicates antibody IgG heavy chain.

(E) HASPB IP and CuAAC using ex vivo amastigotes.

dependent on the availability of an antibody for the protein of interest. To carry out the global identification of YnMyr tagged proteins, Neutravidin-enriched proteins were digested on-bead with trypsin and peptides analyzed via liquid chromatography-tandem mass spectrometry (LC-MS/MS). For the MS experiments, a capture reagent (AzRB, Supplemental Experimental Procedures) containing a trypsin-cleavable motif between the azide and biotin groups was designed and used. We have previously used similar reagents to facilitate release of YnMyr-modified peptides from Neutravidin beads for identification (Thinon et al., 2014). A label-free quantification (LFQ) approach (Cox et al., 2014) was used to assess enrichment over myristic acid controls. The nature of an enrichment experiment means that hits are often not detected in controls, and analysis of the data

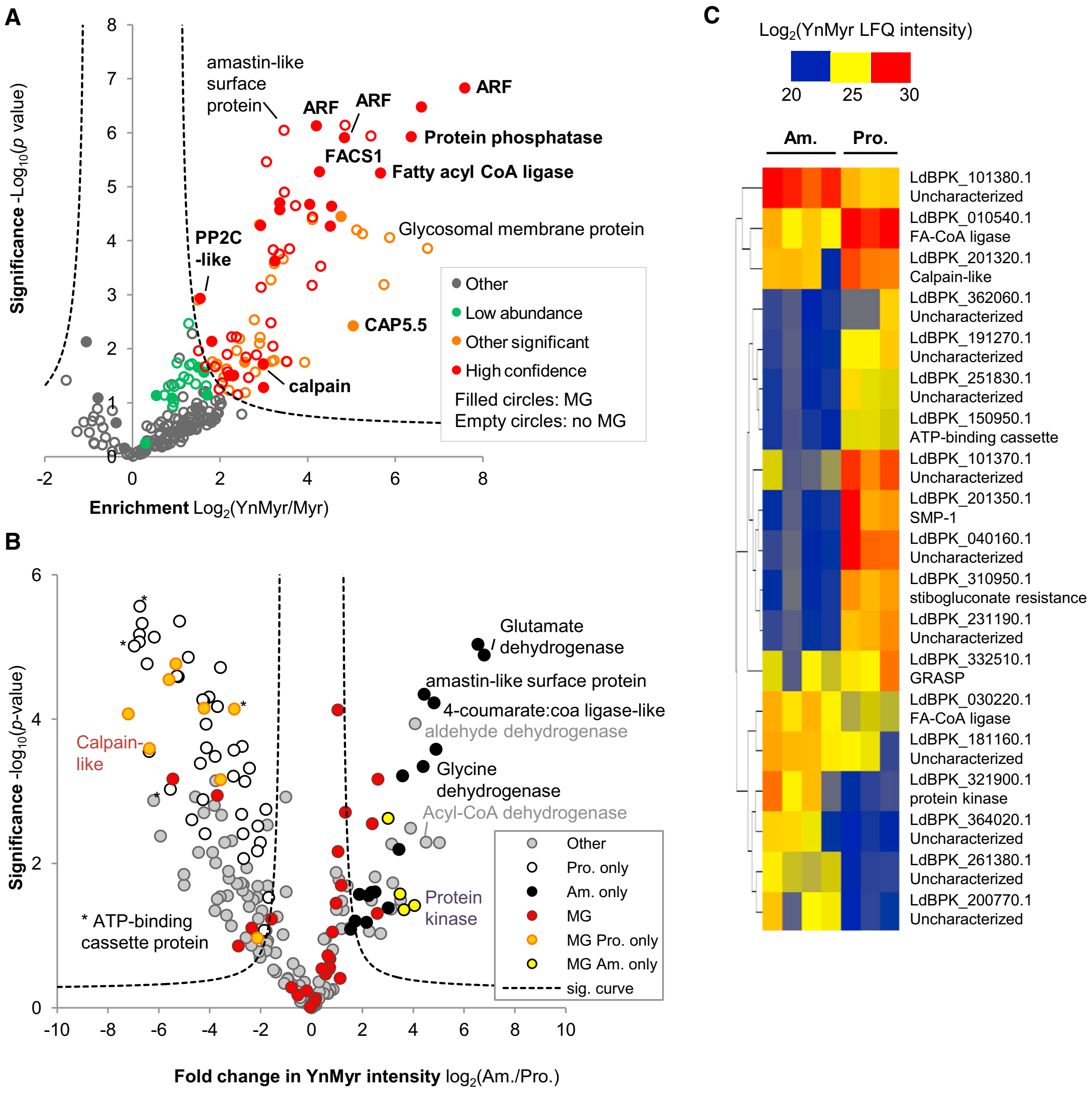

Figure 3. Proteomic Analysis of YnMyr Tagged Proteins in *Leishmania*

(A) Volcano plot showing the results of a two-sample t test (FDR 0.05, S0 1) applied to amastigote LFQ intensities to assess enrichment in YnMyr samples over myristic acid controls (myr). MG, sequence contains an N-terminal glycine; high confidence, significant and absent from myr controls; low abundance, not significant in the t test but absent from controls; other significant, significant at FDR 0.05 but also detected in myr controls; other, other proteins identified. See also Figure S2.

(B) Comparison of YnMyr intensities of amastigote and promastigote hits (t test significant and low abundance hits) by t test (FDR 0.05, S0 2) after imputation of missing values. Proteins are color coded to indicate those containing an N-terminal glycine (MG) and those found in only one of promastigote (Pro.) or amastigote (Am.) data sets.

(C) Heatmap of YnMyr intensities across replicates for those MG proteins significantly differing between Am. and Pro.

was approached in two ways. First, values for missing LFQ intensities were imputed from a normal distribution to simulate values at the lower limit of detection and a permutation-corrected (false discovery rate, FDR) t test was applied to define significance (Figure 3A; Figure S2). Second, the absence of a protein from controls was considered. Proteins were defined as high confidence hits if they reached significance in the t test and were absent from controls, while a protein was defined as a low abundance hit if it was absent from controls but did not reach statistical significance due to low intensity in the YnMyr samples. This latter group is more likely to contain false positives in addition to true hits.

In amastigotes, 81 proteins were significantly enriched (FDR 0.05) and, of these, 49 proteins were assigned as high confidence using the definition above. A further 20 proteins were classified as low abundance hits (Table S1). In promastigotes, 134 proteins were significantly enriched (FDR 0.01) and, of these, 113 were high confidence; 28 proteins were assigned as low abundance (Table S2). Notably, 59% of high confidence hits in amastigotes did not contain an N-terminal glycine (MG),

proposed to be a prerequisite of NMT-dependent myristoylation. In promastigotes, this figure rose to 78%, suggesting that YnMyr may not exclusively tag *N*-myristoylated proteins in *Leishmania*. However, a number of proteins were indeed detected for which there is prior experimental evidence for *N*-myristoylation in *Leishmania*, including ARL1 (Sahin et al., 2008), SMP-1 (Tull et al., 2004), CAP5.5 (Hertz-Fowler et al., 2001), and PPEF (Mills et al., 2007). Other identified MG proteins include other ARF/ARL proteins, Golgi reassembly stacking protein GRASP, several other calpain-like cysteine peptidases, and protein phosphatases/kinases.

Probable integral membrane proteins were prominent in the non-MG data sets; of the 107 significant non-MG hits in promastigotes, 57 had predicted transmembrane (TM) helices, and of the 58 non-MG hits in amastigotes, 28 had this feature (Tables S1 and S2). Conversely, only 3 MG proteins across both data sets had a predicted TM domain. *S*-Palmitoylation of TM proteins is well known to occur in other eukaryotes (Blaskovic et al., 2013) and it is possible that YnMyr is incorporated into these proteins by palmitoyltransferases. Indeed, bioinformatic analysis of the protein sequence suggested that many hits that lacked the MG motif could be *S*-palmitoylated (Figure S2; Table S3). Putative GPI-anchored proteins were enriched by YnMyr tagging, including five uncharacterized proteins (LdBPK_331200.1, LdBPK_352190.1, LdBPK_090690.1, LdBPK_364340.1, LdBPK_350860.1), three surface antigens (LdBPK_050900.1, LdBPK_051210.1, LdBPK_040180.1), and a putative vacuolar-type proton translocating pyrophosphatase (Table S3).

Comparison of hits (defined as proteins significantly enriched over myristic acid controls or low abundance hits) in the two *L. donovani* life stages showed high overlap of 67% (Table S3). A t test was used to compare the YnMyr LFQ intensities of hits (Figure 3B) revealing that select MG proteins are differentially expressed. Proteomic studies have revealed changes related to the transition from promastigote to amastigote stage, in particular an increase in proteins associated with gluconeogenesis and mitochondrial metabolism in amastigotes (Biyani and Madhubala, 2012; Rosenzweig et al., 2008). The most strongly differentially regulated putative *N*-myristoylated proteins identified here are mostly proteins of unknown function, apart from SMP-1 and sodium stibogluconate resistance protein, enriched in promastigotes, and a putative protein kinase found only in amastigotes (Figure 3C). Overall, likely *N*-myristoylated proteins with important functions including transport, protein (de)phosphorylation, proteasomal degradation, and Golgi function are conserved in both *Leishmania* life stages.

Chemical Knockdown of *N*-Myristoylation Defines NMT Substrates

Although the global proteomic approach identified a number of likely *N*-myristoylated proteins, the complexity of YnMyr labeling in *Leishmania* complicates the firm assignment of specific proteins as NMT substrates. We therefore sought to use chemical knockdown of NMT in combination with YnMyr tagging to distinguish those proteins that are *N*-myristoylated from other lipidated proteins, an approach recently used to define substrates of the human NMTs in cancer cells (Thinon et al., 2014). Compound **1** (Figure 4A) was first reported as a *T. brucei* NMT inhibitor with in vivo efficacy in a rodent model of trypanosomiasis (Brand et al., 2012; Frearson et al., 2010), and we have subsequently shown that this inhibitor also acts on-target to inhibit NMT in malaria parasites (Wright et al., 2014) and human cancer cell lines (Thinon et al., 2014). Compound **1** has a $K_i^{(app)}$ (apparent inhibition constant) of 2.5 nM against LdNMT and EC_{50}s (half maximal effective concentrations) of 7 μM and 23 μM against Ld amastigotes and promastigotes, respectively (Paape et al., 2014). Parasites were preincubated with **1** for 1 hr at varying concentrations and then YnMyr was added for 12 hr. Following CuAAC, a drop in intensity in specific bands could be observed in both life stages in response to inhibitor (Figures 4B and 4C). Many bands were completely unaffected, possibly because these proteins are not tagged via NMT activity, consistent with the proteomic analyses above suggesting that YnMyr does not label only *N*-myristoylated proteins. Blotting for HASPB after pull-down revealed a dose-dependent decrease in the YnMyr tagging of this protein (Figures 4B and 4C). These data suggest that compound **1** inhibits NMT inside the cell.

While **1** translates very poorly from enzyme to cellular potency, analog **2** shows similar enzyme inhibitory activity but is nearly 50-fold more potent in the amastigote growth inhibition assay (Figure 4A). We hypothesized that this contrast could be explained by the distinct physicochemical properties of the compounds, which can affect uptake, efflux, and metabolism, or by off-targets of **2**. Incubation with **2** at a concentration around its EC_{50} (0.2 μM) gave a similar decrease in YnMyr labeling to that observed at the EC_{50} of **1** (7 μM) (Figure 4D), suggesting that despite very different translation from recombinant enzyme to cell potency, both inhibitors engage NMT inside the cell. As expected, a lower concentration of **1** (0.2 μM) resulted in only a partial loss of specific labeling.

Inhibited samples were subject to pull-down, digest, and LC-MS/MS. Biological and technical reproducibility of LFQ intensities across replicates was high (R^2 0.76–0.96 after imputation of missing values; Figure S4) and the 580 proteins present in biological duplicates in the YnMyr samples were selected for further analysis (Table S4). After filtering out nonspecific binders, missing values were imputed. ANOVA (Benjamini Hochberg correction, FDR 0.001) was used to compare across all four conditions (YnMyr, 0.2 μM **2**, 0.2 μM **1**, and 7 μM **1**) and permutation-corrected t tests were used to compare each inhibition condition with YnMyr controls. In all analyses there was a significant reduction in intensity in a subset of MG proteins (Figure 5A; Table S4; Figure S5). Relative to the YnMyr control, 30 proteins in the 7 μM **1** data set were significantly reduced and all contained an N-terminal glycine.

Several bioinformatic tools (Bologna et al., 2004; Maurer-Stroh et al., 2002) have been developed to predict *N*-myristoylation. However, these tools are based on learning sets derived from other eukaryotes and, of the MG proteins identified here as enriched through YnMyr tagging, the predictors disagreed on whether a protein was a substrate in 30% of cases (Table S5). In addition, several high confidence hits were assigned as non-myristoylated by both tools, underlining the value of experimental approaches to test bioinformatic predictions.

For those hits detected and assigned as significant at both concentrations of inhibitor **1**, there was a clear dose-dependent decrease in intensity in response to **1** (Figure 5C). Several MG

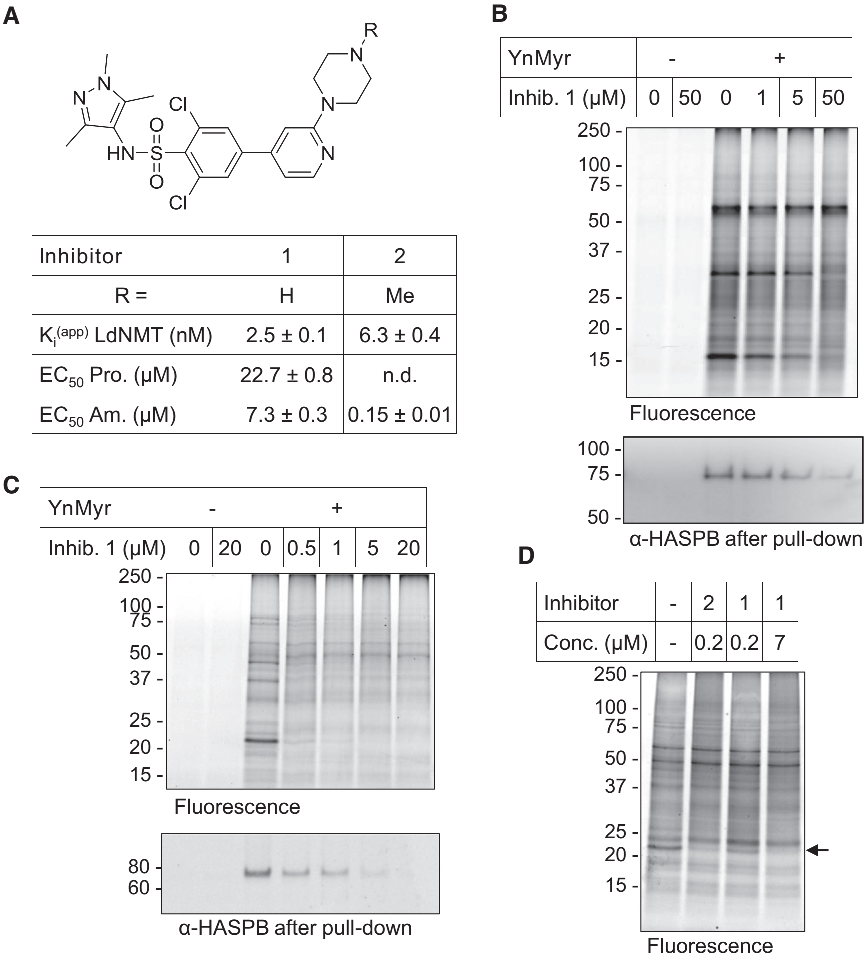

Figure 4. Effect of NMT Inhibition on YnMyr Tagging

(A) Structures of inhibitors **1** and **2** and summary of their activity against recombinant LdNMT (apparent inhibition constant, $K_i^{(app)}$) and against promastigotes (Pro.) or ex vivo amastigotes (Am.) (half maximal effective concentration, EC_{50}).

(B) Gel-based analysis of YnMyr tagging in promastigotes treated with inhibitor **1**. Top: in-gel fluorescence showing the effect of increasing **1** concentrations. Bottom: western blot (WB) of HASPB following enrichment of YnMyr tagged proteins by pull-down (PD) onto streptavidin beads.

(C) Gel-based analysis of YnMyr tagging in amastigotes treated with inhibitor **1**. Top: in-gel fluorescence showing the effect of increasing **1** concentrations. Bottom: WB of HASPB following enrichment of YnMyr tagged proteins by PD onto streptavidin beads.

(D) In-gel fluorescence analysis of YnMyr tagging in amastigotes treated with inhibitor **1** or **2**. Arrow indicates most prominent loss of YnMyr tagging upon inhibition. Full blots and Coomassie gels are shown in Figure S3.

proteins showed no response and a subset of hits were significantly decreased only at the higher concentration (7 µM) of **1** (Table S4). This latter group may contain proteins that are less sensitive to NMT inhibition, possibly higher affinity substrates (Thinon et al., 2014). Other proteins (Figure 5D) also did not show strong responses to **1** and **2**. All 16 proteins significantly affected by inhibitor **2** were also hits in the **1** data set (Figure 5B; Table S4). However, comparing the LFQ intensities of hits qualitatively and via a t test indicated that in fact 0.2 µM **2** did not inhibit *N*-myristoylation as effectively as 7 µM **1** (Figure S5). A potential explanation for this result is that **2** does inhibit NMT in amastigotes but may have additional off-target effects that slightly increase its potency in cells.

Since 7 µM **1** resulted in the strongest response, this data set was used in combination with visual examination of heatmaps to define high and low confidence hits (Table 1; Table S4; Figure 6A). All 30 proteins significant in the t test were defined as high confidence NMT substrates since they showed either a dose-response relationship or were absent from inhibited samples. Low confidence hits were defined as those not reaching significance in the t test but also not detected in the inhibited sample, and include proteins expected to be *N*-myristoylated such as GRASP, a calpain, and a serine/threonine phosphatase, but also four proteins that have no N-terminal MG motif and so are unlikely to be myristoylated. Of the 30 high confidence proteins, 18 had been identified as significant or low abundance hits in the global amastigote analysis, but a further 11 had been identified in only one or two replicates (Table S1), highlighting the value of employing additional tools to explore such complex samples.

To provide further insight into the YnMyr modification of proteins, we used the trypsin-cleavable site in AzRB to enable direct identification of YnMyr-modified peptides. De novo-aided sequencing (Zhang et al., 2012) was used to search for peptides containing the YnMyr-AzRB-derived adduct at their N terminus. A total of 20 proteins were detected with this modification on a glycine residue corresponding to the N terminus of the protein (Table S6), and no peptides were identified in searches of myristic acid controls. Of these 20 proteins, 8 had been identified as high confidence NMT substrates via quantification of their response to NMT inhibition and include known *N*-myristoylated protein LdARL1, a proteasome subunit, a PP2C-like protein, a predicted protein kinase and four uncharacterized proteins (Tables 1 and S6; Figure 6A). An additional four lower confidence substrates were also modified. Interestingly, modification on the N-terminal glycine of three proteins (two fatty acyl-CoA ligases and uncharacterized LdBPK_300680.1) that did not respond robustly to NMT inhibition was detected; these proteins may be NMT substrates that are additionally modified elsewhere, for example, at their enzyme active site or at alternative sites, or may be modified on their N terminus by a mechanism distinct from NMT. Several other modified proteins were identified that had not been included in the global analyses due to weak detection. Gene ontology analysis of the 30 high confidence NMT substrates revealed that they have diverse functions and include

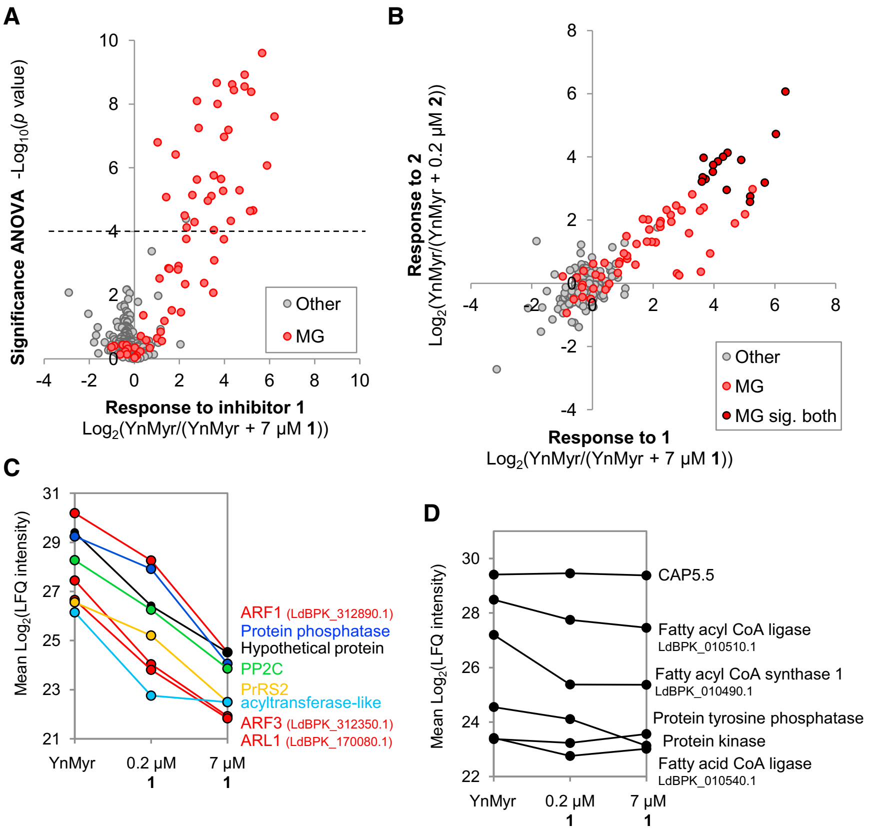

Figure 5. Proteomics Analysis of YnMyr Tagged Proteins in Amastigotes Treated with NMT Inhibitors

(A) ANOVA test (Benjamini Hochberg correction, FDR 0.001) applied to LFQ intensities to compare inhibition conditions. The fold change in response to 7 μM **1** is plotted against $-\log_{10}(p$ value). Proteins above the dashed line reached significance in this test. MG, sequence contains an N-terminal glycine. Volcano plots of two-sample t tests are shown in Figure S5.

(B) Comparison of LFQ ratios for 0.2 μM **2** and 7 μM **1** data sets after removal of nonspecific binders. The proteins assigned as significantly affected by both inhibitors are indicated. See Figure S5 for direct comparison of these ratios by t test.

(C) Response of selected MG proteins to 0.2 and 7 μM **1**. Mean LFQ intensity before imputation of missing values is plotted.

(D) MG proteins where YnMyr tagging does not respond to NMT inhibition.

phosphatases and kinases, proteins involved in transport and degradation, as well as many (~50%) proteins of unknown function (Figure 6B).

DISCUSSION

Here we combine chemical proteomic tools with inhibitors and quantitative mass spectrometry to define the complement of *N*-myristoylated proteins in *L. donovani* parasites, and explore NMT as a potential drug target in this important human pathogen. Proteomic and gel-based analyses reveal that YnMyr is incorporated into diverse sets of proteins, including *N*-myristoylated and GPI-anchored proteins, as well as into what are most likely complex glycolipids. YnMyr incorporation into proteins other than NMT substrates is not unprecedented; for example, we observed significant YnMyr tagging of GPI-anchored proteins in malaria parasites (Wright et al., 2014). However, the proteomic analyses carried out here suggest that while YnMyr does tag GPI-anchored proteins in Ld, these are by no means the most abundant hits. A possibility is incorporation into *S*-acylation sites, since in mammalian cells YnMyr and similar probes label both *N*-myristoylated and *S*-palmitoylated proteins (Charron et al., 2009; Thinon et al., 2014) and palmitoyltransferases have varying acyl-CoA specificities (Jennings and Linder, 2012). Alternatively, YnMyr may be metabolized to longer or shorter chain analogs retaining the alkyne tag that are subsequently incorporated into proteins, or it may be that alternative lipid modifications, such as the poorly characterized lysine *N*-acylation (Lin et al., 2012), are prevalent in *Leishmania*. Although enzymes with lysine deacylase activity have been described (Jiang et al., 2013), those that carry out lysine acylation have not been identified and it has been suggested that such modifications may in fact be nonenzymatic (Wagner and Hirschey, 2014). *Leishmania* may metabolize YnMyr to a reactive intermediate that nonenzymatically modifies proteins.

In addition to identification of lipidated proteins, we provide evidence that compounds **1** and **2** inhibit NMT in live parasites. While the potent in vitro activities of these compounds translate to cellular activity rather poorly, and to very different extents, we show here that this activity is accompanied by a drop in *N*-myristoylation levels of specific proteins and thus provide the first direct evidence for the druggability of NMT in *Leishmania*. Problems with translation from enzyme to cellular assays have been reported previously in *Leishmania*, particularly in amastigotes (Walker et al., 2011), but we anticipate that the methodology developed here will be useful for screening NMT inhibitors for target engagement inside the cell. Further experiments are now required to confirm that the antiparasitic effects of inhibitors **1** and **2** arise solely through inhibition of NMT, and to identify which NMT substrates mediate these effects.

The depth of coverage of the YnMyr tagged proteome identified could be improved; for example, HASPB, despite being detectably tagged in gel-based analyses, was not identified in MS studies; this may be due in large part to the sequence of this protein, which contains multiple repeats and is highly hydrophilic. Several possible NMT substrates were detected but did not meet the stringent filtering criteria applied and it is thus likely that more NMT substrates remain to be validated in the future.

The set of proteins assigned as NMT substrates here is generally consistent with previous literature on *N*-myristoylated proteins in *Leishmania* and other eukaryotes. Surprisingly, the lipidation levels of CAP5.5 did not respond to inhibition of NMT

Table 1. High and Low Confidence NMT Substrates Identified by Quantitative Proteomics with YnMyr in Combination with NMT Inhibitors

Mean LFQ intensities				Sig.	T-test significant						
Yn Myr	0.2 µM 1	0.2 µM 2	7 µM 1	AN OVA	0.2 µM 1	0.2 µM 2	7 µM 1	Mod. Pept.	MG	Protein ID	Product Description
**High confidence hits.** Response to NMT inhibition.											
30.2	28.3	27.0	24.5	+		+	+		+	LdBPK_312890.1	ADP-ribosylation factor (ARF1)
29.4	26.4	25.5	24.5	+	+	+	+	+	+	LdBPK_101380.1	uncharacterized protein
29.2	27.9	26.7	24.1	+		+	+		+	LdBPK_250780.1	protein phosphatase
28.3	26.3	25.3	23.9	+		+	+		+	LdBPK_300380.1	phosphatase 2C
27.4	24.0	21.9	21.9	+	+	+	+		+	LdBPK_312350.1	ADP-ribosylation factor (ARF3)
26.7	23.8	22.9	21.8	+	+	+	+	+	+	LdBPK_170080.1	ADP-ribosylation factor (ARL1)
26.6	25.2	23.8	22.5	+		+	+	+	+	LdBPK_130990.1	proteasome regulatory ATPase subunit 2
26.6	25.0	24.1	22.3	+			+		+	LdBPK_201320.1	calpain-like cysteine peptidase
26.5	25.6	24.6	22.3	+			+		+	LdBPK_181160.1	uncharacterized protein
26.3	25.8	25.4	23.1				+	+	+	LdBPK_360030.1	uncharacterized protein
26.3	26.3	26.0	23.5	+			+	+	+	LdBPK_321900.1	protein kinase
26.1	22.8	22.3	22.5	+	+	+	+		+	LdBPK_041050.1	acyltransferase-like protein
25.8	25.7	25.5	22.9	+			+		+	LdBPK_210100.1	uncharacterized protein
25.5	25.5	25.3	22.7	+			+	+	+	LdBPK_331090.1	uncharacterized protein
25.3	24.6	23.5	NaN	+			+		+	LdBPK_200770.1	uncharacterized protein
24.8	NaN	NaN	NaN	+	+	+	+	+	+	LdBPK_101370.1	uncharacterized protein
24.7	22.9	NaN	NaN	+		+	+		+	LdBPK_040470.1	ADP-ribosylation factor
24.6	NaN	20.3	NaN	+	+	+	+	+	+	LdBPK_360560.1	protein phosphatase 2C-like protein
24.4	22.9	NaN	NaN	+		+	+		+	LdBPK_271740.1	uncharacterized protein
24.3	20.4	19.1	NaN	+	+	+	+		+	LdBPK_260270.1	uncharacterized protein
24.1	21.5	NaN	NaN	+	+	+	+		+	LdBPK_360400.1	PI-4-phosphate 5-kinase-like protein
24.1	22.8	21.5	NaN	+			+		+	LdBPK_200750.1	uncharacterized protein
24.0	22.2	NaN	NaN	+		+	+		+	LdBPK_010400.1	uncharacterized protein
24.0	22.8	21.7	NaN				+		+	LdBPK_230530.1	uncharacterized protein
23.9	NaN	NaN	NaN	+	+	+	+		+	LdBPK_340980.1	uncharacterized protein
23.6	21.0	NaN	NaN	+			+		+	LdBPK_161450.1	ADP-ribosylation factor-like (ARL6)
23.3	21.3	21.0	20.8	+			+		+	LdBPK_230500.1	trypanothione synthetase
23.2	NaN	NaN	NaN	+	+		+		+	LdBPK_261380.1	uncharacterized protein
23.0	NaN	NaN	NaN	+	+		+		+	LdBPK_200810.1	uncharacterized protein
22.9	21.7	NaN	NaN				+		+	LdBPK_311540.1	uncharacterized protein
**Low confidence hits.** Response to inhibition but detected at low intensity.											
24.9	24.0	23.1	NaN						+	LdBPK_332510.1	Golgi reassembly stacking protein (GRASP)
24.6	24.3	NaN	NaN							LdBPK_201290.1	uncharacterized protein
24.4	NaN	NaN	NaN					+	+	LdBPK_201350.1	calpain-like cysteine peptidase, SMP1
22.9	NaN	NaN	NaN					+	+	LdBPK_241400.1	uncharacterized protein
22.7	NaN	NaN	NaN	+	+					LdBPK_301700.1	uncharacterized protein
22.7	NaN	NaN	NaN	+					+	LdBPK_364660.1	uncharacterized protein
22.7	22.2	NaN	NaN						+	LdBPK_365910.1	uncharacterized protein
22.4	21.4	NaN	NaN					+	+	LdBPK_291980.1	uncharacterized protein
22.3	21.6	21.7	NaN						+	LdBPK_080630.1	uncharacterized protein
22.3	NaN	NaN	NaN						+	LdBPK_091640.1	uncharacterized protein
22.2	NaN	NaN	NaN						+	LdBPK_252140.1	uncharacterized protein
22.2	NaN	NaN	NaN						+	LdBPK_030720.1	cytochrome c oxidase copper chaperone
22.1	21.4	NaN	NaN					+	+	LdBPK_311430.1	uncharacterized protein
21.9	21.8	NaN	NaN						+	LdBPK_311530.1	uncharacterized protein
21.9	NaN	NaN	NaN						+	LdBPK_100620.1	zinc-binding dehydrogenase-like protein
21.8	NaN	NaN	NaN							LdBPK_353550.1	uncharacterized protein
20.9	NaN	NaN	NaN						+	LdBPK_231150.1	uncharacterized protein
20.5	NaN	NaN	NaN						+	LdBPK_161290.1	uncharacterized protein
**Possible substrates (unclear).** Little or no response to inhibition, but modified peptide identified in some cases.											
27.2	25.4	25.2	25.4	+					+	LdBPK_010490.1	fatty acyl CoA syntetase 1
23.7	21.9	21.8	21.4	+					+	LdBPK_120610.1	ser/thr protein phosphatase-like, PPEF
28.5	27.7	27.5	27.5	+				+	+	LdBPK_010510.1	long chain fatty acid CoA ligase
27.0	26.9	26.5	25.6	+				+	+	LdBPK_300680.1	uncharacterized protein
23.4	22.8	22.8	23.0					+	+	LdBPK_010540.1	long chain fatty acid CoA ligase

Mean log_2(LFQ intensity) is given and cells are color coded based on value. NaN, not a number (not found); Sig., significant. Proteins for which the YnMyr-modified N terminus was detected are indicated. See also Tables S4 and S6.

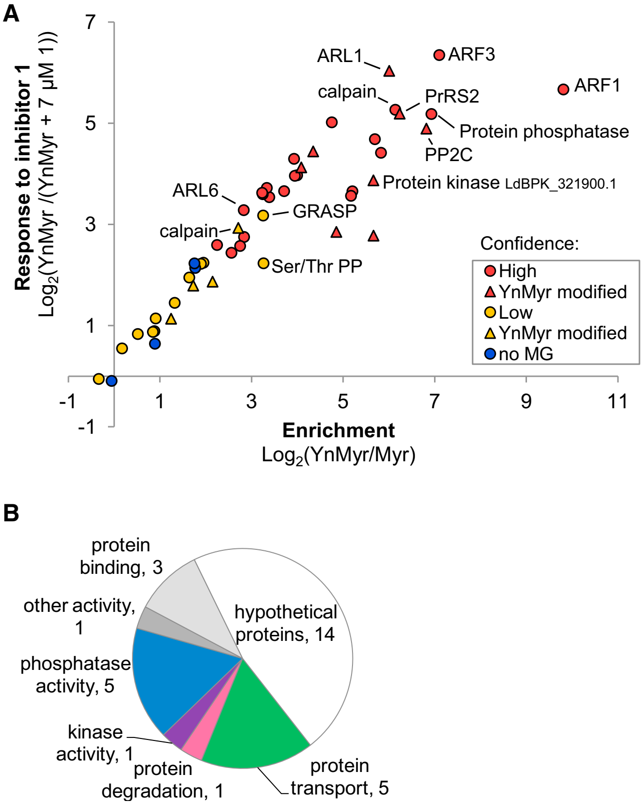

Figure 6. Analysis of Proteins whose Acylation Responds to NMT Inhibitors

(A) Enrichment over myristic acid (Myr) control compared with response to inhibitor (7 μM **1**) for hits. High confidence, significantly affected by inhibition (t test FDR 0.001); low confidence, not detected in inhibitor-treated samples but not significant in the t test due to low intensity in the YnMyr controls. Proteins for which YnMyr modification on the N-terminal glycine was detected are indicated (see also Table S6).

(B) GO annotation (TriTrypDB) of 30 high confidence NMT substrates.

(Figure 5D; Table S4). CAP5.5 contains a strongly predicted myristoylation motif (Table S5) and its ortholog has been shown experimentally to be *N*-myristoylated and *S*-palmitoylated in *T. brucei*, the latter presumably on a nearby cysteine residue (Hertz-Fowler et al., 2001). Similarly, although the phosphatase PPEF did respond to NMT inhibition, basal YnMyr tagging was still detectable (Table 1). There is evidence that PPEF is dually acylated in *L. major*, with palmitoylation being dependent on myristoylation, suggesting that in the absence of myristoylation no labeling should occur (Mills et al., 2007). It is possible that the relatively high concentration of YnMyr used here could force incorporation into *S*-acylation or other sites, or that experimental differences between studies may account for these different results; the dynamic nature of *S*-acylation may render labeling highly dependent on incubation time and other culture conditions. Alternatively, we cannot exclude the existence of another enzyme with acyltransferase activity that carries out N-terminal *N*-lipidation in *Leishmania*, although no experimental or bioinformatic evidence has yet been reported to suggest this, and there is no evidence for compensation on deletion of NMT in promastigotes (Brannigan et al., 2010).

Just over half of the 30 high confidence NMT substrates identified here are uncharacterized proteins, highlighting the huge amount of NMT-related biology yet to be explored in *Leishmania*. However, some predictions for the outcome of NMT inhibition can be drawn from these data. ARF/ARL proteins, of which five were identified as strongly affected by NMT inhibition, are important mediators of intracellular trafficking in all eukaryotes and their specific localization is often dependent on *N*-myristoylation. LdARL1 is localized to the *trans*-Golgi network and involved in control of endocytosis (Sahin et al., 2008); its *T. brucei* homolog is also a Golgi protein essential in bloodstream parasites, where its depletion causes defects in exocytosis and Golgi structure (Price et al., 2005). TbARL6 is involved in flagellum extension, interaction with microtubules, and the BBSome (Price et al., 2012), which is important for specific trafficking events in all eukaryotes and is essential for virulence in *Leishmania* (Price et al., 2013). Therefore basic cellular functions such as Golgi structure, endo- and exocytosis are very likely to be affected by inhibition of NMT, perhaps affecting parasite virulence. The identification of three protein phosphatases, one kinase and phosphatidylinositol phosphate kinase, suggests that signaling processes dependent on specific (de)phosphorylation events will also be disrupted. Finally, the proteasome has already been shown to be a potential drug target in *Plasmodium* (Li et al., 2014) and *N*-myristoylation of this subunit linked to function in yeast, where removal of the myristoylation site causes proteasome mislocalization, accumulation of misfolded protein, and growth defects (Kimura et al., 2012). In summary, these data predict a strongly pleiotropic effect of NMT inhibition on parasite cellular function and provide evidence for NMT as a valid drug target in *Leishmania*.

SIGNIFICANCE

The leishmaniases are a spectrum of neglected tropical diseases for which few drugs are available and novel approaches are urgently required. A deeper understanding of the underlying biology, and chemical validation, of potential drug targets may improve the chances of successfully developing new treatments. NMT catalyzes a specific and essential lipidation of proteins in eukaryotes and the present study explores protein lipidation in *Leishmania donovani*, the causative agent of the most severe form of the leishmaniases. We take advantage of the global picture afforded by a chemical proteomic approach using a bioorthogonally tagged lipid analog to show that protein lipidation in the parasite is diverse and complex. To overcome this complexity and identify NMT substrates, we quantify protein lipidation levels in the presence of NMT inhibitors and support this with direct lipidation site identification. These results predict pleiotropic effects of NMT inhibition in parasites and provide a method to assay for target engagement in the cell. This work also constitutes the first global experimental analysis of protein lipidation in *Leishmania* and contributes a large proteomic data set for this neglected human pathogen. These data may provide further insights into other drug targets, for example, the complex glycolipid pathways or individual functionally important NMT substrates.

EXPERIMENTAL PROCEDURES

Supplemental Experimental Procedures

The following procedures were carried out essentially as described previously and details are given in the Supplemental Experimental Procedures: ex vivo amastigote inhibition, macrophage cytotoxicity, and enzyme inhibition assays (Hutton et al., 2014); sample preparation for proteomics analysis and LC-MS/MS (Thinon et al., 2014).

Chemical Tools

The following chemical tools were synthesized as described previously: YnMyr, YnPal, and AzTB (Heal et al., 2011); AzMyr and YnTB (Heal et al., 2012); inhibitors **1** and **2** (Thinon et al., 2014). Synthesis of AzRB is given in the Supplemental Experimental Procedures. Myristic and palmitic acids, cycloheximide (CHX), and all other chemicals were purchased.

Ethics Statement

Animal experiments were approved by the University of York Animal Procedures and Ethics Committee and performed under UK Home Office license (Immunity and Immunopathology of Leishmaniasis Ref. no. PPL 60/3708).

Parasite Culture

The Ethiopian LV9 strain of *Leishmania donovani* (MHOM/ET/67/L28) was maintained by serial passage in Rag-$2^{-/-}$ mice. Amastigotes were extracted from spleen as described previously (Smelt et al., 1997) and cultured in RPMI/10% fetal calf serum (FCS). Promastigotes were obtained by transforming 1×10^7 freshly isolated amastigotes in promastigote medium (St-Denis et al., 1999) at 26°C (~2 days for transformation). Promastigotes were passaged twice a week for up to 10 passages. Further details are given in the Supplemental Experimental Procedures.

Metabolic Tagging Experiments

Promastigotes were cultured in RPMI/10% FCS containing the appropriate probe (50 μM YnMyr, myristic acid, or as indicated) at a parasite density of 7.5×10^7 parasites/ml. Cultures were incubated at 26°C for 12 hr (or as indicated). Cells were collected (800 × *g*, 15 min, 4°C), washed twice with cold PBS, then lysed at 1×10^9 parasites/ml by sonication on ice (4 × 10 s burst, amplitude 45 with 1 min interval) in 1% NP40, 1% sodium deoxycholate, 0.5% SDS, 50 mM Tris (pH 7.4), 150 mM NaCl, EDTA-free protease inhibitor. Insoluble material was separated by centrifugation (16,100 × *g*, 30 min, 4°C). Tagging of ex vivo amastigotes was carried out as above but parasites were cultured at 37°C. For inhibition studies, the appropriate inhibitor was preincubated with parasite culture for 1 hr at 37°C, parasites pelleted and resuspended in fresh medium containing inhibitor plus probe (YnMyr etc). Further details are given in the Supplemental Experimental Procedures.

Intracellular Amastigote Tagging

BALB/c mice were obtained from Charles River. Macrophages (BMDM) were differentiated from bone marrow of BALB/c mice as described previously (Weinheber et al., 1998) and plated out at 1×10^6 cells per well in six-well plates. BMDMs were adhered overnight and then infected at a multiplicity of infection of 10 with freshly isolated *L. donovani* amastigotes (see above). After 24 hr no free parasites were observed. Tagging was performed for 18 hr with YnMyr or myristic acid at a final concentration of 100 μM. Cells were washed three times with PBS at room temperature (RT) before 300 μl of lysis buffer was added and cells scraped off. The lysate was sonicated (3× 10 s, amplitude 45 with 1 min intervals), then centrifuged for 30 min at 16,100 × *g* at 4°C. The supernatant was transferred into a fresh tube and flash frozen in liquid nitrogen. BMDMs were maintained in DMEM supplemented with 4 mM L-glutamine and 4% L929-cell conditioned medium. All experiments were performed at 37°C and 5% CO_2.

CuAAC Labeling and Pull-down

Proteins were precipitated with chloroform/methanol ($MeOH:CHCl_3:ddH_2O$ 4:1:3), or acetone (4 volumes at −20°C for 1 hr) and then resuspended at 1 mg/mL in 1% SDS in PBS. This precipitation step was found to increase labeling intensity after CuAAC. Premixed click reagents (100 μM AzTB, 1 mM $CuSO_4$, 1 mM TCEP, 100 μM TBTA, final concentrations) were added (Heal et al., 2012) and samples vortexed for 1 hr at RT, then quenched by the addition of 10 mM EDTA. Proteins were precipitated again with MeOH/$CHCl_3$ or with 10 volumes of MeOH (overnight at −80°C), washed with ice-cold MeOH, air-dried, and resuspended in 2% SDS, 10 mM EDTA in PBS. For direct gel analysis, 4× sample loading buffer (NuPAGE LDS sample buffer) with 2-mercaptoethanol (4% final) was added. For hydroxylamine (NH_2OH) and NaOH treatment, samples were treated with 1 M NH_2OH (pH 7) or 0.2 M NaOH at RT for 1 hr. Samples were quenched by addition of 4× sample loading buffer. Note that addition of EDTA to the samples before NH_2OH treatment is important to avoid sample degradation. Proteins were heated for 3 min at 95°C prior to SDS-PAGE. Pull-down was carried out with Dynabeads MyOne Streptavidin C1 as described previously (Wright et al., 2014) (see Supplemental Experimental Procedures).

Gel and Western Blot Analysis

Samples were separated by SDS-PAGE and scanned with Cy3 filters to detect the TAMRA fluorophore using an Ettan DIGE scanner (GE Healthcare).

For western blot, proteins were transferred from gels to polyvinylidene fluoride membrane (Immobilon-P^{SQ}, Millipore) using a semidry system (Invitrogen). Following blocking (5% milk in Tris-buffered saline, 1% Tween), membranes were incubated with primary antibody for 1 hr at RT or overnight at 4°C in blocking solution, then incubated with secondary antibody (goat anti-rabbit IgG-HRP, Invitrogen, 1:10,000) for 1 hr in blocking solution. Detection was carried out using Luminata Crescendo Western HRP substrate (Millipore) according to the manufacturer's instructions and on a Fujifilm LAS 3000 imager. Primary antibodies, LdHASPB (rabbit; ab 2-AE) (Alce et al., 1999), LmHASPB (rabbit, 336) (Alce et al., 1999), GP63 (rabbit, polyclonal; provided by R. McMaster, University of British Columbia) (Frommel et al., 1990), GFP (mouse, Santa Cruz), were used at 1:1,000–2,000.

Proteomic Data Searching and Analysis

Data Searching and Analysis

The data were processed with MaxQuant version 1.3.0.5 and the peptides were identified from the MS/MS spectra searched against The TriTrypDB-6.0 *L. donovani* LdBPK282A1 database using the Andromeda search engine. The protein HASPB was not present in this database and so the Uniprot sequence (O77300_LEIDO) was appended to the FASTA file. Cysteine carbamidomethylation was used as a fixed modification and methionine oxidation and N-terminal acetylation as variable modifications. The false discovery rate was set to 0.01 for peptides, proteins, and sites. Other parameters were used as preset in the software. "Unique and razor peptides" mode was selected to allow for protein grouping; this calculates ratios from unique and razor peptides (razor peptides are uniquely assigned to protein groups and not to individual proteins). Data were elaborated using Perseus versions 1.4.0.20 and 1.5.0.31. LFQ experiments in MaxQuant were performed using the built-in label-free quantification algorithm (Cox et al., 2014).

Modified Peptide Identification

MS data were processed with PEAKS7 suite (Zhang et al., 2012) The data were searched against a reference *L. donovani* database from Uniprot (27/11/2014). Trypsin (specific, up to three missed cleavages allowed) was selected for database searches and no enzyme was chosen for de novo searches. The maximal mass error was set to 5 ppm for precursor ions and 0.01 Da for product ions. Carbamidomethylation was selected as a fixed modification and methionine oxidation as well as the lipid-derived adduct (+534.3278 Da) to any amino acid at peptide N terminus were set as variable modifications. The maximal number of modifications per peptide was set as five. The false discovery rate was set to 0.01 for peptides and b1 ions were required for N-terminally modified peptides. Within PEAKS, a module called SPIDER searches for point mutations to further enhance the discovery (Ma and Johnson, 2011).

Data Processing: YnMyr Tagged Proteins in Amastigotes and Promastigotes

For amastigotes, four replicates were performed independently, starting from the same metabolically labeled lysates (Myr and YnMyr samples). Data were filtered to require three valid values across the four replicates, label-free intensities were logarithmized (base 2), and empty values were imputed with random numbers from a normal distribution, whose mean and SD were chosen to simulate low abundance values close to noise level (impute criteria: width 0.1, downshift 1.8). A modified t test with permutation-based FDR statistics was applied (250 permutations; FDR 0.05; S0 1).

For promastigotes, three replicates were performed independently, starting from the same metabolically labeled lysates (Myr and YnMyr samples). Data were analyzed as described above with three valid values per group required, imputation (width 0.1, downshift 1.8), and t test (250 permutations; FDR 0.01; S0 1).

For the comparison of amastigotes and promastigotes, data were filtered for at least three valid values in at least one group (groups: Am_Myr, Am_YnMyr, Pro_Myr, Pro_YnMyr). YnMyr intensities of hits (proteins that were either t test significant or classed as low abundance hits in the independent analyses) were compared by t test (250 permutations; FDR 0.05; S0 2) after imputation of missing values (width 0.1, downshift 2.5). Hierarchical clustering was carried out in Perseus.

Data Processing: YnMyr Tagging in the Presence of NMT Inhibitors (Amastigotes)

Independent biological experiments were carried out where amastigotes were incubated with YnMyr, YnMyr + 0.2 μM **2**, YnMyr + 0.2 μM **1**, or YnMyr + 7 μM **1**. A Myr control was included in one of these experiments. For proteomics analysis, all samples were prepared and processed in duplicate (technical replicates: A and B; C and D). The "Match between runs" option (time window 2 min) in MaxQuant was enabled during the searches. Data were grouped, filtered to retain only those proteins present in both biological experiments, and nonspecific binders (enrichment over control <2) removed. Missing values were imputed from a normal distribution (width 0.1, downshift 1.8) and modified two-sample t tests were applied (250 permutations; FDR 0.001; S0 1). ANOVA (Benjamini Hochberg correction, FDR 0.001) was also applied to LFQ intensities to compare across all data sets.

SUPPLEMENTAL INFORMATION

Supplemental Information includes Supplemental Experimental Procedures, six figures, and six tables and can be found with this article online at http://dx.doi.org/10.1016/j.chembiol.2015.01.003.

AUTHOR CONTRIBUTIONS

M.H.W. performed sample handling and analysis downstream of parasite culture. D.P. performed parasite culture, metabolic tagging, and live parasite assays. E.M.S. synthesized reagent AzRB. R.A.S. carried out searches to identify modified peptides. D.F.S. and E.W.T. conceived and directed the study. M.H.W. wrote the manuscript with input from all the other authors.

ACKNOWLEDGMENTS

This work was supported by grants from the UK Engineering and Physical Sciences Research Council (Institute of Chemical Biology studentship and Doctoral Prize Fellowship awards to M.H.W.), Wellcome Trust (087792), British Heart Foundation (studentship to E.M.S.), European Commission (PIEF-GA-2010-273868), UK Biotechnology and Biological Sciences Research Council (BB/D02014X/1) and UK Medical Research Council (MR/L000148/1). The authors thank L. Haigh for assistance with mass spectrometry, W.P. Heal for assistance with the synthesis of inhibitors **1** and **2**, M. Broncel for assistance with AzRB design, and R. McMaster, University of British Columbia, for the GP63 antibody.

Received: October 16, 2014
Revised: December 16, 2014
Accepted: January 13, 2015
Published: February 26, 2015

REFERENCES

Alce, T.M., Gokool, S., McGhie, D., Stager, S., and Smith, D.F. (1999). Expression of hydrophilic surface proteins in infective stages of *Leishmania donovani*. Mol. Biochem. Parasitol. *102*, 191–196.

Alvar, J., Velez, I.D., Bern, C., Herrero, M., Desjeux, P., Cano, J., Jannin, J., and den Boer, M. (2012). Leishmaniasis worldwide and global estimates of its incidence. PLoS One *7*, e35671.

Bell, A.S., Mills, J.E., Williams, G.P., Brannigan, J.A., Wilkinson, A.J., Parkinson, T., Leatherbarrow, R.J., Tate, E.W., Holder, A.A., and Smith, D.F. (2012). Selective inhibitors of protozoan protein N-myristoyltransferases as starting points for tropical disease medicinal chemistry programs. PLoS Negl. Trop. Dis. *6*, e1625.

Biyani, N., and Madhubala, R. (2012). Quantitative proteomic profiling of the promastigotes and the intracellular amastigotes of *Leishmania donovani* isolates identifies novel proteins having a role in *Leishmania* differentiation and intracellular survival. Biochim. Biophys. Acta *1824*, 1342–1350.

Blaskovic, S., Blanc, M., and van der Goot, F.G. (2013). What does S-palmitoylation do to membrane proteins? FEBS J. *280*, 2766–2774.

Bologna, G., Yvon, C., Duvaud, S., and Veuthey, A.L. (2004). N-Terminal myristoylation predictions by ensembles of neural networks. Proteomics *4*, 1626–1632.

Brand, S., Cleghorn, L.A., McElroy, S.P., Robinson, D.A., Smith, V.C., Hallyburton, I., Harrison, J.R., Norcross, N.R., Spinks, D., Bayliss, T., et al. (2012). Discovery of a novel class of orally active trypanocidal N-myristoyltransferase inhibitors. J. Med. Chem. *55*, 140–152.

Brannigan, J.A., Smith, B.A., Yu, Z., Brzozowski, A.M., Hodgkinson, M.R., Maroof, A., Price, H.P., Meier, F., Leatherbarrow, R.J., Tate, E.W., et al. (2010). N-myristoyltransferase from *Leishmania donovani*: structural and functional characterisation of a potential drug target for visceral leishmaniasis. J. Mol. Biol. *396*, 985–999.

Brannigan, J.A., Roberts, S.M., Bell, A.S., Hutton, J.A., Hodgkinson, M.R., Tate, E.W., Leatherbarrow, R.J., Smith, D.F., and Wilkinson, A.J. (2014). Diverse modes of binding in structures of *Leishmania major* N-myristoyltransferase with selective inhibitors. IUCrJ *1*, 250–260.

Charron, G., Zhang, M.M., Yount, J.S., Wilson, J., Raghavan, A.S., Shamir, E., and Hang, H.C. (2009). Robust fluorescent detection of protein fatty-acylation with chemical reporters. J. Am. Chem. Soc. *131*, 4967–4975.

Cox, J., Hein, M.Y., Luber, C.A., Paron, I., Nagaraj, N., and Mann, M. (2014). Accurate proteome-wide label-free quantification by delayed normalization and maximal peptide ratio extraction, termed MaxLFQ. Mol. Cell. Proteomics *13*, 2513–2526.

Cuvillier, A., Redon, F., Antoine, J.C., Chardin, P., DeVos, T., and Merlin, G. (2000). LdARL-3A, a *Leishmania* promastigote-specific ADP-ribosylation factor-like protein, is essential for flagellum integrity. J. Cell Sci. *113*, 2065–2074.

Denny, P.W., Gokool, S., Russell, D.G., Field, M.C., and Smith, D.F. (2000). Acylation-dependent protein export in *Leishmania*. J. Biol. Chem. *275*, 11017–11025.

Etges, R., Bouvier, J., and Bordier, C. (1986). The major surface protein of *Leishmania* promastigotes is anchored in the membrane by a myristic acid-labeled phospholipid. EMBO J. *5*, 597–601.

Ferguson, M.A. (1997). The surface glycoconjugates of trypanosomatid parasites. Philos. Trans. R. Soc. Lond. B Biol. Sci. *352*, 1295–1302.

Frearson, J.A., Brand, S., McElroy, S.P., Cleghorn, L.A., Smid, O., Stojanovski, L., Price, H.P., Guther, M.L., Torrie, L.S., Robinson, D.A., et al. (2010). N-Myristoyltransferase inhibitors as new leads to treat sleeping sickness. Nature *464*, 728–732.

Frommel, T.O., Button, L.L., Fujikura, Y., and McMaster, W.R. (1990). The major surface glycoprotein (GP63) is present in both life stages of *Leishmania*. Mol. Biochem. Parasitol. *38*, 25–32.

Galvin, B.D., Li, Z., Villemaine, E., Poole, C.B., Chapman, M.S., Pollastri, M.P., Wyatt, P.G., and Carlow, C.K. (2014). A target repurposing approach identifies N-myristoyltransferase as a new candidate drug target in filarial nematodes. PLoS Negl. Trop. Dis. *8*, e3145.

Grammel, M., and Hang, H.C. (2013). Chemical reporters for biological discovery. Nat. Chem. Biol. *9*, 475–484.

Heal, W.P., Jovanovic, B., Bessin, S., Wright, M.H., Magee, A.I., and Tate, E.W. (2011). Bioorthogonal chemical tagging of protein cholesterylation in living cells. Chem. Commun. (Camb.) *47*, 4081–4083.

Heal, W.P., Wright, M.H., Thinon, E., and Tate, E.W. (2012). Multifunctional protein labeling via enzymatic N-terminal tagging and elaboration by click chemistry. Nat. Protoc. *7*, 105–117.

Hertz-Fowler, C., Ersfeld, K., and Gull, K. (2001). CAP5.5, a life-cycle-regulated, cytoskeleton-associated protein is a member of a novel family of calpain-related proteins in *Trypanosoma brucei*. Mol. Biochem. Parasitol. *116*, 25–34.

Hutton, J.A., Goncalves, V., Brannigan, J.A., Paape, D., Wright, M.H., Waugh, T.M., Roberts, S.M., Bell, A.S., Wilkinson, A.J., Smith, D.F., et al. (2014). Structure-based design of potent and selective Leishmania N-myristoyltransferase inhibitors. J. Med. Chem. *57*, 8664–8670.

Jennings, B.C., and Linder, M.E. (2012). DHHC protein S-acyltransferases use similar ping-pong kinetic mechanisms but display different acyl-CoA specificities. J. Biol. Chem. *287*, 7236–7245.

Jiang, H., Khan, S., Wang, Y., Charron, G., He, B., Sebastian, C., Du, J., Kim, R., Ge, E., Mostoslavsky, R., et al. (2013). SIRT6 regulates TNF-alpha secretion through hydrolysis of long-chain fatty acyl lysine. Nature *496*, 110–113.

Kimura, A., Kato, Y., and Hirano, H. (2012). N-Myristoylation of the Rpt2 subunit regulates intracellular localization of the yeast 26S proteasome. Biochemistry *51*, 8856–8866.

Li, H., van der Linden, W.A., Verdoes, M., Florea, B.I., McAllister, F.E., Govindaswamy, K., Elias, J.E., Bhanot, P., Overkleeft, H.S., and Bogyo, M. (2014). Assessing subunit dependency of the *Plasmodium* proteasome using small molecule inhibitors and active site probes. ACS Chem. Biol. *9*, 1869–1876.

Lin, H., Su, X., and He, B. (2012). Protein lysine acylation and cysteine succination by intermediates of energy metabolism. ACS Chem. Biol. *7*, 947–960.

Ma, B., and Johnson, R. (2011). De novo sequencing and homology searching. Mol. Cell. Proteomics *11*, O111 014902.

Maclean, L.M., O'Toole, P.J., Stark, M., Marrison, J., Seelenmeyer, C., Nickel, W., and Smith, D.F. (2012). Trafficking and release of *Leishmania* metacyclic HASPB on macrophage invasion. Cell. Microbiol. *14*, 740–761.

Maurer-Stroh, S., Eisenhaber, B., and Eisenhaber, F. (2002). N-Terminal N-myristoylation of proteins: prediction of substrate proteins from amino acid sequence. J. Mol. Biol. *317*, 541–557.

McConville, M.J., and Blackwell, J.M. (1991). Developmental changes in the glycosylated phosphatidylinositols of *Leishmania donovani*. Characterization of the promastigote and amastigote glycolipids. J. Biol. Chem. *266*, 15170–15179.

Mills, E., Price, H.P., Johner, A., Emerson, J.E., and Smith, D.F. (2007). Kinetoplastid PPEF phosphatases: dual acylated proteins expressed in the endomembrane system of *Leishmania*. Mol. Biochem. Parasitol. *152*, 22–34.

Olaleye, T.O., Brannigan, J.A., Roberts, S.M., Leatherbarrow, R.J., Wilkinson, A.J., and Tate, E.W (2014). Peptidomimetic inhibitors of N-myristoyltransferase from human malaria and leishmaniasis parasites. Org. Biomol. Chem. *12*, 8132–8137.

Paape, D., Bell, A.S., Heal, W.P., Hutton, J.A., Leatherbarrow, R.J., Tate, E.W., and Smith, D.F. (2014). Using a non-image-based medium-throughput assay for screening compounds targeting N-myristoylation in intracellular *Leishmania* amastigotes. PLoS Negl. Trop. Dis. *8*, e3363.

Price, H.P., Menon, M.R., Panethymitaki, C., Goulding, D., McKean, P.G., and Smith, D.F. (2003). Myristoyl-CoA:protein N-myristoyltransferase, an essential enzyme and potential drug target in kinetoplastid parasites. J. Biol. Chem. *278*, 7206–7214.

Price, H.P., Panethymitaki, C., Goulding, D., and Smith, D.F. (2005). Functional analysis of TbARL1, an N-myristoylated Golgi protein essential for viability in bloodstream trypanosomes. J. Cell Sci. *118*, 831–841.

Price, H.P., Hodgkinson, M.R., Wright, M.H., Tate, E.W., Smith, B.A., Carrington, M., Stark, M., and Smith, D.F. (2012). A role for the vesicle-associated tubulin binding protein ARL6 (BBS3) in flagellum extension in *Trypanosoma brucei*. Biochim. Biophys. Acta *1823*, 1178–1191.

Price, H.P., Paape, D., Hodgkinson, M.R., Farrant, K., Doehl, J., Stark, M., and Smith, D.F. (2013). The *Leishmania major* BBSome subunit BBS1 is essential for parasite virulence in the mammalian host. Mol. Microbiol. *90*, 597–611.

Ralton, J.E., Mullin, K.A., and McConville, M.J. (2002). Intracellular trafficking of glycosylphosphatidylinositol (GPI)-anchored proteins and free GPIs in *Leishmania mexicana*. Biochem. J. *363*, 365–375.

Rosenzweig, D., Smith, D., Opperdoes, F., Stern, S., Olafson, R.W., and Zilberstein, D. (2008). Retooling *Leishmania* metabolism: from sand fly gut to human macrophage. FASEB J. *22*, 590–602.

Sadlova, J., Price, H.P., Smith, B.A., Votypka, J., Volf, P., and Smith, D.F. (2010). The stage-regulated HASPB and SHERP proteins are essential for differentiation of the protozoan parasite Leishmania major in its sand fly vector, *Phlebotomus papatasi*. Cell. Microbiol. *12*, 1765–1779.

Sahin, A., Espiau, B., Tetaud, E., Cuvillier, A., Lartigue, L., Ambit, A., Robinson, D.R., and Merlin, G. (2008). The leishmania ARL-1 and Golgi traffic. PLoS One *3*, e1620.

Smelt, S.C., Engwerda, C.R., McCrossen, M., and Kaye, P.M. (1997). Destruction of follicular dendritic cells during chronic visceral leishmaniasis. J. Immunol. *158*, 3813–3821.

St-Denis, A., Caouras, V., Gervais, F., and Descoteaux, A. (1999). Role of protein kinase C-alpha in the control of infection by intracellular pathogens in macrophages. J. Immunol. *163*, 5505–5511.

Tate, E.W., Bell, A.S., Rackham, M.D., and Wright, M.H. (2014). N-Myristoyltransferase as a potential drug target in malaria and leishmaniasis. Parasitology *141*, 37–49.

Thinon, E., Serwa, R.A., Broncel, M., Brannigan, J.A., Brassat, U., Wright, M.H., Heal, W.P., Wilkinson, A.J., Mann, D.J., and Tate, E.W. (2014). Global profiling of co- and post-translationally *N*-myristoylated proteomes in human cells. Nat. Commun. *5*, 4919.

Tull, D., Vince, J.E., Callaghan, J.M., Naderer, T., Spurck, T., McFadden, G.I., Currie, G., Ferguson, K., Bacic, A., and McConville, M.J. (2004). SMP-1, a member of a new family of small myristoylated proteins in kinetoplastid parasites, is targeted to the flagellum membrane in *Leishmania*. Mol. Biol. Cell *15*, 4775–4786.

Tull, D., Heng, J., Gooley, P.R., Naderer, T., and McConville, M.J. (2012). Acylation-dependent and-independent membrane targeting and distinct functions of small myristoylated proteins (SMPs) in *Leishmania major*. Int. J. Parasitol. *42*, 239–247.

Wagner, G.R., and Hirschey, M.D. (2014). Nonenzymatic protein acylation as a carbon stress regulated by sirtuin deacylases. Mol. Cell *54*, 5–16.

Walker, R.G., Thomson, G., Malone, K., Nowicki, M.W., Brown, E., Blake, D.G., Turner, N.J., Walkinshaw, M.D., Grant, K.M., and Mottram, J.C. (2011). High throughput screens yield small molecule inhibitors of Leishmania CRK3:CYC6 cyclin-dependent kinase. PLoS Negl. Trop. Dis. *5*, e1033.

Weinheber, N., Wolfram, M., Harbecke, D., and Aebischer, T. (1998). Phagocytosis of *Leishmania mexicana* amastigotes by macrophages leads to a sustained suppression of IL-12 production. Eur. J. Immunol. *28*, 2467–2477.

Wright, M.H., Heal, W.P., Mann, D.J., and Tate, E.W. (2010). Protein myristoylation in health and disease. J. Chem. Biol. *3*, 19–35.

Wright, M.H., Clough, B., Rackham, M.D., Rangachari, K., Brannigan, J.A., Grainger, M., Moss, D.K., Bottrill, A.R., Heal, W.P., Broncel, M., et al. (2014). Validation of *N*-myristoyltransferase as an antimalarial drug target using an integrated chemical biology approach. Nat. Chem. *6*, 112–121.

Zhang, J., Xin, L., Shan, B., Chen, W., Xie, M., Yuen, D., Zhang, W., Zhang, Z., Lajoie, G.A., and Ma, B. (2012). PEAKS DB: de novo sequencing assisted database search for sensitive and accurate peptide identification. Mol. Cell. Proteomics *11*, M111 010587.

Chemistry & Biology, Volume *22*

Supplemental Information

Global Analysis of Protein *N*-Myristoylation and Exploration of *N*-Myristoyltransferase as a Drug Target in the Neglected Human Pathogen *Leishmania donovani*

Megan H. Wright, Daniel Paape, Elisabeth M. Storck, Remigiusz A. Serwa, Deborah F. Smith, and Edward W. Tate

Supplemental Information

Table of Contents

Supplemental Figures and Tables 2
Supplemental Experimental Procedures 22
Synthesis of AzRB 22
Parasite culture (extra detail) 23
Metabolic tagging experiments (extra detail) 24
Ex vivo amastigotes inhibition assay 24
Macrophage cytotoxicity assay 24
CuAAC labeling and pull-down 24
Gel and Western blot analysis 25
Proteomics experiments 25
Bioinformatics 28
Supplemental References 29

Supplemental Figures and Tables

Supplemental Figures

Figure S1: Additional gel-based data and controls, related to Figures 1 & 2. Time-dependent labeling; competition with myristic acid; sensitivity of labeling to cycloheximide treatment; hydroxylamine treatment of lysates; YnMyr tagging of GP63; YnMyr tagging in *L. major* 18AA-HASPB-GFP strains; structures of AzTB and YnTB.

Figure S2: Proteomics data related to Figure 3. Volcano plots of YnMyr proteomics (t-test); prediction of *S*-palmitoylation for non-MG hits in amastigotes and promastigotes.

Figure S3: Inhibition full gel and blot data, including controls related to Figure 4.

Figure S4: Scatter plots to assess biological and technical reproducibility of inhibition proteomics experiments related to Figure 5.

Figure S5: Inhibitor proteomics additional data related to Figures 5 & 6.

Figure S6: YnMyr modified peptide, assigned spectra related to Figure 6 and Table 1.

Supplemental Tables

Supplemental Tables are provided as separate Excel spreadsheets.

Table S1: Identification of YnMyr tagged proteins in amastigotes related to Figure 3. Identification of proteins following metabolic tagging with Myr or YnMyr, CuAAC, pull-down, tryptic digest and LC-MS/MS analysis. Four replicates were performed independently, starting from the same metabolically labelled lysates (Myr and YnMyr samples). Data processing is described In Supplemental Procedures below (Data processing: YnMyr tagged proteins in amastigotes and promastigotes).

Table S2: Identification of YnMyr tagged proteins in promastigotes related to Figure 3. Identification of proteins following metabolic tagging with Myr or YnMyr, CuAAC, pull-down, tryptic digest and LC-MS/MS analysis. Three replicates were performed independently, starting from the same metabolically labelled lysates (Myr and YnMyr samples). Data processing is described In Supplemental Procedures below (Data processing: YnMyr tagged proteins in amastigotes and promastigotes).

Table S3: **Comparison of proteomics in promastigotes and amastigotes, and bioinformatic annotation of hits; related to Figure 3.** The datasets shown in Tables S1 & S2 were compared. GPI-anchor and *S*-palmitoylation prediction was carried out for hits. Data processing is described In Supplemental Procedures below (Data processing: YnMyr tagged proteins in amastigotes and promastigotes).

Table S4: **YnMyr proteomics in the presence of NMT inhibitors related to Figures 5 & 6.** Amastigotes were metabolically tagging with Myr or YnMyr in the presence of inhibitors 1 or 2 at indicated concentrations; CuAAC, pull-down, tryptic digest and LC-MS/MS analysis were performed to identify proteins. Two technical replicates (sample preparation from the lysates) of two biological replicates were carried out. Data processing is described In Supplemental Procedures below (Data processing: YnMyr tagging in the presence of NMT inhibitors (amastigotes)).

Table S5: All N-terminal glycine-containing (MG) *L. donovani* proteins; related to Figures 3, 5 & 6. Prediction of *N*-myristoylation by bioinformatic tools and cross-referencing to current study was performed for all *L. donovani* MG proteins in the database.

Table S6: Identification of YnMyr modified peptides, related to Figure 6 and Table 1. Proteomic data were further analyzed by de novo-aided sequencing to identify peptides modified by the the lipid-derived adduct (+534.3278 Da) on any amino acid at peptide N-terminus. All peptides reported are N-terminal peptides unique for the protein that they were assign to by the software.

Figure S1. Additional labeling data for *L. donovani* promastigotes. Related to Figs. 1 & 2. **A.** *Ld* promastigotes were cultured for the indicated timepoints in the presence of myristic acid (M) or YnMyr (Yn), lysed and proteins subject to CuAAC reaction and analysis by in-gel fluorescence. **B.** Promastigotes were labeled with YnMyr in the presence of varying amounts of myristic acid (Myr). **C.** YnMyr incorporation into proteins is dependent on protein synthesis, but incorporation into putative glycolipid is less affected. Promastigotes were tagged with myristic acid (-) or YnMyr in the presence of 50 µM cycloheximide (CHX) for indicated timepoints and samples processed by CuAAC. **D.** Treatment of YnMyr tagged samples with base (NaOH) or hydroxylamine (HA; pH7). C/M: precipitation with $CHCl_3$/MeOH. **E.** Analysis of YnMyr tagged samples by Western blot for GPI-anchored protein GP63 after CuAAC and pull-down. Click: sample reacted with CuAAC reagents; PD B: sample pulled-down onto streptavidin-coated beads and proteins eluted from beads by boiling. * = non-specific band, streptavidin monomer. Note that the percentage incorporation of YnMyr is clearly low, as it must compete with endogenous lipid. Numbers are molecular weight markers in kDa. **F.** Related to Fig. 2; total protein labeling in HASPB GFP-fusion strains. **G.** Structures of AzTB and YnTB.

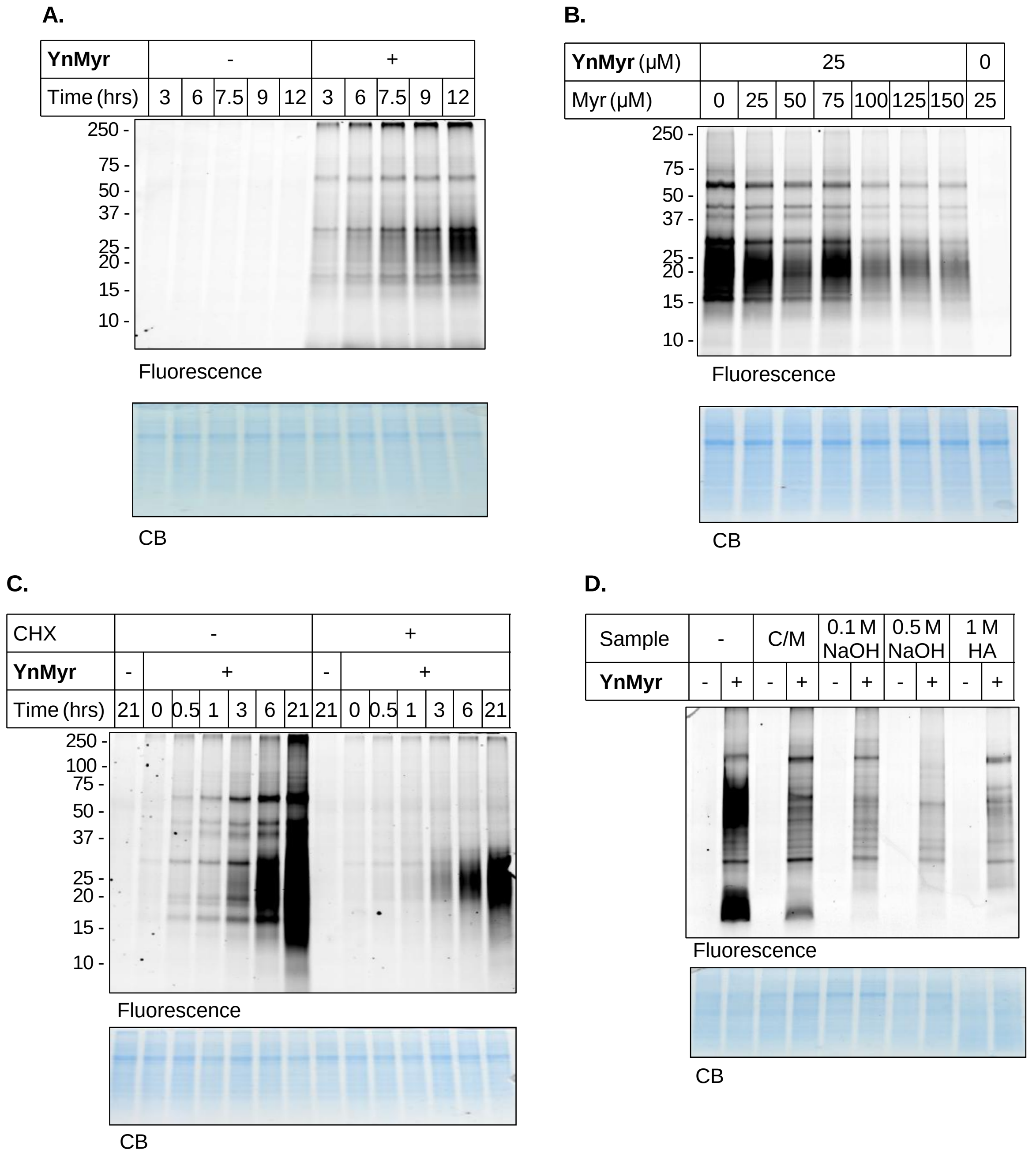

E.

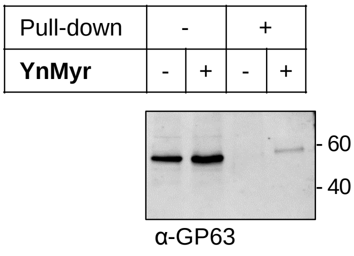

Pull-down
-
+
YnMyr
-
+
-
+
60
40
α-GP63

F.

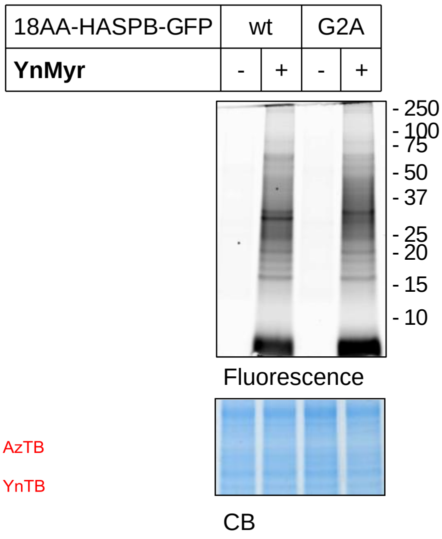

18AA-HASPB-GFP
wt
G2A
YnMyr
-
+
-
+
250
100
75
50
37
25
20
15
10
Fluorescence
CB

G.

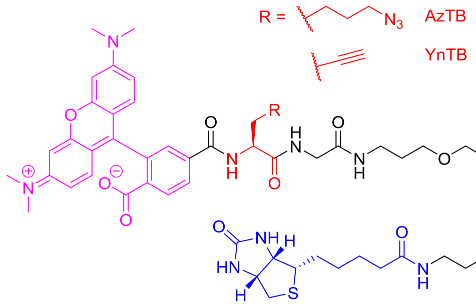

R =
AzTB
YnTB

Figure S2: YnMyr proteomics in amastigotes and promastigotes; related to Fig. 3. Permutation-corrected two-sample t-tests were applied to compare YnMyr and myristic acid (Myr) controls after imputation of missing values. An LFQ enrichment ratio was calculated from the ratio of YnMyr and Myr LFQ intensities (mean). 'High confidence': protein significant and absent from controls; 'low abundance': protein absent from myristic acid controls but did not reach statistical significance due to low intensity in the YnMyr samples; 'Other significant': protein significant but present in controls **A.** T-test in promastigotes. The 274 proteins present in all three replicates. T-test: FDR = 0.01, S0 = 1, 250 permutations. **B.** T-test in amastigotes. T-test: FDR = 0.05, S0 = 1, 250 permutations. **C.** & **D.** Volcano plots with prediction of *S*-palmitoylation using CSS-Palm (Ren et al., 2008) indicated for non-MG proteins in amastigotes and promastigotes (see also Supp. Table S3).

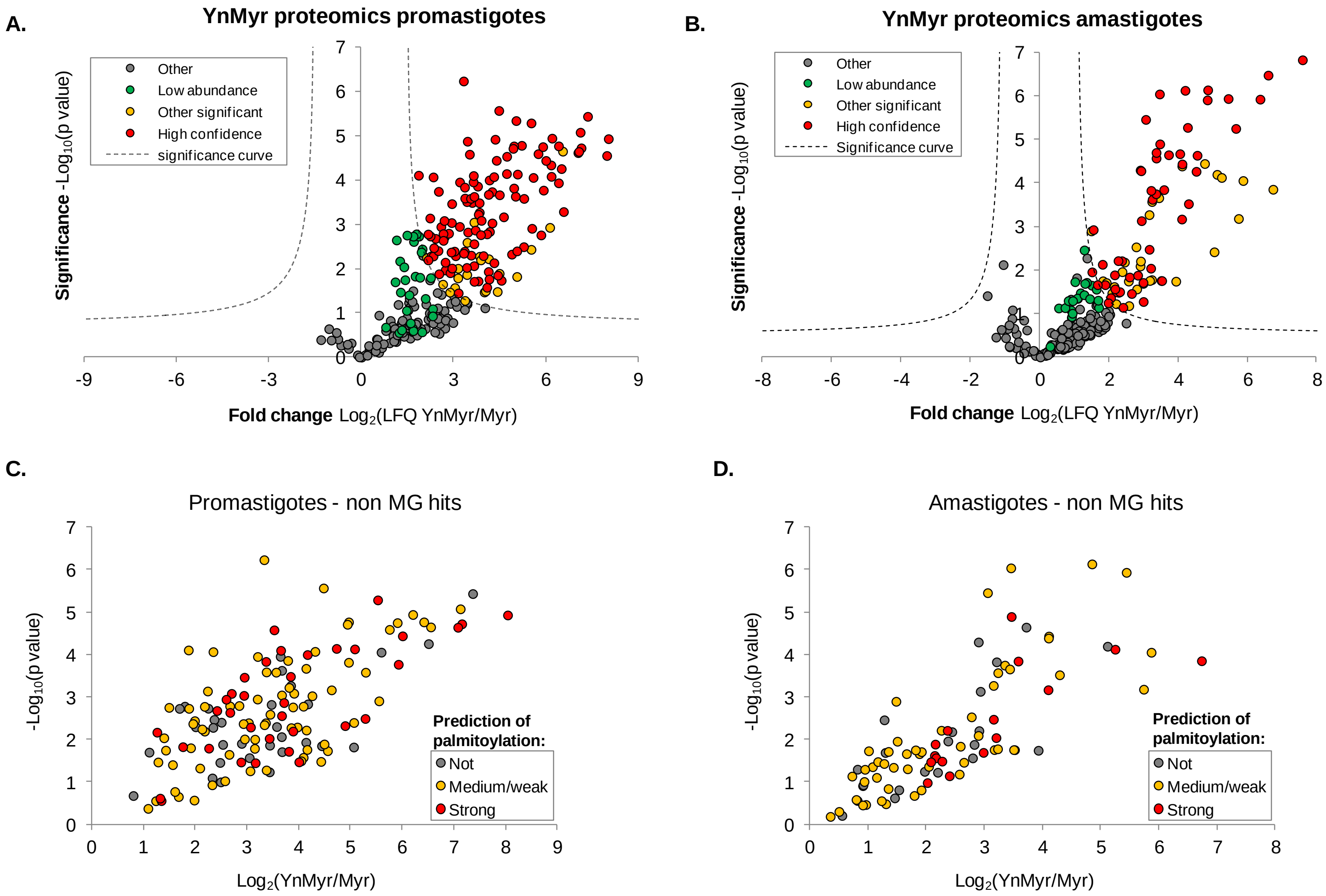

Figure S3: Related to Fig. 4; full blots and control gels for inhibition experiments. **A.** Coomassie (CB) staining for the gel shown in Fig. 4B. **B.** Western blot against HASPB (portion of image boxed with dashed line is shown in Fig. 4B) before and after pull-down of HASPB onto streptavidin beads after CuAAC in promastigotes. Four-fold more protein is loaded after pull-down compared to before. **C.** Coomassie staining for the gel shown in Fig. 4C. **D.** Western blots against HASPB before and after pull-down of HASPB in amastigotes. Four-fold more protein loaded after pull-down compared to before. Top and bottom are replicate experiments performed independently starting from the same lysate. **E.** Independent biological experiment processed in the same way as those in D. Numbers are molecular weights in kDa.

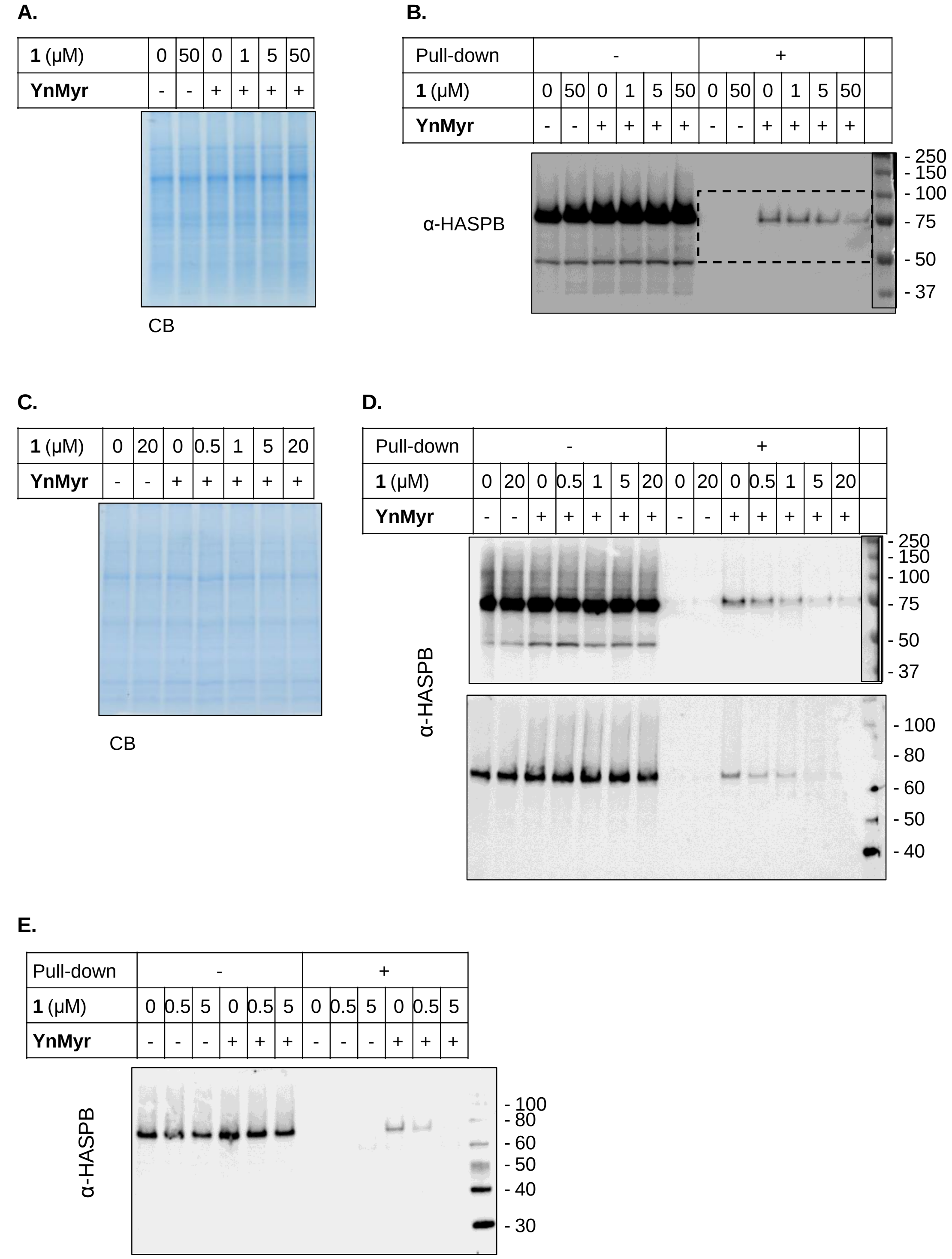

Figure S4: Related to Fig. 5. Multiscatter plots to assess biological and technical reproducibility of replicate inhibition proteomics samples. A & B: technical replicates of first biological replicate; C & D: technical replicates of second biological replicate. Dataset consists of 580 proteins identified in both independent biological experiments; Log_2(LFQ intensity) is plotted after imputation of missing values. Numbers in the top left corner of each plot indicates R^2 value (correlation); proteins containing an N-terminal glycine are coloured red, other proteins are black.

YnMyr

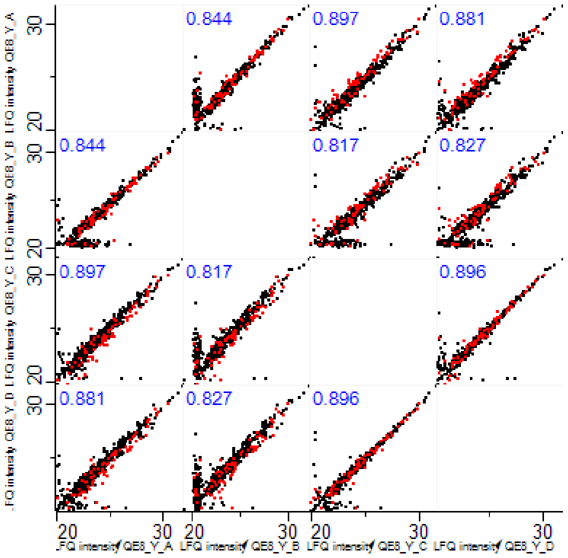

YnMyr + 0.2 µM **2**

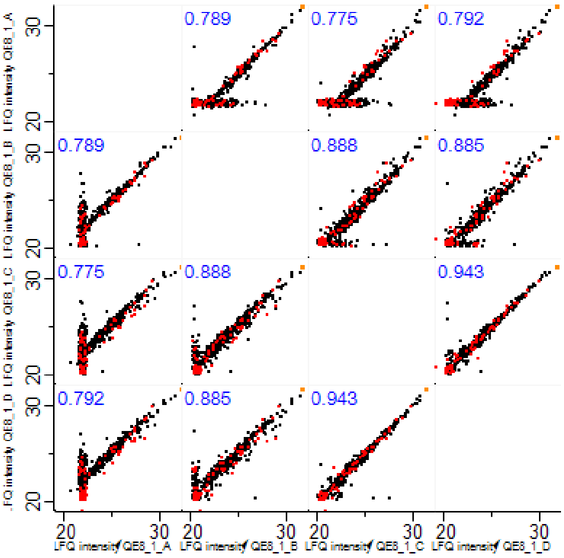

YnMyr + 0.2 µM **1**

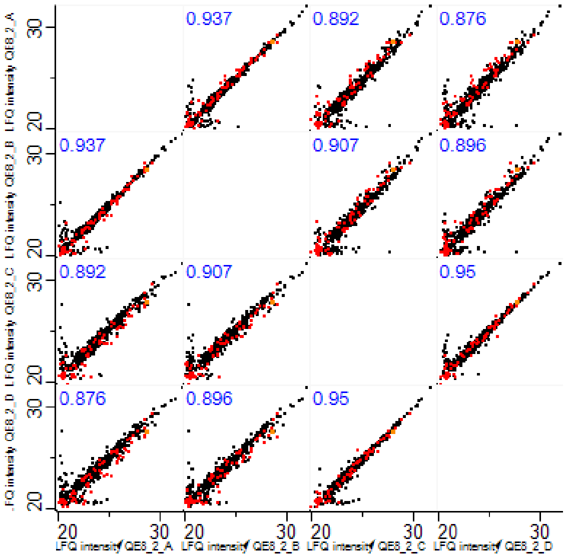

YnMyr + 7 µM **1**

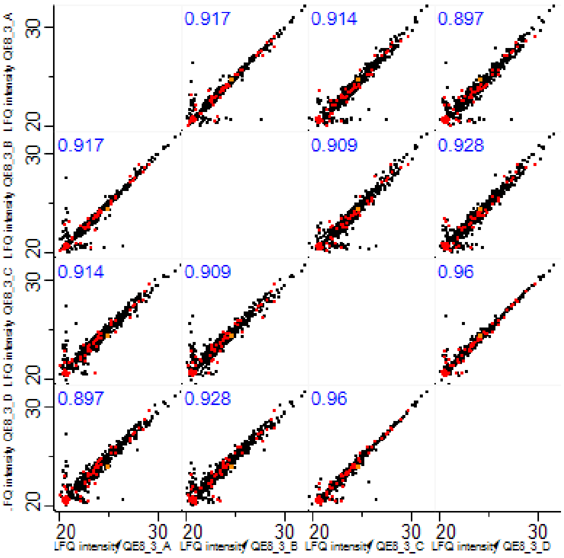

Figure S5: YnMyr inhibition proteomics; related to Figs. 5 & 6. **A.** T-test comparing 7 µM **1** (left) or 0.2 µM **1** (right) and YnMyr datasets, after removal of non-specific binders. FDR = 0.001, S0 = 1, 250 permutations. **B.** T-test comparing 0.2 µM **2** and YnMyr datasets, after removal of non-specific binders. FDR = 0.001, S0 = 1, 250 permutations. **C.** T-test comparing response to 0.2 µM **1** and 7 µM **1**: a ratio of LFQ intensities for YnMyr/(inhibitor treatment) was calculated for each replicate experiment (A-D) and a two-sample t-test (FDR 0.05, S0 1) performed to compare the response to the two inhibitors. **D.** Profile plots of the response of selected MG proteins to inhibition. Log_2(mean LFQ intensity) across four replicates. As for main text Figure 5C but including data for 0.2 µM **2**.

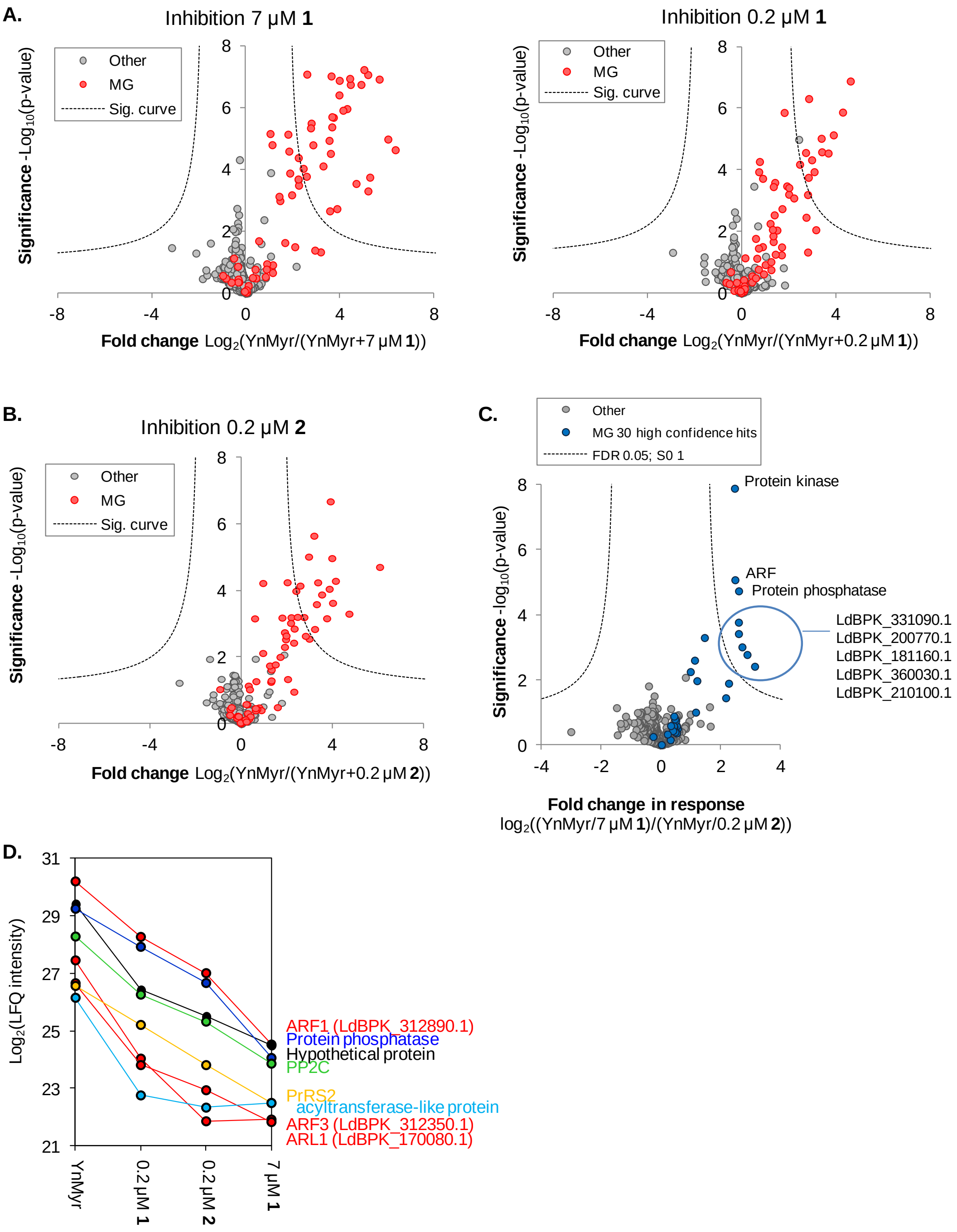

Figure S6: assigned spectra for YnMyr modified peptides. Related to Fig. 6 and Table S6.

<u>LDBPK_231190.1 (uncharacterized protein)</u>

Experiment PRO2

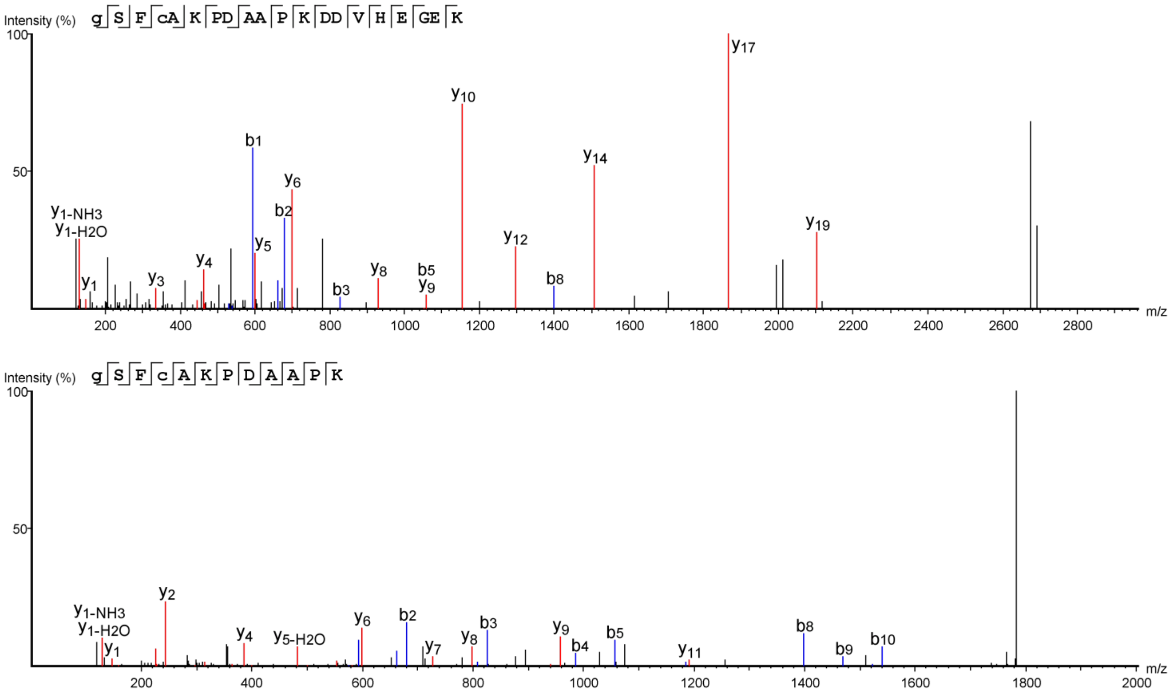

Experiment PRO3

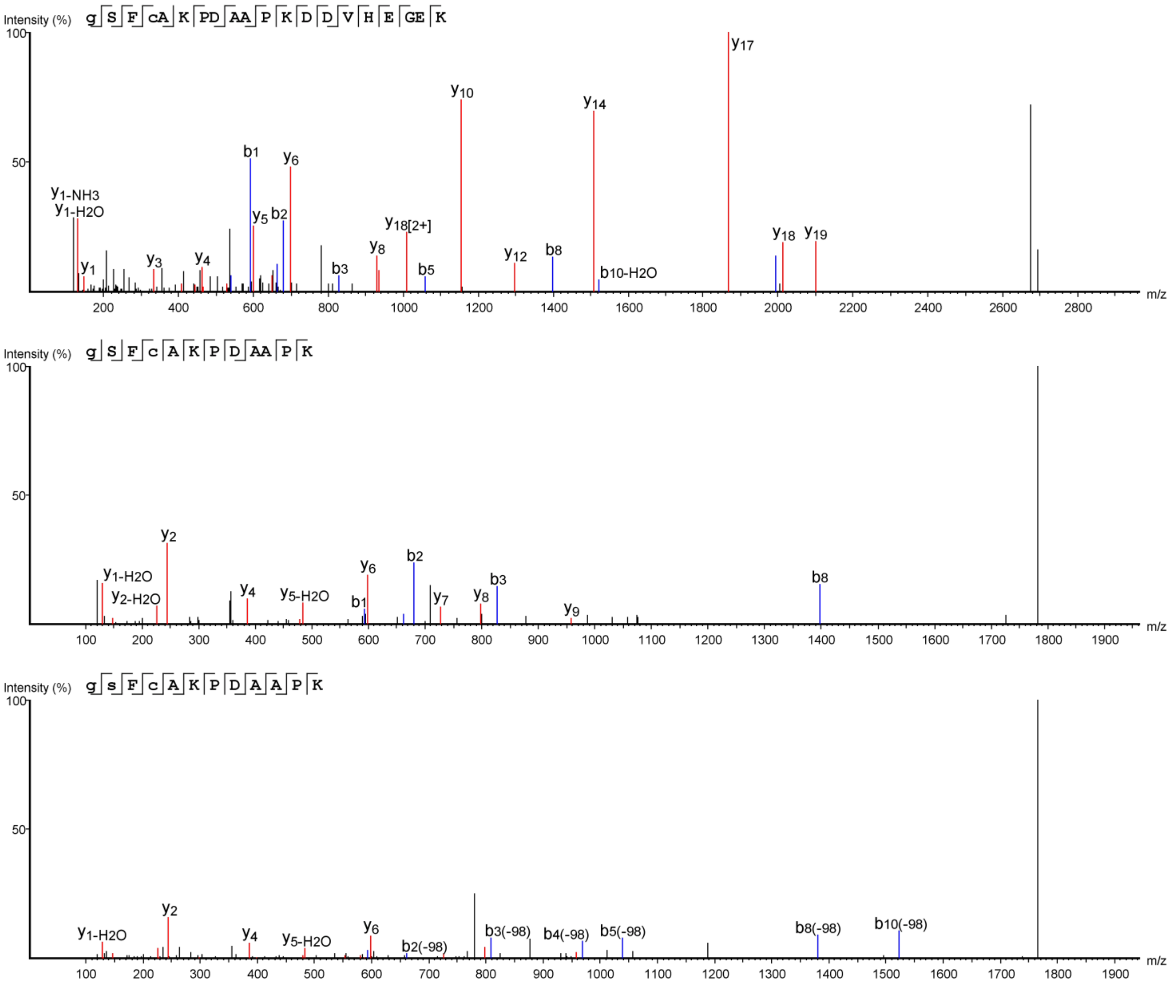

LDBPK_101370.1 (uncharacterized protein)

Experiment PRO2

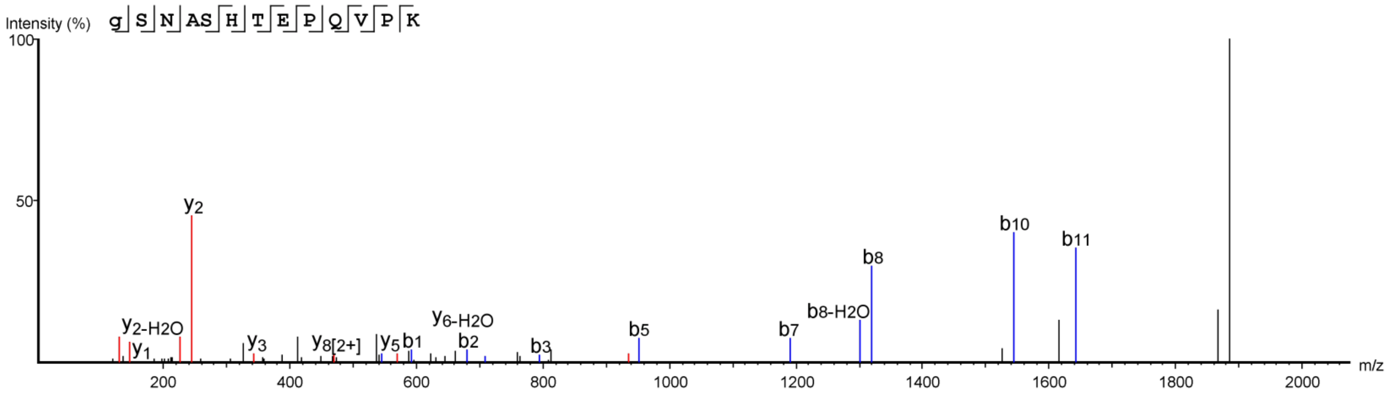

Experiment PRO3

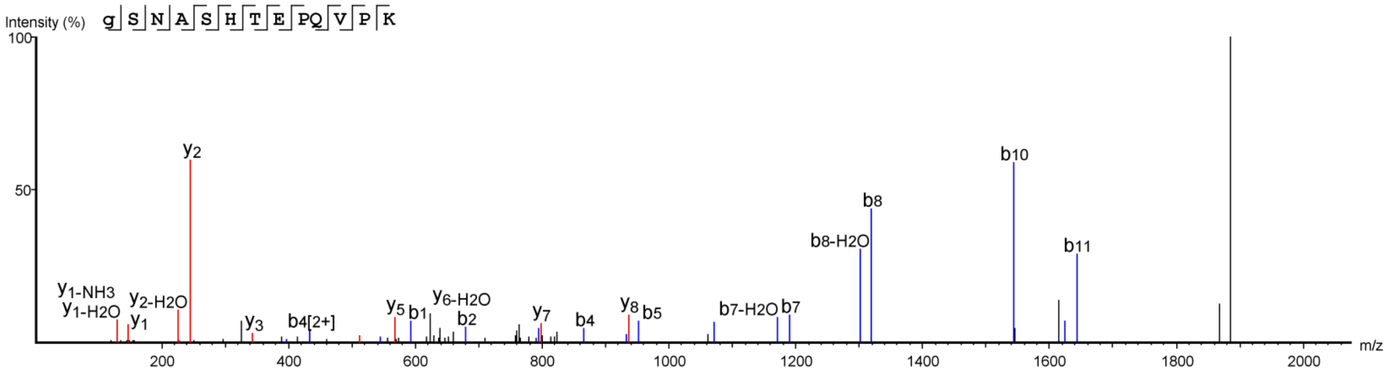

Experiment Inhib. YN_B

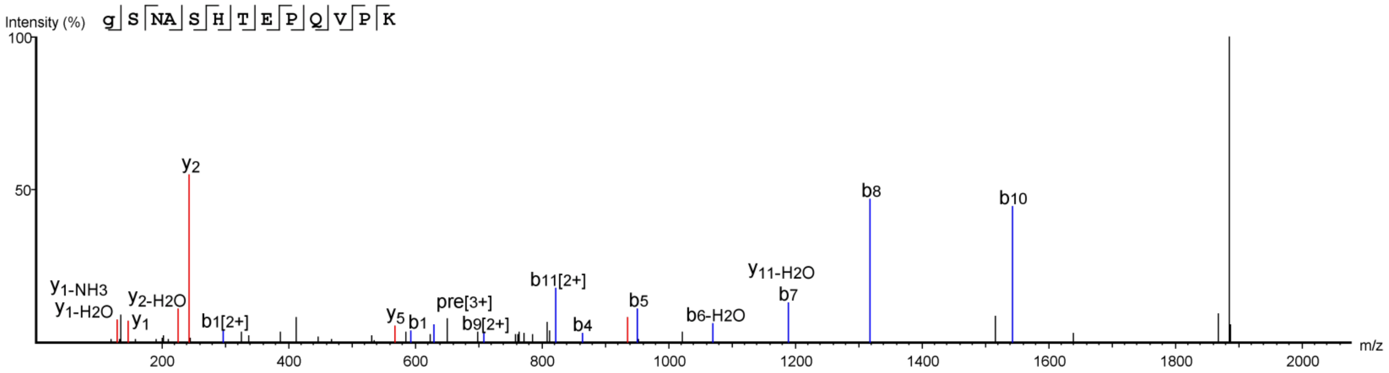

LDBPK_201260.1 (Uncharacterized protein)

Experiment PRO3

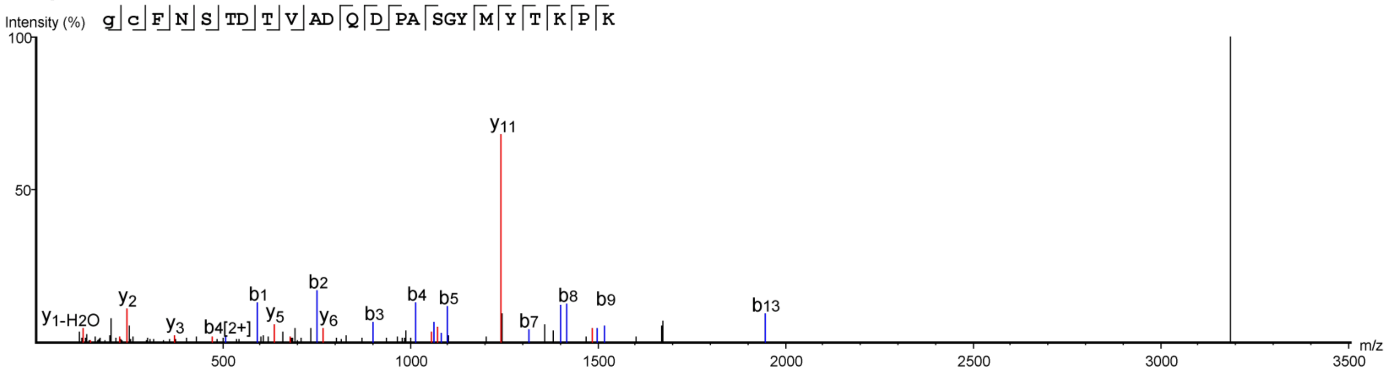

LDBPK_101380.1 (uncharacterized protein)

Experiment PRO2

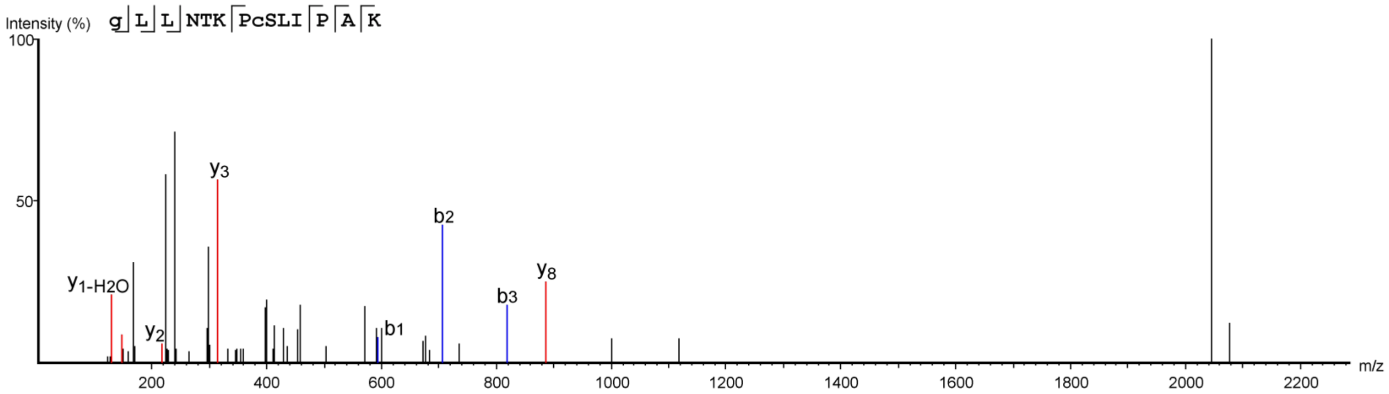

Experiment PRO3

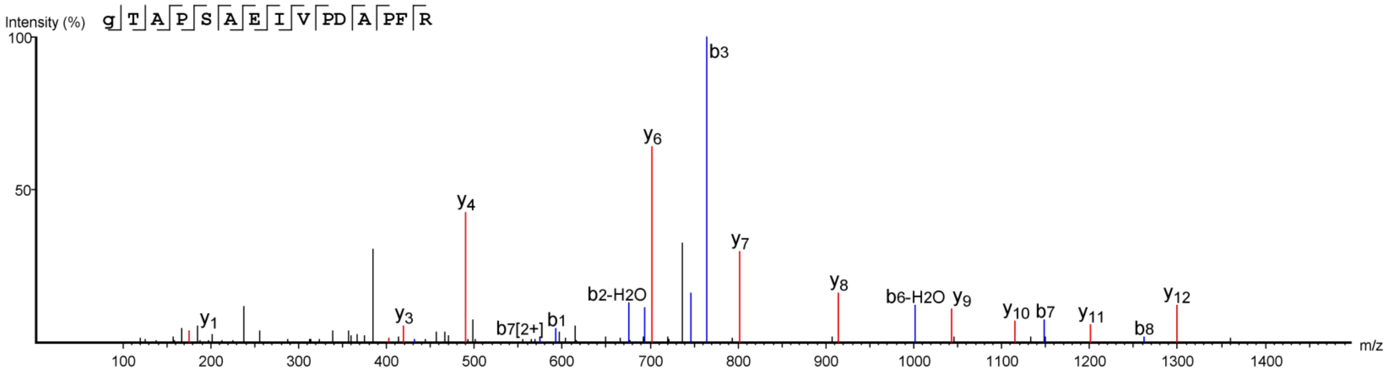

Experiment AM3

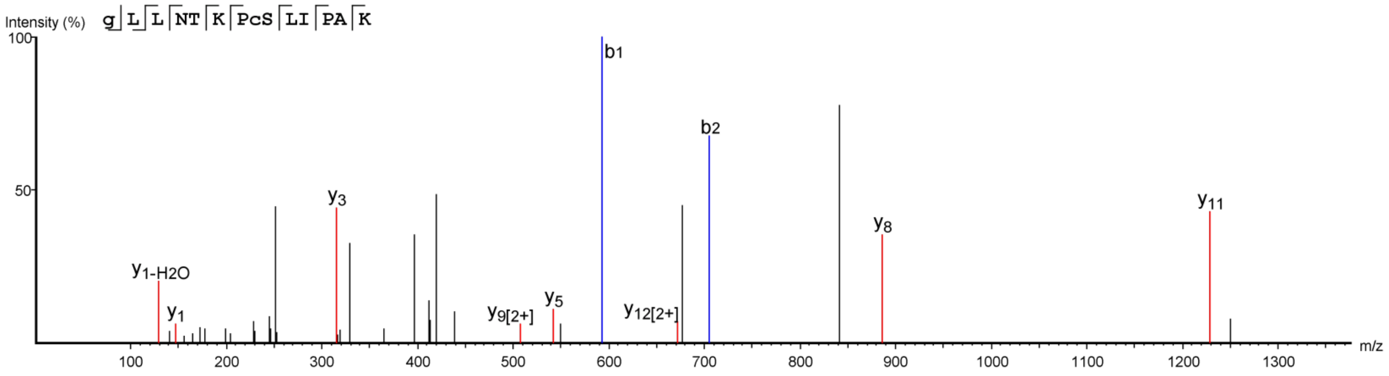

Experiment AM4

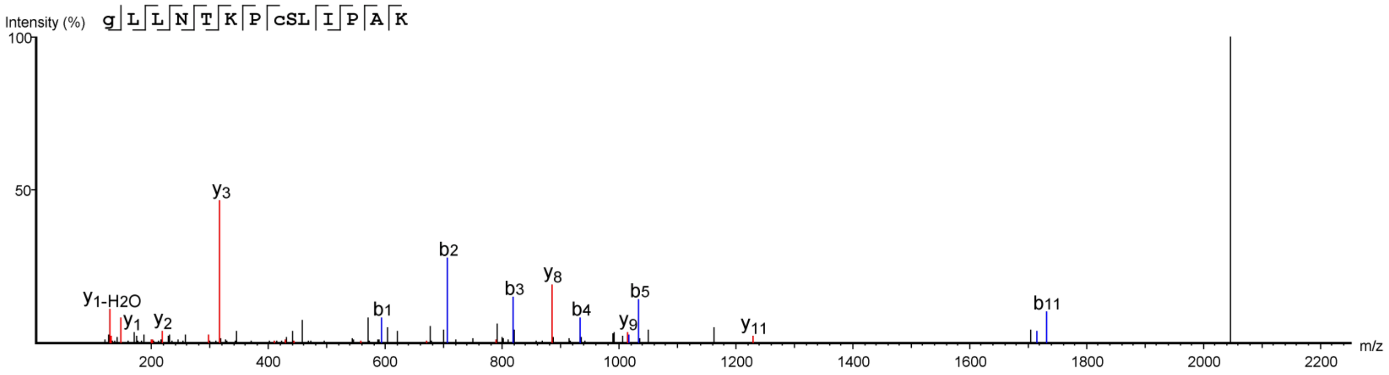

LDBPK_101380.1 (uncharacterized protein) cont.

Experiment Inhib. YN_A

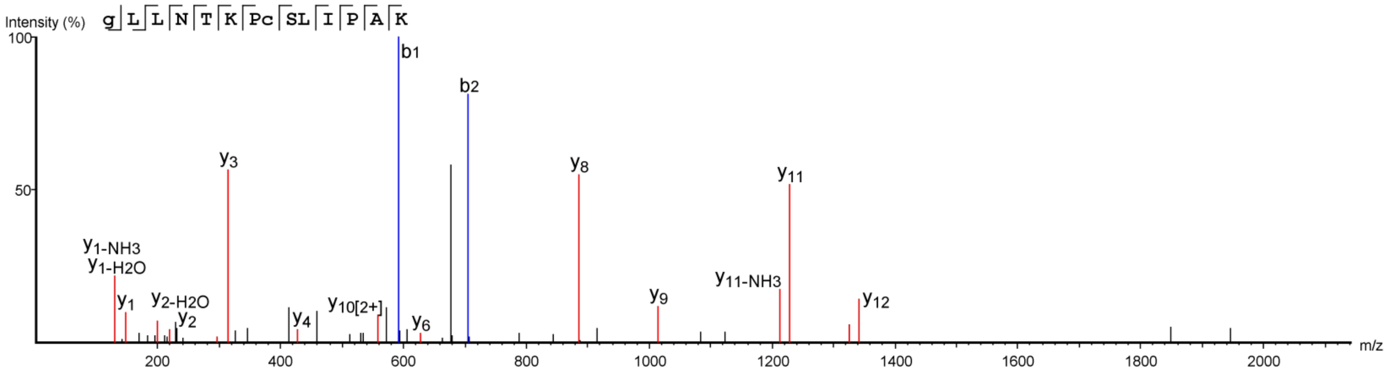

Experiment Inhib. YN_B

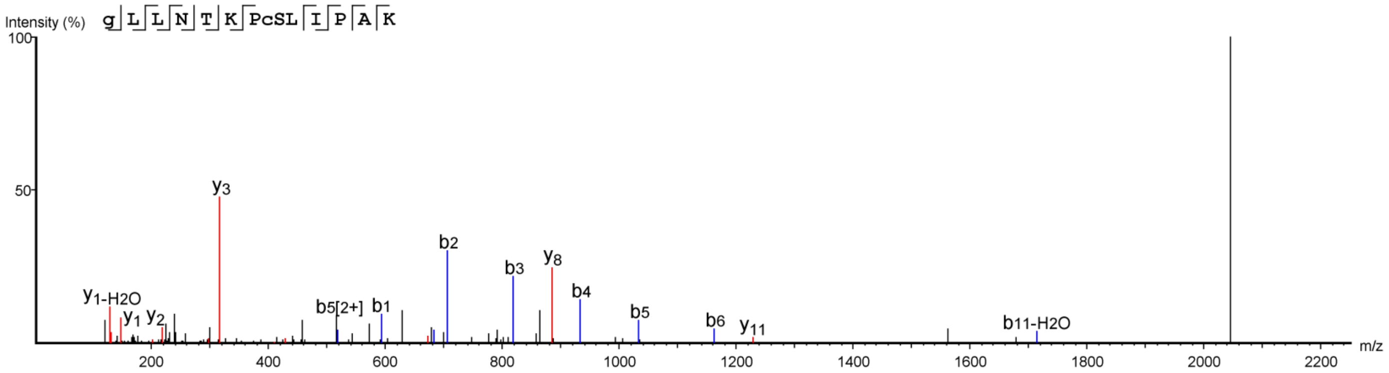

Experiment Inhib. YN_C

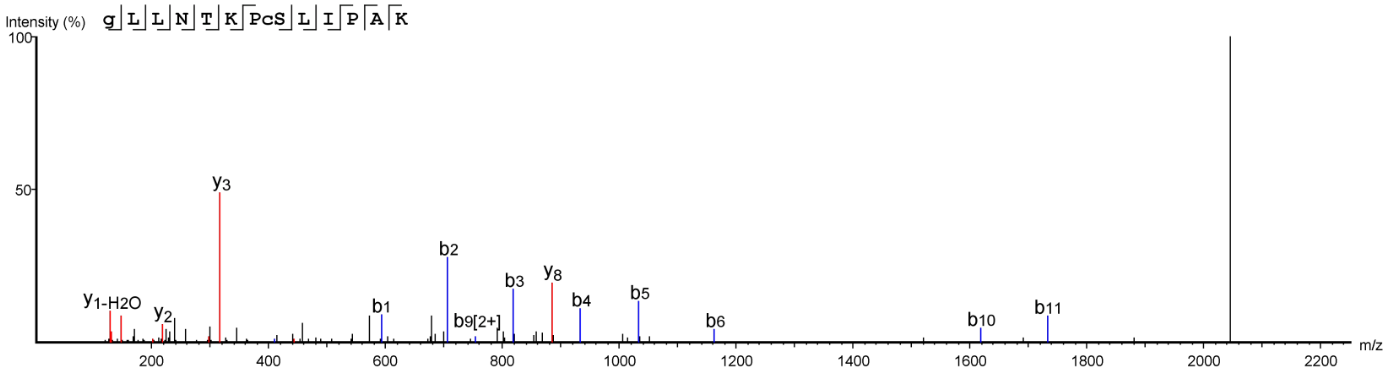

Experiment Inhib. YN_D

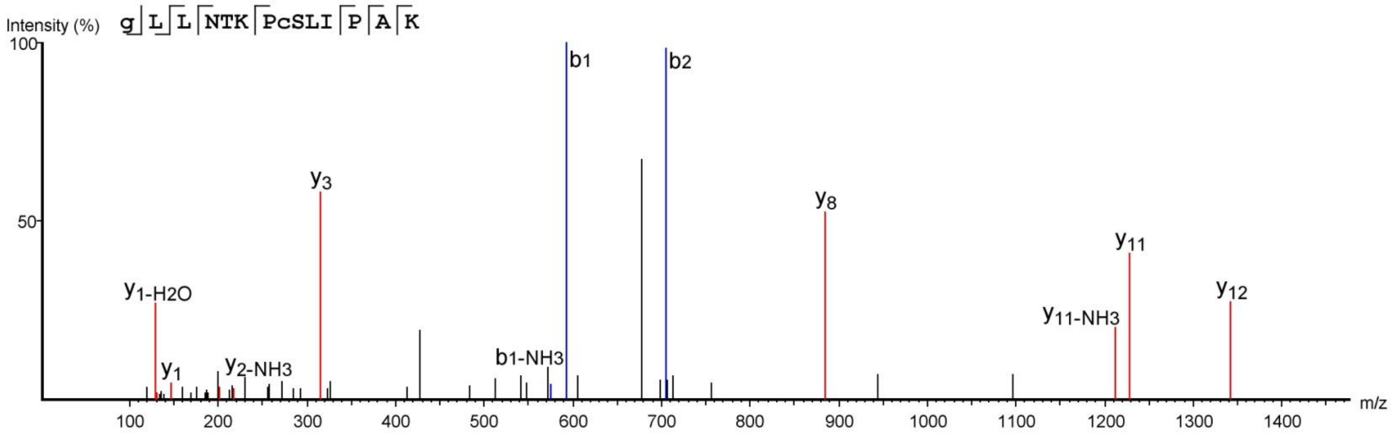

LDBPK_360560.1 (PP2C-like protein)

Experiment PRO3

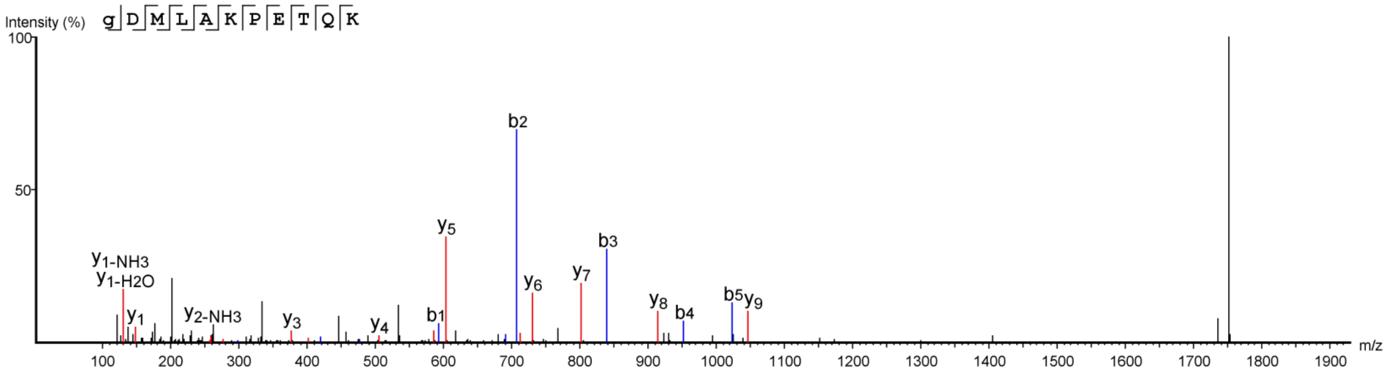

Experiment AM3

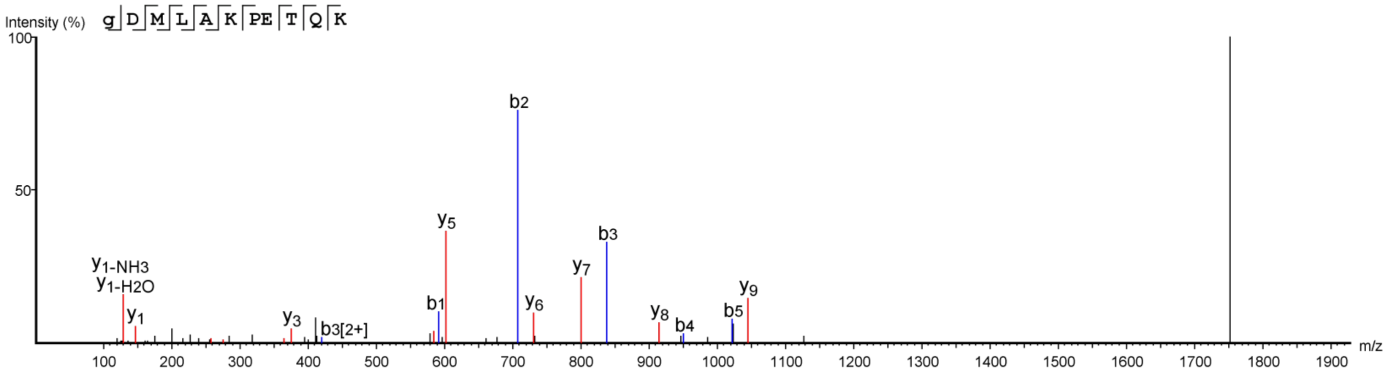

Experiment Inhib. YN_A

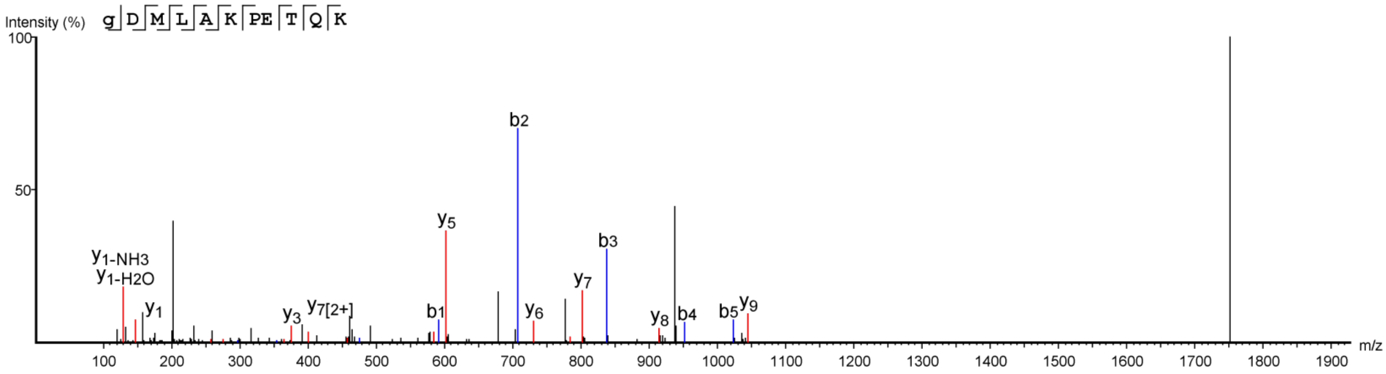

LDBPK_160930.1 (Flagellar calcium-binding protein)

Experiment PRO3

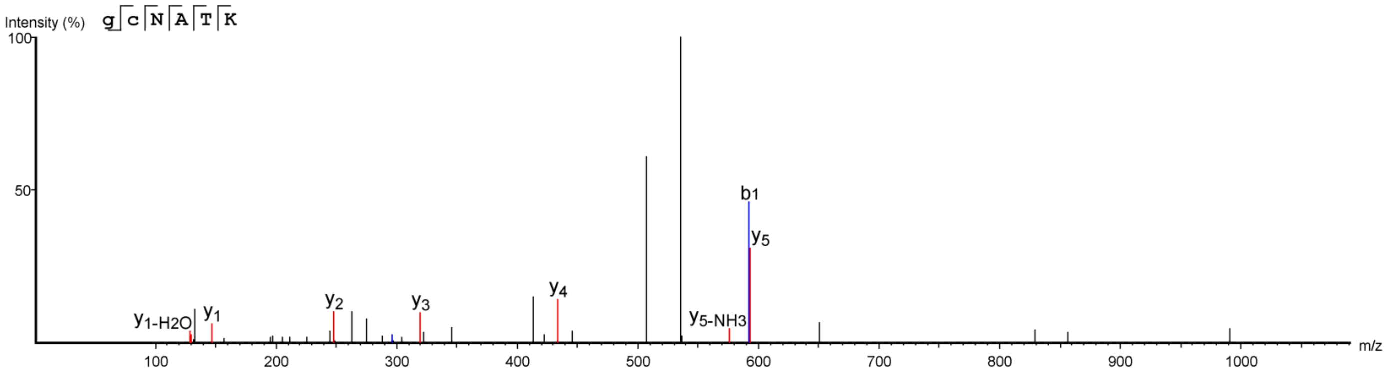

LDBPK_010510.1 (Long chain fatty acid CoA ligase)

Experiment PRO3

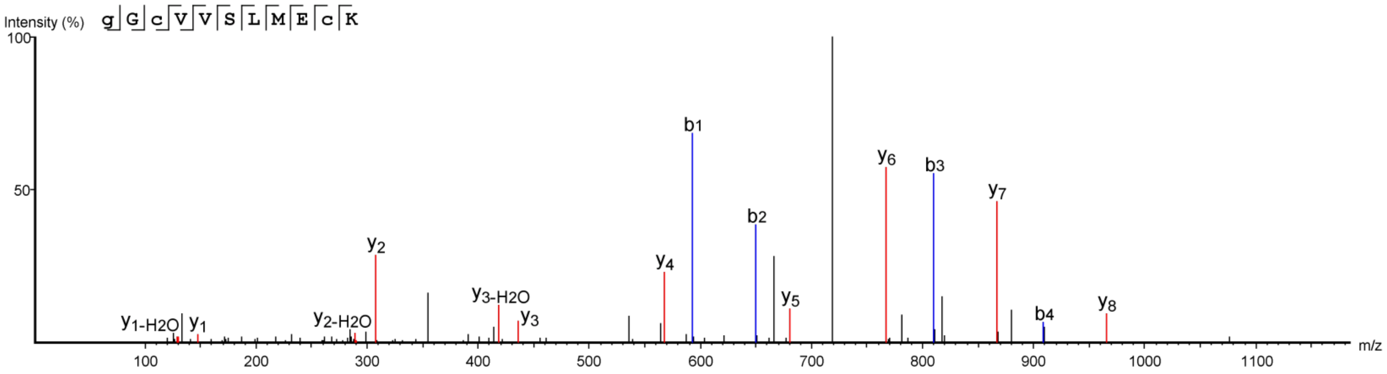

Experiment Inhib. YN_B

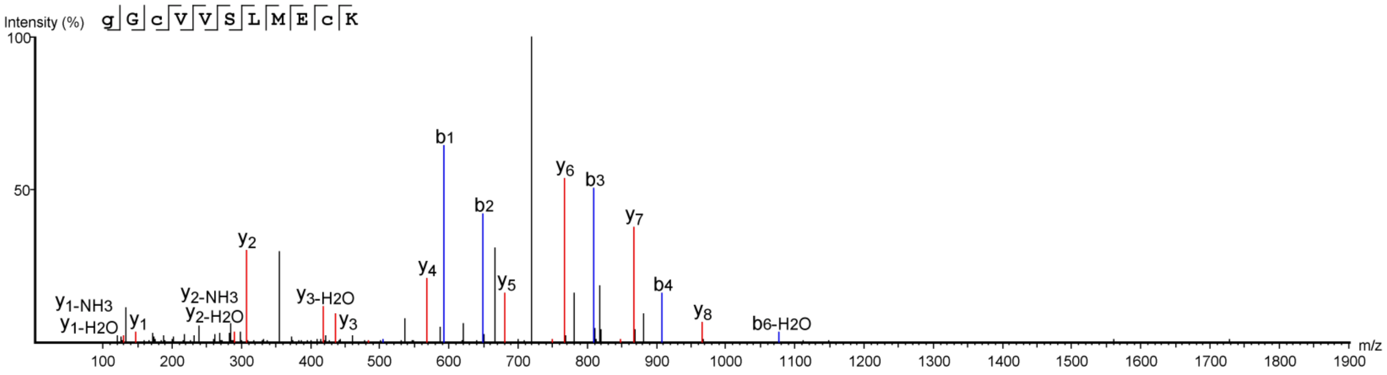

Experiment Inhib. YN_C

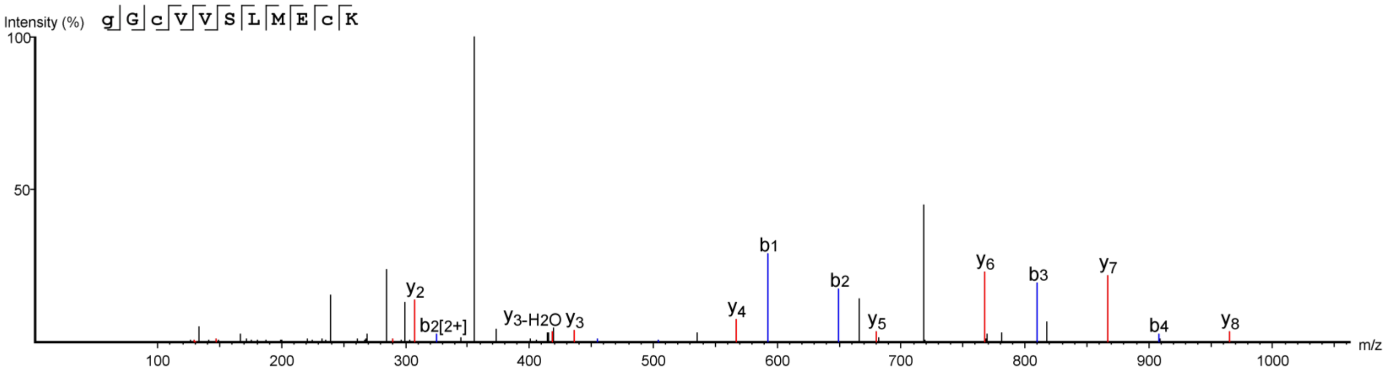

LDBPK_366730.1 (Uncharacterized protein)

Experiment Inhib. Y_A

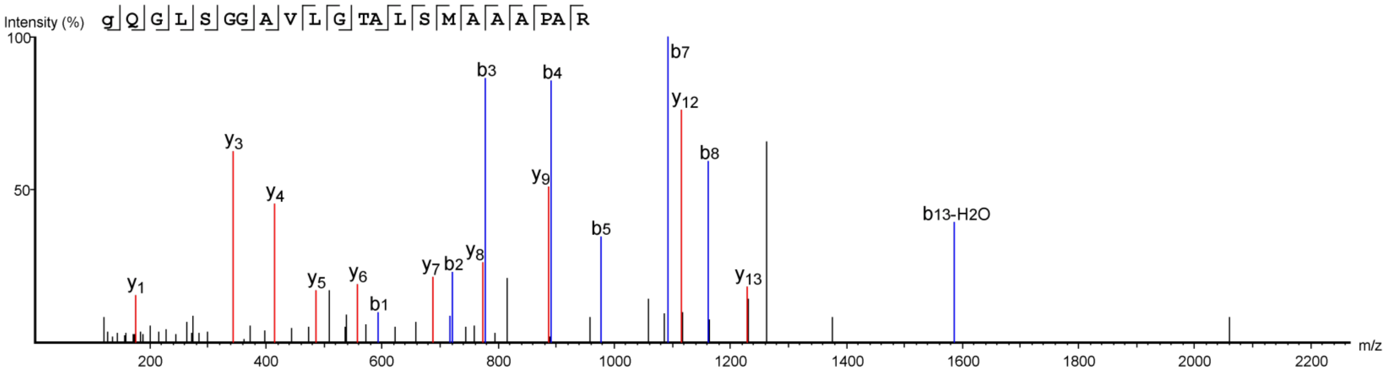

LDBPK_300680.1 (uncharacterized protein)

Experiment PRO3

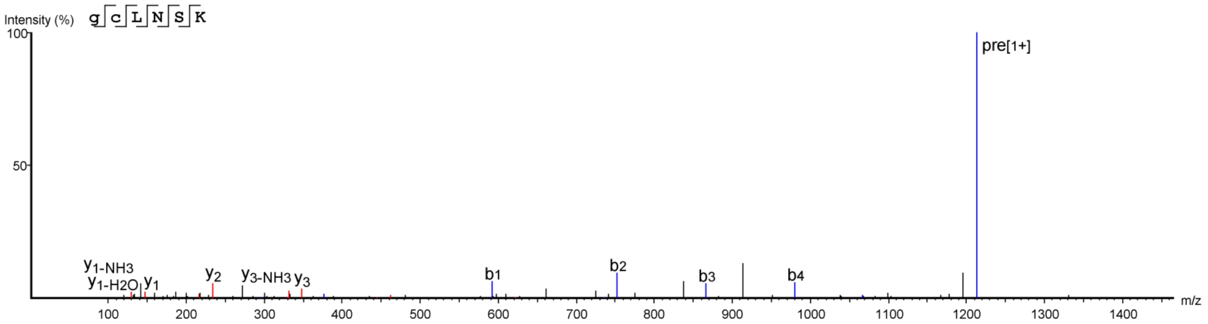

Experiment AM4

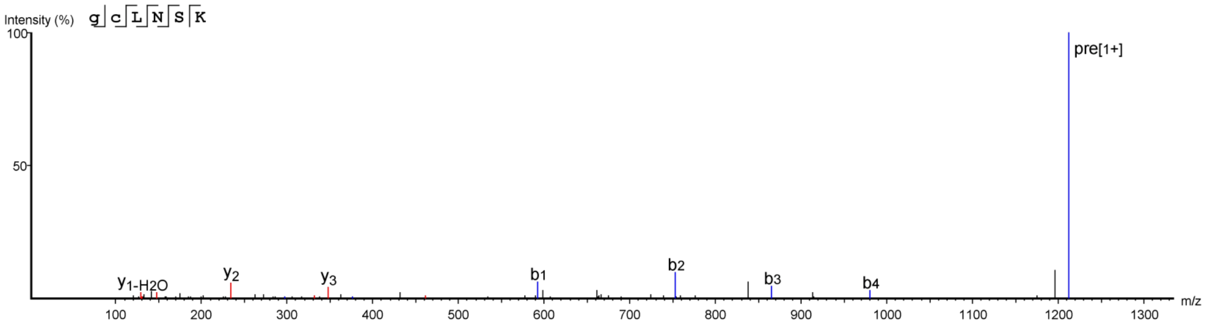

Experiment Inhib. YN_A

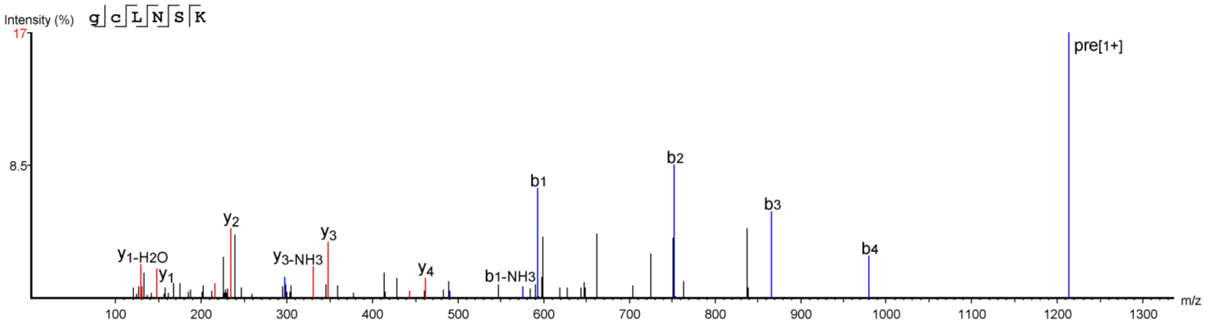

Experiment Inhib. YN_C

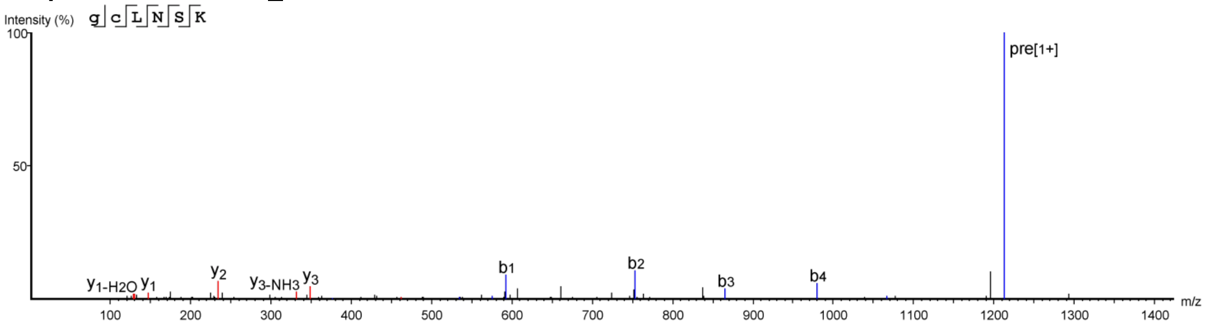

Experiment Inhib. YN_D

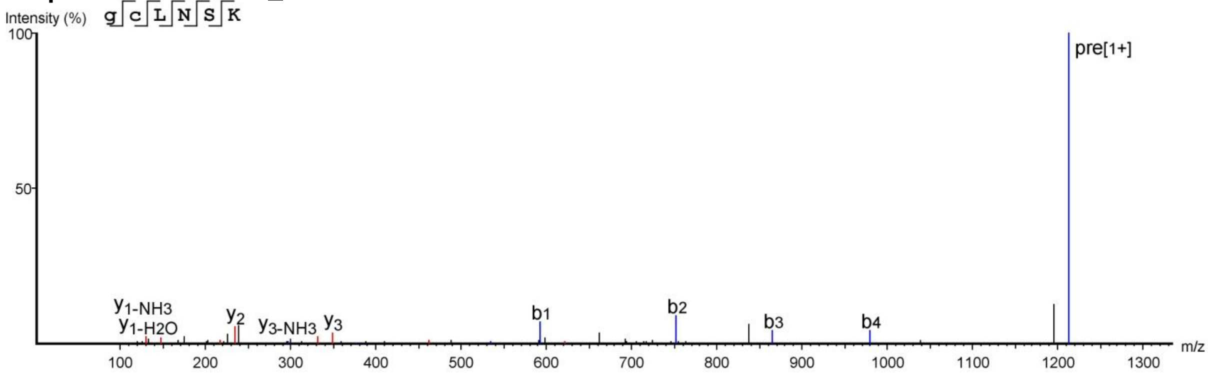

LDBPK_010540.1 (Long chain fatty acid CoA ligase)

Experiment PRO3

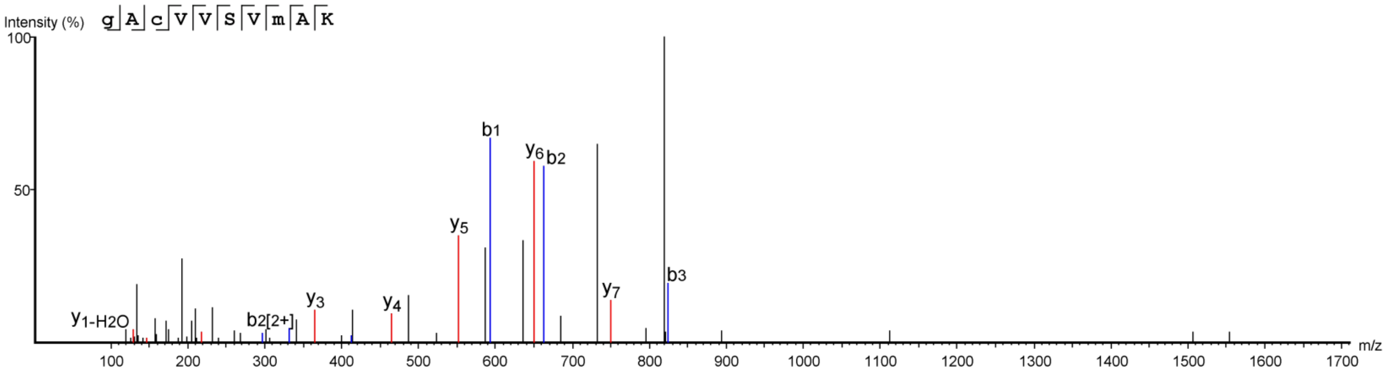

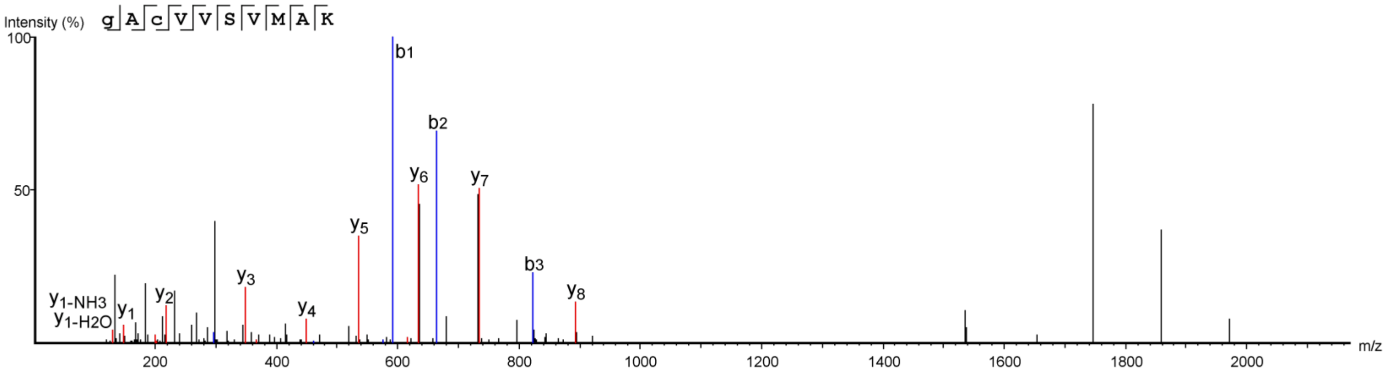

LDBPK_291980.1 (Uncharacterized protein)

Experiment PRO3

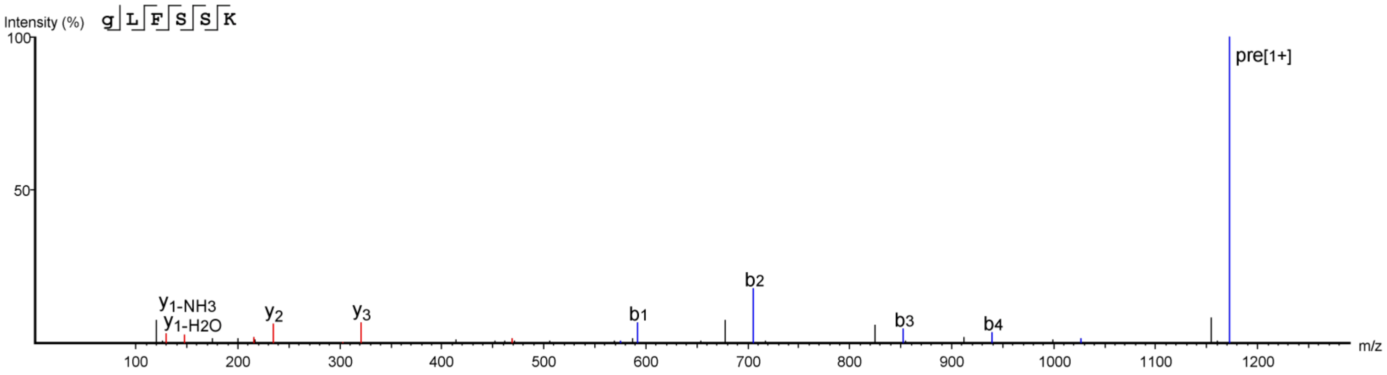

Experiment Inhib. Y_C

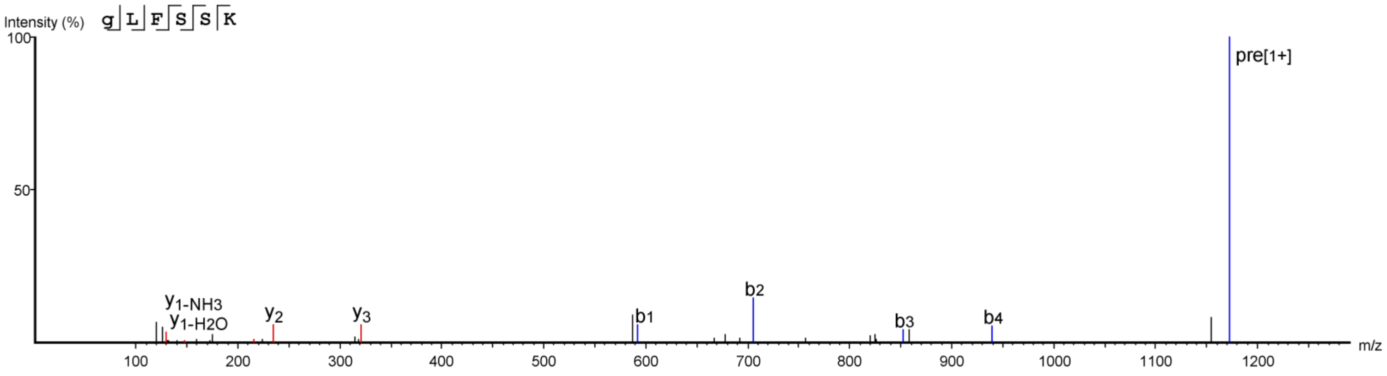

LDBPK_170080.1 (ARF)

Experiment PRO3

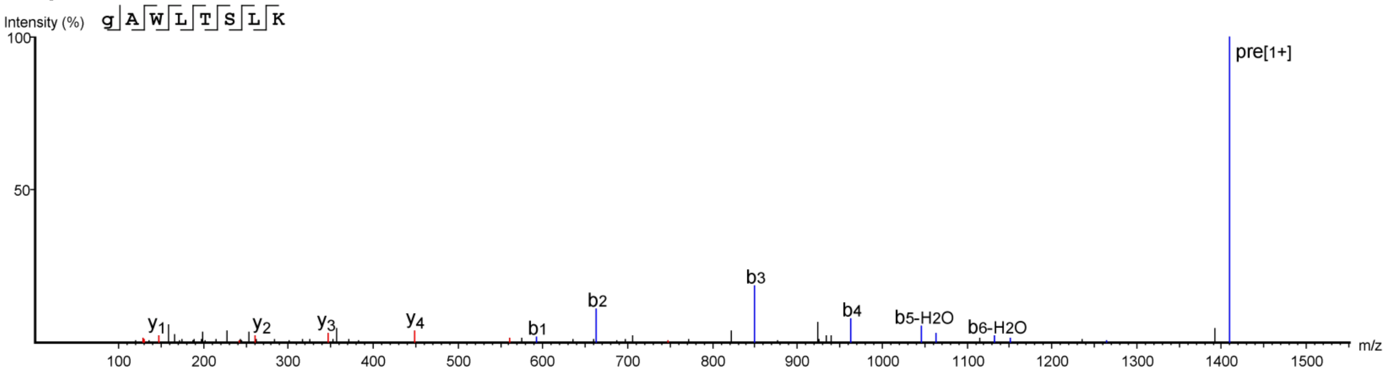

Experiment Inhib. Y_C

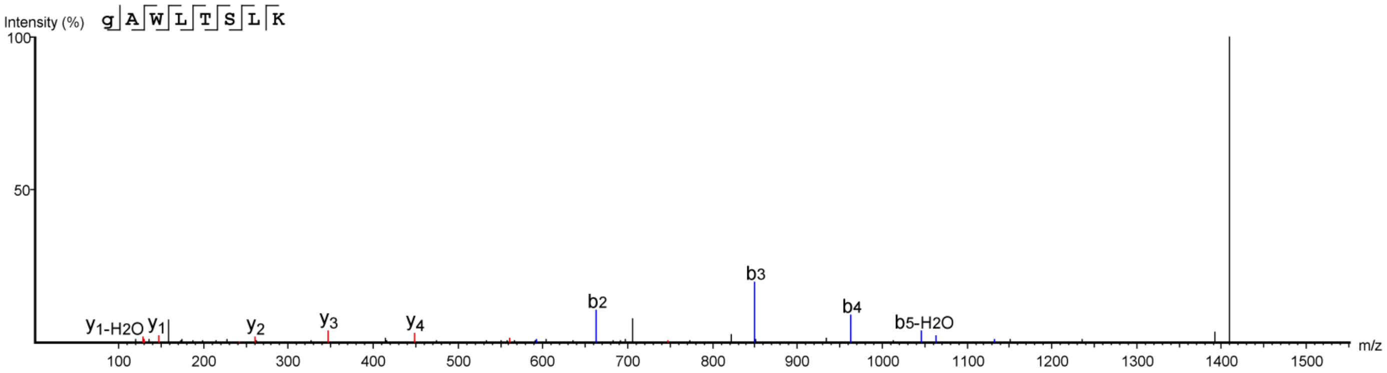

LDBPK_201350.1 (Calpain-like cysteine peptidase)

Experiment PRO3

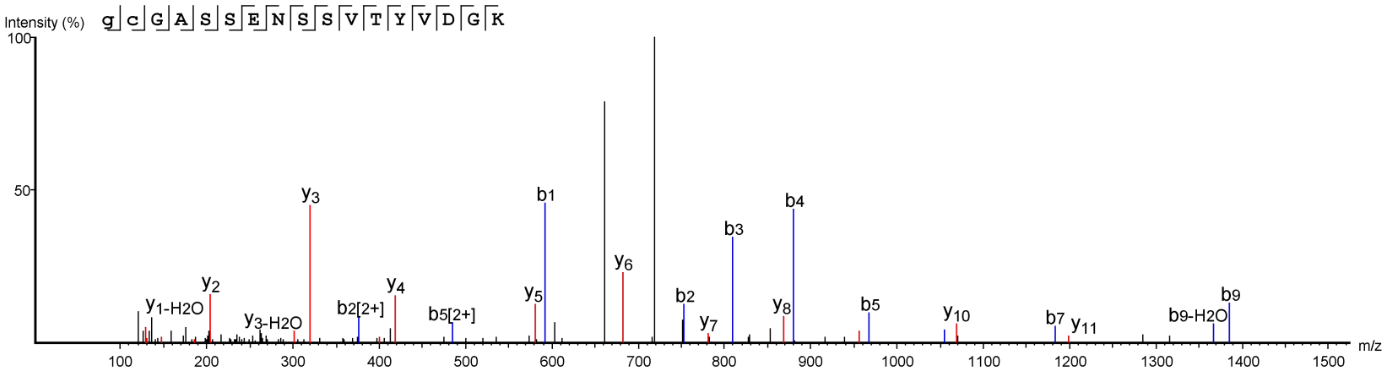

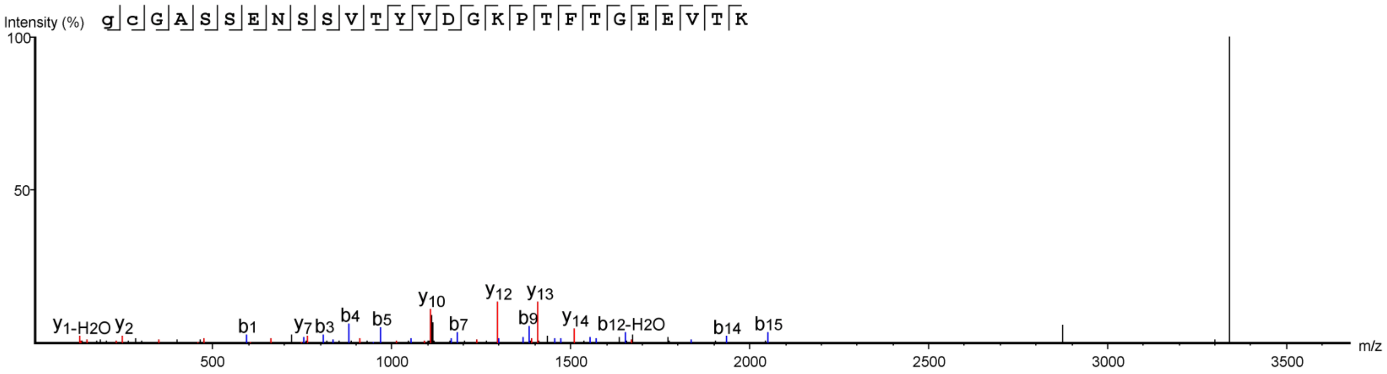

LDBPK_ 331090.1 (Uncharacterized protein)

Experiment PRO3

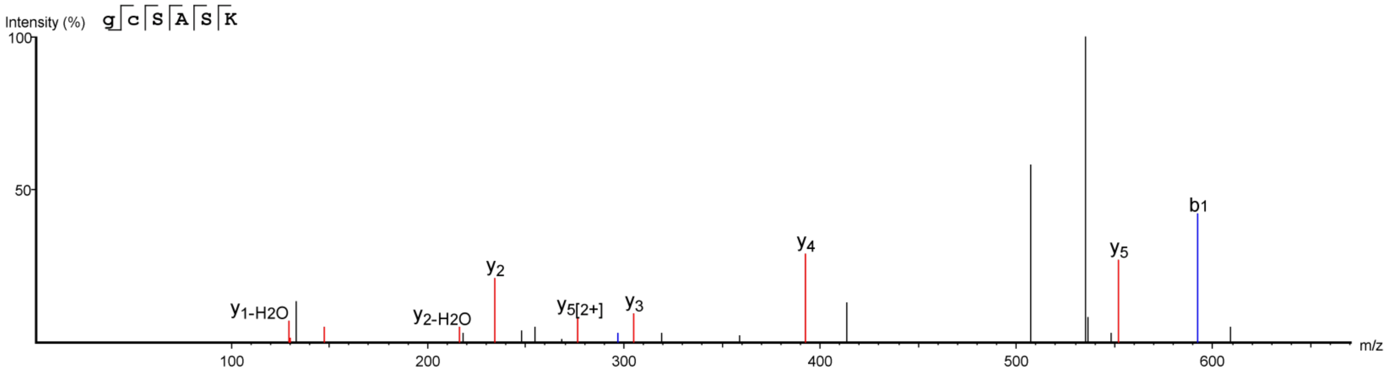

Experiment Inhib. Y_B

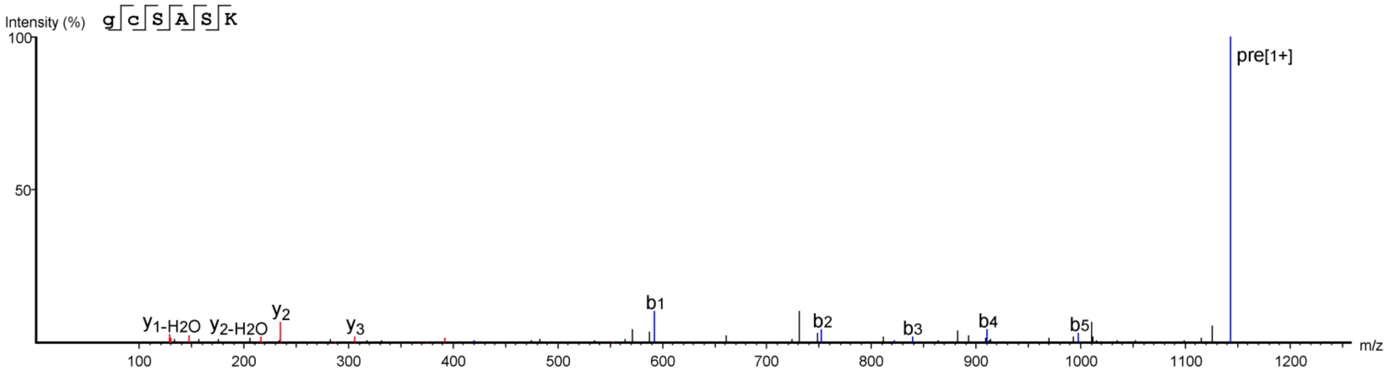

Experiment Inhib. Y_C

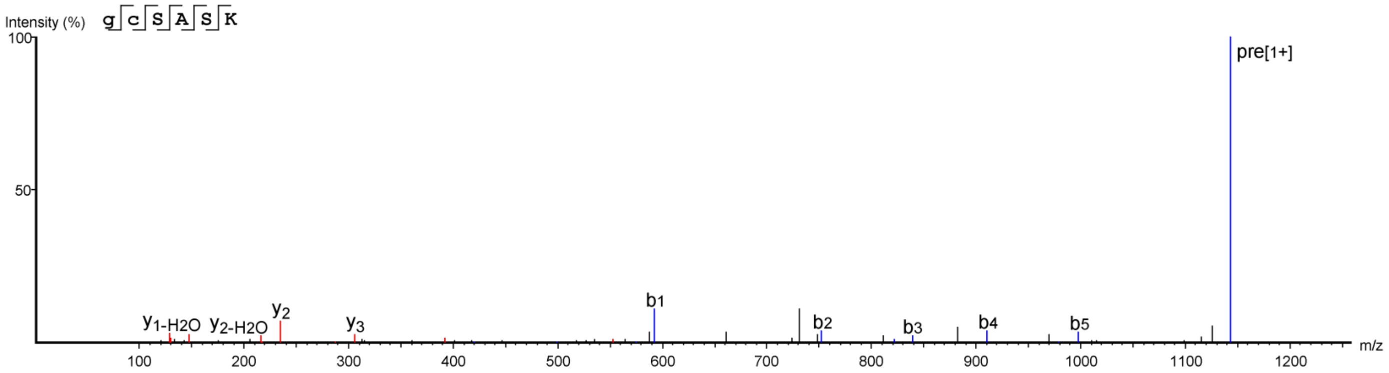

Experiment Inhib. Y_D

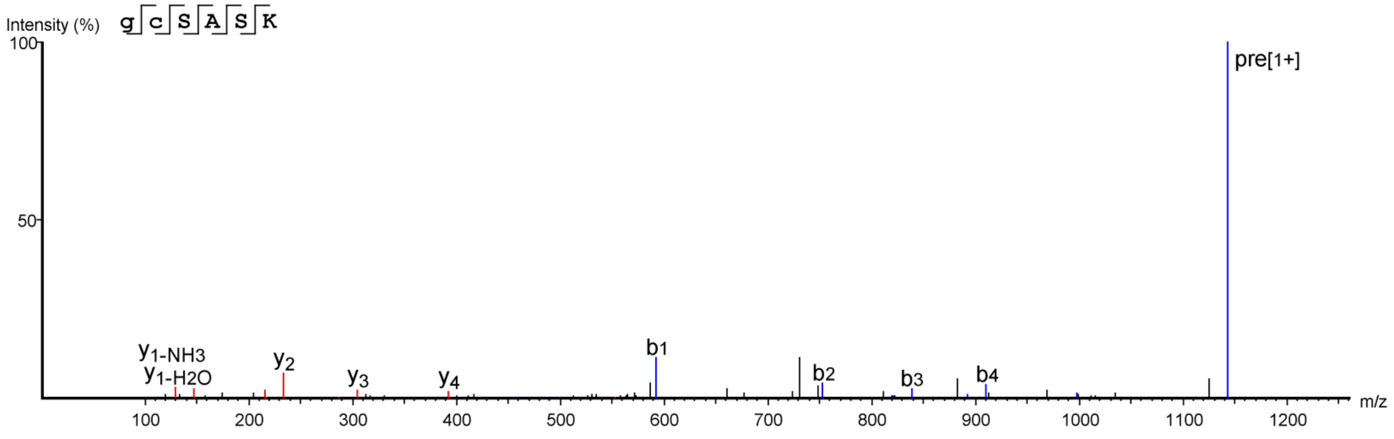

LDBPK_130990.1 (Proteasome regulatory ATPase subunit 2)

Experiment Inhib. Y_A

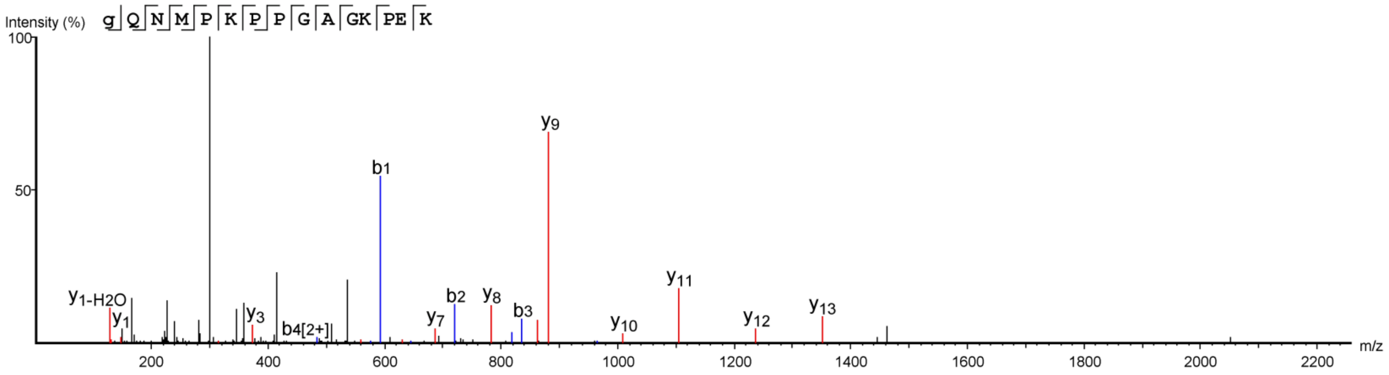

Experiment Inhib. Y_C

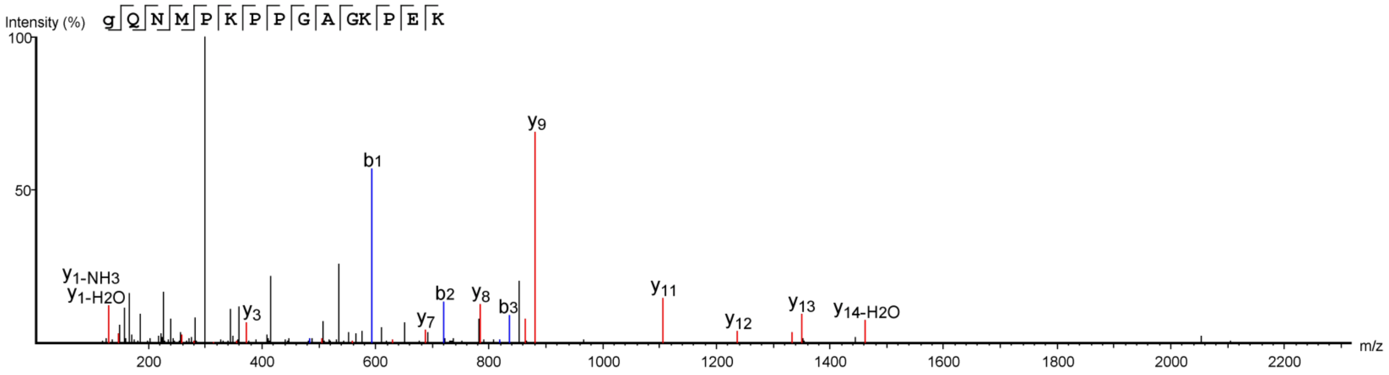

Experiment Inhib. Y_D

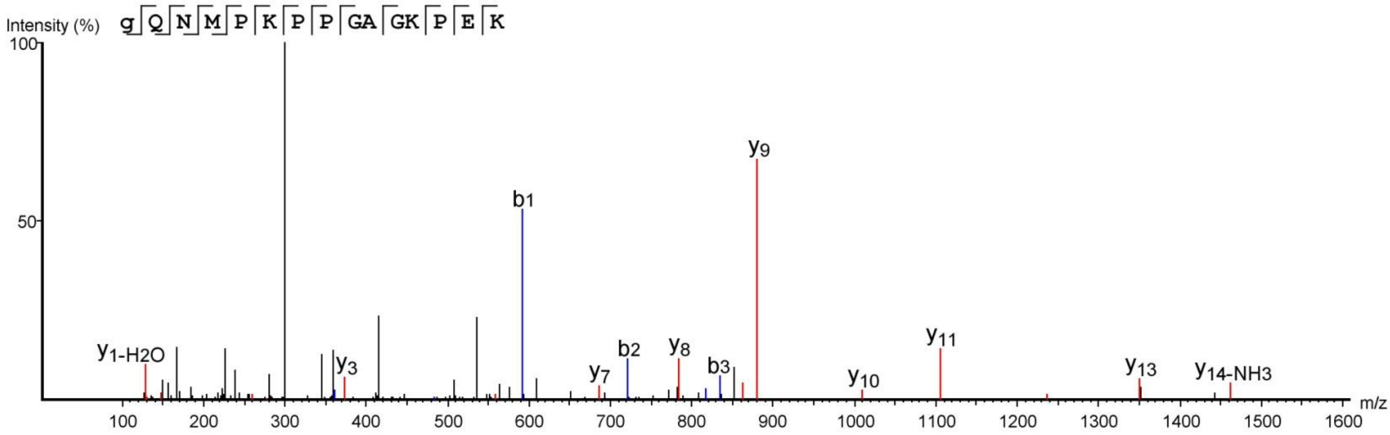

LDBPK_241400.1 (Uncharacterized protein)

Experiment Inhib. Y_A

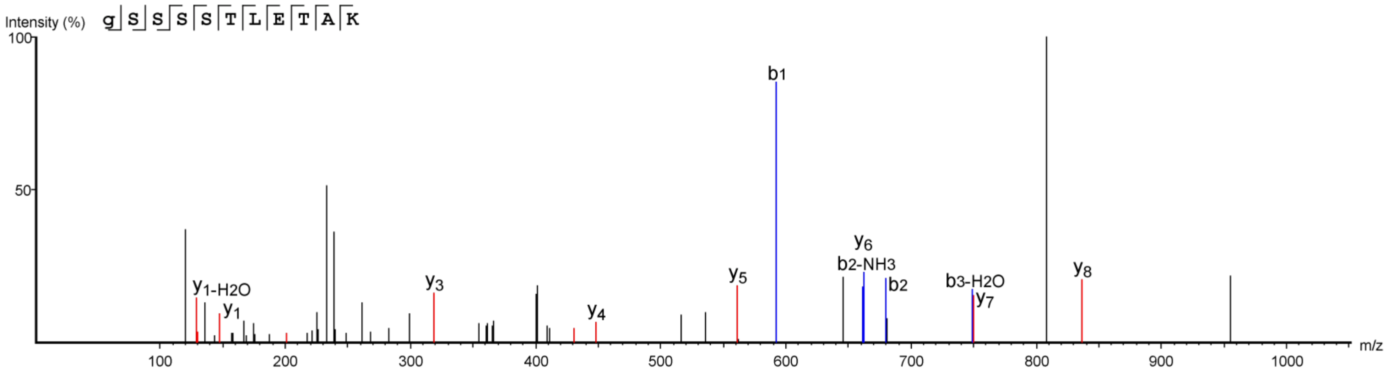

LDBPK_311430.1 (Uncharacterized protein)

Experiment Inhib. Y_C

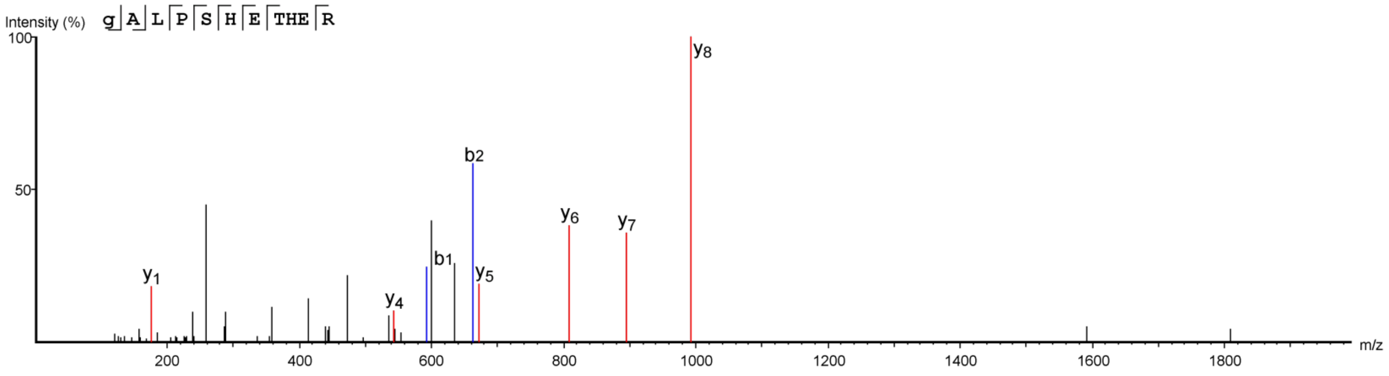

LDBPK_360030.1 (Uncharacterized protein)

Experiment Inhib. Y_C

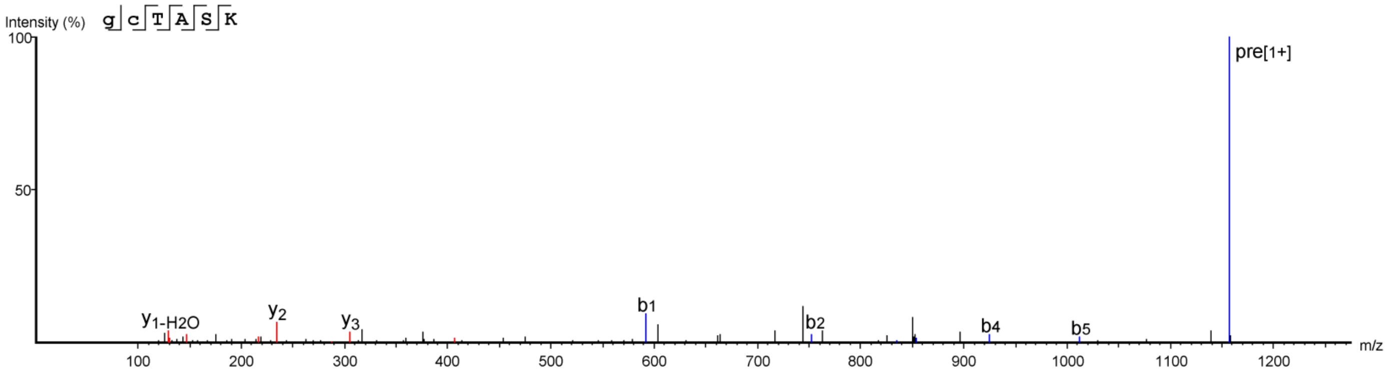

LDBPK_321900.1 (Protein kinase)

Experiment Inhib. Y_C

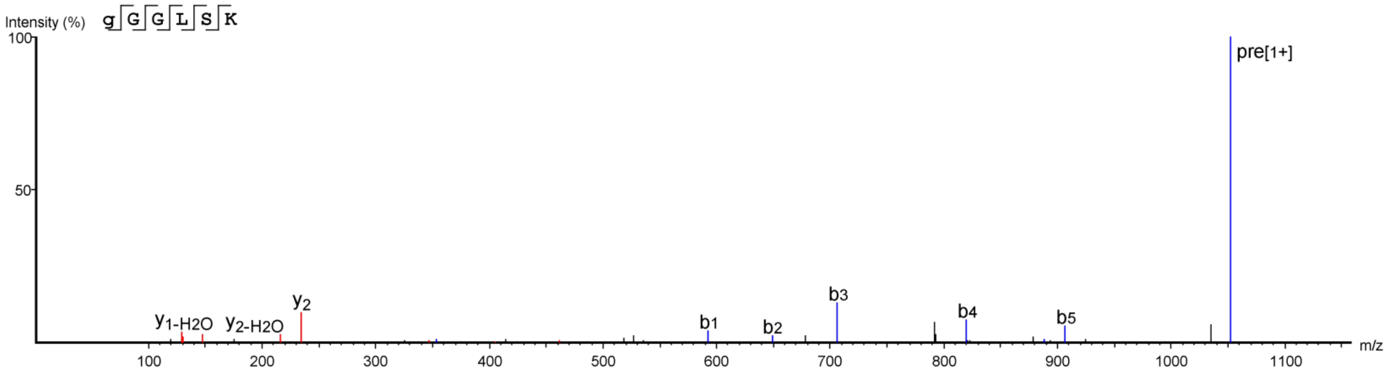

Supplemental Experimental Procedures

Synthesis of AzRB

Capture reagent AzRB was synthesised by solid phase peptide synthesis on a *Biotin-PEG NovaTag™* resin (Novabiochem®). Fmoc-protected amino acids and coupling reagents were sourced from AGTC Bioproducts. Azidoacetic acid was sourced from Sigma Aldrich. Ultrapure water was obtained from a MilliQ Millipore water purification system. LC-MS analysis and purification was performed on a Waters HPLC system fitted with a Waters 515 HPLC pump, Waters 2767 autosampler, Waters XBridge C18 4.6 mm × 100 mm (analytical) or 19mm × 100 mm (preparative) column, Waters 3100 mass spectrometer and Waters 2998 photodiode array. NMR was performed on a Bruker AV-400 instrument. High resolution mass spectrometry was performed by the Imperial College Department of Chemistry Mass Spectrometry Service.

The resin (208 mg, 100 µmol) was placed in a fritted syringe and swollen in DMF for 30 min before Fmoc deprotection in 20% piperidine/DMF (3 mL, 3 x 3 min). The resin was washed in DMF (3 x 3mL), DCM (3 x 3 mL) and DMF (3 x 3mL) after which Fmoc-ε-Ahx-OH (177 mg, 500 µmol), Fmoc-Gly-OH (149 mg, 500 µmol), Fmoc-Arg(Pbf)-OH (324 mg, 500 µmol), Fmoc-Ala-OH (156 mg, 500 µmol) and azidoacetic acid (37 µL, 500 µmol) were double-coupled sequentially. The coupling of Fmoc-ε-Ahx-OH was performed using HATU (186 mg, 490 µmol) and DIPEA (174 µL, 1.0 mmol) and for all other residues HBTU (186 mg, 490 µmol) and DIPEA (174 µL, 1.0 mmol) were used. Each coupling was performed as follows: the amino acid and coupling reagents were dissolved in DMF (2 mL) and activated for 5 min before addition to the resin. The resin was shaken for 1-3 hours, washed in DMF (3 x 3mL), DCM (3 x 3 mL) and DMF (3 x 3 mL). A fresh batch of activated amino acid was added and the resin shaken for a further 1-3 hours. After washing, Fmoc deprotection was performed as above, followed by washing. The procedure was then sequentially repeated for each residue, finishing with the coupling of the azidoacetic acid moiety, which required no Fmoc deprotection. After the final coupling the resin was washed in DMF (3 x 3 mL), DCM (3 x 3 mL), MeOH (3 x 3 mL) and Et_2O (3 x 3 mL) and dried under vacuum overnight. The capture reagent was cleaved from the resin by incubation in a solution of 95% TFA, 2.5% TIS and 2.5% DTT (3 mL) for 3 hours. The cleavage mixture containing the cleaved product was transferred into a 15 mL falcon tube, and the resin washed twice with cleavage mixture (0.5 mL). The solution was concentrated to a volume of 1 mL under a stream of nitrogen, after which the product was precipitated by addition of ice-cold *tert*-butyl methyl ester (TBME) (10 mL). The product was pelleted by centrifugation. The pellet was washed twice with TBME (10 mL) and dried overnight in a vacuum dessicator. The pellet was resuspended in ultrapure water and purified by preparative RP-HPLC in a gradient of methanol and water both supplemented with 0.1% formic acid (0-10 min 5-98% MeOH, 10-12 min 98% MeOH). The fractions containing product were combined and the solvents removed by speedvac. The product was resuspended in ultrapure water (1 mL) and lyophilised to dryness to yield a white solid (24.3 mg, 26 µmol, 26%).

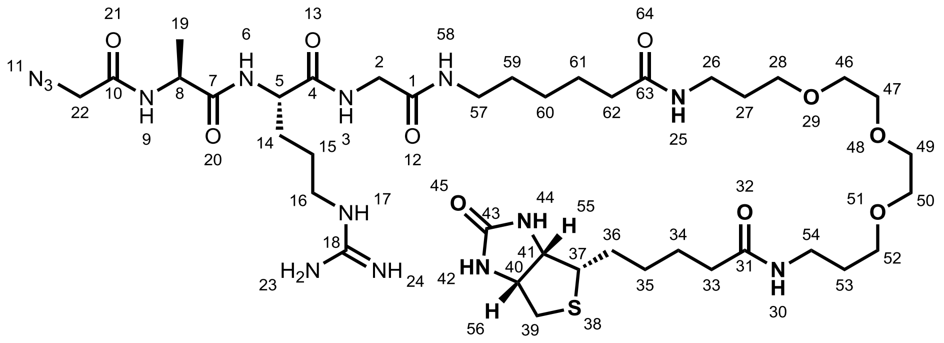

δ_H/ppm (400 MHz, D_2O) 8.39 (s, 1H), 4.58 (dd, J = 7.9, 4.8 Hz, 1H, *H56*), 4.40 (dd, J = 8.0, 4.4 Hz, 1H, *H55*), 4.36 – 4.23 (m, 2H, *H8* & *H5*), 4.02 (s, 2H, *H22*), 3.93 – 3.77 (m, 2H, *H2*), 3.73-3.59 (m, 8H), 3.55 (2xt, J = 6.4, 4H), 3.31 (dt, J = 9.7, 5.1 Hz, 1H, *H37*), 3.27 – 3.09 (m, 8H), 2.97 (dd, J = 13.1, 5.0 Hz, 1H, ½*H39*), 2.75 (d, J = 13.2 Hz, 1H, ½*H39*), 2.22 (2xt, J = 7.6 Hz, 4H), 2.03 – 1.17 (m, 23H); δ_C/ppm (100 MHz, CD_3OD) 175.99, 175.94, 175.34, 174.14, 171.21, 170.52, 170.26, 166.09 (6 x C=O, 1 x C=O tautomer), 158.65 (*C17*), 71.53 (2xC), 71.23 (2xC), 69.91 (2xC), 63.39, 61.62, 57.04, 54.53, 52.52, 50.98, 43.39, 41.95, 41.06, 40.30, 37.82 (2xC), 37.02, 36.86, 30.45 (2xC), 29.99, 29.82, 29.59, 29.53, 27.49, 26.92, 26.68, 26.15, 17.66; HRMS, *m/z* (ESI) found 927.5200 ($C_{39}H_{71}N_{14}O_{10}S$, $[M + H]^+$, requires 927.5198).

Parasite culture (extra detail)

Animal experiments were approved by the University of York Animal Procedures and Ethics Committee and performed under UK Home Office licence (‘Immunity and Immunopathology of Leishmaniasis’ Ref # PPL 60/3708).

The Ethiopian strain of *Leishmania donovani* (MHOM/ET/67/HU3, also known as LV9) was maintained by serial passage in Rag-$2^{-/-}$ mice. Rag-$2^{-/-}$ mice were bred in-house, housed under specific pathogen-free conditions and used at 6–12 weeks of age. Amastigotes were extracted from spleen as described previously (Smelt et al., 1997): briefly, the spleen was removed into unsupplemented RPMI, transferred to a homogeniser and topped up with RPMI medium (approx. 40 mL). The spleen was homogenised to a single cell suspension, transferred to a tube and centrifuged at 800 rpm for 5 minutes; the supernatant was retained. A 50 mL falcon tube was coated with 25 mg saponin per 40 mL supernatant, the supernatant poured in, mixed and left to stand at RT for 5 min. The tube was centrifuged at 3100 rpm for 10 min and supernatant discarded. The pellet was washed 3× by resuspending in 25 mL RPMI and centrifuging at 3100 rpm for 10 minutes. The washed pellet was resuspended in 20 mL RPMI with 10% FCS.

Mice were infected with 3×10^7 *L. donovani* amastigotes intravenously (i.v.) via the tail vein in 200 µl of RPMI 1640 (GIBCO, Paisley, UK).

Promastigotes were obtained by transforming 1×10^7 freshly isolated amastigotes in promastigotes medium (St-Denis et al., 1999) at 26 °C (~2 days for transformation). Parasites were maintained in T25 flasks with non vented lids containing 10 mL medium and passaged twice a week for up to 10 passages.

RPMI medium for *L. donovani* promastigote culture (St-Denis et al., 1999): RPMI 1640 (Sigma #R6748), containing 20 % FCS, 1 % Pen/Strep, 100 μM adenine, 20 mM MES (pH 5.5 with NaOH), 5 μM hemin, 3 μM biopterin and 1 μM biotin.

Metabolic tagging experiments (extra detail)

Promastigotes were pelleted by centrifugation at 800 ×g, 15 min, 26 °C, and resuspended in RPMI/10% FCS containing the appropriate probe (50 μM YnMyr, or myristic acid, 100 μM AHA etc, unless otherwise indicated; from 100 mM stocks) at a parasite density of 7.5×10^7 parasites/mL. Cultures were incubated at 26 °C for 12 hours (or as indicated). Cells were pelleted by centrifugation 800 ×g, 15 min, 4 °C, washed twice with cold PBS then pelleted at 3200 rpm for 10 min at 4°C. After removal of the supernatant, cells were lysed in lysis buffer (1 % NP40, 1% sodium deoxycholate, 0.5 % SDS, 50 mM Tris pH 7.4, 150 mM NaCl, EDTA-free protease inhibitor, Roche) at 1×10^9 parasites/mL by sonication on ice (4× 10 sec burst, amplitude 45 with 1 min interval). Insoluble material was separated by centrifugation at 16,100 ×g for 30 min at 4°C, the supernatant transferred to fresh tubes and snap frozen in liquid N_2, then stored at -80 °C. Tagging of *ex vivo* amastigotes was carried out as above but parasites were cultured at 37 °C.

For inhibition studies, the appropriate inhibitor was pre-incubated with parasite culture for 1 h at 37°C, parasites pelleted by centrifugation at 2200 ×g, 10 min, supernatant removed and parasites resuspended in fresh medium containing inhibitor plus probe (YnMyr, myristic acid etc).

Ex vivo amastigotes inhibition assay

As described in (Hutton et al., 2014). Briefly, *L. donovani* amastigotes (MHOM/ET/67/L28/LV9) isolated from the spleens of infected Rag-$2^{-/-}$ mice were placed in 96 well plates at a density of 4×10^5 *ex vivo* amastigotes per well in a total volume of 200 μL of supplemented RPMI 1640 medium (see 'promastigote medium', above) containing test compounds diluted over a threefold range. After incubation for 72 hr at 26 °C, 20 μL of an Alamar blue solution (Trek Diagnostics) was added and plate incubated for a further 6 hr at 26°C. Fluorescence per well was measured using a POLARstar Optima instrument (BMG labtech; ex.: 544 nm, em.: 590 nm). All assays were run in triplicate and the data analysed by a standard package (GraphPad Prism v5).

Macrophage cytotoxicity assay

As described in (Hutton et al., 2014). Briefly, compounds were tested in parallel against mouse primary macrophages (Alamar blue assay) in triplicate, and analysed as above. In brief, primary macrophages derived from BALB/c bone marrow were seeded into 96 well plates at 4.2×10^4 macrophages/well in a total volume of 200 μL (DMEM (Invitrogen, UK), 10% FCS, 4% L-929 cell conditioned supernatant). Compounds diluted over a threefold range were added, with assays run in duplicate. After incubation for 72 hr at 37°C/5% CO_2, 20 μL Alamar blue was added and the plate incubated for a further 6 hr at 37°C/5% CO_2, prior to fluorescence measurements as above.

CuAAC labeling and pull-down

Proteins were precipitated with chloroform/methanol (MeOH:$CHCl_3$:ddH_2O 4:1:3), or acetone (4 vol. -20 °C 1 h) and then resuspended at 1 mg/mL in 1 % SDS in PBS. This precipitation step was found to increase labeling intensity after CuAAC, likely due to the presence of probe-incorporating glycolipids in the lysates (see main text). Premixed click reagents (100 µM AzTB, 1 mM $CuSO_4$, 1 mM TCEP, 100 µM TBTA, final concentrations) were added as described previously (Heal et al., 2012) and samples vortexed for 1 h RT, then quenched by the addition of 10 mM EDTA. Proteins were precipitated again with MeOH/$CHCl_3$, washed with ice-cold MeOH, air-dried for ~15 min, and then resuspended in 2 % SDS, 10 mM EDTA in PBS. For direct gel analysis, 4 x sample loading buffer (NuPAGE LDS sample buffer) with 2-mercaptoethanol (4 % final) was added and proteins heated for 3 min at 95 °C prior to SDS-PAGE.

For hydroxylamine (NH_2OH) and NaOH treatment, samples were resuspended after CuAAC as above, then 1 M NH_2OH pH7 or 0.2M NaOH added and samples incubated at RT for 1 hour. Samples were quenched by addition of 4 x sample loading buffer. Note that addition of EDTA to the samples before NH_2OH treatment is important to avoid sample degradation.

For pull-down, protein was resuspended at 10 mg/mL in 2 % SDS, 10 mM EDTA in PBS, and then diluted to 1 mg/mL with PBS. DTT (from a fresh 100 × stock in water) was added to give a final concentration of 1 mM. Proteins were incubated with Dynabeads® MyOne™ Streptavidin C1 (pre-washed 3 × 0.2 % SDS in PBS) for 1.5-2 h at RT with rotation. Following removal of the supernatant, beads were washed with 3 × 1 % SDS in PBS, then boiled for 10 mins in sample loading buffer elute bound proteins.

Gel and Western blot analysis

Samples were separated by SDS-PAGE and the gel soaked in fixing solution (10 % AcOH, 40 % MeOH), then rinsed in water for in-gel fluorescent imaging: gels were scanned with Cy3 filters to detect the TAMRA fluorophore using an Ettan DIGE scanner, GE Healthcare. Molecular weight markers (Precision Plus All Blue Standards, Bio-Rad) were detected with Cy5 filters.

For Western blot, proteins were transferred from gels to PVDF membrane (Immobilon-P^{SQ}, Millipore) using a semi-dry system (Invitrogen). Following blocking (5 % milk in TBS, 1% Tween), membranes were incubated with primary antibody for 1 hour at room temperature or overnight at 4°C in blocking solution, then incubated with secondary antibody (goat anti-rabbit IgG-HRP, Invitrogen, 1:10000) for 1 hr in blocking solution. Detection was carried out using Luminata Crescendo Western HRP substrate (Millipore) according to the manufactures instructions and on a Fujifilm LAS 3000 imager. Primary antibodies, LdHASPB (rabbit; ab 2-AE) (Alce et al., 1999), LmHASPB (rabbit, 336) (Alce et al., 1999), GP63 (rabbit, polyclonal; provided by R. McMaster, University of British Columbia) (Frommel et al., 1990), GFP (mouse, Santa Cruz), were used at 1:1000-2000.

Proteomics experiments

Pull-down and preparation of peptides for MS. Proteins were captured by CuAAC as before with the following modifications: CuAAC reaction was carried out for 2 hours and, for some samples, with AzRB in place of AzTB. When AzRB was used, proteins were precipitated following CuAAC via a modified chloroform/methanol precipitation procedure: 4 volumes of MeOH, 1 vol. $CHCl_3$, 3 vol. H_2O were added to the sample, which was

centrifuged at 17,000 ×g for 5 min to pellet proteins at the interface. Both layers were then removed simultaneously, the pellet resuspended in 0.2 % SDS/PBS to the original volume and the precipitation procedure repeated. The pellet was then washed twice with MeOH. Resuspension and affinity enrichment were performed for all samples as before with the following modifications: NeutrAvidin agarose resin (Thermo Scientific, pre-washed 3 × 0.2 % SDS in PBS; typically 50 µL of bead slurry was used for 0.5 mg of lysate) was used in place of the magnetic Dynabeads due to increased stability of the resin during on-bead reduction and alkylation of proteins. Beads were stringently washed following pull-down: 3 × 1 % SDS in PBS, 3 × 4M Urea in 50 mM PBS, 5 × AMBIC (50 mM ammonium bicarbonate). For a 50 µL bed of beads resuspended in 50 µL AMBIC, samples were reduced (5 µL of 100 mM DTT in 50 mM AMBIC) at 60 °C for 30 minutes and allowed to cool to room temperature. The beads were washed with 2 x AMBIC. Cysteines were alkylated (5 µL of 100 mM iodoacetamide in AMBIC) at room temperature for 30 min in the dark. The beads were washed with 2 x AMBIC. Trypsin (3 µg Sequencing Grade Modified Trypsin (Promega) dissolved at 0.2 µg/µL in AMBIC per mg starting lysate) was added to the beads and samples were placed on a shaker and digested overnight at 37 °C. The samples were centrifuged and the supernatant was transferred into clean tubes. The beads were washed twice with 0.1% aqueous formic acid, and these washes were combined with the first supernatant. The solutions were stage-tipped according to a published protocol (Rappsilber et al., 2003). Elution from the sorbent (SDC-XC from 3M) with 70 % acetonitrile in water was followed by speed-vac-assisted solvent removal, reconstitution of peptides in 0.5 % TFA, 2 % acetonitrile in water, and transferred into LC-MS sample vials.

LC-MS/MS analysis. The analysis was performed using an Acclaim PepMap RSLC column 50 cm × 75 µm inner diameter (Thermo Fisher Scientific) using a 2 h acetonitrile gradient in 0.1 % aqueous formic acid at a flow rate of 250 nL/min. Easy nLC-1000 was coupled to a Q Exactive mass spectrometer via an easy-spray source (all Thermo Fisher Scientific). The Q Exactive was operated in data-dependent mode with survey scans acquired at a resolution of 75,000 at m/z 200 (transient time 256 ms). Up to 10 of the most abundant isotope patterns with charge +2 or higher from the survey scan were selected with an isolation window of 3.0 m/z and fragmented by HCD with normalized collision energies of 25. The maximum ion injection times for the survey scan and the MS/MS scans (acquired with a resolution of 17 500 at m/z 200) were 20 and 120 ms, respectively. The ion target value for MS was set to 10^6 and for MS/MS to 10^5, and the intensity threshold was set to 8.3×10^2.

Data searching and analysis. The data were processed with MaxQuant version 1.3.0.5, and the peptides were identified from the MS/MS spectra searched against TriTrypDB-6.0 L. donovani LdBPK282A1 database using the Andromeda search engine. The protein HASPB was not present in this database and so the Uniprot sequence (O77300_LEIDO) was appended to the FASTA file. Cysteine carbamidomethylation was used as a fixed modification, and methionine oxidation and N-terminal acetylation as variable modifications. The false discovery rate was set to 0.01 for peptides, proteins and sites. Other parameters were used as pre-set in the software. “Unique and razor peptides” mode was selected to allow for protein grouping; this calculates ratios from unique and razor peptides (razor peptides are uniquely assigned to protein groups and not to individual proteins). Data were elaborated using Microsoft Office Excel 2007 and Perseus versions 1.4.0.20 and 1.5.0.31. Label free quantification (LFQ) experiments in MaxQuant were performed using the built-in label-free quantification algorithm (Cox et al., 2014).

Modified peptide identification. MS data were processed with PEAKS7 suite (Zhang et al., 2012). The data were searched against a reference *L. donovani* database from Uniprot (27/11/2014). Trypsin (specific, up to three missed cleavages allowed) was selected for database searches, and no enzyme was chosen for de novo searches. The maximal mass error was set to 5 ppm for precursor ions and 0.01 Da for product ions. Carbamidomethylation was selected as a fixed modification, and methionine oxidation as well as the lipid-derived adduct (+534.3278 Da) to any amino acid at peptide N-terminus were set as variable modifications. The maximal number of modifications per peptide was set as five. The false discovery rate was set to 0.01 for peptides, and b1 ions were required for N-terminally modified peptides. Within PEAKS, a module called SPIDER searches for point mutations to further enhance the discovery (Ma and Johnson, 2011). All peptides reported are N-terminal peptides unique for the protein that they were assign to by the software.

Data processing: YnMyr tagged proteins in amastigotes and promastigotes. Amastigote experiments: four replicates were performed independently, starting from the same metabolically labelled lysates (Myr and YnMyr samples) through the sample preparation steps and LC-MS/MS analysis. The replicates were grouped together (groups: Myr, YnMyr). YnMyr protein group was filtered to require three valid values across the four replicates. Label free intensities were logarithmized (base 2) and empty values were imputed with random numbers from a normal distribution, whose mean and standard deviation were chosen to simulate low abundance values close to noise level (impute criteria: width 0.1 and down shift 1.8). A modified t-test with permutation based FDR statistics was applied (250 permutations; FDR = 0.05; S0 = 1). Imputing missing values enables assessment of whether a protein is enriched above background noise levels and facilitates statistical analyses, but is likely to generate false negatives where a genuine hit protein is of low abundance in the sample. To explore this possibility empty values were reinstated and data analysed to assess whether a protein was absent from the control but still met the other criteria (present in three out of four amastigote replicates). Proteins absent from the control sample but not reaching significance were designated 'low abundance hits'. Proteins reaching significance in the t-test and absent from the control sample were designated as 'high confidence hits'.

Promastigote experiments: three replicates were performed independently, starting from the same metabolically labelled lysates (Myr and YnMyr samples) through the sample preparation steps and LC-MS/MS analysis. Data were analysed as described above with 3 valid values per group required, imputation (width 0.1 and down shift 1.8), and t-test (250 permutations; FDR = 0.01; S0 = 1).

For the comparison of amastigotes and promastigotes, data were filtered for at least three valid values in at least one group (groups: Am_Myr, Am_YnMyr, Pro_Myr, Pro_YnMyr). Missing values were imputed as above and proteins cross-referenced with the independent analyses described above that defined t-test significant, low abundance and high confidence hits. Datasets were also compared by a two sample t-test: YnMyr intensities of hits (defined as proteins that were either t-test significant or classed as low abundance hits in the independent analyses) were compared by permutation-corrected t-test (250 permutations; FDR 0.05, S0 2) after imputation of missing values (width 0.1, downshift 2.5). These parameters were chosen so that proteins only found in one of the two datasets were significant in the t-test.

Data processing: YnMyr tagging in the presence of NMT inhibitors (amastigotes). The experiment design was as follows: independent biological experiments were carried out where amastigotes were incubated with YnMyr, YnMyr + 0.2 μM **2**, YnMyr + 0.2 μM **1**, or YnMyr + 7 μM **1**. A Myr control was included in one of these experiments. For proteomics analysis, all samples were prepared and processed in duplicate (technical replicates: A&B; C&D). The 'Match between runs' option (time window 2 minutes) in Maxquant was enabled during the searches. Data were grouped, filtered to retain only those proteins present in both biological experiments, and non-specific binders removed (leaving 346 proteins); for the latter step, enrichment over control ('Myr LFQ ratio'), calculated by taking the mean of the YnMyr intensities and dividing by the mean of the Myr intensities, was required to be ≥2. Missing values were imputed from a normal distribution (width 0.1 and down shift 1.8) and modified two sample t-tests with permutation based FDR statistics were applied to assess differences between groups (250 permutations; FDR = 0.001; S0 = 1, unless otherwise indicated). An ANOVA (Benjamini Hochberg correction, FDR=0.001) was also applied to LFQ intensities to compare across all datasets.

Bioinformatics

Modification of proteins was predicted using the following bioinformatic tools:

For *N*-myristoylation: the Myr predictor (MPred) (Maurer-Stroh et al., 2002) and the Myristoylator (Myr) (Bologna et al., 2004).

For *S*-palmitoylation CSS-Palm 3.0 (Ren et al., 2008; Zhou et al., 2006).

For GPI-anchor modification PredGPI (Pierleoni et al., 2008), GPI-SOM (Fankhauser and Maser, 2005) and FragAnchor (Poisson et al., 2007).

Supplemental References

Alce, T.M., Gokool, S., McGhie, D., Stager, S., and Smith, D.F. (1999). Expression of hydrophilic surface proteins in infective stages of Leishmania donovani. Mol. Biochem. Parasitol. *102*, 191-196.

Bologna, G., Yvon, C., Duvaud, S., and Veuthey, A.L. (2004). N-Terminal myristoylation predictions by ensembles of neural networks. Proteomics *4*, 1626-1632.

Cox, J., Hein, M.Y., Luber, C.A., Paron, I., Nagaraj, N., and Mann, M. (2014). Accurate Proteome-wide Label-free Quantification by Delayed Normalization and Maximal Peptide Ratio Extraction, Termed MaxLFQ. Mol. Cell. Proteomics *13*, 2513-2526.

Fankhauser, N., and Maser, P. (2005). Identification of GPI anchor attachment signals by a Kohonen self-organizing map. Bioinformatics *21*, 1846-1852.

Frommel, T.O., Button, L.L., Fujikura, Y., and McMaster, W.R. (1990). The major surface glycoprotein (GP63) is present in both life stages of Leishmania. Mol. Biochem. Parasitol. *38*, 25-32.

Heal, W.P., Wright, M.H., Thinon, E., and Tate, E.W. (2012). Multifunctional protein labeling via enzymatic N-terminal tagging and elaboration by click chemistry. Nat. Protoc. *7*, 105-117.

Hutton, J.A., Goncalves, V., Brannigan, J.A., Paape, D., Wright, M.H., Waugh, T.M., Roberts, S.M., Bell, A.S., Wilkinson, A.J., Smith, D.F., *et al.* (2014). Structure-Based Design of Potent and Selective Leishmania N-Myristoyltransferase Inhibitors. J. Med. Chem.

Ma, B., and Johnson, R. (2011). De novo sequencing and homology searching. Mol. Cell. Proteomics *11*, O111 014902.

Maurer-Stroh, S., Eisenhaber, B., and Eisenhaber, F. (2002). N-terminal N-myristoylation of proteins: prediction of substrate proteins from amino acid sequence. J. Mol. Biol. *317*, 541-557.

Pierleoni, A., Martelli, P.L., and Casadio, R. (2008). PredGPI: a GPI-anchor predictor. BMC Bioinformatics *9*, 392.

Poisson, G., Chauve, C., Chen, X., and Bergeron, A. (2007). FragAnchor: a large-scale predictor of glycosylphosphatidylinositol anchors in eukaryote protein sequences by qualitative scoring. Genomics Proteomics Bioinformatics *5*, 121-130.

Rappsilber, J., Ishihama, Y., and Mann, M. (2003). Stop and go extraction tips for matrix-assisted laser desorption/ionization, nanoelectrospray, and LC/MS sample pretreatment in proteomics. Anal. Chem. *75*, 663-670.

Ren, J., Wen, L., Gao, X., Jin, C., Xue, Y., and Yao, X. (2008). CSS-Palm 2.0: an updated software for palmitoylation sites prediction. Protein Eng Des Sel *21*, 639-644.

Smelt, S.C., Engwerda, C.R., McCrossen, M., and Kaye, P.M. (1997). Destruction of follicular dendritic cells during chronic visceral leishmaniasis. J. Immunol. *158*, 3813-3821.

St-Denis, A., Caouras, V., Gervais, F., and Descoteaux, A. (1999). Role of protein kinase C-alpha in the control of infection by intracellular pathogens in macrophages. J. Immunol. *163*, 5505-5511.

Zhang, J., Xin, L., Shan, B., Chen, W., Xie, M., Yuen, D., Zhang, W., Zhang, Z., Lajoie, G.A., and Ma, B. (2012). PEAKS DB: de novo sequencing assisted database search for sensitive and accurate peptide identification. Mol. Cell. Proteomics *11*, M111 010587.

Zhou, F., Xue, Y., Yao, X., and Xu, Y. (2006). CSS-Palm: palmitoylation site prediction with a clustering and scoring strategy (CSS). Bioinformatics *22*, 894-896.
